# Supplementary material for: Prevalence and risks of tuberculosis multimorbidity in low-income and middle-income countries: a meta-review
Source: BMJ Open. 2022 Sep 28;12(9):e060906. doi: 10.1136/bmjopen-2022-060906 (PMC9528681; doi:10.1136/bmjopen-2022-060906)
Supplement: Supplementary data [file bmjopen-2022-060906supp001.pdf]

Supplemental material

Supplementary Table 1: Search terms used in Medline (run on October 23rd, 2020)

	Searches
1	exp Noncommunicable Diseases/ or ((Non-communicable or Noncommunicable or Non-infectious) adj (disease* or condition* or illness*)).mp.
2	exp Chronic Disease/ or ((chronic or long-term) adj (disease* or condition* or illness*)).mp.
3	exp Heart Diseases/ or (heart adj (disease* or disorder* or failure)).mp. or (cardiac adj (disease* or disorder* or failure)).mp.
4	exp Cardiovascular Diseases/ or (cardiovascular adj (disease* or disorder* or failure)).mp.
5	exp Coronary Disease/ or (coronary adj (disease* or disorder* or failure)).mp.
6	exp Cerebrovascular Disorders/ or (cerebrovascular adj (disease* or disorder* or insufficienc* or occlusion*)).mp. or (vascular adj (disease* or disorder*)).mp. or (carotid* adj (disease* or disorder*)).mp.
7	exp Peripheral Arterial Disease/ or (arter* adj (disease* or disorder*)).mp.
8	exp Rheumatic Heart Disease/ or exp Heart Defects, Congenital/ or (heart adj3 (malform* or defect* or congeni*)).mp.
9	exp Venous Thrombosis/ or ((deep vein or deep venous) adj thrombos*).mp. or phlebothrombos*.mp.
10	exp Pulmonary Embolism/ or (pulmonar* adj (thromboembolism* or embolism* or disease* or disorder*)).mp.
11	exp Stroke/ or stroke.mp.
12	exp Neoplasms/ or Cancer*.mp. or neoplas*.mp. or tumor*.mp.
13	exp Lung Diseases/ or exp Respiratory Tract Diseases/ or exp Lung Diseases, Obstructive/ or ((lung* or respiratory or pulmonar* or airflow or airway) adj2 (disease* or obstruct* or hypersensitiv*)).mp. or exp Asthma/ or asthma*.mp. or exp Pulmonary Disease, Chronic Obstructive/ or exp Respiratory Hypersensitivity/
14	exp Diabetes Mellitus/ or diabet*.mp.
15	exp Autoimmune Diseases/ or ((autoimmun* or auto immun* or autoaggress* or auto aggress*) adj (disorder* or disease*)).mp.
16	exp Metabolic Syndrome/ or exp Metabolic Diseases/ or ((metabolic or insulin resistance) adj (disorder* or disease* or syndrome*)).mp.
17	exp Obesity/ or obes*.mp.
18	exp Osteoporosis/ or osteoporo*.mp. or bone loss.mp. or exp osteolysis/ or osteolysis.mp. or bone resorption.mp.
19	exp Parkinson disease/ or parkinson*.mp. or paralysis agitans.mp.
20	exp Arthritis/ or arthriti*.mp. or polyarthriti*.mp. or rheumarthriti*.mp.
21	exp Kidney Diseases/ or (kidney adj (disease* or disorder*)).mp.
22	exp Liver Diseases/ or (liver adj (disease* or disorder* or dysfunction*)).mp.
23	exp Hypertension/ or high blood pressure*.mp. or hypertens*.mp.
24	exp Hyperlipidemias/ or hyperlipem*.mp. or hyperlipidem*.mp. or lipem*.mp. or lipidem*.mp.
25	exp Hypercholesterolemia/ or ((high* or elevat*) adj cholesterol*).mp. or hypercholesterem*.mp. or hypercholesterolem*.mp.
26	exp Hypertriglyceridemia/ or hypertriglyceridem*.mp.
27	exp Thyroid Diseases/ or (thyroid adj (disease* or disorder*)).mp. or exp Hyperthyroidism/ or hyperthyroid*.mp. or exp Hypothyroidism/ or hypothyroid*.mp. or ((thyroid-stimulating hormone* or tsh) adj deficien*).mp.
28	exp Motor Neuron Disease/ or motor neuron* disease*.mp. or lateral scleros*.mp. or motor system disease*.mp.
29	exp Multiple Sclerosis/ or multiple sclerosis.mp. or disseminated sclerosis.mp.
30	exp Emphysema/ or emphysema*.mp.
31	exp Bronchitis/ or bronchit*.mp.
32	1 or 2 or 3 or 4 or 5 or 6 or 7 or 8 or 9 or 10 or 11 or 12 or 13 or 14 or 15 or 16 or 17 or 18 or 19 or 20 or 21 or 22 or 23 or 24 or 25 or 26 or 27 or 28 or 29 or 30 or 31
33	exp Mental Disorders/ or exp Psychotic Disorders/ or ((mental* or psychiatr* or psycho*) adj (disorder* or disease* or illness*)).mp.
34	exp Depressive Disorder, Major/ or exp Depression/ or Depress*.mp. or MDD.mp.
35	exp Anxiety Disorders/ or exp Anxiety/ or anxi*.mp.

Jarde A, *et al. BMJ Open* 2022; 12:e060906. doi: 10.1136/bmjopen-2022-060906

36	exp Phobic Disorders/ or phobi*.mp.
37	exp Schizophrenia/ or schizophreni*.mp. or hebephreni*.mp.
38	exp Somatoform Disorders/ or ((somatoform* or somati* or medically unexplained or briquet or pain) adj (disorder* or syndrome* or symptom*)).mp. or exp Medically Unexplained Symptoms/
39	exp Dissociative Disorders/ or (dissociative adj (disorder* or hysteri* or reaction*)).mp. or dissociation*.mp.
40	exp Hysteria/ or hysteri*.mp.
41	exp Mood Disorders/ or ((affective* or mood*) adj (disorder* or disease* or illness* or symptom*)).mp.
42	exp Stress Disorders, Post-Traumatic/ or PTSD.mp. or ((post trauma* or posttrauma*) adj (stress* or neurose*)).mp. or combat disorder*.mp. or war disorder*.mp.
43	exp Cognition Disorders/ or ((cognitive or cognition or mental or neurocognitive) adj (dysfunction* or decline* or impairment* or deterioration* or disorder* or illness* or disease*)).mp.
44	exp Personality Disorders/ or personality disorder*.mp.
45	exp "Disruptive, Impulse Control, and Conduct Disorders"/ or impulse control disorder*.mp. or intermittent explosive disorder*.mp.
46	exp "Feeding and Eating Disorders"/ or ((eating or appetite or feeding) adj disorder*).mp.
47	exp Bipolar Disorder/ or ((bipolar or mani*) adj (disorder* or illness* or disease*)).mp.
48	exp Obsessive-Compulsive Disorder/ or OCD*.mp. or ((obsess*-compulsi* or obsess* or compulsi*) adj (disorder* or illness* or disease* or neuros*)).mp.
49	exp Panic Disorder/ or (panic adj (attack* or disorder*)).mp.
50	exp Agoraphobia/ or agoraphobi*.mp.
51	exp Neurotic Disorders/ or neuros*.mp. or neurotic disorder*.mp. or psychoneuros*.mp.
52	33 or 34 or 35 or 36 or 37 or 38 or 39 or 40 or 41 or 42 or 43 or 44 or 45 or 46 or 47 or 48 or 49 or 50 or 51
53	exp Communicable Diseases/ or ((communic* or contag* or transmi* or infect*) adj (disease* or infection* or illness*)).mp.
54	exp Bacterial Infections/ or bacteri* infection*.mp.
55	exp Conjunctivitis/ or conjunctivitis.mp.
56	exp HIV/ or hiv.mp. or Human immuno deficiency virus.mp.
57	exp Acquired Immunodeficiency Syndrome/ or AIDS.mp. or immunodeficiency associated virus.mp. or immun* deficiency associated virus.mp. or acquired immunodeficiency syndrome*.mp. or acquired immun* deficiency syndrome*.mp.
58	exp Buruli Ulcer/ or Bairnsdale.mp. or Buruli.mp.
59	exp Onchocerciasis/ or onchocer*.mp.
60	hepatitis.mp. or exp Hepatitis B/ or exp Hepatitis C/
61	exp Leishmaniasis/ or leishmania*.mp.
62	exp Leprosy/ or lepros*.mp. or hansen*.mp.
63	exp Elephantiasis, Filarial/ or elephantias*.mp. or filaria*.mp.
64	exp Trachoma/ or egyptian ophthalmia*.mp. or trachoma*.mp.
65	exp Chikungunya Fever/ or chickungunya.mp. or chikungunya.mp.
66	exp Taeniasis/ or taenia*.mp.
67	exp Cysticercosis/ or cysticercos*.mp.
68	exp Echinococcosis/ or hydatid*.mp. or echinococc*.mp.
69	exp Chagas Disease/ or trypanosom*.mp. or chagas.mp.
70	exp Trypanosomiasis/ or sleeping sickness.mp.
71	exp Encephalitis, Japanese/ or (japanese adj3 encephalitis).mp.
72	exp Syphilis/ or syphilis.mp.
73	53 or 54 or 55 or 56 or 57 or 58 or 59 or 60 or 61 or 62 or 63 or 64 or 65 or 66 or 67 or 68 or 69 or 70 or 71 or 72
74	exp Tuberculosis/
75	Tuberculos*.mp.
76	TB.mp.

77	koch*.mp.
78	exp Tuberculosis/ or Tuberculos*.mp. or TB.mp. or koch*.mp.
79	(multiple adj (ill* or disease* or condition* or syndrom* or disorder*)).mp.
80	((Cooccur* or co-occur* or coexist* or co-exist* or multipl* or concord* or discord* or long-term or physical*) adj3 (disease* or ill* or care or condition* or disorder* or health* or medication* or symptom* or syndrom*)).mp.
81	(comorbid* or multimorbid* or co-occurren* or co-morbid* or Multidisease* or multi-disease*).mp.
82	(comorbid* or multimorbid* or co-occurren* or co-morbid* or multi-morbid* or Multidisease* or multi-disease*).mp.
83	exp Comorbidity/ or exp Multimorbidity/ or exp Multiple Chronic Conditions/
84	79 or 80 or 81 or 82 or 83
85	exp "Systematic Review"/
86	"systematic review*".m_titl.
87	exp Meta-Analysis/
88	"meta-analys*".m_titl.
89	exp "Systematic Review"/ or "systematic review*".m_titl. or exp Meta-Analysis/ or "meta-analys*".m_titl.
90	32 or 52 or 73
91	(32 or 52 or 73) and 78
92	(32 or 52 or 73) and 78 and 84
93	(32 or 52 or 73) and 78 and 84 and 89
94	exp Animals/ not exp Humans/
95	((32 or 52 or 73) and 78 and 84) not 94
96	((32 or 52 or 73) and 78 and 84 and 89) not 94
97	((32 or 52 or 73) and 78 and 89) not 94

Supplementary Table 2. Reasons for exclusion of references assessed in full text.

**Wrong population**	
1.	Shivakoti, Rupak and Sharma, Davina and Mamoon, Gabeena and Pham, Kiemanh. Association of HIV infection with extrapulmonary tuberculosis: a systematic review. Infection, 2017; 45(1):44501.
2.	Nasiri, Mohammad Javad and Dabiri, Hossein and Darban-Sarokhalil, Davood and Hashemi Shahraki, Abdolrazagh. Prevalence of Non-Tuberculosis Mycobacterial Infections among Tuberculosis Suspects in Iran: Systematic Review and Meta-Analysis. PloS one, 2015; 10(6):e0129073.
3.	Yaghoubi A. and Salehabadi S. and Abdeahad H. and Hasanian S.M. and Avan A. and Yousefi M. and Jamehdar S.A. and Ferns G.A. and Khazaei M. and Soleimanpour S. Tuberculosis, human immunodeficiency viruses and TB/HIV co-infection in pregnant women: A meta-analysis. Clinical Epidemiology and Global Health, 2020; 8(4):1312-1320.
4.	Manrique, RD and CastaÃ±eda, HL. RelaciÃ³n entre haber tenido tuberculosis y desarrollar enfermedad pulmonar obstructiva crÃ³nica. RevisiÃ³n sistemÃ¡tica y meta anÃ¡lisis. Med. U.P.B, 2015; 34(2):115-125.
5.	Fadaee, Manouchehr and Rashedi, Jalil and Arabi, Sepideh and Poor, Behroz Mahdavi and Kafil, Hossein Samadi and Pourostadi, Mahya and Kazemi, Abdolhassan and Asgharzadeh, Mohammad. Stopping of the Downtrend of Tuberculosis in Iran, a Systematic Review of Associated Risk Factors. Infectious disorders drug targets, 2020; 20(3):367-373.
6.	Bailey, S L and Ayles, H. Association between diabetes mellitus and active tuberculosis in Africa and the effect of HIV. Tropical medicine & international health : TM & IH, 2017; 22(3):261-268.
7.	Berhan, Asres and Berhan, Yifru and Yizengaw, Desalegn. A meta-analysis of drug resistant tuberculosis in Sub-Saharan Africa: how strongly associated with previous treatment and HIV co-infection?. Ethiopian journal of health sciences, 2013; 23(3):271-82.
8.	Gao Y. and Liu M. and Chen Y. and Shi S. and Geng J. and Tian J. Association between tuberculosis and COVID-19 severity and mortality: A rapid systematic review and meta-analysis. Journal of Medical Virology, 2020; ():.
9.	Berhan A. and Berhan Y. and Yizengaw D. A meta-analysis of drug resistant tuberculosis in Sub-Saharan Africa: how strongly associated with previous treatment and HIV co-infection?. Ethiopian journal of health sciences, 2013; 23(3):271-282.
10.	Judith Udeh, Jabulani Ncayiyana, Sibusiso Mkwananzi. The prevalence of latent tuberculosis infection and associated risk factors among young people in South Africa: a systematic review and meta-analysis.
11.	Demeke Geremew. Tuberculosis and its association with ART initiation in HIV patients in Ethiopia.
12.	Eleni Seyoum, Alemayehu Worku. The effect of co-infection (hepatitis b or c viruses & tuberculosis) on the outcome of antiretroviral therapy in resource limited countries: systematic review and meta-analysis.
13.	Diana Ramos, Ana Carolina Fragoso Motta, Alan Grupioni LourenÃ§o, Lara Maria Alencar Ramos Innocentini, Maria ConceiÃ§Ã£o Pereira Saraiva. Influence of HIV and tuberculosis co-infection on the occurrence of oral candidiasis: a systematic review and meta analysis.
**Does not report data on people with TB+1 chronic condition**	
14.	Oga-Omenka, Charity and Tseja-Akinrin, Azhee and Sen, Paulami and Mac-Seing, Muriel and Agbaje, Aderonke and Menzies, Dick and Zarowsky, Christina. Factors influencing diagnosis and treatment initiation for multidrug-resistant/rifampicin-resistant tuberculosis in six sub-Saharan African countries: a mixed-methods systematic review. BMJ global health, 2020; 5(7):.
15.	Tamuzi, Jacques L and Ayele, Birhanu T and Shumba, Constance S and Adetokunboh, Olatunji O and Uwimana-Nicol, Jeannine and Haile, Zelalem T and Inugu, Joseph and Nyasulu, Peter S. Implications of COVID-19 in high burden countries for HIV/TB: A systematic review of evidence. BMC infectious diseases, 2020; 20(1):744.
16.	Walaza, Sibongile and Cohen, Cheryl and Tempia, Stefano and Moyes, Jocelyn and Nguweneza, Athermon and Madhi, Shabir A and McMorrow, Meredith and Cohen, Adam L. Influenza and tuberculosis co-infection: A systematic review. Influenza and other respiratory viruses, 2020; 14(1):77-91.
17.	Tola, Habteyes Hailu and Holakouie-Naieni, Kourosh and Lejisa, Tadesse and Mansournia, Mohammad Ali and Yaseri, Mehdi and Tesfaye, Ephrem and Mola, Million. Is hypothyroidism rare in multidrug resistance tuberculosis patients on treatment? A systematic review and meta-analysis. PloS one, 2019; 14(6):e0218487.
18.	Nliwasa, Marriott and MacPherson, Peter and Gupta-Wright, Ankur and Mwapasa, Mphatso and Horton, Katherine and Odland, Jon O and Flach, Clare and Corbett, Elizabeth L. High HIV and active tuberculosis prevalence and increased mortality risk in adults with symptoms of TB: a systematic review and meta-analyses. Journal of the International AIDS Society, 2018; 21(7):e25162.
19.	Seegert, Anneline Borchsenius and Rudolf, Frauke and Wejse, Christian and Neupane, Dinesh. Tuberculosis and hypertension-a systematic review of the literature. International journal of infectious diseases : IJID : official publication of the International Society for Infectious Diseases, 2017; 56():54-61.
20.	Wu, Shan-shan and Zhang, Yue-lun and Wang, Wei-wei and Chen, Ru and Sun, Feng and Zhan, Si-yan. [Liver injury associated with treatment of multidrug-resistant tuberculosis: a systematic review and meta-analysis]. Beijing da xue xue bao. Yi xue ban = Journal of Peking University. Health sciences, 2014; 46(3):417-23.
21.	Patra, Jayadeep and Jha, Prabhat and Rehm, Jurgen and Suraweera, Wilson. Tobacco smoking, alcohol drinking, diabetes, low body mass index and the risk of self-reported symptoms of active tuberculosis: individual participant data (IPD) meta-analyses of 72,684 individuals in 14 high tuberculosis burden countries. PloS one, 2014; 9(5):e96433.
22.	Li, Xin-Xu and Zhou, Xiao-Nong. Co-infection of tuberculosis and parasitic diseases in humans: a systematic review. Parasites & vectors, 2013; 6(101462774):79.
23.	Sathiyamoorthy R. and Kalaivani M. and Aggarwal P. and Gupta S.K. Prevalence of pulmonary tuberculosis in India: A systematic review and meta-analysis. Lung India, 2020; 37(1):45-52.
24.	Zaidi A.K.M. and Awasthi S. and DeSilva H.J. Burden of infectious diseases in South Asia. British Medical Journal, 2004; 328(7443):811-815.
25.	Mortazavi, Hamed and Ghazalibina, Mehran and Mansouri, Shamseddin and Khaledi, Azad and Saburi, Ehsan. Pulmonary Fungal Co-Infection Prevalence among Iranian Patients with Pulmonary Tuberculosis: A Systematic Review and Meta-Analysis. SAINS MALAYSIANA, 2019; 48(12):2717-2725.
26.	Tanya Diefenbach-Elstob, Patricia Graves, Emma McBryde, David Plummer, Jeffrey Warner. Extrapulmonary tuberculosis in a global context: a systematic review of epidemiology and risk factors.
27.	Worku Jimma, Ahmed Abdulahi. Prevalence and risk factors of multidrug-resistant tuberculosis in Iran and its neighboring countries: systematic review and meta-analysis.

28.	Simon Collin, Dominik Zenner, Ibrahim Abubakar, Marieke van der Werf, Sarah Anderson, Gerard de Vries, Knut Lonnroth, Emily Newton. Effectiveness of interventions for TB control and prevention in countries of low and medium TB incidence: a systematic review of reviews.
29.	Yohannes Gezahagn, Yasin Mohammed, Alemseged Abdissa, Sophia Hussien, Guday Emire, Mirgissa Kaba. Delays in the diagnosis and treatment of tuberculosis in Ethiopia: a systematic review.
30.	Victor Vega, Sharon Rodriguez, Larissa Otero, Carlos Seas. Systematic review and meta-analysis on recurrent tuberculosis and associated risk factors.
31.	Mahdi Afshari, Bagheri, Saeed Barzegari. A protocol for estimating the risk factors of treatment default among patients with tuberculosis using systematic review and meta-analysis.
32.	Nguyen Tien Huy, Hieu Truong Hong, Mohammad Rashidul Hashan, Hazem Faraj, Tran Thuy Huong Quynh, Ahmed Saber Abdelrahman, Ahmad Morad, Khaled Mohy Ismaeil, Mohamed Gomaa Kamel. Hyponatremia in tubercular meningitis: a systematic review and meta-analysis.
33.	Trent Herdman, Justine Zhang, Sumona Datta, Matthew Saunders, Marco Tovar, Rosario Montoya, Carlton Evans. Clinical characteristics of unrecognised pulmonary tuberculosis detected through comprehensive population-based prevalence surveys: a systematic review.
34.	Olena Ivanova, Verena Hoffmann, Celso Khosa, Jule Witzleb, Michael Hoelscher, Andrea Rachow. Pulmonary outcomes in patients with tuberculosis: systematic review and meta-analysis.
35.	Shamanthi Jayasooriya, Caroline Mitchell. A systematic review of what proportion of adult patients attending TB treatment programmes in Africa have been identified as not having active tuberculosis?.
36.	Petrus Kosmas, Elize Pietersen, Jabulani Ncayiyana, Mark Engel. Extensively drug resistant tuberculosis in Africa; prevalence and factors associated; a systematic review and meta-analysis.
37.	Rodney Ehrlich, Paula Akugizibwe, Nandi Siegfried, David Rees. Systematic review of association between silica/silicosis and tuberculosis.
38.	Saravanan N, Silambhu Chelvi Ramesh, Rajesh Mondal, Banu Rekha, Basilea Watson, Rajendran Krishnan, Kannan Thiruvengadam. Nutritional status of patients with tuberculosis among the tribal population in India.
39.	Philippe Armel Awana, Celestin Danwang, Joel Noutakdie Tochie, Jean Joel Bigna. Global epidemiology of venous thromboembolism in people with active tuberculosis: a systematic review and meta-analysis.
40.	Sally Hayward, Rachel Wittenberg, Lisa Stockdale. Systematic review of the relationship between Herpesviridae infections and tuberculosis.
41.	Linh Nguyen, Quyen Bui. The incidence rate of multidrug-resistant Tuberculosis/ Rifampicin-resitant Tuberculosis and associated factors: a meta-analysis.
42.	Mpho Refilwe Disang, Christine Campbell, David Weller. Prevalence and patterns of communicable and noncommunicable diseases multimorbidity in sub-Saharan Africa; a protocol for systematic review.
43.	Tamuzi Lukenze Jacques, Peter Nyasulu, Ayele Birhanu. COVID-19 impact on HIV and tuberculosis programmes: a systematic review and meta-synthesis of evidence.
44.	Sara Hussein, May Al-Asmar, Ahmed Awaisu, Yaw Owusu, Alaa Soliman, Sara Murshid, Muna Al-Maslmani, Faraj Howady. Prevalence and Risk Factors of Drug Resistant Tuberculosis in the Eastern Mediterranean Region: A Systematic Review.
45.	Nannan Wang, Jia Zhu, Wenpei Liu, Miaomiao Yang, Honggang Yi, Shaowen Tang. Incidence, temporal trend and factors associated with anti-tuberculosis drug-induced liver injury: A systematic review and meta-analysis.
46.	Tasmiya Ira, Elizabeth Ojewole, Pravina Laljeeth, Richard Beharilal. Determinants of adverse drug reactions due to tuberculosis therapy in African countries: a systematic review.
47.	Danwang, Celestin and Bigna, Jean Joel and Awana, Armel Philippe and Nzalie, Rolf Nyah-Tuku and Robert, Annie. Global epidemiology of venous thromboembolism in people with active tuberculosis: a systematic review and meta-analysis. Journal of thrombosis and thrombolysis, 2020; ():.
48.	Dessie, Getenet and Negesse, Ayenew and Wagnew, Fasil and Amare, Desalegne and Tiruneh, Balew Zeleke and Mulugeta, Henok and Mekonen, Berhanu Abebaw and Haile, Dessalegn and Ayalew, Tilksew and Habtewold, Tesfa Dejenie. Intestinal parasites and HIV in Ethiopian tuberclosis patients: A systematic review and meta-analysis. Current therapeutic research, clinical and experimental, 2020; 93():100603.
49.	Hadadi-Fishani, Mehdi and Shakerimoghaddam, Ali and Khaledi, Azad. Candida coinfection among patients with pulmonary tuberculosis in Asia and Africa; A systematic review and meta-analysis of cross-sectional studies. Microbial pathogenesis, 2020; 139():103898.
50.	Alemu, Ayinalem and Bitew, Zebenay Workneh and Worku, Teshager. Intestinal parasites co-infection among tuberculosis patients in Ethiopia: a systematic review and meta-analysis. BMC infectious diseases, 2020; 20(1):510.
51.	Barzegari, Saeed and Afshari, Mahdi and Movahednia, Mahtab and Moosazadeh, Mahmood. Prevalence of anemia among patients with tuberculosis: A systematic review and meta-analysis. The Indian journal of tuberculosis, 2019; 66(2):299-307.
52.	Girum, Tadele and Muktar, Ebrahim and Lentiro, Kifle and Wondiye, Habtamu and Shewangizaw, Misgun. Epidemiology of multidrug-resistant tuberculosis (MDR-TB) in Ethiopia: a systematic review and meta-analysis of the prevalence, determinants and treatment outcome. Tropical diseases, travel medicine and vaccines, 2018; 4(101674442):5.
53.	Keflie, Tibebeselassie Seyoum and Nolle, Nils and Lambert, Christine and Nohr, Donatus and Biesalski, Hans Konrad. Vitamin D deficiencies among tuberculosis patients in Africa: A systematic review. Nutrition (Burbank, Los Angeles County, Calif.), 2015; 31(10):1204-12.
54.	Allwood, Brian W and Myer, Landon and Bateman, Eric D. A systematic review of the association between pulmonary tuberculosis and the development of chronic airflow obstruction in adults. Respiration; international review of thoracic diseases, 2013; 86(1):76-85.
55.	Azzeri A. and Ching G.H. and Jaafar H. and Noor M.I.M. and Razi N.A. and Then A.Y.-H. and Suhaimi J. and Kari F. and Dahlui M. A review of published literature regarding health issues of coastal communities in Sabah, Malaysia. International Journal of Environmental Research and Public Health, 2020; 17(5):1533.
56.	Hosseini M. and Shakerimoghaddam A. and Ghazalibina M. and Khaledi A. Aspergillus coinfection among patients with pulmonary tuberculosis in Asia and Africa countries; A systematic review and meta-analysis of cross-sectional studies. Microbial Pathogenesis, 2020; 141():104018.
57.	Cormier M. and Schwartzman K. and N'Diaye D.S. and Boone C.E. and dos Santos A.M. and Gaspar J. and Cazabon D. and Ghiasi M. and Kahn R. and Uppal A. and Morris M. and Oxlade O. Proximate determinants of tuberculosis in Indigenous peoples worldwide: a systematic review. The Lancet Global Health, 2019; 7(1):e68-e80.

58.	Azhar G. DOTS for TB relapse in India: A systematic review. Lung India, 2012; 29(2):147-153.
59.	Flannery E. and Rosenthal M. and Roberts E.R. and Henderson J.J. and Davis B.M. and Foxman B. Synergy and anergy in the context of infectious disease epidemiology: The role of co-infection. American Journal of Epidemiology, 2011; 173():S234.
60.	Khadija Ibrahim, Nan Shwe Htun, Karim Manji, Peter Odermatt, Marcel Tanner, JÃ¼rg Utzinger, Nicole Probst Hensch, Lukas Fenner. Does the association between helminths and tuberculosis have influence on each other?.
61.	Katherine Horton, Peter MacPherson, Richard White, Rein Houben, Liz Corbett. Gender differences in tuberculosis prevalence in low- and middle-income countries.
62.	Dalya Eltayeb, Elize Pietersen, Mark Engel, Leila Abdullahi. Factors associated with patient and health system delay in diagnosis and treatment for pulmonary tuberculosis in Middle East and North Africa (MENA).
63.	Erick Bunyasi, Leila Abdullahi, Bey-Marrie Schmidt, Hennie Geldenhuys, Robin Wood, Mark Hatherill. A systematic review on the prevalence and incidence of tuberculosis disease and infection prevention and control measures among high school students in 22 high tuberculosis disease burden countries defined by the World Health Organisation.
64.	Anthony Byrne, Ben Marais, Carole Mitnick, Leonid Lecca, Guy Marks. Tuberculosis and asthma: a systematic review.
65.	Amare Tariku, Destaw Fetene. The effect of under-nutrition on non-adherence to anti-TB drugs in TB patients in Sub-Saharan Africa: a systematic review and meta-analysis.
66.	Habteyes Tola, Kourosh Holakouie-Naieni, Ephrem Tesfaye, Mohammad Mansournia, Mehdi Yaseri. Tuberculosis treatment interruption and its associated factors in Ethiopia: a systematic review and meta-analysis.
67.	Ahmed Hossain, Zeeba Sultana, Farhana Hoque. Association between HIV infection and multidrug-resistant tuberculosis in Africa and Asia: a systematic review and meta-analysis.
68.	Ayinalem Alemu Shitie, Zebenay Workneh Bitew, Teshager Worku. Intestinal parasites co-infection among tuberculosis patients in Ethiopia: a systematic review and meta-analysis.
69.	Birhan Alemnew, Setegn Eshetie, Asmamaw Demis. The prevalence and its associated risk factors of extra pulmonary tuberculosis in Ethiopia; systematic review and meta-analysis.
70.	Getu Diriba, Habteyes Tola, Ayinalem Alemu, Abebaw Kebede. Prevalence of drug resistance and its risk factors among extra pulmonary tuberculosis patients in Ethiopia: a systematic review and meta-analysis.
71.	Balew Arega. Prevalence rate of undiagnosed tuberculosis in the community in Ethiopia from 2001 to 2014: systematic review and meta-analysis.
72.	Kerri Viney, Luis Furuya-Kanamori, Kefyalew Addis Alene, Kinley Wangdi, Anthony Byrne, Justin Clark. Types and burden of disabilities associated with tuberculosis: protocol for a systematic review.
73.	Getahun Molla Kassa, Atalay Goshu Muluneh, Dawit Tefera Fentie, Mehari Woldemariam Merid. Magnitude and effect of anemia on treatment outcome of drug-resistance tuberculosis in Sub-Sahara Africa: systematic review and meta-analysis.
74.	Adam Wondmieneh, Getnet Gedefaw, Addisu Getie, Asmamaw Demis. Undernutrition among adult tuberculosis patients in Ethiopia: A Systematic review and meta- analysis.
75.	Badawi A. and Gregg B. and Vasileva D.. Systematic analysis for the relationship between obesity and tuberculosis. Public Health, 2020; 186():246-256.
**Does not report data on any of our outcomes of interest**	
76.	Lohiya, Ayush and Suliankatchi Abdulkader, Rizwan and Rath, Rama Shankar and Jacob, Olivia and Chinnakali, Palanivel and Goel, Akhil Dhanesh and Agrawal, Sumita. Prevalence and patterns of drug resistant pulmonary tuberculosis in India-A systematic review and meta-analysis. Journal of global antimicrobial resistance, 2020; 22(101622459):308-316.
77.	Noykhovich, Ekaterina and Mookherji, Sangeeta and Roess, Amira. The Risk of Tuberculosis among Populations Living in Slum Settings: a Systematic Review and Meta-analysis. Journal of urban health : bulletin of the New York Academy of Medicine, 2019; 96(2):262-275.
78.	Tola, Habteyes Hailu and Tol, Azar and Shojaeizadeh, Davoud and Garmaroudi, Gholamreza. Tuberculosis Treatment Non-Adherence and Lost to Follow Up among TB Patients with or without HIV in Developing Countries: A Systematic Review. Iranian journal of public health, 2015; 44(1):44501.
79.	Naing, Cho and Mak, Joon Wah and Maung, Mala and Wong, Shew Fung and Kassim, Ani Izzuani Binti Mohd. Meta-analysis: the association between HIV infection and extrapulmonary tuberculosis. Lung, 2013; 191(1):27-34.
80.	Tengan F.M. and Figueiredo G.M. and Leite O.H.M. and Nunes A.K.S. and Manchiero C. and Dantas B.P. and Magri M.C. and Barone A.A. and Bernardo W.M. Prevalence of multidrug-resistant tuberculosis in Latin America and the Caribbean: a systematic review and meta-analysis. Tropical Medicine and International Health, 2020; 25(9):1065-1078.
81.	Xia Y.Y. and Zhan S.Y. Systematic review of anti-tuberculosis drug induced adverse reactions in China. Zhonghua jie he he hu xi za zhi = Zhonghua jiehe he huxi zazhi = Chinese journal of tuberculosis and respiratory diseases, 2007; 30(6):419-423.
82.	Perez-Guzman C. and Vargas M.H. and Torres-Cruz A. and Villarreal-Velarde H. Does aging modify pulmonary tuberculosis?: A meta-analytical review. Chest, 1999; 116(4):961-967.
83.	Mhlengi Vella Ncube, Tivani Mashamba-Thompson. Evidence on implementation of HIV/AIDS, STIs and TB related point of care diagnostics in low and medium income countries.
84.	Marie Varughese, Michael Li, Courtney Heffernan. Time to diagnosis and treatment of pulmonary tuberculosis in indigenous peoples: a systematic review.
85.	Md Asiful Islam, Mahfuza Marzan, Shoumik Kundu. Prevalence of antibiotic-resistant pulmonary tuberculosis in Bangladesh: A systematic review and meta-analysis.
86.	Balewgizie Sileshi Tegegne, Tesfa Dejenie Habtewold, Melkamu Merid Mengesha, Hans Burgerhof. Diabetes mellitus and multi-drug resistant tuberculosis: a protocol for a systematic review and meta-analysis.
87.	Benjamin Momo Kadia, Fongwen Noah Takah, Christian Akem Dimala, Victoria Simms. A systematic review and meta-analysis of the association between integrated tuberculosis and human immuno-deficiency virus therapy and tuberculosis treatment outcomes in sub-Saharan Africa.
**Wrong study design**	
88.	Ruslami, Rovina and Aarnoutse, Rob E and Alisjahbana, Bachti and van der Ven, Andre J A M and van Crevel, Reinout. Implications of the global increase of diabetes for tuberculosis control and patient care. Tropical medicine & international health : TM & IH, 2010; 15(11):1289-99.
89.	Sarkar M. and Srinivasa and Madabhavi I. and Kumar K. Tuberculosis associated chronic obstructive pulmonary disease. Clinical Respiratory Journal, 2017; 11(3):285-295.

90. Pablo-Villamor M.P. and Benedicto J.P. and Benedicto M.T.J.U. and Perez V.M. Screening for diabetes mellitus in patients diagnosed with pulmonary tuberculosis. Phillippine Journal of Internal Medicine, 2014; 52(4):44440.

91. Sharma P. and Visnegarwala F. and Tripathi V. Burgeoning double burden of tuberculosis and diabetes in India: Magnitude of the problem - Strategies and solutions. Clinical Epidemiology and Global Health, 2014; 2(3):107-116.

92. Wenjie Fang, Min Chen, Jia Liu, Weihua Pan, Wanqing Liao. Tuberculosis/Cryptococcosis co-infection in China from 1965 to 2016: a probably underestimated challenge.

93. Kouemo Motse, Dorgelesse F and Nsagha, Dickson Shey and Adiogo, Dieudonne and Kojom Foko, Loick P and Teyim, Pride M and Chichom-Mefire, Alain and Nguedia Assob, Jules C. Tuberculosis Chemotherapy Outcome in the Littoral Region of Cameroon: A Meta-analysis of Treatment Success Rate between 2014 and 2016. BioMed research international, 2020; 2020(101600173):8298291.

94. Cheng, Jun and Zhang, Hui and Zhao, Yan Lin and Wang, Li Xia and Chen, Ming Ting. Mutual Impact of Diabetes Mellitus and Tuberculosis in China. Biomedical and environmental sciences : BES, 2017; 30(5):384-389.

95. Balinda I.G. and Sugrue D.D. and Ivers L.C. More Than Malnutrition: A Review of the Relationship between Food Insecurity and Tuberculosis. Open Forum Infectious Diseases, 2019; 6(4):.

96. Guto J.A. and Bii C.C. and Denning D.W. Estimated burden of fungal infections in Kenya. Journal of Infection in Developing Countries, 2016; 10(8):777-784.

97. Doherty A.M. and Kelly J. and McDonald C. and O'Dywer A.M. and Keane J. and Cooney J. A review of the interplay between tuberculosis and mental health. General Hospital Psychiatry, 2013; 35(4):398-406.

98. Creswell J. and Raviglione M. and Ottmani S. and Migliori G.B. and Uplekar M. and Blanc L. and Sotgiu G. and Lonnroth K. Series: "Update on tuberculosis" - Tuberculosis and noncommunicable diseases: Neglected links and missed opportunities. European Respiratory Journal, 2011; 37(5):1269-1282.

99. Agnihotram R.V. Reviewing disease burden among rural Indian women. Online Journal of Health and Allied Sciences, 2004; 3(2):.

100. Baziz A. Miliary tuberculosis associated with adult respiratory distress syndrome. Annales de Medecine Interne, 1995; 146(2):114-122.

101. CedeÃ±o-Burbano, Anuar Alonso and CerÃ³n-Ortega, Ronal Fredy and Pacichana-Agudelo, Carlos Eberth and MuÃ±oz-GarcÃa, David AndrÃ©s and Galeano-TriviÃ±o, Gerardo Alfonso and Cardona-GÃ³mez, Diana Catalina and Manquillo-Arias, William AndrÃ©s and Plaza-Rivera, Regina Victoria. Parasitismo intestinal y tuberculosis. Rev. Fac. Med. (BogotÃ¡), 2017; 65(4):673-677.

No data from LMICs

102. Lonnroth, Knut and Williams, Brian G and Cegielski, Peter and Dye, Christopher. A consistent log-linear relationship between tuberculosis incidence and body mass index. International journal of epidemiology, 2010; 39(1):149-55.

Duplicated reference

103. Noubiap, Jean Jacques and Nansseu, Jobert Richie and Nyaga, Ulrich Flore and Nkeck, Jan Rene and Endomba, Francky Teddy and Kaze, Arnaud D and Agbor, Valirie N and Bigna, Jean Joel. Global prevalence of diabetes in active tuberculosis: a systematic review and meta-analysis of data from 2.3 million patients with tuberculosis. The Lancet. Global health, 2019; 7(4):e448-e460.

104. Zheng, Yixiang and Ma, Shujuan and Tan, Deming and Lu, Menghou. [A meta-analysis of liver lesions in hepatitis B patients undergoing anti-tuberculosis therapy]. Zhonghua gan zang bing za zhi = Zhonghua ganzangbing zazhi = Chinese journal of hepatology, 2014; 22(8):585-9.

105. Liu, Jiao and Lu, Bing and Yan, Yan. [Meta analysis on the co-infection between Mycobacterium tuberculosis and HIV/AIDS in China]. Zhonghua liu xing bing xue za zhi = Zhonghua liuxingbingxue zazhi, 2013; 34(1):85-90.

106. Pang P. and Sun C. and Duan W. and Liu S. and Bai S. and Ma Y. and Li R. and Liu F. A review on global head and neck tuberculosis cases from 980 papers and 5881 patients. International Journal of Oral and Maxillofacial Surgery, 2019; 48():185.

107. Liu J. and Lu B. and Yan Y. [Meta analysis on the co-infection between Mycobacterium tuberculosis and HIV/AIDS in China]. Zhonghua liu xing bing xue za zhi = Zhonghua liuxingbingxue zazhi, 2013; 34(1):85-90.

108. Carlos Almeida, Rachel Couban, Sun Kallyth, Li Wang, Jason Busse, Denise Silva. Predictors of in-hospital mortality among patients with pulmonary tuberculosis: systematic review and meta-analysis of observational studies.

109. Bisson G.P. and Bastos M. and Campbell J.R. and Bang D. and Brust J.C. and Isaakadis P. and Lange C. and Menzies D. and Migliori G.B. and Pape J.W. and Palmero D. and Baghei P. and Tabarsi P. and Viiklepp P. and Vilbrun S. and Walsh J. and Marks S.M. Mortality in adults with multidrug-resistant tuberculosis and HIV by antiretroviral therapy and tuberculosis drug use: an individual patient data meta-analysis. The Lancet, 2020; 396(10248):402-411.

110. Hong-Guang CHEN and Min LIU and Fang-Hui GU. Meta-analysis on the co-morbidity rate between tuberculosis and diabetes mellitus in China. Chinese Journal of Epidemiology, 2013; (12):1128-1133.

Protocol of an already included study

111. Tankeu, Aurel T and Bigna, Jean Joel and Nansseu, Jobert Richie and Endomba, Francky Teddy A and Wafeu, Guy Sadeu and Kaze, Arnaud D and Noubiap, Jean Jacques. Global prevalence of diabetes mellitus in patients with tuberculosis: a systematic review and meta-analysis protocol. BMJ open, 2017; 7(6):e015170.

112. Marriott Nliwasa, Elizabeth Corbett, Peter MacPherson, Ankur-Gupta Wright, Katherine Horton. The Prevalence of HIV and Risk of Early Mortality in Adults with Suspected Tuberculosis in Low- and Middle- Income Countries: A Systematic Literature Review.

113. Tamuzi Lukenze Jacques, Birhanu Bayele. Covid-19 implications in high burden HIV/TB countries: a systematic review of evidence.

114. sanju gautam. Prevalence of diabetes mellitus among tuberculosis patients and itâ€™s impact on treatment outcome in South Asian Region: a systematic review and meta-analysis.

115. Cesar Ugarte-Gil, Fiona Pearson. Systematic review and meta-analysis on the diabetes mellitus role on tuberculosis treatment outcomes.

116. Aurel. T. Tankeu, Jean JoÃ«l R. Bigna, Jobert Richie N. Nansseu, Francky Teddy A. Endomba, Guy Sadeu Wafeu, Arnaud D. Kaze, Jean Jacques N. Noubiap. Global prevalence of diabetes mellitus in patients with tuberculosis: a systematic review and meta-analysis protocol.

117. Chi Yan Leung, Hsi Lan Huang, Md. Mizanur Rahman, Sarah Krull Abe, Stuart Gilmour, Kenji Shibuya. Pulmonary and extra-pulmonary tuberculosis infection and the risk of pulmonary and extra-pulmonary malignancies: a systematic review and meta-analysis.

118.	Animut Alebel, Amsalu Taye Wendemagen, Cheru Tesema, Getiye Dejenu, Fasil Wagnew, Fasil Wagnew, Pammla Petrucka, Setegn Eshetie. Prevalence of diabetes mellitus among tuberculosis patients in Sub-Saharan Africa, and the impact of HIV infection: a systematic review and meta-analysis.
119.	Hasan Abolghasem Gorji, Nicola Luigi Bragazzi. A systematic review and meta-analysis of the prevalence of Hepatitis C virus (HCV) among tuberculosis patients.
120.	Aklilu Endalamaw, Setegn Eshetie, Sintayehu Ambachew, Demeke Geremew, Tesfa Dejenie Habtewold. HIV-infection and unknown HIV status of tuberculosis patients in Ethiopia: a systematic review and meta-analysis.
121.	C. Andrew Basham, Sarah Smith, James C. Johnston. Tuberculosis (TB) and cardiovascular disease (CVD) risk: a systematic review and meta-analysis.
122.	Abraham Assefa, Awoke Derbie, Abebe Shumet, Abaineh Munshae, Endalkachew Nibret, Fantahun Biadglegne. Epidemiology of Tuberculosis Lymphadenitis in Africa: a systematic reviews and meta-analysis.
123.	Habteyes Tola, Kourosh Holakouie-Naieni, Mohammad Mansournia, Mehdi Yaseri, Ephrem Tesfaye, Million Mola, Tadesse Lejisa Lejisa. Is hypothyroidism rare and does it require screening in multidrug-resistant tuberculosis patients on treatment? A systematic review and meta-analysis.
124.	Alvaro Schwalb, CÃ©sar Ugarte, Rodrigo Cachay, Paulo Ruiz, Adriana De la Flor. The association between tuberculosis and depression on poor outcomes of tuberculosis treatment: a systematic review.
125.	Dumessa Edessa, Moti Tolera, Bisrat Hagos. Poor outcomes to second-line tuberculosis treatment among multidrug-resistant patients in sub-Saharan Africa: a systematic review and meta-analysis.
**Full text could not be found**	
126.	Wongtrakul, Wasit and Charoenngam, Nipith and Ungprasert, Patompong. Tuberculosis and risk of coronary heart disease: A systematic review and meta-analysis. The Indian journal of tuberculosis, 2020; 67(2):182-188.
127.	Pang P. and Sun C. and Duan W. and Liu S. and Bai S. and Ma Y. and Li R. and Liu F. A review on global head and neck tuberculosis cases from 980 papers and 5881 patients. International Journal of Oral and Maxillofacial Surgery, 2019; 48():182.
128.	Yixiang ZHENG and Shujuan MA and Deming TAN and Menghou LU. A meta-analysis of liver lesions in hepatitis B patients undergoing anti-tuberculosis therapy. Chinese Journal of Hepatology, 2014; (12):585-589.
**Protocol**	
129.	Benjamin Momo Kadia, Desmond Aroke, Kevin Pene Njefi, Yves-Joel Tochie Noutakdie, Frank-Leonel Tianyi, Reine Suzanne Kadia, Christian Akem Dimala. Systematic review of therapeutic outcomes of multi-drug resistant tuberculosis and their predictors in adults receiving integrated treatment of tuberculosis and Human Immuno-deficiency Virus in low and middle-income countries: a study protocol.
130.	Noemia Siqueira-Filha. Costs of tuberculosis and HIV co-infection for the health system and families worldwide: a systematic literature review.
131.	Minmin Li. National, regional, and global prevalence of diabetes mellitus in patients with tuberculosis: a systematic review and meta-analysis.
132.	Yonggang Zhang, Linli Zheng, Lu Huang. The prevalence of depression in tuberculosis patients: a meta-analysis.
133.	Tamaryn J. Nicholson, Muhammad Osman, Rory Dunbar, Anneke. C. Hesseling, Florian Marx, Elisa Lopez Varela, James A. Seddon, Kogie Naidoo. A systematic review of risk factors for mortality among drug-susceptible and drug-resistant TB patients in South Africa (2010 â€“ 2018).
134.	Rebecca Harris, Fidel Vazquez. A systematic review and meta-analysis of the effect of HIV status on the incidence of tuberculosis disease among individuals with latent Mycobacterium tuberculosis infection.
135.	Nicolas Nunez, Aiswarya Nandakumar, Lakshmi, Aparna Menon, Boney Joseph. Risk of depression in pulmonary tuberculosis.
136.	Melese Teferi, Mekonnen Mekonnen, Hawult Adane. Determinants of tuberculosis (TB) treatment outcome in resource-limited settings: protocol for systematic review and meta-analysis.
137.	Liu Chun, yang Xie, Hulei Zhao, Jiansheng Li. Prevalence of pneumoconiosis and that complicated with pulmonary tuberculosis in China: a systemic review and meta-analysis.
138.	Melaku Kindie Yenit, Wubet Worku Takele, Achenef Asmamaw Muche, Setegn Eshetie Kebede, Dessie Abebaw Angaw. The effect of depression on Tuberculosis (TB) treatment outcome in Africa: A Protocol for systematic review and meta-analysis.
139.	Lucy Kaluvu, Asogwa Ogechukwu, Anna Marza Florensa, Daniel Boateng, Kerstin Klipstein-Grobusch. A systematic review to examine the patterns, prevalence and management of multimorbidity of communicable and non-communicable diseases in low- and middle- income countries.
140.	Marie Charmaine Sy, Adrian Espiritu, Jose Leonard Pascual. Global frequency of stroke in tuberculous meningitis: A systematic review and meta-analysis.
141.	Mogesie Necho, Mekonnen Tsehay, Asmare Belete. Prevalence and Associated Factors of Alcohol Use Disorder Among Tuberculosis Patients: A Systematic Review and Meta-analysis.
142.	Mogesie Necho, Mengesha Birkie. Psychological distress and Depression in Tuberculosis patients in Africa: A systematic Review and Meta-analysis.
143.	Javier Ogembo, Rebecca Ogembo, Paul Bain. Prevalence of drug-resistant tuberculosis in sub-Saharan Africa: systematic review and meta-analysis.
144.	Adhanom Baraki, Abel Dadi, Hanna Desyibelew. Epidemiology of depression among tuberculosis patients: a systematic review and meta-analysis of observational studies from Africa.
145.	Jing Wu, Jennifer M McGoogan, Zunyou Wu. Prevalence of HIV/TB co-infection worldwide: a systematic review and meta-analysis.
146.	Birhanie Mekuriaw, Alemayehu Molla, Zelalem Belayneh, Tsegaye Mehare. Depression and its determinants among individuals with Tuberculosis in Ethiopia: a systematic review and meta-analysis.
147.	Chalachew Adugna, Aklilu Enalamaw. Poor treatment adherence and associated factors among patients taking anti-tuberculosis drug in Ethiopia: systematic review and meta-analysis.
148.	Gebremedhin Berhe Gebregergs, Gebreamlak Gidey, Fitwi Tinsae, Gebremedhin Gebreziher, Selam Desalegn. Gender difference in co-morbid depression among tuberculosis patients in high burden countries: systematic review and meta-analysis.
149.	Belayneh Kefale, Mulugeta Molla, Amien Ewunetie, Amsalu Degu, Gobezie Temesgen. Treatment outcomes and associated factors among tuberculosis patients in Ethiopia: a systematic review and meta analysis.

150. Alvaro Schwalb, Jorge InolopÃº, Eduardo Gotuzzo, Rodrigo Cachay, Fernando MejÃa, Larissa Otero, Carlos Seas, CÃ©sar Ugarte-Gil, Kristien Verdonck, Nicole Young. Clinical and epidemiological association between HTLV-1/2 and tuberculosis: systematic review.
151. Demeke Geremew. Latent TB and its associated factors in Ethiopian: an aggregated and individual patientsâ€™ data meta-analysis.
152. Carole Mitnick, Molly Franke, Celia Fung, Andrew Lindeborg. Clinical Outcomes of Individuals with COVID-19 and Tuberculosis Disease: a Living Systematic Review.
153. Javier Cabrera, Vicente Cuba. Tuberculosis and lung cancer risk: a systematic review and meta-analysis.
154. Jemal Abdela, Fuad Adem, Abraham Nigussie. Incidences and risks of drug-induced hepatotoxicity among Tuberculosis and HIV/ Tuberculosis co-infected patients in Sub Saharan Africa: a systematic review and meta-analysis.

Conference abstract

155. Schafer J.M. and Welwarth J. and Balk D.S. and Lee C. and Hardin J. and Hoffmann B. Rates of pericardial effusion in patients with HIV/tuberclosis co-infection: A systematic review and meta-analysis. Academic Emergency Medicine, 2017; 24():S97.
156. Moniruzzaman A. and Kazanjian A. and Wong H. and Chowdhury M.M. and Elwood R.K. and Fitzgerald J.M. A systematic review on risk factors of mortality among Tb patients. American Journal of Respiratory and Critical Care Medicine, 2010; 181(1):.
157. Yeats, J. and Patel, S. and Shete, P. B. and Cattamanchi, A. and Baker, B. J. Reactivation Risk Of Latent Tuberculosis Infection By Population Subgroup: A Systematic Review And Meta-Analysis. AMERICAN JOURNAL OF RESPIRATORY AND CRITICAL CARE MEDICINE, 2016; 193():.
158. Huang Y.-S. Chronic hepatitis C may increase the risk of anti-tuberculosis drug-induced liver injury: A systematic review and meta-analysis. Hepatology International, 2017; 11(1):S842.
159. Chen H. and Liu M. and Gu F. Meta-analysis on the co-morbidity rate between tuberculosis and diabetes mellitus in China. Zhonghua liu xing bing xue za zhi = Zhonghua liuxingbingxue zazhi, 2013; 34(11):1128-1133.
160. Arruda S. and Loureiro C. and Almeida M. and Mendes D. and Grassi M.F.R. and Lapa J.R. and Kritski A. and Verdonck K. and Gotuzzo E. and Galvao-Castro B. Association between human T-Cell lymphotropic virus type 1 and 2 (HTLV 1/2) infection and tuberculosis: Systematic review and meta-analysis. Retrovirology, 2011; 8():A80.
161. Hussain, M. S. and Siddiqui, A. N. and Najmi, A. K. Burden of diabetes mellitus among tuberculosis patients in Asia-Pacific region: Evidence from meta-analysis using real-world data. INTERNATIONAL JOURNAL OF INFECTIOUS DISEASES, 2018; 73():87.
162. Jiao LIU and Bing LV and Yan YAN. Meta analysis on the co-infection between Mycobacterium tuberculosis and HIV/AIDS in China. Chinese Journal of Epidemiology, 2013; (12):85-90.

Wrong publication type (letters to the editor, corrections, protocol for this meta-review)

163. Pai, Madhukar and McCulloch, Michael and Colford, John M Jr. Meta-analysis of the impact of HIV on the infectiousness of tuberculosis: methodological concerns. Clinical infectious diseases : an official publication of the Infectious Diseases Society of America, 2002; 34(9):1285-7.
164. Chen M. and Al-Hatmi A.M. and Chen Y. and Ying Y. and Fang W. and Xu J. and Hagen F. and Hong N. and Boekhout T. and Liao W. and Pan W. Cryptococcosis and tuberculosis co-infection in mainland China. Emerging Microbes and Infections, 2016; 5(9):e98.
165. Anonymous. Correction: Association between HIV/AIDS and multi-drug resistance tuberculosis: A systematic review and meta-analysis (PLoS ONE). PLoS ONE, 2014; 9(2):e89709.
166. Chen L. and Li N. and Liu M. and Zhang J. and Zhang H. High prevalence of multidrug-resistant tuberculosis in Zunyi, Guizhou province of China. Journal of Antimicrobial Chemotherapy, 2011; 66(10):2435-2437.
167. Alexander Jarde, Ruimin Ma, Eugenia Romano, Helen Elsey, Kamran Siddiqi, Najma Siddiqi, Brendon Stubbs. A meta-review of systematic reviews of chronic disease multimorbidity in people with tuberculosis in low- and middle-income countries.

Supplementary Table 3: Study characteristics of systematic reviews reporting pooled outcomes of studies from LMICs

Lead author and year	Search dates and limits	Number of studies in LMIC/Total number of studies (countries covered)	Clusters covered	Outcomes	Quality rating
**TB + HIV**					
Alemu 2020[S54]	2009 to Jan 2020; English language, in Ethiopia	17/17 (Ethiopia)	TB +HIV	Prevalence*	Low
Arega 2020 [S24]	200 to 2019; English language, in Ethiopia	47 / 47 (Ethiopia)	TB +HIV	Prevalence*	Critically low
Bastos 2019 [S25]	period between 2008 and 2017; Brazil only	15 /15 (Brazil)	TB +HIV	Mortality*, Treatment outcomes*	Critically low
Bisson 2020 [S48]	2009 to Sep 2015	52 (South Africa, Philippines, Georgia, Russia, Latvia, Peru, India, Haiti, Pakistan, Belarus, Brazil, Thailand, Mexico, Bulgaria, Argentina, Ecuador)	MDR-TB +HIV	Prevalence (pooled individuals - no meta-analysis)	Low
Chem 2019 [S5]	2004 to May 2018; English language, in SSA countries	9 / 9 (South Africa, Lesotho, Botswana, Ethiopia)	MDR-TB +HIV	OR of treatment success and prevalence of death and cured. Prevalence*	Critically low
Edessa 2020 [S6]	up to Feb 2020; English language, in SSA countries	19/19 (South Africa, Lesotho, Ethiopia, Kenya, Tanzania, Niger, Botswana)	DR-TB +HIV	RR of unfavourable outcome (Failed treatment/Lost from treatment/died), death, treatment failure and loss to follow-up	High
Endalamaw 2019 [S7]	2003-2018; English language, in Ethiopia	13/13 (Ethiopia)	TB +HIV ; PTB +HIV	Prevalence	Moderate
Eshetie 2018 [S2]	up to 2017; English language, in Ethiopia	34 /34 (Ethiopia)	TB +HIV	Prevalence of successful and unsuccessful treatment	Critically low
Gao 2010 [S8]	up to Apr 2010; English or Chinese language, in mainland China	29/29 (China)	TB +HIV	Prevalence (also by sex)	Critically low
Gao 2013 [S9]	up to Dec 2011; English language, any country except China	31/47 (Brazil, Nigeria, Ethiopia, India, Iran, South Africa, Zambia, Zimbabwe, Cambodia, Tanzania, Thailand, Togo, Ukraine, Vietnam)	TB +HIV	Prevalence	Critically low
Gelaw 2019 [S10]	up to Sep 2017; English language in SSA countries	68/68 (Ethiopia, Tanzania, Kenya, Eritrea, Uganda, South Africa, Zambia, Zimbabwe, Angola, Malawi, Nigeria, Cote d'Ivoire, Ghana, Burkina Faso, Togo, Cameroon, Republic of Congo)	TB +HIV	Prevalence (in SSA and in the central, southern, western and eastern regions of SSA)	High
Huddart 2020 [S1]	2006 to Jan 2019; in India	212 / 212 (India)	TB +HIV	Case-fatality rate (during treatment and after treatment)	Critically low
Lukoye 2015 [S26]	2003 to 2013; SSA countries only	27/27 (SSA)	TB +HIV	Prevalence*	Critically low
McMurry 2019 [S27]	1990 to 2016.; English language, LMIC only	84/84 (India, China, Mexico, Tanzania, Ethiopia, Malaysia, Pakistan, Brazil, Nigeria, South Africa, Bangladesh, Indonesia, Marshall Islands, Turkey, Benin, Fiji, Georgia, Guinea, Guinea-Bissau, Guyana, Iran, Kazakhstan, Kiribati, Kyrgystan, Micronesia, Peru, Philippines, Sri Lanka, Thailand, Zambia)	TB +HIV ; TB +DM	Prevalence*	Low
Mekonnen 2019 [S11]	up to Mar 2018; English language, in African countries	28 / 28 (Ethiopia, Zambia, South Africa, Nigeria, Burkina Faso, Uganda, Djibouti, Mozambique, Sudan, Tunisia, Tanzania, Malawi)	TB lymphadenitis +HIV	Prevalence	High
Mesfin 2014 [S49]	up to April 2012.; English language	4/24 (Ucraine, Haiti, Georgia, South Africa)	MDR-TB +HIV	Prevalence*, OR*	Critically low
Pormohammad 2018 [S52]	1985 to Mar 2018	16 / 20 (Dominican Republic, South Africa, Indonesia, China, Vietnam, Turkey, India, Brazil, Peru, Zambia)	TB Meningitis +HIV	Prevalence*	Critically low

Pourakbari 2019 [S28]	up to Apr 2017; Persian and English languages, Iran only	48/48 (Iran)	TB +HIV	Prevalence*	Critically low
Purmohamad 2020 [S53]	2000 to Jan 2017; English language	22/26 (South Africa, Turkey, China, India, Egypt, Brazil, Peru, Vietnam, Indonesia)	TB Meningitis +HIV	Prevalence*	Critically low
Rajendran 2020 [S29]	2009 to Dec 2018; English language, Malaysia only	23/23 (Malaysia)	TB +HIV; TB +DM	Prevalence*	Critically low
Reddy 2010 [S30]	up to Jun 2009; African countries only	22 / 22 but only 5 provided data on TB (Tanzania, Malawi, Uganda, and Cote d'Ivoire)	TB +HIV	Prevalence (pooled individuals - no meta-analysis)	Critically low
Samuels 2018 [S12]	1980 to Jun 2016; English, French and Spanish language	39 / 48 (Ethiopia, Georgia, Russia, Latvia, South Africa, India, Cameroon, Nigeria, Pakistan, Vietnam, Uzbekistan, Moldovia, Belarus, Peru, China, Turkey, Haiti, Philippines,)	MDR/XDR-TB +HIV; MDR/XDR-TB +DM	RR of unsuccessful treatment (composite of failure, death, and default) and treatment failure. Prevalence*	Low
Seid 2018 [S31]	up to Mar 2017; English language, Ethiopia only	34 / 34 (Ethiopia)	TB +HIV	Prevalence*	Critically low
Sotgiu 2009 [S47]	2006 to Dec 2008.; English language	5 / 13 (South Africa, Russia, Peru)	MDR-TB +HIV; XDR-TB +HIV	Mortality*, Treatment outcomes*	Critically low
Straetemans 2011 [S32]	up to Mar 2011; English language	50 / 70 (studies with TB + HIV: 14 / 22) (South Africa, Ivory Coast, Uganda, Somalia, Iran, Malawi, Thailand, Zambia, Mexico, Russia, Sudan, Cambodia, Central African Republic, Guinea-Bissau, Nepal, Ivory Coast, India, Burkina Faso, Cameroon, Kenya, Zaire, China, Vietnam)	TB +HIV	Mortality*	Critically low
Tesfaye 2018 [S33]	2007 to 2016; English language, Ethiopia only	21/21 (15 for prevalence data) (Ethiopia)	TB +HIV	Prevalence*	Critically low
Teweldemedhin 2018 [S34]	1995 to Nov 2017; English language, Ethiopia only	30/30, but only 19/30 determined HIV infection among TB patients (Ethiopia)	TB +HIV	Prevalence*	Critically low
Uchida 2019 [S35]	1919 to 2017; English language	2/7 (India, Nigeria)	TB +HIV	Treatment outcomes*	Critically low
Waitt 2011 [S36]	1966 to 2010; English language	40 / 62 (China, Sudan, Brazil, Vietnam, South Africa, Gambia, Malawi, India, Russia, Thailand, Tanzania, Guinea Bissau, Peru, Mexico, Zambia, Uganda, Bolivia)	TB +HIV; TB +Non-infective comorbidities	Mortality*	Critically low
Wang 2019 [S13]	up to May 2018; English language	21 / 22 (Turkey, India, China, Vietnam, South Africa, Indonesia, Malaysia, Madagascar)	TB meningitis +HIV	Prevalence and prevalence of death	Critically low
Wu 2016 [S50]	up to Oct 2012; English or Chinese language	23/39 (Peru, India, Bangladesh, Lesotho, Iran, China, Latvia, Russia, Uzbekistan, Turkey, South Africa)	MDR-TB +HIV	Prevalence*	Critically low
**TB + DM**					
Alebel 2019 [S14]	up to Sep 2017; English language, in SSA countries	16/16 (Benin, Tanzania, Guinea-Bissau, Uganda, Nigeria, Ethiopia, Guinea, Madagascar, Kenya, Cameroon)	TB +DM	Prevalence	Low
Almeida 2018 [S51]	up to Nov 2015	2/11 (Iran, China)	PTB +DM	Mortality*	Critically low
Baker 2011 [S37]	1980 to Dec 2010	12 / 33 (Indonesia, Thailand, India, Turkey, Iran, Russia, Tunisia, Republic of the Congo, Mexico, China)	TB +DM	Prevalence*, Mortality*, Treatment outcomes*	Critically low
Chen 2013 [S15]	2000-Apr 2013; in China	22/22 (China)	PTB +DM	Prevalence	Critically low
Gautam 2021 [S4]	1980 to Jul 2020; English language, in Afghanistan, Bangladesh, Bhutan, India, Maldives, Nepal, Pakistan, Sri Lanka	65/ 74 (India, Pakistan, Nepal, Bangladesh, and Sri-Lanka)	TB +DM	Prevalence (also by countries), OR of mortality, treatment failure, culture conversion, recurrence, and MDR-TB	Low

Han 2016 [S38]	1980 to Jul 2015; English language	27/54 (total articles included), 27/33 (articles used for the meta-analysis) (Congo, Tunisia, Russia, Turkey, Thailand, China, India, Mexico, Iran, Kiribati, Brazil, Malaysia, Tanzania, Peru, Indonesia)	TB +DM	Mortality*, Treatment outcomes*	Critically low
Huang 2020 [S39]	1966 to Jul 2019; English only	10/13 (India, China, Mexico, Thailand, Georgia, Iran)	TB +DM	Prevalence*	Critically low
Huangfu 2019 [S16]	1980 to Jul 2018	57/104 (Congo, Indonesia, Iran, Thailand, Tunisia, Turkey, Brazil, India, Mexico, China, Malaysia, Fiji, Kiribati, Ethiopia, Argentina, Poland, Tanzania, Russia, Egypt, Uganda, Georgia, Saudi Arabia, Senegal)	TB +DM	OR of death only and treatment failure and death	Low
Jeon 2010 [S40]	up to May 2009 (databases), 2007 to 2008 (World Lung Conferences abstracts)	16 / 18 studies that met our inclusion criteria on screening for DM among patients with TB (32 studies included in total) (India, Russia, Nigeria, Guinea, Pakistan, Turkey, Indonesia, Tanzania, Mexico, Iran)	TB +DM	Prevalence*, RR*	Critically low
Lutfiana 2019 [S41]	2012 to Sep 2017; English language	32/41 (South Africa, China, India, Thailand, Bangladesh, Georgia, Brazil, Tanzania, Vietnam, Mongolia)	TB +DM	Prevalence*	Critically low
Noubiap 2019 [S17]	1986 to Jun 2017	138/ 200 (Benin, Ethiopia, Guinea-Bissau, Nigeria, Senegal, South Africa, Tanzania, Uganda, Georgia, Kazakhstan, Romania, Turkey, Egypt, Iran, Libya, Pakistan, Tunisia, Yemen, Guyana, Mexico, Brazil, Peru, Bangladesh, India, Sri Lanka, China, Fiji, Indonesia, Kiribati, Malaysia, Marshall Islands, Thailand)	TB +DM	Prevalence (by income level, regions and countries)	Critically low
Shao-hua 2016 [S18]	up to Nov 2015; in China	13 / 13 (China)	PTB +DM	OR and aOR of adverse outcomes (failure to retreatment, death, and loss)	Critically low
Tegegne 2018 [S42]	up to Jul 2018; English language	17 / 25 (Iran, Georgia, Mexico, Egypt, Thailand, Peru, China, Indonesia, Bangladesh, Turkey)	TB +DM	Prevalence*	High
Workneh 2017 [S43]	up to Mar 2016; English language	80/94 (India, China, Iran, Indonesia, Pakistan, Sri Lanka, Nepal, Thailand, Georgia, Philippines, Malaysia, Benin, Ethiopia, Tanzania, Guinea, Kenya, Ethiopia, Uganda, Nigeria, South Africa, Madagascar, Mexico, Peru, Brazil, Kiribati, Marshall Islands, Guyana, Fiji)	TB +DM	Prevalence*	Low
**TB + Mental disorders**					
Alene 2018 [S19]	up to September 2017	37/40 (1 studies on Canada and 2 on South Korea) (China, Peru, Russia, Argentina, Turkey, Iran, Lesotho, Latvia, South Africa, Tanzania, Haiti, Pakistan, Ethiopia, Vietnam, Indonesia, Nigeria, Namibia)	MDR-TB +Depression; MDR-TB +Anxiety; MDR-TB +Psychosis	Prevalence (also by regions)	Critically low
Duko 2020 [S20]	up to Dec 2019; English language	25/25 (Pakistan, Turkey, India, Brazil, China, Nigeria, Cameroon, Ethiopia)	TB +Depression; MDR-TB +Depression	Prevalence (also by sex)	High
Lee 2020 [S21]	1990 to Oct 2018; English, French, Spanish, Portuguese, and Korean languages	10/10 (South Africa, Ethiopia, Zimbabwe, Zambia, Tanzania, Peru, China)	TB +Mental Disorders	OR of poor TB treatment outcomes, loss to follow-up and non-adherence to treatment. Prevalence*, Mortality*	Critically low
Rensburg 2020 [S44]	2000 to 2019; English language	100/100 (Pakistan, South Africa, Peru, Pakistan, China, Ethiopia, Thailand, India, Sudan, Cameroon, Kazakhstan, Sri Lanka, Nigeria, Zambia, Russia, Brazil, Poland, Burkina Faso, Estonia, Angola, Romania, Ukraine)	TB +Mental illness (Depression, Anxiety, Alcohol use, and General Mental health)	Prevalence*	Critically low

Ruiz-Grosso 2020 [S3]	up to Aug 2019; English language	8/8 (South Africa, Peru, Ethiopia, China, Zimbabwe, Zambia, Tanzania)	TB +Depression	OR of negative outcomes (death and loss to follow-up), death, loss to follow-up and non-adherence	Critically low
**TB + HCV**					
Behzadifar 2019 [S22]	2000 to Mar 2018; English language	13/21 (Georgia, Argentina, Iran, Brazil, Egypt, Pakistan, China, Sudan, Iraq)	TB +HCV	Prevalence	Moderate
**TB + other**					
Basham 2020 [S45]	up to Jan 2020 (databases), 2013 to Dec 2019 (The International Journal of Tuberculosis and Lung Disease); English language	5/16 (Tanzania, Egypt, Peru, Russia, Estonia)	TB + cardiovascular disease	OR*, Mortality*	Critically low
Leung 2020 [S23]	up to Jun 2019; any language	14/47 (Lithuania, China, Czech Republic)	TB +Lung cancer; TB +non-Hodgkin's lymphoma; TB +Leukaemia	RR of lung cancer, non-Hodgkin's lymphoma, and leukaemia	Low
Rehm 2009 [S46]	up to Sep 2008; English language	14/53 (Russia, India , Brazil, Belarus, Kazakhstan, Romania, Slovenia)	TB+AUD (Alcohol Use Disorder)	Prevalence*	Critically low

Notes: aOR: adjsuted OR; DM: Diabetes Mellitus; DR-TB: Drug resistant TB; HCV: Hepatitis C Virus; HIV: Human immunodeficiency virus; LMIC: Low- and middle-income countries; MDR-TB: Multidrug resistant TB; OR: Odds Ratio; PTB: Pulmonary TB; RR: Relative Risk; SSA: Sub-Saharan Africa; Tuberculosis; XDR-TB: Extensively drug-resistant TB.

* Outcomes available for individual studies, but not pooled

Supplementary Table 4: Outcomes reported by each systematic review

Lead author and year	Clusters covered	Outcomes	Quality rating
**TB+HIV**			
Alemu 2020	TB +HIV	Outcomes available for individual studies, but not pooled: Prevalence*: range 7.1% - 30.4%	Low
Arega 2020	TB +HIV	Outcomes available for individual studies, but not pooled: Prevalence*: range 1.70%-45.80%	Critically low
Bastos 2019	TB +HIV	Outcomes available for individual studies, but not pooled: Mortality*: range 3.6% - 30.9%, Treatment outcomes*: cure (33% - 62%), abandonment of treatment (4.2% - 13.6%)	Critically low
Bisson 2020	MDR-TB +HIV	Low and lower-middle income countries: 5.1% (130 of (2421+130) pooled individuals from two studies) - no MA<br>Upper-middle: 3585/(3585+3244)= 52.5% (pooled individuals from two studies) - no MA	Low
Chem 2019	MDR-TB +HIV	Successful treatment: OR 0.87 (0.79 - 0.96), 6 studies, number of participants NR, I^2 NR, range 0.75 - 1.26)<br>Mortality: 18% (14%-23%, 9 studies, number of participants NR, I^2=91.1%, range=9%-31%)<br>Cured: 34% (22%-45%, 9 studies, number of participants NR, I^2=98.9%, range= 3%-60%)<br>Outcomes available for individual studies, but not pooled: Prevalence*: 21.73% - 100%	Critically low
Edessa 2020	DR-TB +HIV	Unsuccessful treatment: [†] RR 1.18 (1.07-1.30, 19 studies, 8301 participants, I^2=48%, range=0.71-2.37)<br>Unsuccessful treatment (western SSA region) : RR 1.42 (0.95-2.13, 2 studies, 790 participants, I^2=12%, range=1.31-2.37)<br>Unsuccessful treatment (eastern SSA region): RR 1.47 (95% CI: 1.23–1.75, 6 studies, 1970 participants, I^2=0%, range=1.14-1.77)<br>Unsuccessful treatment (southern SSA region): [†] RR 1.09 (0.98-1.20, 11 studies, 5541 participants, I^2=43%, range=0.71-1.41)<br><br>Mortality: [†] RR 1.50 (1.30-1.74, 16 studies, 7365 participants, I^2=39%, range=0.73-2.18)<br>Mortality (western SSA region) : RR 1.42 (0.96-2.09, 1 study, 588 participants)<br>Mortality (eastern SSA region): RR 1.52 (95% CI: 1.19–1.93, 5 studies, 1442 participants, ,I^2=0%, range=1.20-2.18)<br>Mortality (southern SSA region): [†] RR 1.49 (1.21-1.83, 10 studies, 5335 participants, I^2=60%, range=0.73-1.47)<br><br>Treatment failure: [†] RR 0.66 (0.38-1.13, 10 studies, 5474 participants, I^2=73%, range=0.15-2.40)<br>Loss to follow up: [†] RR 0.82 (0.74-0.92, 14 studies, 7051 participants, I^2=0%, range=0.49-2.61)	High
Endalamaw 2019	TB +HIV; PTB +HIV	Prevalence: 23.40% (95% CI 19.56%-27.24%, 13 studies, 19212 participants, I^2=97.6%, range=9.50%-52.10%)<br>PTB. 22.08% (95% CI 14.36%-29.81%, 3 studies, 1079 participants, I^2=89.9%, range 14.97%-28.60%)	Moderate
Eshetie 2018	TB +HIV	Successful treatment: prev 67% (56%-79%, number of studies NR, number of participants NR, I^2 NR, range NR)<br>Unsuccessful treatment: prev 33% (21%-44%, number of studies NR, number of participants NR, I^2 NR, range NR)<br>Unsuccessful treatment: OR (TB+HIV vs TB) 1.98 (1.56-2.52, 20 studies, number of participants NR, I^2 =81.0%, range 0.82-14.31)	Critically low
Gao 2010	TB +HIV	Prevalence: 0.9% (95% CI 0.6%–1.4%, 18 studies, number of participants NR, I^2= 92.21, range 0.1%–4.5%)<br>Men: 1.1% (95% CI 0.6% - 2.0%, 9 studies, number of participants NR, I^2=94.7%)<br>Women: 0.6% (95% CI 0.3% - 1.1%, 9 studies, number of participants NR, I^2=71.8%)	Critically low
Gao 2013	TB +HIV	Africa: 31.2% (95% CI 19.3% - 43.2%), 17 studies, number of participants NR, I^2=99.6%, range NR)<br>Latin America: 25% (95% CI 19.3% - 30.8%), 7 studies, number of participants NR, I^2=95.2%, range NR)	Critically low
Gelaw 2019	TB +HIV	SSA: prevalence 31.81% (95% CI 27.83%-36.07%; 68 studies, 62696 participants, I^2=98%, range=6.03%-72.25%)<br>Eastern region (SSA): prev. 31.14% (95% CI 25.39%-37.54%, 32 studies, 33637 participants, I^2=98%, range=6.03%-60.51%)<br>Western region (SSA): prev. 25.48% (95% CI 19.70%-32.27%), 21 studies, 16145 participants, I^2=98%, range=10.26%-72.13%)<br>Southern region (SSA): prev. 43.67% (95% CI 35.05%-52.69%, 12 studies, 11148 subject, I^2=99%, range=23.84%-72.25%)<br>Central region (SSA): prev. 41.33% (95% CI 30.39%-53.19%, 3 studies, 2039 participants, I^2=96%, range=31.29%-51.56%)	High
Huddart 2020	TB +HIV	Case-fatality rate (during treatment): 10.91% (7.68%-15.50%), 35 studies, number of participants NR, Tau^2=0.90 (considered low heterogeneity if <4, according to the authors)<br>Case-fatality rate (after treatment): 4.15% (1.06% to 16.24%), 5 studies, number of participants NR, Tau2=1.902 (considered low heterogeneity if <4, according to the authors)	Critically low

Jarde A, *et al. BMJ Open* 2022; 12:e060906. doi: 10.1136/bmjopen-2022-060906

Lukoye 2015	TB +HIV	Outcomes available for individual studies, but not pooled: Prevalence*: range 25.4% - 79.9%	Critically low
McMurry 2018	TB +HIV; TB +DM	Outcomes available for individual studies, but not pooled: Prevalence*: range 1.9% - 45%	Low
Mekonnen 2019	TB lymphadenitis +HIV	Africa: 52% (95% CI 33%-71%, 14 studies, number of participants NR, I^2=99.2%, range 6%-91%)<br>Ethiopia: 21% (95% CI 12%-30%, 6 studies, number of participants NR, I^2=92.9%, range 6%-67%)	High
Mesfin 2014	MDR-TB +HIV	Outcomes available for individual studies, but not pooled: Prevalence*: range 3.4% - 31.6%, OR*	Critically low
Pormohammad 2018	TB Meningitis +HIV	Outcomes available for individual studies, but not pooled: Prevalence*: range 0% - 100%	Critically low
Pourakbari 2019	TB +HIV	Outcomes available for individual studies, but not pooled: Prevalence*: range 0.4% - 38%	Critically low
Purmohamad 2020	TB meningitis +HIV	Outcomes available for individual studies, but not pooled: Prevalence*: range 0% - 91%	Critically low
Rajendran 2020	TB +HIV; TB +DM	Outcomes available for individual studies, but not pooled: Prevalence*: MDR-TB+DM: 26.7% (1 study); MDR-TB+HIV: 17.6% (1 study)	Critically low
Reddy 2010	TB +HIV	161 / 166 (97%) – no meta-analysis	Critically low
Samuels 2018	MDR/XDR-TB +HIV ; MDR/XDR-TB +DM	Unsuccessful treatment:<br>MDR/XDR-TB + HIV (low gross domestic product countries): RR 2.23 (1.60-3.11, 7 studies, 2662 participants, I^2=41%, range=0.67-3.33)<br>MDR/XDR-TB + HIV (LMIC): RR 1.34 (1.04-1.72, 13 studies, 5816 participants, I^2=88%, range=0.55-3.33)<br>MDR/XDR-TB + DM (vs MDR/XDR-TB only): RR 0.90 (0.65-1.23, 3 studies, 687 participants, I^2=19%, range=0.23-0.98)<br><br>Treatment Failure (defined as 5 cultures positive within the last 12 months of therapy or any culture positivity within the last 3 cultures; alternatively, failure was defined as treatment discontinuation due to lack of appropriate response or significant adverse events):<br>MDR/XDR-TB + HIV (vs MDR/XDR-TB only): RR 0.75 (0.44-129, 7 studies, 5930 participants, I^2=55%, range=0.32-2.40)<br>Outcomes available for individual studies, but not pooled: Prevalence*:	Low
Seid 2018	TB +HIV	Outcomes available for individual studies, but not pooled: Prevalence*: range 2.0% - 100%	Critically low
Sotgiu 2009	MDR-TB +HIV; XDR-TB +HIV	Outcomes available for individual studies, but not pooled: Mortality*: , Treatment outcomes*:	Critically low
Straetemans 2011	TB +HIV	Outcomes available for individual studies, but not pooled: Mortality*: range 2.2% - 34.4%	Critically low
Tesfaye 2018	TB +HIV	Outcomes available for individual studies, but not pooled: Prevalence*: range 11.4% - 36.2%	Critically low
Teweldemedhin 2018	TB +HIV	Outcomes available for individual studies, but not pooled: Prevalence*: range 6%-52.1%	Critically low
Uchida 2019	TB +HIV	Outcomes available for individual studies, but not pooled: Treatment outcomes*: Unsuccessful outcomes (death, failure, loss to follow-up and transferred-out HIV positive): aOR 3.6 (95%CI 1.1-11.7) (1 study)	Critically low
Waitt 2011	TB +HIV; TB + Non-infective comorbidities	Outcomes available for individual studies, but not pooled: Mortality*:	Critically low
Wang 2019	TB meningitis +HIV	Prevalence: 10.6% (95% CI: 4.2%–24.6%, number of studies NR, number of participants NR, I^2 NR, range NR)<br>Mortality: 53.4% (42.4%–64.1%, 7 studies, 547 participants, I^2= 2.1%, range NR)	Critically low
Wu 2016	MDR-TB +HIV	Outcomes available for individual studies, but not pooled: Prevalence*:	Critically low
**TB+DM**			

Jarde A, *et al. BMJ Open* 2022; 12:e060906. doi: 10.1136/bmjopen-2022-060906

Alebel 2019	TB +DM	Prevalence TB+DM: 9% (95% CI 6%-12%, 16 studies, 13286 participants, I^2= 97.48%, range: 2%-38%)<br>Nigeria: 15% (95% CI 7%-23%, 4 studies, 4998 participants, I^2 NR, range NR)<br>Ethiopia: 10% (95% CI 6%-13%, 3 studies, 1633 participants, I^2 NR, range NR)<br>Tanzania: 11% (95% CI 9%-12%, 2 studies, 1309 participants, I^2 NR, range NR)	Low
Almeida 2018	PTB +DM	Outcomes available for individual studies, but not pooled: Mortality*: OR 9.70 (95% CI 2.92-32.22)	Critically low
Baker 2011	TB +DM	Outcomes available for individual studies, but not pooled: Prevalence*: , Mortality*: range of RR 1.26 - 28.47, Treatment outcomes*: relapse: range of RR 1.88 - 5.96; remaining sputum culture positive: range of RR 0.79 - 2.17, failure/death: range of RR 1.44 - 3.13	Critically low
Chen 2013	PTB +DM	Prev: 7.20% (95% CI 6.01%-8.39%, 22 studies, 56805 participants, I^2 NR, range 2.08%-16.16%)	Critically low
Gautam 2021	TB +DM	Pooled prevalence 21% (95% CI 18%-23%, 65 studies, 49,792 patients, I^2=98.28%, range NR)<br>MDR-TB OR 1.05 (95% CI 0.63-1.74, 4 studies, number of participants NR, I^2=40,71%, range NR)<br>Among adults only: 21.0% (95% CI 18.0–23.0%, 55 studies, number of participants NR, I^2= 97.99%, range NR)<br><br>Bangladesh prevalence 11.0% (95% CI 10.0%–12.0%, 2 studies, number of participants NR, I^2 NR, range NR)<br>India prevalence 22.0% (95% CI 19.0%–25.0%, 47 studies, number of participants NR, I^2=97.92%, range NR)<br>Nepal prevalence 12.0% (95% CI 4.0%–20.0%, 4 studies, number of participants NR, I^2=96.70%, range NR)<br>Pakistan prevalence 19.0% (95% CI 11.0%–27.0%, 10 studies, number of participants NR, I^2=99.18%, range NR)<br>Sri Lanka prevalence 24.0% (95% CI 21.0%–27.0%, 2 studies, number of participants NR, I^2 NR, range NR)<br>Mortality in TB+DM vs TB: OR 1.74 (1.21-2.51, 5 studies, number of participants NR, I^2=19.43%, range 0.14-1.95)<br>Treatment failure: OR 1.65 (1.12-2.44, 5 studies, number of participants NR, I^2=49.63%, range 1.34-21.91)<br>Cured: OR 0.32, 95% CI 0.10 - 1.05, 1 study)<br>Recurrence: OR 0.53 (95% CI 0.32, 0.87, 1 study)<br>MDR-TB: OR 1.05 (0.63-1.74, 4 studies, number of participants NR, I^2=40.71%, range=0.45-4.70)	Low
Han 2016	TB +DM	Outcomes available for individual studies, but not pooled: Mortality*: range of OR 0.41 - 29.22, Treatment outcomes*: Sputum culture conversion at 2 to 3 months: range of OR 0.57 - 5.27; Failure/death: range of OR 0.86 - 18.91; Relapse: range of OR 0.97 - 6.35	Critically low
Huang 2020	TB +DM	Outcomes available for individual studies, but not pooled: Prevalence*: range 9% - 49%	Critically low
Huangfu 2019	TB +DM	Mortality: OR 1.80 (95%CI 1.35–2.40; 32 studies, number of participants NR, I^2=91%, range NR)<br>Treatment failure or death: OR 1.90 (95%CI 1.43–2.53; 22 studies, number of participants NR, I^2=87.3%, range NR)	Low
Jeon 2010	TB +DM	Outcomes available for individual studies, but not pooled: Prevalence*: , RR*	Critically low
Lutfiana 2019	TB +DM	Outcomes available for individual studies, but not pooled: Prevalence*: range 3.3%-100%	Critically low
Noubiap 2019	TB +DM	Prevalence:<br>Low income countries: 7.9% (95% CI 4.9%-11.5%, 15 studies, 9434 participants, I^2=96.8%, range NR)<br>Lower-middle income: 17.7% (95% CI 15.1%-20.5%, 48 studies, 48036 participants, I^2=98.3%, range NR)<br>Upper-middle income: 14.4% (95% CI 12.8%-16.0%, 75 studies, 1,994,027 participants, I^2=99.9%, range NR)<br><br>African region: 8.0% (95% CI 5.9%-10.4%, 119 studies, 474,944 participants, I^2=99.8%, range 1.9%-32.4%)<br>Southeast Asia: 19.0% (95% CI 16.2%-21.9%, 30 studies, 30382 participants, I^2=97.0%, range 5.1%-54.1%)<br><br>Benin: 1.9% (95% CI 0.2%-4.7%, 1 study, 159 participants)<br>Ethiopia: 18.8% (95% CI 1.9%-47.1%, 2 studies, 1749 participants, I^2=99.2%, range: 8.3%-32.4%)<br>Guinea-Bissau: 2.7% (95% CI 0.3%-6.8%, 1 study, 110 participants)<br>Nigeria: 7.8% (95% CI 4.4%-12.0%, 4 studies, 9821 participants, I^2=97.8%, range=4.8%-12.0%)<br>Senegal: 4.9% (95% CI 2.2%-8.5%, 2 studies, 2848 participants, I^2=75.1%, range=3.8%-7.0%)<br>South Africa: 9.4% (95% CI 7.6%-11.3%, 1 study, 947 participants)<br>Tanzania: 8.5% (95% CI 4.8%-13.0%, 7 studies, 4178 participants, I^2=95.1%, range=2.6%-16.7%)	Critically low

Jarde A, *et al. BMJ Open* 2022; 12:e060906. doi: 10.1136/bmjopen-2022-060906

		Uganda: 7.3% (95% CI 4.7%-10.3%, 2 studies, 390 participants, I^2=9.9%, range=5.4%-8.5%)<br><br>Kazakhstan: 7.1% (95% CI 5.1%-9.4%, 1 study, 562 participants)<br>Romania: 18.4% (95% CI 13.6%-23.7%, 1 study, 228 participants)<br>Turkey: 7.8% (95% CI 6.8%-8.8%, 3 studies, 2773 participants, I2=0%, range=7.9%-8.6%)<br>Georgia: 12.4% (95% CI 7.4%-18.5%, 1 study, 137 participants)<br><br>Egypt: 22.8% (95% CI 15.2%-31.4%, 3 studies, 578 participants, I2=81.4%, range=15.8%-27.7%)<br>Iran: 17.8% (95% CI 12.5%-23.8%, 11 studies, 3134 participants, I2=93.3%, range=5.5%-40.0%)<br>Libya: 6.1% (95% CI 3.5%-9.4%, 1 study, 262 participants)<br>Pakistan: 22.0% (95% CI 12.8%-32.8%, 6 studies, 5201 participants, I2=98.8%, range=11.4%-39.6%)<br>Tunisia: 7.6% (95% CI 5.9%-9.6%, 1 study, 788 subject)<br>Yemen: 9.5% (95% CI 6.0%-13.8%, 1 study, 220 participants)<br><br>Guyana: 14.0% (95% CI 7.8%-21.6%, 1 study, 100 participants)<br>Mexico: 30.8% (95% CI 26.4%-35.3%, 10 studies, 192420 participants, I2=97.9%, range=19.3%-54.4%)<br><br>Brazil: 7.2% (95% CI 6.3%-8.1%, 12 study, 1726436 participants, I2=99.7%, range=3.3%-33.1%)<br>Peru: 4.8% (95% CI 1.7%-9.5%, 4 studies, 3983 participants, I2=96.8%, range=2.5%-11.1%)<br><br>Bangladesh: 10.6% (95% CI 7.2%-14.5%, 3 studies, 3010 participants, I2=85.9%, range=8.3%-12.8%)<br>India: 19.9% (95% CI 16.8%-23.2%, 26 studies, 27260 participants, I2=97.2%, range=5.1%-54.1%)<br>Sri Lanka: 24.1% (95% CI 16.6%-32.5%, 1 study, 112 participants)<br><br>China: 14.5% (95% CI 10.5%-19.0%, 14 studies, 19529 participants, I2=98.4%, range=2.7%-30.1%)<br>Fiji: 10.1% (95% CI 4.4%-17.7%, 3 studies, 1139 participants, I2=91.8%, range=5.2%-13.7%)<br>Indonesia: 14.8% (95% CI 12.2%-17.7%, 1 study, 634 participants)<br>Kiribati: 36.7% (95% CI 31.1%-42.5%, 1 study, 275 participants)<br>Malaysia: 26.9% (95% CI 17.8%-37.0%, 5 studies, 23438 participants)<br>Marshall Island: 45.2% (95% CI 32.9%-57.7%, 1 study, 62 participants)<br>Thailand: 7.5% (95% CI 6.2%-8.8%, 5 studies, 17862 participants, I2=81.6%, range=6.0%-16.3%)	
Shao-hua 2016	PTB +DM	Retreatment:<br>OR 2.05 (1.30-3.22, 3 studies, 499 participants, I^2=0%, range NR)<br>aOR 3.38 (1.56-7.29, 2 studies, n participants NR, I^2=75%, range NR)	Critically low
Tegegne 2018	TB +DM	Outcomes available for individual studies, but not pooled: Prevalence*: range 5% - 36%	High
Workneh 2017	TB +DM	Outcomes available for individual studies, but not pooled: Prevalence*: range 1.9% - 45%	Low
**TB+Mental disorders**			
Alene 2018	MDR-TB + Depression; MDR-TB +Anxiety; MDR-TB +Psychosis	Depression:<br>Overall: Prev. 25% (95% CI 14%-39%, 15 studies, n participants NR, I^2=98%, range= 3%-79%)<br>African region: 16% (95% CI 9%-24%, 3 studies, n participants NR, I^2 NR, range NR)<br>The Americas Region: 36% (95% CI 23%-50%, 3 studies, n participants NR, I^2 NR, range NR)<br>South-East Asia Region: 22% (95% CI 0%-60%, 3 studies, n participants NR, I^2 NR, range NR)<br>European region: 11% (95% CI 4%-21%, 3 studies, n participants NR, I^2 NR, range NR)<br>Eastern Mediterranean Region: 73% (95% CI 64%-81%, 2 studies, n participants NR, I^2 NR, range NR)<br>Western Pacific Region: 5% (95% CI 1%-12%, 1 study, , n participants NR, I^2 NR, range NR)	Critically low

		Anxiety:<br>Overall: Prev: 24% (95% CI 2%-57%, 3 studies, n participants NR, I^2=95%, range=12%-56%)<br>The Americas Region: 14% (95% CI 9%-21%, 2 studies, n participants NR, I^2 NR, range NR)<br>South-East Asia Region: 56% (95% CI 45%-66%, 1 studies, n participants NR, I^2 NR, range NR)<br><br>Psychosis:<br>(Overall includes a study from S.Korea)<br>African region: 12% (95% CI 8%-17%, 5 studies, n participants NR, I^2 NR, range NR)<br>The Americas Region: 11% (95% CI 7%-17%, 2 studies, n participants NR, I^2 NR, range NR)<br>South-East Asia Region: 10% (95% CI 5%-17%, 2 studies, n participants NR, I^2 NR, range NR)<br>European region: 6% (95% CI 0%-17%, 2 studies, n participants NR, I^2 NR, range NR)<br>Eastern Mediterranean Region: 7% (95% CI 1%-17%,1 studies, n participants NR, I^2 NR, range NR)	
Duko 2020	TB + Depression; MDR-TB +Depression	Prevalence: 45.19% (95% CI 38.04%-52.55%, 25 studies, 4903 participants, I^2=96.28%, range=15.56%-80.00%)<br>Women: 51.54% (95% CI 40.34%–62.60%, 17 studies, number of participants NR, I^2 = 92. 55%, range NR)<br>Men: 45.25% (95% CI 35.19%–55.71%, 17 studies, number of participants NR, I^2 = 95.09%, range NR)<br>MDR-TB: 52.34% (95% CI 38.09%-66.22%, 5 studies, number of participants NR, I^2=92.55%, range=NR)	High
Lee 2020	TB +Mental Disorders	Unsuccessful treatment: OR 2.13 (95%CI 0.85-5.37, 4 studies, 1196 participants, I^2=82%, range NR)<br>Loss to follow up: OR 1.90 (95%CI 0.33-10.91, 2 studies, 1139 participants, I^2=78%, range NR)<br>Non-adherence to treatment (measured by self-report, missed visits, pill count, or physiological tests): OR 1.60 (95% CI 0.84-3.02, 4 studies, 10851 participants, I^2=86%, range=0.94-3.67)<br>Outcomes available for individual studies, but not pooled: Prevalence*: Depression: range 37.5% - 53.9%; Mental disorder: range 18.9%-22.4%; Psychological distress: range 22% - 67.6%; PTSD: 29.6% (1 study), Mortality*:	Critically low
Rensburg 2020	TB +Mental illness (Depression, Anxiety, Alcohol use, and General Mental health)	Outcomes available for individual studies, but not pooled: Prevalence*: Depression: range 9.3% - 84%; Anxiety: range 2%-47.2%; Alcohol use: range 5% - 63%; Psychiatric comorbidity: range 3%; Psychological distress: range 22% - 83.6%; Poor mental quality: range 13.1% (1 study); Common mental disorder/Mental disorder: range 22.4%-38.3%	Critically low
Ruiz-Grosso 2020	TB +Depression	Mortality or loss to follow-up: OR = 4.26 (95% CI 2.33–7.79, 2 studies, 973 participants, I^2=0%, range=3.65-4.88)<br>Mortality: OR 2.85 (1.52-5.36, 2 studies, 973 participants, I^2=0%, range=1.76-2.99)<br>Loss to follow up: OR 8.70 (4.95-9.09, 2 studies, 973 participants, I^2=0%, range=4.95-9.09)<br>Non-adherence to TB treatment: OR 1.38 (0.70-2.72, 3 studies, 9349 participants, I^2=94.36%, range=0.92-3.67)	Critically low
**TB+HCV**			
Behzadifar 2019	TB +HCV	Africa: 11% (95% CI 1%-23%, 3 studies, 327 participants, I^2=93.9%, range=NR)	Moderate
**TB+other**			
Basham 2020	TB + cardiovascular disease	Outcomes available for individual studies, but not pooled: Mortality*: range of ORs 2.50 - 3.01	Critically low
Leung 2020	TB +Lung cancer; TB +non-Hodgkin's lymphoma; TB +Leukaemia	Upper middle-income countries<br>Lung cancer: RR 1.53 (95% CI 1.25-1.87, 9 studies, number of participants NR, I^2=94.6%, range NR)<br>non-Hodgkin's lymphoma: RR 1.70 (95% CI 1.13-2.56, 1 study, number of participants NR, I^2=NA)<br>leukaemia: RR 1.61 (95% CI 1.13-2.29, 1 study, number of participants NR, I^2= NA)	Low
Rehm 2009	TB+ Alcohol Use Disorder	Outcomes available for individual studies, but not pooled: Prevalence*:	Critically low

Note: Quality was assessed using the AMSTAR2 tool. aOR: adjusted OR; DM: Diabetes Mellitus; DR-TB: Drug resistant TB; HCV: Hepatitis C Virus; HIV: Human immunodeficiency virus; LMIC: Low- and middle-income countries; MDR-TB: Multidrug resistant TB; NR: Not reported; OR: Odds Ratio; prev.: prevalence; PTB: Pulmonary TB; RR: Relative Risk; SSA: Sub-Saharan Africa; Tuberculosis; XDR-TB: Extensively drug-resistant TB.

* Outcomes available for individual studies, but not pooled. Range of effect estimates reported.

† Includes one study focused on children

Jarde A, *et al. BMJ Open* 2022; 12:e060906. doi: 10.1136/bmjopen-2022-060906

Supplementary Table 5: Conditions that were considered chronic (and therefore included) or not (and therefore excluded) for this review

Condition/risk factor	Should be considered as a comorbidity in this review?	Description or details
**Included**		
Acquired immunodeficiency syndrome (AIDS)	Yes	
Anxiety	Yes	Clinical diagnostic of an anxiety disorder or assessed with a validated scale
Arthritis	Yes	
Asthma	Yes	
Autoimmune diseases	Yes	
Cancer	Yes	Any type of cancer
Cardiovascular disease	Yes	
cerebrovascular accident	Yes	
cerebrovascular accident	Yes	Cerebrovascular disease is a form of cardiovascular disease
chronic airflow obstruction	Yes	As a proxy for COPD
Chronic kidney disease / Chronic renal failure	Yes	No if acute renal failure- Yes if chronic renal failure
Chronic liver disease / Cirrhosis / Chronic hepatic dysfunction	Yes	
Chronic lung disease	Yes	
Chronic obstructive pulmonary disease (COPD)	Yes	COPD is form of chronic lung disease
Cor pulmonale	Yes	
Depression	Yes	Clinical diagnostic or assessed with a validated scale (e.g. PHQ-9)
Diabetes mellitus (DM)	Yes	
hearing defect	Yes	
Heart disease / Cardiopathies	Yes	
Heart failure	Yes	
Hepatitis B virus (HBV)	Yes	
Hepatitis C virus (HCV)	Yes	
HIV	Yes	
hyperthyroidism	Yes	
hypothyroidism	Yes	
Mental disorder	Yes	Umbrella term for conditions such as PTSD, OCD, depression, anxiety disorders, etc.
Obsessive compulsive disorder (OCD)	Yes	
Panic disorder	Yes	

Jarde A, *et al. BMJ Open* 2022; 12:e060906. doi: 10.1136/bmjopen-2022-060906

pneumoconiosis	Yes	
Post-traumatic stress disorder (PTSD)	Yes	
seizures (cause not determined)	Yes if called epilepsy	Yes only if called epilepsy
Substance use / drug abuse	Yes	usually reserved for illicit substances
T. pallidum	Yes	
Unstable angina	Yes	
**Excluded**		
Acute Respiratory Distress Syndrome	No	Acute
Anaemia	No	
Aspergilius coinfection	No	complications of HIV
BMI	No	
Candida coinfection	No	complications of HIV
cardiomyopathy	No	Can be acute and/or reversible
Cavitary disease	No	
Chronic corticosteroid therapy	No	Treatment
Chronic diarrhoea	no	Unless it’s inflammatory bowel disorders, no.
cryptoccocus	No	complications of HIV
cryptococcal IRIS (immune reconstitution inflammatory syndrome) adenitis	No	
deep venous thrombosis	NO	
dermatitis	No	
Drinking alcohol	No	No, unless it is something like 'harmful use of alcohol' or 'alcohol dependence'
drug-induced hepatotoxicity/ liver injury	No	
Dyslipidemia	No	
haemorrhoids/fistula-in-ano	No	
HTLV (Human T-Cell LymphotropicVirus)	No	complications of HIV
Hypertension	No	
hypokalaemia	No	
hyponatremia	No	complications of HIV
Intestinal parasites	No	
Leukopenia	no	
Malnourishment	No	
Obesity	No	

Jarde A, *et al. BMJ Open* 2022; 12:e060906. doi: 10.1136/bmjopen-2022-060906

pancreatitis	No	Unless Long term pancreatitis and it is clearly specified
Pneumonia	No	Acute condition
pneumothorax	No	
Pulmonary edema	No	Symptom
pulmonary fungal infection	No	complications of HIV
sacroiliitis	NO	
scabies	No	
severe epistaxis	No	
Skin conditions	no	
smoking	No	Would not fall under ‘substance abuse’, even if it is reported as ‘high dependence smoking’
visual impairment (reported as a symptom of DM)	No (reported as a complication)	

Jarde A, *et al. BMJ Open* 2022; 12:e060906. doi: 10.1136/bmjopen-2022-060906

Supplementary Table 6: AMSTAR2 assessment details for each study

**Study Id**	**1**	**2**	**3**	**4**	**5**	**6**	**7**	**8**	**9**	**10**	**11**	**12**	**13**	**14**	**15**	**16**	**confidence rating**
Duko 2020	Yes	**Partial Yes**	Yes	**Partial Yes**	Yes	Yes	**Partial Yes**	Yes	**Yes**	No	**Yes**	Yes	**Yes**	Yes	**Yes**	Yes	High
Alemu 2020	Yes	**Partial Yes**	Yes	**Partial Yes**	Yes	Yes	**No**	Yes	**Yes**	No	**Yes**	Yes	**Yes**	Yes	**Yes**	Yes	Low
Arega 2020	Yes	**No**		**Partial Yes**	Yes	Yes	**No**	Yes	**Yes**	No	**Yes**	Yes	**Yes**	Yes	**Yes**	Yes	Critically low
Huddart 2020	Yes	**No**	Yes	**Partial Yes**	Yes	Yes	**Partial Yes**	Partial Yes	**No**	No	**No**	Yes	**Yes**	Yes	**No**	Yes	Critically low
Huang 2020	Yes	**Partial Yes**	Yes	**No**	Yes	Yes	**No**	Yes	**Yes**	No	**Yes**	Yes	**Yes**	Yes	**Yes**	Yes	Critically low
Edessa 2020	Yes	**Partial Yes**	Yes	**Partial Yes**	Yes	Yes	**Partial Yes**	Yes	**Partial Yes**	No		Yes	**Yes**	Yes	**Yes**	Yes	High
Basham 2020	Yes	**No**	Yes	**Partial Yes**	Yes	Yes	**No**	Yes	**Partial Yes**	No	**No**	Yes	**Yes**	Yes			Critically low
Bisson 2020	Yes	**Yes**	Yes	**Partial Yes**	No	No	**Partial Yes**	No	**No**	No	**NA**	NA	**NA**	No	**NA**	Yes	Low
Huangfu 2019	Yes	**Partial Yes**	Yes	**Partial Yes**	Yes	Yes	**Partial Yes**	Partial Yes	**Partial Yes**	No	**Yes**	Yes	**Yes**	Yes	**No**	Yes	Low
Bastos 2019	Yes	**Yes**	Yes	**Partial Yes**	Yes	Yes	**No**	Partial Yes	**No**	No	**NA**	NA	**NA**	No	**NA**	Yes	Critically low
Endalamaw 2019		**Partial Yes**	Yes	**Partial Yes**	No	Yes	**Partial Yes**	Partial Yes	**Partial Yes**	No	**Yes**	Yes	**Yes**	No	**Yes**	Yes	Moderate
Chem 2019	Yes	**Partial Yes**	Yes	**Partial Yes**	Yes	No	**Partial Yes**	Yes	**Partial Yes**	Yes	**Yes**	Yes	**No**	Yes	**No**	Yes	Critically low
Alebel 2019	Yes	**Yes**	Yes	**Partial Yes**	No	Yes	**No**	Partial Yes	**Yes**	No	**Yes**	Yes	**Yes**	Yes	**Yes**	Yes	Low
Behzadifar 2019	Yes	**Partial Yes**	Yes	**Partial Yes**	Yes	No	**Partial Yes**	Partial Yes	**Yes**	No	**Yes**	Yes	**Yes**	Yes	**Yes**	Yes	Moderate
Gelaw 2019	Yes	**Partial Yes**	Yes	**Partial Yes**	Yes	Yes	**Partial Yes**	Yes	**Yes**	No	**Yes**	Yes	**Yes**	Yes	**Yes**	Yes	High
Alene 2018		**Partial Yes**	Yes	**Partial Yes**	Yes	Yes	**Partial Yes**	Yes	**No**	No	**No**	No	**No**	No	**No**	Yes	Critically low
Almeida 2018	Yes	**Partial Yes**	Yes	**Partial Yes**	Yes	Yes	**Partial Yes**	Partial Yes	**No**	No	**No**	No	**No**	No	**No**	Yes	Critically low
Eshetie 2018		**No**	Yes	**Partial Yes**	Yes	Yes	**No**	Yes	**Yes**	No	**Yes**	Yes	**Yes**	Yes	**No**	Yes	Critically low
Gao 2013	Yes	**No**	Yes	**Partial Yes**	Yes	Yes	**Partial Yes**	Partial Yes	**No**	No	**Yes**	No	**No**	Yes	**No**	Yes	Critically low
	Yes	**No**		**Partial Yes**	No	No	**Partial Yes**	Partial Yes	**No**	No	**NA**	NA	**NA**	No	**NA**	No	Critically low
Baker 2011	Yes	**No**	Yes	**Partial Yes**	No	Yes	**Partial Yes**	Partial Yes	**No**	No	**Yes**	Yes	**No**	Yes	**Yes**	Yes	Critically low
Jeon 2010	Yes	**No**	Yes	**Partial Yes**	No	Yes	**No**	Yes	**No**	No	**NA**	NA	**NA**	No	**NA**	Yes	Critically low
Gao 2010	Yes	**No**	Yes	**Partial Yes**	No	No	**Yes**	Yes	**No**	No	**No**	No	**No**	Yes	**No**	Yes	Critically low

	Yes	**Partial Yes**	Yes	**Partial Yes**	Yes	Yes	**Partial Yes**	Yes	**No**	No	**No**	No	**No**	Yes	**Yes**	Yes	Critically low
Leung 2020	Yes	**Partial Yes**	Yes	**Partial Yes**	Yes	Yes	**Yes**	Yes	**Partial Yes**	No	**No**	Yes	**Yes**	Yes	**Yes**	Yes	Low
Rensburg 2020	Yes	**No**	Yes	**Partial Yes**	Yes	No	**No**	Partial Yes	**No**	No	**NA**	NA	**NA**	No	**NA**	Yes	Critically low
Han 2016	Yes	**No**	Yes	**Partial Yes**	Yes	Yes	**Partial Yes**	No	**No**	No	**No**	No	**No**	Yes	**Yes**	No	Critically low
Lee 2020	Yes	**Yes**	Yes	**Partial Yes**	Yes	Yes	**Partial Yes**	Yes	**Yes**	No	**Yes**	No	**No**	No	**No**	Yes	Critically low
Purmohamad 2020	Yes	**No**	Yes	**Partial Yes**	Yes	Yes	**Partial Yes**	Partial Yes	**No**	No	**No**	No	**No**	Yes	**Yes**	Yes	Critically low
Rajendran 2020	Yes	**No**	Yes	**Partial Yes**	No	No	**Yes**	No	**No**	No	**NA**	NA	**NA**	No	**NA**	Yes	Critically low
Ruiz-Grosso 2020	Yes	**Partial Yes**	Yes	**No**	Yes	Yes	**No**	No	**No**	No	**Yes**	Yes	**No**	No	**No**	Yes	Critically low
Pourakbari 2019	Yes	**No**	Yes	**Partial Yes**	Yes	Yes	**No**	Yes	**No**	No	**Yes**	No	**Yes**	Yes	**Yes**	Yes	Critically low
Lutfiana 2019	Yes	**No**	Yes	**Partial Yes**	No	No	**Partial Yes**	Partial Yes	**No**	No	**NA**	NA	**NA**	No	**NA**	Yes	Critically low
Wang 2019		**No**	Yes	**Partial Yes**	Yes	Yes	**Partial Yes**	Yes	**No**	No	**Yes**	No	**No**	Yes	**Yes**	Yes	Critically low
Uchida 2019	Yes	**No**	Yes	**No**	Yes	No	**No**	Partial Yes	**No**	No	**NA**	NA	**NA**	No	**NA**	Yes	Critically low
Mekonnen 2019	Yes	**Partial Yes**	Yes	**Partial Yes**	Yes	Yes	**Partial Yes**	Yes	**Partial Yes**	No	**Yes**	Yes	**Yes**	Yes	**Yes**	Yes	High
Teweldemedhin 2018	Yes	**No**	Yes	**No**	Yes	No	**Yes**	Partial Yes	**No**	No	**Yes**	No	**No**	Yes	**Yes**	Yes	Critically low
Tegegne 2018	Yes	**Partial Yes**	Yes	**Partial Yes**	Yes	Yes	**Yes**	Partial Yes	**Yes**	No	**Yes**	Yes	**Yes**	Yes	**Yes**	Yes	High
Tesfaye 2018	Yes	**Partial Yes**	Yes	**Partial Yes**	Yes	Yes	**No**	Partial Yes	**No**	No	**Yes**	Yes	**No**	Yes	**Yes**	Yes	Critically low
Seid 2018	Yes	**No**	Yes	**Partial Yes**	Yes	Yes	**Partial Yes**	Partial Yes	**No**	No	**No**	No	**No**	No	**Yes**	Yes	Critically low
Pormohammad 2018	Yes	**No**	Yes	**Partial Yes**	Yes	Yes	**Partial Yes**	Partial Yes	**No**	No	**No**	Yes	**Yes**	No	**Yes**	Yes	Critically low
Samuels 2018	Yes	**Partial Yes**	Yes	**Partial Yes**	Yes	Yes	**Partial Yes**	Yes	**No**	No	**Yes**	Yes	**Yes**	Yes	**Yes**	Yes	Low
Lukoye 2015	Yes	**No**	Yes	**Partial Yes**	Yes	Yes	**Partial Yes**	Yes	**No**	No	**No**	No	**No**	No	**Yes**	Yes	Critically low
Mesfin 2014	Yes	**No**	Yes	**Partial Yes**	Yes	Yes	**Partial Yes**	Yes	**Partial Yes**	No	**No**	No	**No**	Yes	**Yes**	Yes	Critically low
Straetemans 2011	Yes	**Partial Yes**	Yes	**Partial Yes**	No	No	**Yes**	Partial Yes	**No**	No	**Yes**	No	**No**	Yes	**Yes**	Yes	Critically low
Waitt 2011	Yes	**No**	Yes	**No**	No	No	**Partial Yes**	Partial Yes	**No**	No	**No**	NA	**No**	No	**No**	No	Critically low
Reddy 2010	Yes	**No**	No	**Partial Yes**	Yes	Yes	**Yes**	No	**No**	No	**NA**	NA	**NA**	No	**NA**	Yes	Critically low
Rehm 2009	Yes	**No**	Yes	**Partial Yes**	Yes	No	**No**	Partial Yes	**No**	No	**NA**	NA	**NA**	No	**NA**	Yes	Critically low

Sotgiu 2009	Yes	**No**	Yes	**Partial Yes**	Yes	No	**No**	Partial Yes	**No**	No	**NA**	NA	**NA**	No	**NA**	Yes	Critically low
Noubiap 2019		**Partial Yes**	Yes	**Partial Yes**	Yes	Yes	**Partial Yes**	Partial Yes	**No**	No	**No**	Yes	**Yes**	Yes	**Yes**	Yes	Critically low
Wu 2016	Yes	**No**	Yes	**Partial Yes**	No	No	**Partial Yes**	Yes	**No**	No	**Yes**	Yes	**No**	Yes	**No**	Yes	Critically low
Gautam 2021	Yes	**Partial Yes**	Yes	**Partial Yes**	Yes	Yes	**Yes**	Partial Yes	**Partial Yes**	No	**No**	No	**Yes**	Yes	**Yes**	Yes	Low
Chen 2013	Yes	**No**	Yes	**Partial Yes**	Yes	Yes	**Partial Yes**	Partial Yes	**No**	No	**No**	Yes	**Yes**	Yes	**Yes**	No	Critically low
McMurry 2018	Yes	**Partial Yes**	Yes	**Partial Yes**	**Yes**	No	**Partial Yes**	Partial Yes	**No**	No	**NA**	NA	**NA**	No	**NA**	yes	Low
Workneh 2017	Yes	**Partial Yes**	**Yes**	**Partial Yes**	No	No	**Partial Yes**	**Partial Yes**	**No**	No	**NA**	NA	**NA**	no	**NA**	Yes	Low
Shao-hua 2016	Yes	**No**	Yes	**Partial Yes**	Yes	Yes	**Partial Yes**	Partial Yes	**No**	No	**No**	No	**No**	Yes	**No**	No	Critically low

Note: Items assessing critical domains are bolded.
1. Did the research questions and inclusion criteria for the review include the components of PICO?
2. Did the report of the review contain an explicit statement that the review methods were established prior to conduct of the review and did the report justify any significant deviations from the protocol
3. Did the review authors explain their selection of the study designs for inclusion in the review?
4. Did the review authors use a comprehensive literature search strategy?
5. Did the review authors perform study selection in duplicate?
6. Did the review authors perform data extraction in duplicate?
7. Did the review authors provide a list of excluded studies and justify the exclusions?
8. Did the review authors describe the included studies in adequate detail?
9. Did the review authors use a satisfactory technique for assessing the risk of bias (RoB) in individual studies that were included in the review?
10. Did the review authors report on the sources of funding for the studies included in the review?
11. If meta-analysis was performed did the review authors use appropriate methods for statistical combination of results?
12. If meta-analysis was performed, did the review authors assess the potential impact of RoB in individual studies on the results of the meta-analysis or other evidence synthesis?
13. Did the review authors account for RoB in individual studies when interpreting/discussing the results of the review?
14. Did the review authors provide a satisfactory explanation for, and discussion of, any heterogeneity observed in the results of the review?
15. If they performed quantitative synthesis did the review authors carry out an adequate investigation of publication bias (small study bias) and discuss its likely impact on the results of the review?
16. Did the review authors report any potential sources of conflict of interest, including any funding they received for conducting the review?

Jarde A, *et al. BMJ Open* 2022; 12:e060906. doi: 10.1136/bmjopen-2022-060906
