# Supplementary material for: Prevalence and risks of tuberculosis multimorbidity in low-income and middle-income countries: a meta-review
Source: BMJ Open. 2022 Sep 28;12(9):e060906. doi: 10.1136/bmjopen-2022-060906 (PMC9528681; doi:10.1136/bmjopen-2022-060906)
Supplement: Supplementary data [file bmjopen-2022-060906supp002.pdf]

Medline (October 23rd 2020)

# ▲	Searches	Results
1	exp Noncommunicable Diseases/ or ((Non-communicable or Noncommunicable or Non-infectious) adj (disease* or condition* or illness*)).mp.	11685
2	exp Chronic Disease/ or ((chronic or long-term) adj (disease* or condition* or illness*)).mp.	343000
3	exp Heart Diseases/ or (heart adj (disease* or disorder* or failure)).mp. or (cardiac adj (disease* or disorder* or failure)).mp.	1252363
4	exp Cardiovascular Diseases/ or (cardiovascular adj (disease* or disorder* or failure)).mp.	2482639
5	exp Coronary Disease/ or (coronary adj (disease* or disorder* or failure)).mp.	224638
6	exp Cerebrovascular Disorders/ or (cerebrovascular adj (disease* or disorder* or insufficienc* or occlusion*)).mp. or (vascular adj (disease* or disorder*)).mp. or (carotid* adj (disease* or disorder*)).mp.	460609
7	exp Peripheral Arterial Disease/ or (arter* adj (disease* or disorder*)).mp.	172699
8	exp Rheumatic Heart Disease/ or exp Heart Defects, Congenital/ or (heart adj3 (malform* or defect* or congeni*)).mp.	182266
9	exp Venous Thrombosis/ or ((deep vein or deep venous) adj thrombos*).mp. or phlebothrombos*.mp.	68358
10	exp Pulmonary Embolism/ or (pulmonar* adj (thromboembolism* or embolism* or disease* or disorder*)).mp.	138141
11	exp Stroke/ or stroke.mp.	320701
12	exp Neoplasms/ or Cancer*.mp. or neoplas*.mp. or tumor*.mp.	4416189
13	exp Lung Diseases/ or exp Respiratory Tract Diseases/ or exp Lung Diseases, Obstructive/ or ((lung* or respiratory or pulmonar* or airflow or airway) adj2 (disease* or obstruct* or hypersensitiv*)).mp. or exp Asthma/ or asthma*.mp. or exp Pulmonary Disease, Chronic Obstructive/ or exp Respiratory Hypersensitivity/	1474479
14	exp Diabetes Mellitus/ or diabet*.mp.	710026
15	exp Autoimmune Diseases/ or ((autoimmun* or auto immun* or autoaggress* or auto aggress*) adj (disorder* or disease*)).mp.	516672
16	exp Metabolic Syndrome/ or exp Metabolic Diseases/ or ((metabolic or insulin resistance) adj (disorder* or disease* or syndrome*)).mp.	1067250
17	exp Obesity/ or obes*.mp.	365459
18	exp Osteoporosis/ or osteoporo*.mp. or bone loss.mp. or exp osteolysis/ or osteolysis.mp. or bone resorption.mp.	153480

19	exp Parkinson disease/ or parkinson*.mp. or paralysis agitans.mp.	131499
20	exp Arthritis/ or arthriti*.mp. or polyarthriti*.mp. or rheumarthriti*.mp.	321229
21	exp Kidney Diseases/ or (kidney adj (disease* or disorder*)).mp.	544657
22	exp Liver Diseases/ or (liver adj (disease* or disorder* or dysfunction*)).mp.	591670
23	exp Hypertension/ or high blood pressure*.mp. or hypertens*.mp.	522886
24	exp Hyperlipidemias/ or hyperlipem*.mp. or hyperlipidem*.mp. or lipem*.mp. or lipidem*.mp.	84750
25	exp Hypercholesterolemia/ or ((high* or elevat*) adj cholesterol*).mp. or hypercholesterem*.mp. or hypercholesterolem*.mp.	50873
26	exp Hypertriglyceridemia/ or hypertriglyceridem*.mp.	15254
27	exp Thyroid Diseases/ or (thyroid adj (disease* or disorder*)).mp. or exp Hyperthyroidism/ or hyperthyroid*.mp. or exp Hypothyroidism/ or hypothyroid*.mp. or ((thyroid-stimulating hormone* or tsh) adj deficien*).mp.	166497
28	exp Motor Neuron Disease/ or motor neuron* disease*.mp. or lateral scleros*.mp. or motor system disease*.mp.	38409
29	exp Multiple Sclerosis/ or multiple sclerosis.mp. or disseminated sclerosis.mp.	84720
30	exp Emphysema/ or emphysema*.mp.	38522
31	exp Bronchitis/ or bronchit*.mp.	41201
32	1 or 2 or 3 or 4 or 5 or 6 or 7 or 8 or 9 or 10 or 11 or 12 or 13 or 14 or 15 or 16 or 17 or 18 or 19 or 20 or 21 or 22 or 23 or 24 or 25 or 26 or 27 or 28 or 29 or 30 or 31	10292435
33	exp Mental Disorders/ or exp Psychotic Disorders/ or ((mental* or psychiatr* or psycho*) adj (disorder* or disease* or illness*)).mp.	1313460
34	exp Depressive Disorder, Major/ or exp Depression/ or Depress*.mp. or MDD.mp.	552268
35	exp Anxiety Disorders/ or exp Anxiety/ or anxi*.mp.	289411
36	exp Phobic Disorders/ or phobi*.mp.	17668
37	exp Schizophrenia/ or schizophreni*.mp. or hebephreni*.mp.	150158
38	exp Somatoform Disorders/ or ((somatoform* or somati* or medically unexplained or briquet or pain) adj (disorder* or syndrome* or symptom*)).mp. or exp Medically Unexplained Symptoms/	49713
39	exp Dissociative Disorders/ or (dissociative adj (disorder* or hysteri* or reaction*)).mp. or dissociation*.mp.	115459
40	exp Hysteria/ or hysteri*.mp.	6074
41	exp Mood Disorders/ or ((affective* or mood*) adj (disorder* or disease* or illness* or symptom*)).mp.	158894

42	exp Stress Disorders, Post-Traumatic/ or PTSD.mp. or ((post trauma* or posttrauma*) adj (stress* or neurose*)).mp. or combat disorder*.mp. or war disorder*.mp.	48359
43	exp Cognition Disorders/ or ((cognitive or cognition or mental or neurocognitive) adj (dysfunction* or decline* or impairment* or deterioration* or disorder* or illness* or disease*)).mp.	377493
44	exp Personality Disorders/ or personality disorder*.mp.	48830
45	exp "Disruptive, Impulse Control, and Conduct Disorders"/ or impulse control disorder*.mp. or intermittent explosive disorder*.mp.	9781
46	exp "Feeding and Eating Disorders"/ or ((eating or appetite or feeding) adj disorder*).mp.	38361
47	exp Bipolar Disorder/ or ((bipolar or mani*) adj (disorder* or illness* or disease*)).mp.	51927
48	exp Obsessive-Compulsive Disorder/ or OCD*.mp. or ((obsess*-compulsi* or obsess* or compulsi*) adj (disorder* or illness* or disease* or neuros*)).mp.	22060
49	exp Panic Disorder/ or (panic adj (attack* or disorder*)).mp.	12638
50	exp Agoraphobia/ or agoraphobi*.mp.	4210
51	exp Neurotic Disorders/ or neuros*.mp. or neurotic disorder*.mp. or psychoneuros*.mp.	190989
52	33 or 34 or 35 or 36 or 37 or 38 or 39 or 40 or 41 or 42 or 43 or 44 or 45 or 46 or 47 or 48 or 49 or 50 or 51	2107749
53	exp Communicable Diseases/ or ((communic* or contag* or transmi* or infect*) adj (disease* or infection* or illness*)).mp.	212955
54	exp Bacterial Infections/ or bacteri* infection*.mp.	916393
55	exp Conjunctivitis/ or conjunctivitis.mp.	24690
56	exp HIV/ or hiv.mp. or Human immuno deficiency virus.mp.	362332
57	exp Acquired Immunodeficiency Syndrome/ or AIDS.mp. or immunodeficiency associated virus.mp. or immun* deficiency associated virus.mp. or acquired immunodeficiency syndrome*.mp. or acquired immun* deficiency syndrome*.mp.	226679
58	exp Buruli Ulcer/ or Bairnsdale.mp. or Buruli.mp.	1104
59	exp Onchocerciasis/ or onchocer*.mp.	5978
60	hepatitis.mp. or exp Hepatitis B/ or exp Hepatitis C/	256450
61	exp Leishmaniasis/ or leishmania*.mp.	37940
62	exp Leprosy/ or lepros*.mp. or hansen*.mp.	31193
63	exp Elephantiasis, Filarial/ or elephantias*.mp. or filaria*.mp.	14354
64	exp Trachoma/ or egyptian ophthalmia*.mp. or trachoma*.mp.	20432
65	exp Chikungunya Fever/ or chickungunya.mp. or chikungunya.mp.	5766

66	exp Taeniasis/ or taenia*.mp.	12366
67	exp Cysticercosis/ or cysticercos*.mp.	7146
68	exp Echinococcosis/ or hydatid*.mp. or echinococc*.mp.	29913
69	exp Chagas Disease/ or trypanosom*.mp. or chagas.mp.	44250
70	exp Trypanosomiasis/ or sleeping sickness.mp.	23416
71	exp Encephalitis, Japanese/ or (japanese adj3 encephalitis).mp.	5847
72	exp Syphilis/ or syphilis.mp.	37477
73	53 or 54 or 55 or 56 or 57 or 58 or 59 or 60 or 61 or 62 or 63 or 64 or 65 or 66 or 67 or 68 or 69 or 70 or 71 or 72	1872806
74	exp Tuberculosis/	192915
75	Tuberculos*.mp.	256334
76	TB.mp.	56951
77	koch*.mp.	9469
78	exp Tuberculosis/ or Tuberculos*.mp. or TB.mp. or koch*.mp.	283447
79	(multiple adj (ill* or disease* or condition* or syndrom* or disorder*)).mp.	5522
80	((Cooccur* or co-occur* or coexist* or co-exist* or multipl* or concord* or discord* or long-term or physical*) adj3 (disease* or ill* or care or condition* or disorder* or health* or medication* or symptom* or syndrom*)).mp.	269814
81	(comorbid* or multimorbid* or co-occurren* or co-morbid* or Multidisease* or multi-disease*).mp.	268450
82	(comorbid* or multimorbid* or co-occurren* or co-morbid* or multi-morbid* or Multidisease* or multi-disease*).mp.	268954
83	exp Comorbidity/ or exp Multimorbidity/ or exp Multiple Chronic Conditions/	111238
84	79 or 80 or 81 or 82 or 83	519584
85	exp "Systematic Review"/	137409
86	"systematic review*".m_titl.	134940
87	exp Meta-Analysis/	121268
88	"meta-analys*".m_titl.	115466
89	exp "Systematic Review"/ or "systematic review*".m_titl. or exp Meta-Analysis/ or "meta-analys*".m_titl.	260449
90	32 or 52 or 73	13060984
91	(32 or 52 or 73) and 78	228989
92	(32 or 52 or 73) and 78 and 84	4072

93	(32 or 52 or 73) and 78 and 84 and 89	89
94	exp Animals/ not exp Humans/	4747614
95	((32 or 52 or 73) and 78 and 84) not 94	4004
96	((32 or 52 or 73) and 78 and 84 and 89) not 94	89
97	((32 or 52 or 73) and 78 and 89) not 94	1701

Embase (October 23rd 2020)

# ▲	Searches	Results
1	exp non communicable disease/ or ((Non-communicable or Noncommunicable or Non-infectious) adj (disease* or condition* or illness*)).mp.	16547
2	exp chronic disease/ or ((chronic or long-term) adj (disease* or condition* or illness*)).mp.	268915
3	exp heart disease/ or (heart adj (disease* or disorder* or failure)).mp. or (cardiac adj (disease* or disorder* or failure)).mp.	1935434
4	exp cardiovascular disease/ or (cardiovascular adj (disease* or disorder* or failure)).mp.	4171750
5	exp coronary artery disease/ or (coronary adj (disease* or disorder* or failure)).mp.	338836
6	exp cerebrovascular disease/ or (cerebrovascular adj (disease* or disorder* or insufficienc* or occlusion*)).mp. or (vascular adj (disease* or disorder*)).mp. or (carotid* adj (disease* or disorder*)).mp.	760319
7	exp peripheral occlusive artery disease/ or (arter* adj (disease* or disorder*)).mp.	427607
8	exp rheumatic heart disease/ or exp congenital heart malformation/ or (heart adj3 (malform* or defect* or congeni*)).mp.	187115
9	exp vein thrombosis/ or ((deep vein or deep venous) adj thrombos*).mp. or phlebothrombos*.mp.	137063
10	exp lung embolism/ or (pulmonar* adj (thromboembolism* or embolism* or disease* or disorder*)).mp.	205531
11	exp cerebrovascular accident/ or stroke.mp.	513128
12	exp neoplasm/ or Cancer*.mp. or neoplas*.mp. or tumor*.mp.	5810559
13	exp lung disease/ or exp respiratory tract disease/ or exp chronic obstructive lung disease/ or ((lung* or respiratory or pulmonar* or airflow or airway) adj2 (disease* or obstruct* or hypersensitiv*)).mp. or exp asthma/ or asthma*.mp. or exp chronic obstructive lung disease/ or exp respiratory tract allergy/	2529309
14	exp diabetes mellitus/ or diabet*.mp.	1165861
15	exp autoimmune disease/ or ((autoimmun* or auto immun* or autoaggress* or auto aggress*) adj (disorder* or disease*)).mp.	628015
16	exp metabolic syndrome X/ or exp metabolic disorder/ or ((metabolic or insulin resistance) adj (disorder* or disease* or syndrome*)).mp.	2728282
17	exp obesity/ or obes*.mp.	632387
18	exp osteoporosis/ or osteoporo*.mp. or bone loss.mp. or exp osteolysis/ or osteolysis.mp. or bone resorption.mp.	237604
19	exp Parkinson disease/ or parkinson*.mp. or paralysis agitans.mp.	212471

20	exp arthritis/ or arthriti*.mp. or polyarthriti*.mp. or rheumarthriti*.mp.	510423
21	exp kidney disease/ or (kidney adj (disease* or disorder*)).mp.	973568
22	exp liver disease/ or (liver adj (disease* or disorder* or dysfunction*)).mp.	1021173
23	exp hypertension/ or high blood pressure*.mp. or hypertens*.mp.	1010094
24	exp hyperlipidemia/ or hyperlipem*.mp. or hyperlipidem*.mp. or lipem*.mp. or lipidem*.mp.	170635
25	exp hypercholesterolemia/ or ((high* or elevat*) adj cholesterol*).mp. or hypercholesterem*.mp. or hypercholesterolem*.mp.	90843
26	exp hypertriglyceridemia/ or hypertriglyceridem*.mp.	31673
27	exp thyroid disease/ or (thyroid adj (disease* or disorder*)).mp. or exp hyperthyroidism/ or hyperthyroid*.mp. or exp hypothyroidism/ or hypothyroid*.mp. or ((thyroid-stimulating hormone* or tsh) adj deficien*).mp.	238358
28	exp motor neuron disease/ or motor neuron* disease*.mp. or lateral scleros*.mp. or motor system disease*.mp.	52581
29	exp multiple sclerosis/ or multiple sclerosis.mp. or disseminated sclerosis.mp.	143653
30	exp emphysema/ or emphysema*.mp.	52663
31	exp bronchitis/ or bronchit*.mp.	70185
32	1 or 2 or 3 or 4 or 5 or 6 or 7 or 8 or 9 or 10 or 11 or 12 or 13 or 14 or 15 or 16 or 17 or 18 or 19 or 20 or 21 or 22 or 23 or 24 or 25 or 26 or 27 or 28 or 29 or 30 or 31	13930977
33	exp mental disease/ or exp psychosis/ or ((mental* or psychiatr* or psycho*) adj (disorder* or disease* or illness*)).mp.	2252075
34	exp major depression/ or exp depression/ or Depress*.mp. or MDD.mp.	837980
35	exp anxiety disorder/ or exp anxiety/ or anxi*.mp.	518902
36	exp phobia/ or phobi*.mp.	35665
37	exp schizophrenia/ or schizophreni*.mp. or hebephreni*.mp.	209868
38	exp somatoform disorder/ or ((somatoform* or somati* or medically unexplained or briquet or pain) adj (disorder* or syndrome* or symptom*)).mp. or exp medically unexplained symptom/	82640
39	exp dissociative disorder/ or (dissociative adj (disorder* or hysteri* or reaction*)).mp. or dissociation*.mp.	142193
40	exp hysteria/ or hysteri*.mp.	7294
41	exp mood disorder/ or ((affective* or mood*) adj (disorder* or disease* or illness* or symptom*)).mp.	539796
42	exp posttraumatic stress disorder/ or PTSD.mp. or ((post trauma* or posttrauma*) adj (stress* or neurose*)).mp. or combat disorder*.mp. or war disorder*.mp.	68828

43	exp cognitive defect/ or ((cognitive or cognition or mental or neurocognitive) adj (dysfunction* or decline* or impairment* or deterioration* or disorder* or illness* or disease*)).mp.	802224
44	exp personality disorder/ or personality disorder*.mp.	64830
45	exp impulse control disorder/ or impulse control disorder*.mp. or intermittent explosive disorder*.mp.	12514
46	exp eating disorder/ or ((eating or appetite or feeding) adj disorder*).mp.	64625
47	exp bipolar disorder/ or ((bipolar or mani*) adj (disorder* or illness* or disease*)).mp.	73910
48	exp obsessive compulsive disorder/ or OCD*.mp. or ((obsess*-compulsi* or obsess* or compulsi*) adj (disorder* or illness* or disease* or neuros*)).mp.	46156
49	exp panic/ or (panic adj (attack* or disorder*)).mp.	25651
50	exp agoraphobia/ or agoraphobi*.mp.	7128
51	exp neurosis/ or neuros*.mp. or neurotic disorder*.mp. or psychoneuros*.mp.	284790
52	33 or 34 or 35 or 36 or 37 or 38 or 39 or 40 or 41 or 42 or 43 or 44 or 45 or 46 or 47 or 48 or 49 or 50 or 51	3032495
53	exp communicable disease/ or ((communic* or contag* or transmi* or infect*) adj (disease* or infection* or illness*)).mp.	227165
54	exp bacterial infection/ or bacteri* infection*.mp.	875706
55	exp conjunctivitis/ or conjunctivitis.mp.	42120
56	exp Human immunodeficiency virus/ or hiv.mp. or Human immuno deficiency virus.mp.	440016
57	exp acquired immune deficiency syndrome/ or AIDS.mp. or immunodeficiency associated virus.mp. or immun* deficiency associated virus.mp. or acquired immunodeficiency syndrome*.mp. or acquired immun* deficiency syndrome*.mp.	245085
58	exp Buruli ulcer/ or Bairnsdale.mp. or Buruli.mp.	1384
59	exp onchocerciasis/ or onchocer*.mp.	7089
60	hepatitis.mp. or exp hepatitis B/ or exp hepatitis C/	401827
61	exp leishmaniasis/ or leishmania*.mp.	45114
62	exp leprosy/ or lepros*.mp. or hansen*.mp.	32759
63	exp elephantiasis/ or elephantias*.mp. or filaria*.mp.	17315
64	exp trachoma/ or egyptian ophthalmia*.mp. or trachoma*.mp.	25318
65	exp chikungunya/ or chickungunya.mp. or chikungunya.mp.	8172
66	exp taeniasis/ or taenia*.mp.	13801
67	exp Cysticercosis/ or cysticercos*.mp.	5977

68	exp echinococcosis/ or hydatid*.mp. or echinococc*.mp.	30362
69	exp Chagas disease/ or trypanosom*.mp. or chagas.mp.	47764
70	exp trypanosomiasis/ or sleeping sickness.mp.	25238
71	exp Japanese encephalitis/ or (japanese adj3 encephalitis).mp.	7060
72	exp syphilis/ or syphilis.mp.	32633
73	53 or 54 or 55 or 56 or 57 or 58 or 59 or 60 or 61 or 62 or 63 or 64 or 65 or 66 or 67 or 68 or 69 or 70 or 71 or 72	2039904
74	exp tuberculosis/	193084
75	Tuberculos*.mp.	245295
76	TB.mp.	72736
77	koch*.mp.	12432
78	exp tuberculosis/ or Tuberculos*.mp. or TB.mp. or koch*.mp.	284028
79	(multiple adj (ill* or disease* or condition* or syndrom* or disorder*)).mp.	7515
80	((Cooccur* or co-occur* or coexist* or co-exist* or multipl* or concord* or discord* or long-term or physical*) adj3 (disease* or ill* or care or condition* or disorder* or health* or medication* or symptom* or syndrom*)).mp.	468523
81	(comorbid* or multimorbid* or co-occurren* or co-morbid* or Multidisease* or multi-disease*).mp.	461825
82	(comorbid* or multimorbid* or co-occurren* or co-morbid* or multi-morbid* or Multidisease* or multi-disease*).mp.	462635
83	exp Comorbidity/ or exp Multimorbidity/ or exp Multiple Chronic Conditions/	281021
84	79 or 80 or 81 or 82 or 83	895311
85	exp "systematic review"/	268161
86	"systematic review* ".m_titl.	163162
87	exp meta analysis/	200254
88	"meta-analys* ".m_titl.	144571
89	exp "systematic review"/ or "systematic review* ".m_titl. or exp meta analysis/ or "meta-analys* ".m_titl.	411674
90	32 or 52 or 73	16955591
91	(32 or 52 or 73) and 78	237676
92	(32 or 52 or 73) and 78 and 84	9231
93	(32 or 52 or 73) and 78 and 84 and 89	271
94	exp animal/ not exp human/	4710933

95	((32 or 52 or 73) and 78 and 84) not 94	9130
96	((32 or 52 or 73) and 78 and 84 and 89) not 94	271
97	((32 or 52 or 73) and 78 and 89) not 94	3315

PsycINFO (October 23rd 2020)

# ▲	Searches	Results
1	((Non-communicable or Noncommunicable or Non-infectious) adj (disease* or condition* or illness*)).mp.	1054
2	exp Chronic Illness/ or ((chronic or long-term) adj (disease* or condition* or illness*)).mp.	48942
3	exp Heart Disorders/ or (heart adj (disease* or disorder* or failure)).mp. or (cardiac adj (disease* or disorder* or failure)).mp.	22461
4	exp Cardiovascular Disorders/ or (cardiovascular adj (disease* or disorder* or failure)).mp.	68973
5	(coronary adj (disease* or disorder* or failure)).mp.	450
6	exp Cerebrovascular Disorders/ or (cerebrovascular adj (disease* or disorder* or insufficienc* or occlusion*)).mp. or (vascular adj (disease* or disorder*)).mp. or (carotid* adj (disease* or disorder*)).mp.	31536
7	(arter* adj (disease* or disorder*)).mp.	2605
8	(heart adj3 (malform* or defect* or congeni*)).mp.	1007
9	exp Thromboses/ or ((deep vein or deep venous) adj thrombos*).mp. or phlebothrombos*.mp.	1070
10	exp Embolisms/ or (pulmonar* adj (thromboembolism* or embolism* or disease* or disorder*)).mp.	3511
11	exp Cerebrovascular Accidents/ or stroke.mp.	36761
12	exp Neoplasms/ or Cancer*.mp. or neoplas*.mp. or tumor*.mp.	83903
13	exp Lung Disorders/ or exp Respiratory Tract Disorders/ or ((lung* or respiratory or pulmonar* or airflow or airway) adj2 (disease* or obstruct* or hypersensitiv*)).mp. or exp Asthma/ or asthma*.mp. or exp Chronic Obstructive Pulmonary Disease/	20498
14	exp Diabetes Mellitus/ or diabet*.mp.	33566
15	((autoimmun* or auto immun* or autoaggress* or auto aggress*) adj (disorder* or disease*)).mp.	2453
16	exp Metabolic Syndrome/ or ((metabolic or insulin resistance) adj (disorder* or disease* or syndrome*)).mp.	5856

17	exp Obesity/ or obes*.mp.	45201
18	exp Osteoporosis/ or osteoporo*.mp. or bone loss.mp. or osteolysis.mp. or bone resorption.mp.	2582
19	exp Parkinson's Disease/ or parkinson*.mp. or paralysis agitans.mp.	36257
20	exp Arthritis/ or arthriti*.mp. or polyarthriti*.mp. or rheumarthriti*.mp.	7176
21	exp Kidney Diseases/ or (kidney adj (disease* or disorder*)).mp.	2912
22	exp Liver Disorders/ or (liver adj (disease* or disorder* or dysfunction*)).mp.	5371
23	exp Hypertension/ or high blood pressure*.mp. or hypertens*.mp.	20357
24	(hyperlipem* or hyperlipidem* or lipem* or lipidem*).mp.	1314
25	(((high* or elevat*) adj cholesterol*) or hypercholesterem* or hypercholesterolem*).mp.	1637
26	hypertriglyceridem*.mp.	293
27	exp Thyroid Disorders/ or (thyroid adj (disease* or disorder*)).mp. or exp Hyperthyroidism/ or hyperthyroid*.mp. or exp Hypothyroidism/ or hypothyroid*.mp. or ((thyroid-stimulating hormone* or tsh) adj deficien*).mp.	2924
28	exp Nervous System Disorders/ or motor neuron* disease*.mp. or lateral scleros*.mp. or motor system disease*.mp.	319159
29	exp Multiple Sclerosis/ or multiple sclerosis.mp. or disseminated sclerosis.mp.	16491
30	exp Pulmonary Emphysema/ or emphysema*.mp.	285
31	exp Bronchial Disorders/ or bronchit*.mp.	476
32	1 or 2 or 3 or 4 or 5 or 6 or 7 or 8 or 9 or 10 or 11 or 12 or 13 or 14 or 15 or 16 or 17 or 18 or 19 or 20 or 21 or 22 or 23 or 24 or 25 or 26 or 27 or 28 or 29 or 30 or 31	567022
33	exp Mental Disorders/ or exp Psychosis/ or ((mental* or psychiatr* or psycho*) adj (disorder* or disease* or illness*)).mp.	921435
34	exp Major Depression/ or exp "Depression (Emotion)"/ or Depress*.mp. or MDD.mp.	374034
35	exp Anxiety Disorders/ or exp Anxiety/ or anxi*.mp.	273241

36	exp Phobias/ or phobi*.mp.	24242
37	exp Schizophrenia/ or schizophreni*.mp. or hebephreni*.mp.	140371
38	exp Somatoform Disorders/ or ((somatoform* or somati* or medically unexplained or briquet or pain) adj (disorder* or syndrome* or symptom*)).mp.	27313
39	exp Dissociative Disorders/ or (dissociative adj (disorder* or hysteri* or reaction*)).mp. or dissociation*.mp.	24759
40	exp Hysteria/ or hysteri*.mp.	8253
41	exp Affective Disorders/ or ((affective* or mood*) adj (disorder* or disease* or illness* or symptom*)).mp.	170875
42	exp Posttraumatic Stress Disorder/ or PTSD.mp. or ((post trauma* or posttrauma*) adj (stress* or neurose*)).mp. or combat disorder*.mp. or war disorder*.mp.	51508
43	exp Cognitive Impairment/ or ((cognitive or cognition or mental or neurocognitive) adj (dysfunction* or decline* or impairment* or deterioration* or disorder* or illness* or disease*)).mp.	221483
44	exp Personality Disorders/ or personality disorder*.mp.	49598
45	exp Impulse Control Disorders/ or impulse control disorder*.mp. or intermittent explosive disorder*.mp.	2232
46	exp Eating Disorders/ or ((eating or appetite or feeding) adj disorder*).mp.	39055
47	exp Bipolar Disorder/ or ((bipolar or mani*) adj (disorder* or illness* or disease*)).mp.	38332
48	exp Obsessive Compulsive Disorder/ or OCD*.mp. or ((obsess*-compulsi* or obsess* or compulsi*) adj (disorder* or illness* or disease* or neuros*)).mp.	21451
49	exp Panic Disorder/ or (panic adj (attack* or disorder*)).mp.	13643
50	exp Agoraphobia/ or agoraphobi*.mp.	6095
51	exp Neurosis/ or neuros*.mp. or neurotic disorder*.mp. or psychoneuros*.mp.	81072
52	33 or 34 or 35 or 36 or 37 or 38 or 39 or 40 or 41 or 42 or 43 or 44 or 45 or 46 or 47 or 48 or 49 or 50 or 51	1284709

53	exp Infectious Disorders/ or ((communic* or contag* or transmi* or infect*) adj (disease* or infection* or illness*)).mp.	70725
54	exp Bacterial Disorders/ or bacteri* infection*.mp.	2731
55	exp Eye Disorders/ or conjunctivitis.mp.	4950
56	exp HIV/ or hiv.mp. or Human immuno deficiency virus.mp.	57416
57	exp AIDS/ or AIDS.mp. or immunodeficiency associated virus.mp. or immun* deficiency associated virus.mp. or acquired immunodeficiency syndrome*.mp. or acquired immun* deficiency syndrome*.mp.	48739
58	(Bairnsdale or Buruli).mp.	7
59	onchocer*.mp.	47
60	hepatitis.mp. or exp Hepatitis/	4736
61	leishmania*.mp.	61
62	(lepros* or hansen*).mp.	1155
63	(elephantias* or filaria*).mp.	75
64	(egyptian ophthalmia* or trachoma*).mp.	309
65	(chickungunya or chikungunya).mp.	39
66	taenia*.mp.	160
67	cysticercos*.mp.	102
68	(hydatid* or echinococc*).mp.	39
69	(trypanosom* or chagas).mp.	217
70	sleeping sickness.mp.	50
71	(japanese adj3 encephalitis).mp.	73
72	exp Syphilis/ or syphilis.mp.	1870

73	53 or 54 or 55 or 56 or 57 or 58 or 59 or 60 or 61 or 62 or 63 or 64 or 65 or 66 or 67 or 68 or 69 or 70 or 71 or 72	108493
74	exp Tuberculosis/	1223
75	Tuberculos*.mp.	2795
76	TB.mp.	1354
77	koch*.mp.	1045
78	exp Tuberculosis/ or Tuberculos*.mp. or TB.mp. or koch*.mp.	4450
79	(multiple adj (ill* or disease* or condition* or syndrom* or disorder*)).mp.	913
80	((Cooccur* or co-occur* or coexist* or co-exist* or multipl* or concord* or discord* or long-term or physical*) adj3 (disease* or ill* or care or condition* or disorder* or health* or medication* or symptom* or syndrom*)).mp.	116177
81	(comorbid* or multimorbid* or co-occurren* or co-morbid* or multi-morbid* or Multidisease* or multi-disease*).mp.	88147
82	exp Comorbidity/	33088
83	79 or 80 or 81 or 82	192590
84	exp "Systematic Review"/	460
85	"systematic review*".m_titl.	21652
86	exp Meta Analysis/	4825
87	"meta-analys*".m_titl.	18645
88	exp "Systematic Review"/ or "systematic review*".m_titl. or exp Meta Analysis/ or "meta-analys*".m_titl.	37279
89	32 or 52 or 73	1665397
90	(32 or 52 or 73) and 78	3046
91	(32 or 52 or 73) and 78 and 83	362

92	(32 or 52 or 73) and 78 and 83 and 88	6
93	(32 or 52 or 73) and 78 and 88	27

Web of Science (October 23rd 2020)

# 90	**1,039**	(#74 OR #52 OR #32) AND #82 AND #85<br>*Indexes=SCI-EXPANDED, SSCI, A&HCI, CPCI-S, CPCI-SSH, ESCI Timespan=All years*
# 89	**62**	(#74 OR #52 OR #32) AND #82 AND #78 AND #85<br>*Indexes=SCI-EXPANDED, SSCI, A&HCI, CPCI-S, CPCI-SSH, ESCI Timespan=All years*
# 88	**3,336**	(#74 OR #52 OR #32) AND #82 AND #78<br>*Indexes=SCI-EXPANDED, SSCI, A&HCI, CPCI-S, CPCI-SSH, ESCI Timespan=All years*
# 87	**87,846**	(#74 OR #52 OR #32) AND #82<br>*Indexes=SCI-EXPANDED, SSCI, A&HCI, CPCI-S, CPCI-SSH, ESCI Timespan=All years*
# 86	**11,086,931**	#74 OR #52 OR #32<br>*Indexes=SCI-EXPANDED, SSCI, A&HCI, CPCI-S, CPCI-SSH, ESCI Timespan=All years*
# 85	**226,528**	#84 OR #83<br>*Indexes=SCI-EXPANDED, SSCI, A&HCI, CPCI-S, CPCI-SSH, ESCI Timespan=All years*
# 84	**134,872**	TI="Meta-analysis"<br>*Indexes=SCI-EXPANDED, SSCI, A&HCI, CPCI-S, CPCI-SSH, ESCI Timespan=All years*
# 83	**147,319**	TI = "Systematic Review"<br>*Indexes=SCI-EXPANDED, SSCI, A&HCI, CPCI-S, CPCI-SSH, ESCI Timespan=All years*
# 82	**234,479**	#81 OR #80 OR #79<br>*Indexes=SCI-EXPANDED, SSCI, A&HCI, CPCI-S, CPCI-SSH, ESCI Timespan=All years*
# 81	**19,329**	TS=koch*<br>*Indexes=SCI-EXPANDED, SSCI, A&HCI, CPCI-S, CPCI-SSH, ESCI Timespan=All years*
# 80	**80,779**	TS=TB<br>*Indexes=SCI-EXPANDED, SSCI, A&HCI, CPCI-S, CPCI-SSH, ESCI Timespan=All years*

#79	170,591	TS=Tuberculos* *Indexes=SCI-EXPANDED, SSCI, A&HCI, CPCI-S, CPCI-SSH, ESCI Timespan=All years*
#78	659,174	#77 OR #76 OR #75 *Indexes=SCI-EXPANDED, SSCI, A&HCI, CPCI-S, CPCI-SSH, ESCI Timespan=All years*
#77	238,429	TS=(comorbid* or multimorbid* or co-occurren* or co-morbid* or multi-morbid* or Multidisease* or multi-disease*) *Indexes=SCI-EXPANDED, SSCI, A&HCI, CPCI-S, CPCI-SSH, ESCI Timespan=All years*
#76	325,588	TS=((Cooccur* or co-occur* or coexist* or co-exist* or multipl* or concord* or discord* or long-term or physical*) NEAR/3 (disease* or ill* or care or condition* or disorder* or health* or medication* or symptom* or syndrom*)) *Indexes=SCI-EXPANDED, SSCI, A&HCI, CPCI-S, CPCI-SSH, ESCI Timespan=All years*
#75	191,473	TS=(multiple NEAR (ill* or disease* or condition* or syndrom* or disorder*)) *Indexes=SCI-EXPANDED, SSCI, A&HCI, CPCI-S, CPCI-SSH, ESCI Timespan=All years*
#74	2,677,956	#73 OR #72 OR #71 OR #70 OR #69 OR #68 OR #67 OR #66 OR #65 OR #64 OR #63 OR #62 OR #61 OR #60 OR #59 OR #58 OR #57 OR #56 OR #55 OR #54 OR #53 *Indexes=SCI-EXPANDED, SSCI, A&HCI, CPCI-S, CPCI-SSH, ESCI Timespan=All years*
#73	23,389	TS=syphilis *Indexes=SCI-EXPANDED, SSCI, A&HCI, CPCI-S, CPCI-SSH, ESCI Timespan=All years*
#72	5,981	TS=(japanese NEAR/3 encephalitis) *Indexes=SCI-EXPANDED, SSCI, A&HCI, CPCI-S, CPCI-SSH, ESCI Timespan=All years*
#71	4,257	TS=sleeping sickness *Indexes=SCI-EXPANDED, SSCI, A&HCI, CPCI-S, CPCI-SSH, ESCI Timespan=All years*
#70	50,013	TS=trypanosom* or TS=chagas *Indexes=SCI-EXPANDED, SSCI, A&HCI, CPCI-S, CPCI-SSH, ESCI Timespan=All years*
#69	19,785	TS=hydatid* OR TS=echinococc* *Indexes=SCI-EXPANDED, SSCI, A&HCI, CPCI-S, CPCI-SSH, ESCI Timespan=All years*

#68	4,657	TS=cysticercos*<br>*Indexes=SCI-EXPANDED, SSCI, A&HCI, CPCI-S, CPCI-SSH, ESCI Timespan=All years*
#67	8,257	TS=taenia*<br>*Indexes=SCI-EXPANDED, SSCI, A&HCI, CPCI-S, CPCI-SSH, ESCI Timespan=All years*
#66	6,629	TS=chickungunya Or TS=chikungunya<br>*Indexes=SCI-EXPANDED, SSCI, A&HCI, CPCI-S, CPCI-SSH, ESCI Timespan=All years*
#65	19,987	TS=trachoma*<br>*Indexes=SCI-EXPANDED, SSCI, A&HCI, CPCI-S, CPCI-SSH, ESCI Timespan=All years*
#64	5	TS=egyptian ophthalmia*<br>*Indexes=SCI-EXPANDED, SSCI, A&HCI, CPCI-S, CPCI-SSH, ESCI Timespan=All years*
#63	11,257	TS=elephantias* OR TS=filaria*<br>*Indexes=SCI-EXPANDED, SSCI, A&HCI, CPCI-S, CPCI-SSH, ESCI Timespan=All years*
#62	30,101	TS=lepros* OR TS=hansen*<br>*Indexes=SCI-EXPANDED, SSCI, A&HCI, CPCI-S, CPCI-SSH, ESCI Timespan=All years*
#61	41,800	TS=leishmania*<br>*Indexes=SCI-EXPANDED, SSCI, A&HCI, CPCI-S, CPCI-SSH, ESCI Timespan=All years*
#60	267,930	TS=hepatitis<br>*Indexes=SCI-EXPANDED, SSCI, A&HCI, CPCI-S, CPCI-SSH, ESCI Timespan=All years*
#59	6,453	TS=onchocer*<br>*Indexes=SCI-EXPANDED, SSCI, A&HCI, CPCI-S, CPCI-SSH, ESCI Timespan=All years*
#58	1,120	TS=Bairnsdale OR TS=Buruli<br>*Indexes=SCI-EXPANDED, SSCI, A&HCI, CPCI-S, CPCI-SSH, ESCI Timespan=All years*
#57	640,388	TS=AIDS OR TS=immunodeficiency associated virus OR TS=immun* deficiency associated virus OR TS=acquired immunodeficiency syndrome* OR TS=acquired immun* deficiency syndrome*

		Indexes=SCI-EXPANDED, SSCI, A&HCI, CPCI-S, CPCI-SSH, ESCI Timespan=All years
#56	**374,418**	TS=hiv OR TS=Human immuno deficiency virus<br>*Indexes=SCI-EXPANDED, SSCI, A&HCI, CPCI-S, CPCI-SSH, ESCI Timespan=All years*
#55	**10,545**	TS=conjunctivitis<br>*Indexes=SCI-EXPANDED, SSCI, A&HCI, CPCI-S, CPCI-SSH, ESCI Timespan=All years*
#54	**240,625**	TS=bacteri* infection*<br>*Indexes=SCI-EXPANDED, SSCI, A&HCI, CPCI-S, CPCI-SSH, ESCI Timespan=All years*
#53	**1,710,573**	TS=((communic* or contag* or transmi* or infect*) NEAR (disease* or infection* or illness*))<br>*Indexes=SCI-EXPANDED, SSCI, A&HCI, CPCI-S, CPCI-SSH, ESCI Timespan=All years*
#52	**1,891,336**	#51 OR #50 OR #49 OR #48 OR #47 OR #46 OR #45 OR #44 OR #43 OR #42 OR #41 OR #40 OR #39 OR #38 OR #37 OR #36 OR #35 OR #34 OR #33<br>*Indexes=SCI-EXPANDED, SSCI, A&HCI, CPCI-S, CPCI-SSH, ESCI Timespan=All years*
#51	**155,801**	TS=neuros* OR TS=neurotic disorder* OR TS=psychoneuros*<br>*Indexes=SCI-EXPANDED, SSCI, A&HCI, CPCI-S, CPCI-SSH, ESCI Timespan=All years*
#50	**5,165**	TS=agoraphobi*<br>*Indexes=SCI-EXPANDED, SSCI, A&HCI, CPCI-S, CPCI-SSH, ESCI Timespan=All years*
#49	**19,459**	TS=(panic NEAR (attack* or disorder*))<br>*Indexes=SCI-EXPANDED, SSCI, A&HCI, CPCI-S, CPCI-SSH, ESCI Timespan=All years*
#48	**32,551**	TS=OCD* OR TS=((obsess*-compulsi* or obsess* or compulsi*) NEAR (disorder* or illness* or disease* or neuros*))<br>*Indexes=SCI-EXPANDED, SSCI, A&HCI, CPCI-S, CPCI-SSH, ESCI Timespan=All years*
#47	**137,039**	TS=((bipolar or mani*) NEAR (disorder* or illness* or disease*))<br>*Indexes=SCI-EXPANDED, SSCI, A&HCI, CPCI-S, CPCI-SSH, ESCI Timespan=All years*
#46	**35,573**	TS=((eating or appetite or feeding) NEAR disorder*)<br>*Indexes=SCI-EXPANDED, SSCI, A&HCI, CPCI-S, CPCI-SSH, ESCI Timespan=All years*

#45	**4,535**	TS=impulse control disorder* OR TS=intermittent explosive disorder*<br>*Indexes=SCI-EXPANDED, SSCI, A&HCI, CPCI-S, CPCI-SSH, ESCI Timespan=All years*
#44	**52,384**	TS=personality disorder*<br>*Indexes=SCI-EXPANDED, SSCI, A&HCI, CPCI-S, CPCI-SSH, ESCI Timespan=All years*
#43	**325,105**	TS=((cognitive or cognition or mental or neurocognitive) NEAR (dysfunction* or decline* or impairment* or deterioration* or disorder* or illness* or disease*))<br>*Indexes=SCI-EXPANDED, SSCI, A&HCI, CPCI-S, CPCI-SSH, ESCI Timespan=All years*
#42	**69,002**	TS=PTSD OR TS=(((post NEAR trauma*) or posttrauma*) NEAR (stress* or neurose*)) OR TS=combat disorder* OR TS=war disorder*<br>*Indexes=SCI-EXPANDED, SSCI, A&HCI, CPCI-S, CPCI-SSH, ESCI Timespan=All years*
#41	**73,280**	TS=((affective* or mood*) NEAR (disorder* or disease* or illness* or symptom*))<br>*Indexes=SCI-EXPANDED, SSCI, A&HCI, CPCI-S, CPCI-SSH, ESCI Timespan=All years*
#40	**6,468**	TS=hysteri*<br>*Indexes=SCI-EXPANDED, SSCI, A&HCI, CPCI-S, CPCI-SSH, ESCI Timespan=All years*
#39	**215,870**	TS=(dissociative NEAR (disorder* or hysteri* or reaction*)) OR TS=dissociation*<br>*Indexes=SCI-EXPANDED, SSCI, A&HCI, CPCI-S, CPCI-SSH, ESCI Timespan=All years*
#38	**122,031**	TS=((somatoform* or somati* or (medically NEAR unexplained) or briquet or pain) NEAR (disorder* or syndrome* or symptom*))<br>*Indexes=SCI-EXPANDED, SSCI, A&HCI, CPCI-S, CPCI-SSH, ESCI Timespan=All years*
#37	**200,099**	TS=schizophreni* OR TS=hebephreni*<br>*Indexes=SCI-EXPANDED, SSCI, A&HCI, CPCI-S, CPCI-SSH, ESCI Timespan=All years*
#36	**19,359**	TS=phobi*<br>*Indexes=SCI-EXPANDED, SSCI, A&HCI, CPCI-S, CPCI-SSH, ESCI Timespan=All years*
#35	**300,816**	TS=anxi*<br>*Indexes=SCI-EXPANDED, SSCI, A&HCI, CPCI-S, CPCI-SSH, ESCI Timespan=All years*
#34	**645,434**	TS=Depress* OR TS=MDD<br>*Indexes=SCI-EXPANDED, SSCI, A&HCI, CPCI-S, CPCI-SSH, ESCI Timespan=All years*

# 33	**283,212**	TS=((mental* or psychiatr* or psycho*) NEAR (disorder* or disease* or illness*))<br>*Indexes=SCI-EXPANDED, SSCI, A&HCI, CPCI-S, CPCI-SSH, ESCI Timespan=All years*
# 32	**7,479,780**	#31 OR #30 OR #29 OR #28 OR #27 OR #26 OR #25 OR #24 OR #23 OR #22 OR #21 OR #20 OR #19 OR #18 OR #17 OR #16 OR #15 OR #14 OR #13 OR #12 OR #11 OR #10 OR #9 OR #8 OR #7 OR #6 OR #5 OR #4 OR #3 OR #2 OR #1<br>*Indexes=SCI-EXPANDED, SSCI, A&HCI, CPCI-S, CPCI-SSH, ESCI Timespan=All years*
# 31	**20,591**	TS=bronchit*<br>*Indexes=SCI-EXPANDED, SSCI, A&HCI, CPCI-S, CPCI-SSH, ESCI Timespan=All years*
# 30	**26,727**	TS=emphysema*<br>*Indexes=SCI-EXPANDED, SSCI, A&HCI, CPCI-S, CPCI-SSH, ESCI Timespan=All years*
# 29	**129,081**	TS=multiple sclerosis OR TS=disseminated sclerosis<br>*Indexes=SCI-EXPANDED, SSCI, A&HCI, CPCI-S, CPCI-SSH, ESCI Timespan=All years*
# 28	**67,922**	TS=motor neuron* disease* OR TS=lateral scleros* OR TS=motor system disease*<br>*Indexes=SCI-EXPANDED, SSCI, A&HCI, CPCI-S, CPCI-SSH, ESCI Timespan=All years*
# 27	**71,105**	TS=(thyroid NEAR (disease* or disorder*)) OR TS=hyperthyroid* OR TS=hypothyroid* OR TS=(((thyroid-stimulating NEAR hormone*) or tsh) NEAR deficien*)<br>*Indexes=SCI-EXPANDED, SSCI, A&HCI, CPCI-S, CPCI-SSH, ESCI Timespan=All years*
# 26	**14,111**	TS=hypertriglyceridem*<br>*Indexes=SCI-EXPANDED, SSCI, A&HCI, CPCI-S, CPCI-SSH, ESCI Timespan=All years*
# 25	**113,697**	TS=((high* or elevat*) NEAR cholesterol*) Or TS=hypercholesterem* OR TS=hypercholesterolem*<br>*Indexes=SCI-EXPANDED, SSCI, A&HCI, CPCI-S, CPCI-SSH, ESCI Timespan=All years*
# 24	**36,981**	TS=hyperlipem* OR TS=hyperlipidem* or TS=lipem* OR TS=lipidem*<br>*Indexes=SCI-EXPANDED, SSCI, A&HCI, CPCI-S, CPCI-SSH, ESCI Timespan=All years*
# 23	**613,541**	TS=high blood pressure* OR TS=hypertens*<br>*Indexes=SCI-EXPANDED, SSCI, A&HCI, CPCI-S, CPCI-SSH, ESCI Timespan=All years*

#22	161,103	TS=(liver NEAR (disease* or disorder* or dysfunction*))<br>*Indexes=SCI-EXPANDED, SSCI, A&HCI, CPCI-S, CPCI-SSH, ESCI Timespan=All years*
#21	125,492	TS=(kidney NEAR (disease* or disorder*))<br>*Indexes=SCI-EXPANDED, SSCI, A&HCI, CPCI-S, CPCI-SSH, ESCI Timespan=All years*
#20	278,897	TS=arthriti* OR TS=polyarthriti* OR TS=rheumarthriti*<br>*Indexes=SCI-EXPANDED, SSCI, A&HCI, CPCI-S, CPCI-SSH, ESCI Timespan=All years*
#19	181,735	TS=parkinson* OR TS=paralysis agitans<br>*Indexes=SCI-EXPANDED, SSCI, A&HCI, CPCI-S, CPCI-SSH, ESCI Timespan=All years*
#18	195,021	TS=osteoporo* OR TS=bone loss OR TS=osteolysis or TS=bone resorption<br>*Indexes=SCI-EXPANDED, SSCI, A&HCI, CPCI-S, CPCI-SSH, ESCI Timespan=All years*
#17	426,882	TS=obes*<br>*Indexes=SCI-EXPANDED, SSCI, A&HCI, CPCI-S, CPCI-SSH, ESCI Timespan=All years*
#16	174,058	TS=((metabolic or (insulin near resistance)) NEAR (disorder* or disease* or syndrome*))<br>*Indexes=SCI-EXPANDED, SSCI, A&HCI, CPCI-S, CPCI-SSH, ESCI Timespan=All years*
#15	109,133	TS=((autoimmun* or (auto NEAR immun*) or autoaggress* or (auto NEAR aggress*)) NEAR (disorder* or disease*))<br>*Indexes=SCI-EXPANDED, SSCI, A&HCI, CPCI-S, CPCI-SSH, ESCI Timespan=All years*
#14	771,913	TS=diabet*<br>*Indexes=SCI-EXPANDED, SSCI, A&HCI, CPCI-S, CPCI-SSH, ESCI Timespan=All years*
#13	385,258	TS=((lung* or respiratory or pulmonar* or airflow or airway) NEAR/2 (disease* or obstruct* or hypersensitiv*)) OR TS=asthma*<br>*Indexes=SCI-EXPANDED, SSCI, A&HCI, CPCI-S, CPCI-SSH, ESCI Timespan=All years*
#12	3,520,631	TS=Cancer* or TS=neoplas* OR TS=tumor*<br>*Indexes=SCI-EXPANDED, SSCI, A&HCI, CPCI-S, CPCI-SSH, ESCI Timespan=All years*
#11	356,775	TS=stroke<br>*Indexes=SCI-EXPANDED, SSCI, A&HCI, CPCI-S, CPCI-SSH, ESCI Timespan=All years*

# 10	164,438	TS=(pulmonar* NEAR (thromboembolism* or embolism* or disease* or disorder*)) *Indexes=SCI-EXPANDED, SSCI, A&HCI, CPCI-S, CPCI-SSH, ESCI Timespan=All years*
# 9	34,725	TS=(("deep vein" or "deep venous") NEAR thrombos*) OR TS=phlebothrombos* *Indexes=SCI-EXPANDED, SSCI, A&HCI, CPCI-S, CPCI-SSH, ESCI Timespan=All years*
# 8	52,910	TS=(heart NEAR/3 (malform* or defect* or congeni*)) *Indexes=SCI-EXPANDED, SSCI, A&HCI, CPCI-S, CPCI-SSH, ESCI Timespan=All years*
# 7	223,717	TS=(arter* NEAR (disease* or disorder*)) *Indexes=SCI-EXPANDED, SSCI, A&HCI, CPCI-S, CPCI-SSH, ESCI Timespan=All years*
# 6	131,935	TS=(cerebrovascular NEAR (disease* or disorder* or insufficienc* or occlusion*)) OR TS=(vascular NEAR (disease* or disorder*)) OR TS=(carotid* NEAR (disease* or disorder*)) *Indexes=SCI-EXPANDED, SSCI, A&HCI, CPCI-S, CPCI-SSH, ESCI Timespan=All years*
# 5	260,403	TS=(coronary NEAR (disease* or disorder* or failure)) *Indexes=SCI-EXPANDED, SSCI, A&HCI, CPCI-S, CPCI-SSH, ESCI Timespan=All years*
# 4	286,220	TS=(cardiovascular NEAR (disease* or disorder* or failure)) *Indexes=SCI-EXPANDED, SSCI, A&HCI, CPCI-S, CPCI-SSH, ESCI Timespan=All years*
# 3	561,756	TS=(heart NEAR (disease* or disorder* or failure)) OR TS=(cardiac NEAR (disease* or disorder* or failure)) *Indexes=SCI-EXPANDED, SSCI, A&HCI, CPCI-S, CPCI-SSH, ESCI Timespan=All years*
# 2	500,606	TS=((chronic or long-term) NEAR (disease* or condition* or illness*)) *Indexes=SCI-EXPANDED, SSCI, A&HCI, CPCI-S, CPCI-SSH, ESCI Timespan=All years*
# 1	12,403	TS=((Non-communicable or Noncommunicable or Non-infectious) NEAR/1 (disease* or condition* or illness*)) *Indexes=SCI-EXPANDED, SSCI, A&HCI, CPCI-S, CPCI-SSH, ESCI Timespan=All years*

Global Index Medicus (October 23rd 2020)

(((("Noncommunicable Diseases" OR ((("Non-communicable" or Noncommunicable or "Non-infectious") AND (disease* or condition* or illness*)))) OR ("Chronic Disease" OR (((chronic or "long-term") AND (disease* or condition* or illness*)))) OR ("Heart Diseases" OR ((heart AND (disease* or disorder* or failure)) or (cardiac AND (disease* or disorder* or failure)))) OR ("Cardiovascular Diseases" OR ((cardiovascular AND (disease* or disorder* or failure)))) OR ("Coronary Disease" OR (coronary AND (disease* or disorder* or failure))) OR ("Cerebrovascular Disorders" OR ((cerebrovascular AND (disease* or disorder* or insufficienc* or occlusion*)) or (vascular AND (disease* or disorder*)) or (carotid* AND (disease* or disorder*)))) OR ("Peripheral Arterial Disease" OR ((arter* AND (disease* or disorder*)))) OR ("Rheumatic Heart Disease" OR "Heart Defects, Congenital" OR ((heart AND (malform* or defect* or congeni*)))) OR ("Venous Thrombosis" OR ((("deep vein" or "deep venous") AND thrombos*) or phlebothrombos*)) OR ("Pulmonary Embolism" OR ((pulmonar* AND (thromboembolism* or embolism* or disease* or disorder*)))) OR ("Stroke" OR (stroke)) OR ("Neoplasms" OR (Cancer* or neoplas* or tumor*)) OR ("Lung Diseases" OR "Respiratory Tract Diseases" OR "Lung Diseases, Obstructive" OR (((lung* or respiratory or pulmonar* or airflow or airway) AND (disease* or obstruct* or hypersensitiv*))) OR "Asthma" OR asthma* OR "Pulmonary Disease, Chronic Obstructive" OR "Respiratory Hypersensitivity") OR ("Diabetes Mellitus" or diabet*) OR ("Autoimmune Diseases" OR ((autoimmun* or (auto AND immun*) or autoaggress* or (auto AND aggress*)) AND (disorder* or disease*))) OR ("Metabolic Syndrome" OR "Metabolic Diseases" OR (((metabolic or "insulin resistance") AND (disorder* or disease* or syndrome*)))) OR ("Obesity" OR obes*) OR ("Osteoporosis" OR (osteoporo* or "bone loss" or "Osteolysis" or osteolysis or "bone resorption")) OR ("Parkinson Disease" OR (parkinson* or "paralysis agitans")) OR ("Arthritis" OR (arthriti* or polyarthriti* or rheumarthriti*)) OR ("Kidney Diseases" OR ((kidney AND (disease* or disorder*)))) OR ("Liver Diseases" OR (liver AND (disease* or disorder* or dysfunction*))) OR ("Hypertension" OR ("high blood pressure" or hypertens*)) OR ("Hyperlipidemias" OR (hyperlipem* or hyperlipidem* or lipem* or lipidem*)) OR ("Hypercholesterolemia" OR (((high* or elevat*) AND cholesterol*) or hypercholesterem* or hypercholesterolem*)) OR ("Hypertriglyceridemia " OR (hypertriglyceridem*)) OR ("Thyroid Diseases" OR (thyroid AND (disease* or disorder*)) OR "Hyperthyroidism " OR (hyperthyroid*) OR "Hypothyroidism" OR (hypothyroid*) OR ((("thyroid-stimulating hormone" or tsh) AND deficien*))) OR ("Motor Neuron Disease" OR ("motor neuron disease") OR (lateral AND scleros*) OR ("motor system disease")) OR ("Multiple Sclerosis" OR ("multiple sclerosis" or "disseminated sclerosis")) OR ("Emphysema" OR emphysema*) OR ("Bronchitis" OR bronchit*)) OR (("Mental Disorders" OR "Psychotic Disorders" OR (((mental* or psychiatr* or psycho*) AND (disorder* or disease* or illness*)))) OR ("Depressive Disorder, Major" OR "Depression" OR (Depress* or MDD)) OR ("Anxiety Disorders" OR "Anxiety" OR anxi*) OR ("Phobic Disorders" OR phobi*) OR ("Schizophrenia" Or (schizophreni* or hebephreni*)) OR ("Somatoform Disorders" OR "Medically Unexplained Symptoms" OR (((somatoform* or somati* or (medically AND unexplained) or briquet or pain) AND (disorder* or syndrome* or symptom*)))) OR ("Dissociative Disorders" OR (dissociative AND (disorder* or hysteri* or reaction*)) or dissociation*) OR ("Hysteria" or hysteri*) OR ("Mood Disorders" OR ((affective* or mood*) AND (disorder* or disease* or illness* or symptom*))) OR ("Stress Disorders, Post-Traumatic" OR (PTSD or ((posttrauma* or trauma*) AND (stress* or neurose*)) or "combat disorder" or "war disorder")) OR ("Cognition Disorders" OR (((cognitive or cognition or mental or neurocognitive) AND (dysfunction* or decline* or impairment* or deterioration* or disorder* or illness* or disease*)))) OR ("Personality Disorders" OR "personality disorder") OR ("Disruptive, Impulse Control, and Conduct Disorders" OR ("impulse control disorder" or "intermittent explosive disorder")) OR ("Feeding and Eating Disorders" OR ((eating or appetite or feeding) AND disorder*)) OR ("Bipolar Disorder" OR ((bipolar or mani*) AND (disorder* or illness* or disease*))) OR ("Obsessive-Compulsive Disorder" OR OCD* or ((obsess* or compulsi*) AND (disorder* or illness* or disease*

or neuros*))) OR ("Panic Disorder" OR ((panic AND (attack* or disorder*)))) OR ("Agoraphobia" OR agoraphobi*) OR ("Neurotic Disorders" OR (neuros* or "neurotic disorder" or psychoneuros*))) OR (("Communicable Diseases" OR (((communic* or contag* or transmi* or infect*) AND (disease* or infection* or illness*)))) OR ("Bacterial Infections" OR (bacteri* infection*)) OR ("Conjunctivitis") OR ("HIV" OR "Human immuno deficiency virus") OR ("Acquired Immunodeficiency Syndrome" OR (AIDS or "immunodeficiency associated virus" or (immun* deficiency associated virus) or (acquired immunodeficiency syndrome*) or (acquired immun* deficiency syndrome*))) OR ("Buruli Ulcer" OR (Bairnsdale or Buruli)) OR ("Onchocerciasis" OR (onchocer*)) OR ("Hepatitis B" OR "Hepatitis C" OR (hepatitis)) OR ("Leishmaniasis" OR (leishmania*)) OR ("Leprosy" OR (lepros* or hansen*)) OR ("Elephantiasis, Filarial" OR (elephantias* or filaria*)) OR ("Trachoma" OR ((egyptian ophthalmia*) or trachoma*)) OR ("Chikungunya Fever" OR (chickungunya or chikungunya)) OR ("Taeniasis" OR taenia*) OR ("Cysticercosis" OR cysticercos*) OR ("Echinococcosis" OR (hydatid or echinococc*)) OR ("Chagas Disease" OR (trypanosom* or chagas)) OR ("Trypanosomiasis" OR ("sleeping sickness")) OR ("Encephalitis, Japanese" OR (japanese encephalitis)) OR ("Syphilis"))) AND ("Tuberculosis" OR Tuberculos* OR TB OR koch*)) AND ("Systematic Review" OR (systematic AND review*) OR "Meta-Analysis" OR meta-analys*)
131

OpenGrey (23/10/2020)

(((("Noncommunicable Diseases" OR ((("Non-communicable" or Noncommunicable or "Non-infectious") AND (disease* or condition* or illness*)))) OR ("Chronic Disease" OR (((chronic or "long-term") AND (disease* or condition* or illness*)))) OR ("Heart Diseases" OR ((heart AND (disease* or disorder* or failure)) or (cardiac AND (disease* or disorder* or failure)))) OR ("Cardiovascular Diseases" OR ((cardiovascular AND (disease* or disorder* or failure)))) OR ("Coronary Disease" OR (coronary AND (disease* or disorder* or failure))) OR ("Cerebrovascular Disorders" OR ((cerebrovascular AND (disease* or disorder* or insufficienc* or occlusion*)) or (vascular AND (disease* or disorder*)) or (carotid* AND (disease* or disorder*)))) OR ("Peripheral Arterial Disease" OR ((arter* AND (disease* or disorder*)))) OR ("Rheumatic Heart Disease" OR "Heart Defects, Congenital" OR ((heart AND (malform* or defect* or congeni*)))) OR ("Venous Thrombosis" OR ((("deep vein" or "deep venous") AND thrombos*) or phlebothrombos*)) OR ("Pulmonary Embolism" OR ((pulmonar* AND (thromboembolism* or embolism* or disease* or disorder*)))) OR ("Stroke" OR (stroke)) OR ("Neoplasms" OR (Cancer* or neoplas* or tumor*)) OR ("Lung Diseases" OR "Respiratory Tract Diseases" OR "Lung Diseases, Obstructive" OR (((lung* or respiratory or pulmonar* or airflow or airway) AND (disease* or obstruct* or hypersensitiv*))) OR "Asthma" OR asthma* OR "Pulmonary Disease, Chronic Obstructive" OR "Respiratory Hypersensitivity") OR ("Diabetes Mellitus" or diabet*) OR ("Autoimmune Diseases" OR ((autoimmun* or (auto AND immun*) or autoaggress* or (auto AND aggress*)) AND (disorder* or disease*))) OR ("Metabolic Syndrome" OR "Metabolic Diseases" OR (((metabolic or "insulin resistance") AND (disorder* or disease* or syndrome*)))) OR ("Obesity" OR obes*) OR ("Osteoporosis" OR (osteoporo* or "bone loss" or "Osteolysis" or osteolysis or "bone resorption")) OR ("Parkinson Disease" OR (parkinson* or "paralysis agitans")) OR ("Arthritis" OR (arthriti* or polyarthriti* or rheumarthriti*)) OR ("Kidney Diseases" OR ((kidney AND (disease* or disorder*)))) OR ("Liver Diseases" OR (liver AND (disease* or disorder* or dysfunction*))) OR ("Hypertension" OR ("high blood pressure" or hypertens*)) OR ("Hyperlipidemias" OR (hyperlipem* or hyperlipidem* or lipem* or lipidem*)) OR ("Hypercholesterolemia" OR (((high* or elevat*) AND cholesterol*) or hypercholesterem* or hypercholesterolem*)) OR ("Hypertriglyceridemia " OR (hypertriglyceridem*)) OR ("Thyroid Diseases" OR (thyroid AND (disease* or disorder*)) OR

"Hyperthyroidism " OR (hyperthyroid*) OR "Hypothyroidism" OR (hypothyroid*) OR ((("thyroid-stimulating hormone" or tsh) AND deficien*))) OR ("Motor Neuron Disease" OR ("motor neuron disease") OR (lateral AND scleros*) OR ("motor system disease")) OR ("Multiple Sclerosis" OR ("multiple sclerosis" or "disseminated sclerosis")) OR ("Emphysema" OR emphysema*) OR ("Bronchitis" OR bronchit*)) OR (("Mental Disorders" OR "Psychotic Disorders" OR (((mental* or psychiatr* or psycho*) AND (disorder* or disease* or illness*)))) OR ("Depressive Disorder, Major" OR "Depression" OR (Depress* or MDD)) OR ("Anxiety Disorders" OR "Anxiety" OR anxi*) OR ("Phobic Disorders" OR phobi*) OR ("Schizophrenia" Or (schizophreni* or hebephreni*)) OR ("Somatoform Disorders" OR "Medically Unexplained Symptoms" OR (((somatoform* or somati* or (medically AND unexplained) or briquet or pain) AND (disorder* or syndrome* or symptom*)))) OR ("Dissociative Disorders" OR (dissociative AND (disorder* or hysteri* or reaction*)) or dissociation*) OR ("Hysteria" or hysteri*) OR ("Mood Disorders" OR ((affective* or mood*) AND (disorder* or disease* or illness* or symptom*))) OR ("Stress Disorders, Post-Traumatic" OR (PTSD or ((posttrauma* or trauma*) AND (stress* or neurose*)) or "combat disorder" or "war disorder")) OR ("Cognition Disorders" OR (((cognitive or cognition or mental or neurocognitive) AND (dysfunction* or decline* or impairment* or deterioration* or disorder* or illness* or disease*)))) OR ("Personality Disorders" OR "personality disorder") OR ("Disruptive, Impulse Control, and Conduct Disorders" OR ("impulse control disorder" or "intermittent explosive disorder")) OR ("Feeding and Eating Disorders" OR ((eating or appetite or feeding) AND disorder*)) OR ("Bipolar Disorder" OR ((bipolar or mani*) AND (disorder* or illness* or disease*))) OR ("Obsessive-Compulsive Disorder" OR OCD* or ((obsess* or compulsi*) AND (disorder* or illness* or disease* or neuros*))) OR ("Panic Disorder" OR ((panic AND (attack* or disorder*)))) OR ("Agoraphobia" OR agoraphobi*) OR ("Neurotic Disorders" OR (neuros* or "neurotic disorder" or psychoneuros*))) OR (("Communicable Diseases" OR (((communic* or contag* or transmi* or infect*) AND (disease* or infection* or illness*)))) OR ("Bacterial Infections" OR (bacteri* infection*)) OR ("Conjunctivitis") OR ("HIV" OR "Human immuno deficiency virus") OR ("Acquired Immunodeficiency Syndrome" OR (AIDS or "immunodeficiency associated virus" or (immun* deficiency associated virus) or (acquired immunodeficiency syndrome*) or (acquired immun* deficiency syndrome*))) OR ("Buruli Ulcer" OR (Bairnsdale or Buruli)) OR ("Onchocerciasis" OR (onchocer*)) OR ("Hepatitis B" OR "Hepatitis C" OR (hepatitis)) OR ("Leishmaniasis" OR (leishmania*)) OR ("Leprosy" OR (lepros* or hansen*)) OR ("Elephantiasis, Filarial" OR (elephantias* or filaria*)) OR ("Trachoma" OR ((egyptian ophthalmia*) or trachoma*)) OR ("Chikungunya Fever" OR (chickungunya or chikungunya)) OR ("Taeniasis" OR taenia*) OR ("Cysticercosis" OR cysticercos*) OR ("Echinococcosis" OR (hydatid or echinococc*)) OR ("Chagas Disease" OR (trypanosom* or chagas)) OR ("Trypanosomiasis" OR ("sleeping sickness")) OR ("Encephalitis, Japanese" OR (japanese encephalitis)) OR ("Syphilis"))) AND ("Tuberculosis" OR Tuberculos* OR TB OR koch*)) AND ("Systematic Review" OR (systematic AND review*) OR "Meta-Analysis" OR meta-analys*)
3

PROSPERO (07/10/2020)

(((((Non-communicable or Noncommunicable or Non-infectious) AnD (disease* or condition* or illness*))) oR ((MeSH DESCRIPTOR Chronic Disease EXPLODE ALL TREES) or ((chronic or long-term) AnD (disease* or condition* or illness*))) oR ((MeSH DESCRIPTOR Heart Diseases EXPLODE ALL TREES) or ((heart AnD (disease* or disorder* or failure)) or (cardiac AnD (disease* or disorder* or failure)))) oR ((MeSH DESCRIPTOR Cardiovascular Diseases EXPLODE ALL TREES) OR ((cardiovascular AnD (disease* or disorder* or failure)))) oR ((MeSH DESCRIPTOR Coronary Disease EXPLODE ALL TREES) OR ((coronary AnD (disease* or disorder* or failure)))) oR ((MeSH DESCRIPTOR Cerebrovascular Disorders EXPLODE ALL TREES) OR ((cerebrovascular AnD (disease* or disorder* or insufficienc* or occlusion*))) OR ((vascular AnD (disease* or disorder*))) OR ((carotid* AnD (disease* or disorder*)))) oR ((MeSH DESCRIPTOR Peripheral Arterial Disease EXPLODE ALL TREES) OR ((arter* AnD (disease* or disorder*)))) oR ((MeSH DESCRIPTOR Rheumatic Heart Disease EXPLODE ALL TREES) OR (MeSH DESCRIPTOR Heart Defects, Congenital EXPLODE ALL TREES) or ((heart AnD (malform* or defect* or congeni*)))) oR ((MeSH DESCRIPTOR Venous Thrombosis EXPLODE ALL TREES) OR (((deep vein or deep venous) AnD thrombos*)) OR (phlebothrombos*)) oR ((MeSH DESCRIPTOR Pulmonary Embolism EXPLODE ALL TREES) OR ((pulmonar* AnD (thromboembolism* or embolism* or disease* or disorder*)))) oR ((MeSH DESCRIPTOR Stroke EXPLODE ALL TREES) OR (stroke)) oR ((MeSH DESCRIPTOR Neoplasms EXPLODE ALL TREES) OR (Cancer*) OR (neoplas*) OR (tumor*)) oR ((MeSH DESCRIPTOR Lung Diseases EXPLODE ALL TREES) OR (MeSH DESCRIPTOR Respiratory Tract Diseases EXPLODE ALL TREES) OR (MeSH DESCRIPTOR Lung Diseases, Obstructive EXPLODE ALL TREES) OR (((lung* or respiratory or pulmonar* or airflow or airway) AnD (disease* or obstruct* or hypersensitiv*))) OR (MeSH DESCRIPTOR Asthma EXPLODE ALL TREES) Or (asthma*) OR (MeSH DESCRIPTOR Pulmonary Disease, Chronic Obstructive EXPLODE ALL TREES) OR (MeSH DESCRIPTOR Respiratory Hypersensitivity EXPLODE ALL TREES)) oR ((MeSH DESCRIPTOR Diabetes Mellitus EXPLODE ALL TREES) OR (diabet*)) oR ((MeSH DESCRIPTOR Autoimmune Diseases EXPLODE ALL TREES) OR (((autoimmun* or auto immun* or autoaggress* or auto aggress*) AnD (disorder* or disease*)))) oR ((MeSH DESCRIPTOR Metabolic Syndrome X EXPLODE ALL TREES) OR (MeSH DESCRIPTOR Metabolic Diseases EXPLODE ALL TREES) OR (((metabolic or insulin resistance) AnD (disorder* or disease* or syndrome*)))) oR ((MeSH DESCRIPTOR Obesity EXPLODE ALL TREES) OR (obes*)) oR ((MeSH DESCRIPTOR Osteoporosis EXPLODE ALL TREES) OR (osteoporo* or bone loss) OR (MeSH DESCRIPTOR Osteolysis EXPLODE ALL TREES) OR (osteolysis or "bone resorption")) oR ((MeSH DESCRIPTOR Parkinson Disease EXPLODE ALL TREES) OR (parkinson* or "paralysis agitans")) oR ((MeSH DESCRIPTOR Arthritis EXPLODE ALL TREES) OR (arthriti* or polyarthriti* or rheumarthriti*)) oR ((MeSH DESCRIPTOR Kidney Diseases EXPLODE ALL TREES) OR ((kidney AnD (disease* or disorder*)))) oR ((MeSH DESCRIPTOR Liver Diseases EXPLODE ALL TREES) OR ((liver AnD (disease* or disorder* or dysfunction*)))) oR ((MeSH DESCRIPTOR Hypertension EXPLODE ALL TREES) OR (high blood pressure* or hypertens*)) oR ((MeSH DESCRIPTOR Hyperlipidemias EXPLODE ALL TREES) OR (hyperlipem* or hyperlipidem* or lipem* or lipidem*)) oR ((MeSH DESCRIPTOR Hypercholesterolemia EXPLODE ALL TREES) OR (((high* or elevat*) AnD cholesterol*)) OR (hypercholesterem* or hypercholesterolem*)) oR ((MeSH DESCRIPTOR Hypertriglyceridemia EXPLODE ALL TREES) OR (hypertriglyceridem*)) oR ((MeSH DESCRIPTOR Thyroid Diseases EXPLODE ALL TREES) OR ((thyroid AnD (disease* or disorder*))) OR (MeSH DESCRIPTOR Hyperthyroidism EXPLODE ALL TREES) OR (hypothyroid*) OR (((thyroid-stimulating hormone* or tsh) AnD deficien*))) oR ((MeSH DESCRIPTOR Motor Neuron Disease EXPLODE ALL TREES) OR (motor neuron* disease*) Or (lateral scleros*) OR (motor system disease*)) oR ((MeSH DESCRIPTOR Multiple Sclerosis EXPLODE ALL TREES) OR ("multiple sclerosis" or

"disseminated sclerosis")) oR ((MeSH DESCRIPTOR Emphysema EXPLODE ALL TREES) OR (emphysema*)) oR ((MeSH DESCRIPTOR Bronchitis EXPLODE ALL TREES) OR (bronchit*))) OR (((MeSH DESCRIPTOR Mental Disorders EXPLODE ALL TREES) OR (MeSH DESCRIPTOR Psychotic Disorders EXPLODE ALL TREES) OR (((mental* or psychiatr* or psycho*) AnD (disorder* or disease* or illness*)))) oR ((MeSH DESCRIPTOR Depressive Disorder, Major EXPLODE ALL TREES) OR (MeSH DESCRIPTOR Depression EXPLODE ALL TREES) OR (Depress* or MDD)) oR ((MeSH DESCRIPTOR Anxiety Disorders EXPLODE ALL TREES) oR (MeSH DESCRIPTOR Anxiety EXPLODE ALL TREES) OR (anxi*)) oR ((MeSH DESCRIPTOR Phobic Disorders EXPLODE ALL TREES) OR (phobi*)) oR ((MeSH DESCRIPTOR Schizophrenia EXPLODE ALL TREES) OR (schizophreni* or hebephreni*)) oR ((MeSH DESCRIPTOR Somatoform Disorders EXPLODE ALL TREES) OR (((somatoform* or somati* or "medically unexplained" or briquet or pain) AnD (disorder* or syndrome* or symptom*))) or (MeSH DESCRIPTOR Medically Unexplained Symptoms EXPLODE ALL TREES)) oR ((MeSH DESCRIPTOR Dissociative Disorders EXPLODE ALL TREES) OR ((dissociative AnD (disorder* or hysteri* or reaction*))) OR (dissociation*)) oR ((MeSH DESCRIPTOR Hysteria EXPLODE ALL TREES) OR (hysteri*)) oR ((MeSH DESCRIPTOR Mood Disorders EXPLODE ALL TREES) OR (((affective* or mood*) AnD (disorder* or disease* or illness* or symptom*)))) oR ((MeSH DESCRIPTOR Stress Disorders, Post-Traumatic EXPLODE ALL TREES) or (PTSD) OR (((post trauma* or posttrauma*) AnD (stress* or neurose*))) or ("combat disorder") OR ("war disorder")) oR ((MeSH DESCRIPTOR Cognition Disorders EXPLODE ALL TREES) OR (((cognitive or cognition or mental or neurocognitive) AnD (dysfunction* or decline* or impairment* or deterioration* or disorder* or illness* or disease*)))) oR ((MeSH DESCRIPTOR Personality Disorders EXPLODE ALL TREES) OR ("personality disorder") OR ("personality disorders")) oR ((impulse control disorder*) OR (intermittent explosive disorder*)) oR ((MeSH DESCRIPTOR Feeding and Eating Disorders EXPLODE ALL TREES) OR (((eating or appetite or feeding) AnD disorder*))) oR ((MeSH DESCRIPTOR Bipolar Disorder EXPLODE ALL TREES) OR (((bipolar or mani*) AnD (disorder* or illness* or disease*)))) oR ((MeSH DESCRIPTOR Obsessive-Compulsive Disorder EXPLODE ALL TREES) OR (OCD*) OR (((obsess*-compulsi* or obsess* or compulsi*) AnD (disorder* or illness* or disease* or neuros*)))) oR ((MeSH DESCRIPTOR Panic Disorder EXPLODE ALL TREES) OR ((panic AnD (attack* or disorder*)))) oR ((MeSH DESCRIPTOR Agoraphobia EXPLODE ALL TREES) OR (agoraphobi*)) oR ((MeSH DESCRIPTOR Neurotic Disorders EXPLODE ALL TREES) OR (neuros* or "neurotic disorder" or psychoneuros*))) OR (((MeSH DESCRIPTOR Communicable Diseases EXPLODE ALL TREES) OR (((communic* or contag* or transmi* or infect*) AnD (disease* or infection* or illness*)))) oR ((MeSH DESCRIPTOR Bacterial Infections EXPLODE ALL TREES) OR (bacteri* infection*)) oR ((MeSH DESCRIPTOR Conjunctivitis EXPLODE ALL TREES) OR (conjunctivitis)) oR ((MeSH DESCRIPTOR HIV EXPLODE ALL TREES) OR (hiv or "Human immuno deficiency virus")) oR ((MeSH DESCRIPTOR Acquired Immunodeficiency Syndrome EXPLODE ALL TREES) OR (AIDS) OR ("immunodeficiency associated virus") OR (immun* AND "deficiency associated virus") OR ("acquired immunodeficiency syndrome")) oR ((MeSH DESCRIPTOR Buruli Ulcer EXPLODE ALL TREES) OR (Bairnsdale or Buruli)) oR ((MeSH DESCRIPTOR Onchocerciasis EXPLODE ALL TREES) OR (onchocer*)) oR ((MeSH DESCRIPTOR Hepatitis B EXPLODE ALL TREES) OR (MeSH DESCRIPTOR Hepatitis C EXPLODE ALL TREES) OR (hepatitis)) oR ((MeSH DESCRIPTOR Leishmaniasis EXPLODE ALL TREES) OR (leishmania*)) oR ((MeSH DESCRIPTOR Leprosy EXPLODE ALL TREES) OR (lepros* or hansen*)) oR ((MeSH DESCRIPTOR Elephantiasis, Filarial EXPLODE ALL TREES) OR (elephantias* or filaria*)) oR ((MeSH DESCRIPTOR Trachoma EXPLODE ALL TREES) OR ("egyptian ophthalmia" or trachoma*)) oR ((MeSH DESCRIPTOR Chikungunya Fever EXPLODE ALL TREES) OR (chickungunya or chikungunya)) oR ((MeSH DESCRIPTOR Taeniasis EXPLODE ALL TREES) OR taenia*) oR ((MeSH DESCRIPTOR Cysticercosis EXPLODE ALL TREES) OR (cysticercos*)) oR ((MeSH DESCRIPTOR Echinococcosis EXPLODE ALL TREES)

OR (hydatid* or echinococc*)) oR ((MeSH DESCRIPTOR Chagas Disease EXPLODE ALL TREES) OR (trypanosom* or chagas)) oR ((MeSH DESCRIPTOR Trypanosomiasis EXPLODE ALL TREES) OR ("sleeping sickness")) oR ((MeSH DESCRIPTOR Encephalitis, Japanese EXPLODE ALL TREES) OR (japanese AnD encephalitis)) oR ((MeSH DESCRIPTOR Syphilis EXPLODE ALL TREES) OR (syphilis)))) AND ((MeSH DESCRIPTOR Tuberculosis EXPLODE ALL TREES) OR (Tuberculos*) OR (TB) OR (koch*))
1341
