# Supplementary material for: Prevalence and risks of tuberculosis multimorbidity in low-income and middle-income countries: a meta-review
Source: BMJ Open. 2022 Sep 28;12(9):e060906. doi: 10.1136/bmjopen-2022-060906 (PMC9528681; doi:10.1136/bmjopen-2022-060906)
Supplement: Supplementary data [file bmjopen-2022-060906supp003.pdf]

APPENDIX 1: References of included studies

S1 Huddart S, Svadzian A, Nafade V, *et al.* Tuberculosis case fatality in India: a systematic review and meta-analysis. *BMJ Glob Health* 2020;**5**:e002080. doi:10.1136/bmjgh-2019-002080

S2 Eshetie S, Gizachew M, Alebel A, *et al.* Tuberculosis treatment outcomes in Ethiopia from 2003 to 2016, and impact of HIV co-infection and prior drug exposure: A systematic review and meta-analysis. *PloS One* 2018;**13**:e0194675. doi:10.1371/journal.pone.0194675

S3 Ruiz-Grosso P, Cachay R, de la Flor A, *et al.* Association between tuberculosis and depression on negative outcomes of tuberculosis treatment: A systematic review and meta-analysis. *PloS One* 2020;**15**:e0227472. doi:10.1371/journal.pone.0227472

S4 Gautam S, Shrestha N, Mahato S, *et al.* Diabetes among tuberculosis patients and its impact on tuberculosis treatment in South Asia: a systematic review and meta-analysis. *Sci Rep* 2021;**11**:2113. doi:10.1038/s41598-021-81057-2

S5 Chem ED, Van Hout MC, Hope V. Treatment outcomes and antiretroviral uptake in multidrug-resistant tuberculosis and HIV co-infected patients in Sub Saharan Africa: a systematic review and meta-analysis. *BMC Infect Dis* 2019;**19**:723. doi:10.1186/s12879-019-4317-4

S6 Edessa D, Sisay M, Dessie Y. Unfavorable outcomes to second-line tuberculosis therapy among HIV-infected versus HIV-uninfected patients in sub-Saharan Africa: A systematic review and meta-analysis. *PloS One* 2020;**15**:e0237534. doi:10.1371/journal.pone.0237534

S7 Endalamaw A, Ambachew S, Geremew D, *et al.* HIV infection and unknown HIV status among tuberculosis patients in Ethiopia: a systematic review and meta-analysis. *Int J Tuberc Lung Dis Off J Int Union Tuberc Lung Dis* 2019;**23**:187–94. doi:10.5588/ijtld.18.0363

S8 Gao L, Zhou F, Li X, *et al.* HIV/TB co-infection in mainland China: a meta-analysis. *PloS One* 2010;**5**:e10736. doi:10.1371/journal.pone.0010736

S9 Gao J, Zheng P, Fu H. Prevalence of TB/HIV co-infection in countries except China: a systematic review and meta-analysis. *PloS One* 2013;**8**:e64915. doi:10.1371/journal.pone.0064915

S10 Gelaw YA, Williams G, Soares Magalhaes RJ, *et al.* HIV Prevalence Among Tuberculosis Patients in Sub-Saharan Africa: A Systematic Review and Meta-analysis. *AIDS Behav* 2019;**23**:1561–75. doi:10.1007/s10461-018-02386-4

S11 Mekonnen D, Derbie A, Abeje A, *et al.* Epidemiology of tuberculous lymphadenitis in Africa: A systematic review and meta-analysis. *PloS One* 2019;**14**:e0215647. doi:10.1371/journal.pone.0215647

S12 Samuels JP, Sood A, Campbell JR, *et al.* Comorbidities and treatment outcomes in multidrug resistant tuberculosis: a systematic review and meta-analysis. *Sci Rep* 2018;**8**:4980. doi:10.1038/s41598-018-23344-z

S13 Wang M-G, Luo L, Zhang Y, *et al.* Treatment outcomes of tuberculous meningitis in adults: a systematic review and meta-analysis. *BMC Pulm Med* 2019;**19**:200. doi:10.1186/s12890-019-0966-8

S14 Alebel A, Wondemagegn AT, Tesema C, *et al.* Prevalence of diabetes mellitus among tuberculosis patients in Sub-Saharan Africa: a systematic review and meta-analysis of observational studies. *BMC Infect Dis* 2019;**19**:254. doi:10.1186/s12879-019-3892-8

S15 Chen H, Liu M, Gu F. [Meta-analysis on the co-morbidity rate between tuberculosis and diabetes mellitus in China]. *Zhonghua Liu Xing Bing Xue Za Zhi Zhonghua Liuxingbingxue Zazhi* 2013;**34**:1128–33.

S16 Huangfu P, Ugarte-Gil C, Golub J, *et al.* The effects of diabetes on tuberculosis treatment outcomes: an updated systematic review and meta-analysis. *Int J Tuberc Lung Dis Off J Int Union Tuberc Lung Dis* 2019;**23**:783–96. doi:10.5588/ijtld.18.0433

S17 Noubiap JJ, Nansseu JR, Nyaga UF, *et al.* Global prevalence of diabetes in active tuberculosis: a systematic review and meta-analysis of data from 2.3 million patients with tuberculosis. *Lancet Glob Health* 2019;**7**:e448–60. doi:10.1016/S2214-109X(18)30487-X

S18 S.H Y, M.-L D, D J, *et al.* Risk factors of retreatment pulmonary tuberculosis patients with unfavorable treatment outcome in China: A meta-analysis. *Chin J Evid-Based Med* 2016;**16**:795–801.

S19 Alene KA, Clements ACA, McBryde ES, *et al.* Mental health disorders, social stressors, and health-related quality of life in patients with multidrug-resistant tuberculosis: A systematic review and meta-analysis. *J Infect* 2018;**77**:357–67. doi:10.1016/j.jinf.2018.07.007

S20 Duko B, Bedaso A, Ayano G. The prevalence of depression among patients with tuberculosis: a systematic review and meta-analysis. *Ann Gen Psychiatry* 2020;**19**:30. doi:10.1186/s12991-020-00281-8

S21 Ga Eun Lee JS Jerome Galea, Sanghyuk Shin, Annika Sweetland, Elizabeth Magill. Impact of mental illness on active tuberculosis (TB) treatment outcome: a systematic review and meta-analysis.

S22 Behzadifar M, Heydarvand S, Behzadifar M, *et al.* Prevalence of Hepatitis C Virus in Tuberculosis Patients: A Systematic Review and Meta-Analysis. *Ethiop J Health Sci* 2019;**29**:945–56. doi:10.4314/ejhs.v29i1.17

S23 C.Y L, H.-L H, M.M R, *et al.* Cancer incidence attributable to tuberculosis in 2015: Global, regional, and national estimates. *BMC Cancer* 2020;**20**:412.

S24 Arega B, Minda A, Mengistu G, *et al.* Unknown HIV status and the TB/HIV collaborative control program in Ethiopia: systematic review and meta-analysis. *BMC Public Health* 2020;**20**:1021. doi:10.1186/s12889-020-09117-2

S25 Bastos SH, Taminato M, Fernandes H, *et al.* Sociodemographic and health profile of TB/HIV co-infection in Brazil: a systematic review. *Rev Bras Enferm* 2019;**72**:1389–96. doi:10.1590/0034-7167-2018-0285

S26 Lukoye D, Ssengooba W, Musisi K, *et al.* Variation and risk factors of drug resistant tuberculosis in sub-Saharan Africa: a systematic review and meta-analysis. *BMC Public Health* 2015;**15**:291. doi:10.1186/s12889-015-1614-8

S27 McMurry HS, Mendenhall E, Rajendrakumar A, *et al.* Coprevalence of type 2 diabetes mellitus and tuberculosis in low-income and middle-income countries: A systematic review. *Diabetes Metab Res Rev* 2019;**35**:e3066. doi:10.1002/dmrr.3066

S28 Pourakbari B, Mamishi S, Banar M, *et al.* Prevalence of TB/ HIV co-infection in Iran: a systematic review and meta-analysis. *Ann Ig Med Prev E Comunita* 2019;**31**:333–48. doi:10.7416/ai.2019.2295

S29 Rajendran M, Zaki RA, Aghamohammadi N. Contributing risk factors towards the prevalence of multidrug-resistant tuberculosis in Malaysia: A systematic review. *Tuberc Edinb Scotl* 2020;**122**:101925. doi:10.1016/j.tube.2020.101925

S30 Reddy EA, Shaw AV, Crump JA. Community-acquired bloodstream infections in Africa: a systematic review and meta-analysis. *Lancet Infect Dis* 2010;**10**:417–32. doi:10.1016/S1473-3099(10)70072-4

S31 Seid MA, Ayalew MB, Muche EA, *et al.* Drug-susceptible tuberculosis treatment success and associated factors in Ethiopia from 2005 to 2017: a systematic review and meta-analysis. *BMJ Open* 2018;**8**:e022111. doi:10.1136/bmjopen-2018-022111

S32 Straetemans M, Glaziou P, Bierrenbach AL, *et al.* Assessing tuberculosis case fatality ratio: a meta-analysis. *PloS One* 2011;**6**:e20755. doi:10.1371/journal.pone.0020755

S33 Tesfaye B, Alebel A, Gebrie A, *et al.* The twin epidemics: Prevalence of TB/HIV co-infection and its associated factors in Ethiopia; A systematic review and meta-analysis. *PloS One* 2018;**13**:e0203986. doi:10.1371/journal.pone.0203986

S34 Teweldemedhin M, Asres N, Gebreyesus H, *et al.* Tuberculosis-Human Immunodeficiency Virus (HIV) co-infection in Ethiopia: a systematic review and meta-analysis. *BMC Infect Dis* 2018;**18**:676. doi:10.1186/s12879-018-3604-9

S35 Uchida S, Komiya K, Honjo K, *et al.* A mini systematic review of prognostic factors in elderly patients with tuberculosis. *Respir Investig* 2019;**57**:207–12. doi:10.1016/j.resinv.2018.12.004

S36 Waitt CJ, Squire SB. A systematic review of risk factors for death in adults during and after tuberculosis treatment. *Int J Tuberc Lung Dis Off J Int Union Tuberc Lung Dis* 2011;**15**:871–85. doi:10.5588/ijtld.10.0352

S37 Baker MA, Harries AD, Jeon CY, *et al.* The impact of diabetes on tuberculosis treatment outcomes: a systematic review. *BMC Med* 2011;**9**:81. doi:10.1186/1741-7015-9-81

S38 Han X, Wang Q, Wang Y, *et al.* The impact of diabetes on tuberculosis treatment outcomes: evidence based on a cumulative meta-analysis. *Int J DIABETES Dev Ctries* 2016;**36**:490–507. doi:10.1007/s13410-016-0514-5

S39 Huang D, Wang Y, Wang Y, *et al.* The impact of diabetes mellitus on drug resistance in patients with newly diagnosed tuberculosis: a systematic review and meta-analysis. *Ann Palliat Med* 2020;**9**:152–62. doi:10.21037/apm.2020.02.16

S40 Jeon CY, Harries AD, Baker MA, *et al.* Bi-directional screening for tuberculosis and diabetes: a systematic review. *Trop Med Int Health TM IH* 2010;**15**:1300–14. doi:10.1111/j.1365-3156.2010.02632.x

S41 Lutfiana NC, van Boven JFM, Masoom Zubair MA, *et al.* Diabetes mellitus comorbidity in patients enrolled in tuberculosis drug efficacy trials around the world: A systematic review. *Br J Clin Pharmacol* 2019;**85**:1407–17. doi:10.1111/bcp.13935

S42 Tegegne BS, Mengesha MM, Teferra AA, *et al.* Association between diabetes mellitus and multi-drug-resistant tuberculosis: evidence from a systematic review and meta-analysis. *Syst Rev* 2018;**7**:161. doi:10.1186/s13643-018-0828-0

S43 Workneh MH, Bjune GA, Yimer SA. Prevalence and associated factors of tuberculosis and diabetes mellitus comorbidity: A systematic review. *PloS One* 2017;**12**:e0175925. doi:10.1371/journal.pone.0175925

S44 A JVR, A D, R C, *et al.* Comorbidities between tuberculosis and common mental disorders: A scoping review of epidemiological patterns and person-centred care interventions from low-to-middle income and BRICS countries. *Infect Dis Poverty* 2020;**9**:4.

S45 Basham CA, Smith SJ, Romanowski K, *et al.* Cardiovascular morbidity and mortality among persons diagnosed with tuberculosis: A systematic review and meta-analysis. *PloS One* 2020;**15**:e0235821. doi:10.1371/journal.pone.0235821

S46 Rehm J, Samokhvalov AV, Neuman MG, *et al.* The association between alcohol use, alcohol use disorders and tuberculosis (TB). A systematic review. *BMC Public Health* 2009;**9**:450. doi:10.1186/1471-2458-9-450

S47 Sotgiu G, Ferrara G, Matteelli A, *et al.* Epidemiology and clinical management of XDR-TB: a systematic review by TBNET. *Eur Respir J* 2009;**33**:871–81. doi:10.1183/09031936.00168008

S48 Bisson GP, Bastos M, Campbell JR, *et al.* Mortality in adults with multidrug-resistant tuberculosis and HIV by antiretroviral therapy and tuberculosis drug use: an individual patient data meta-analysis. *Lancet Lond Engl* 2020;**396**:402–11. doi:10.1016/S0140-6736(20)31316-7

S49 Mesfin YM, Hailemariam D, Biadgilign S, *et al.* Association between HIV/AIDS and multi-drug resistance tuberculosis: a systematic review and meta-analysis. *PloS One* 2014;**9**:e82235. doi:10.1371/journal.pone.0082235

S50 Wu S, Zhang Y, Sun F, *et al.* Adverse Events Associated With the Treatment of Multidrug-Resistant Tuberculosis: A Systematic Review and Meta-analysis. *Am J Ther* 2016;**23**:e521-30. doi:10.1097/01.mjt.0000433951.09030.5a

S51 de Almeida CPB, Ziegelmann PK, Couban R, *et al.* Predictors of In-Hospital Mortality among Patients with Pulmonary Tuberculosis: A Systematic Review and Meta-analysis. *Sci Rep* 2018;**8**:7230. doi:10.1038/s41598-018-25409-5

S52 Pormohammad A, Nasiri MJ, Riahi SM, *et al.* Human immunodeficiency virus in patients with tuberculous meningitis: systematic review and meta-analysis. *Trop Med Int Health TM IH* 2018;**23**:589–95. doi:10.1111/tmi.13059

S53 Purmohamad A, Azimi T, Nasiri MJ, *et al.* HIV-Tuberculous Meningitis Co-infection: A Systematic Review and Meta-analysis. *Curr Pharm Biotechnol* Published Online First: 2020. doi:10.2174/1389201021666200730143906

S54 Alemu A, Bitew ZW, Worku T. Poor treatment outcome and its predictors among drug-resistant tuberculosis patients in Ethiopia: A systematic review and meta-analysis. *Int J Infect Dis IJID Off Publ Int Soc Infect Dis* 2020;**98**:420–39. doi:10.1016/j.ijid.2020.05.087

S55 Wongtrakul W, Charoenngam N, Ungprasert P. Tuberculosis and risk of coronary heart disease: A systematic review and meta-analysis. *Indian J Tuberc* 2020;**67**:182–8. doi:10.1016/j.ijtb.2020.01.008
