# Supplementary material for: Prevalence and risks of tuberculosis multimorbidity in low-income and middle-income countries: a meta-review
Source: BMJ Open. 2022 Sep 28;12(9):e060906. doi: 10.1136/bmjopen-2022-060906 (PMC9528681; doi:10.1136/bmjopen-2022-060906)
Supplement: Author's manuscript [file bmjopen-2022-060906.draft_revisions.pdf]

BMJ Open is committed to open peer review. As part of this commitment we make the peer review history of every article we publish publicly available.

When an article is published we post the peer reviewers' comments and the authors' responses online. We also post the versions of the paper that were used during peer review. These are the versions that the peer review comments apply to.

The versions of the paper that follow are the versions that were submitted during the peer review process. They are not the versions of record or the final published versions. They should not be cited or distributed as the published version of this manuscript.

BMJ Open is an open access journal and the full, final, typeset and author-corrected version of record of the manuscript is available on our site with no access controls, subscription charges or pay-per-view fees (http://bmjopen.bmj.com).

If you have any questions on BMJ Open's open peer review process please email info.bmjopen@bmj.com

BMJ Open

Prevalence and risks of tuberculosis multimorbidity in low- and middle-income countries: a meta-review

Journal:	*BMJ Open*
Manuscript ID	bmjopen-2022-060906
Article Type:	Original research
Date Submitted by the Author:	08-Jan-2022

Complete List of Authors:	Jarde, Alexander; University of York,<br>Romano, Eugenia; King's College London Institute of Psychiatry Psychology and Neuroscience, Department of Psychological Medicine<br>Afaq, Saima; Khyber Medical University, Institute of Public Health and Social Sciences; Imperial College London, Department of Epidemiology and Biostatistics<br>Elsony, Asma; Epi-Lab, Public Health<br>Lin, Yan; International Union Against Tuberculosis and Lung Disease<br>Huque, Rumana; University of Dhaka, Economics<br>Elsey, Helen ; University of York; Hull York Medical School<br>Siddiqi, Kamran; University of York, Institute of Health Sciences; Hull York Medical School<br>Stubbs, B; King's College London Institute of Psychiatry Psychology and Neuroscience, Department of Psychological Medicine<br>Siddiqi, Najma; University of York, Department of Health Sciences; Hull York Medical School

Keywords:	Epidemiology < INFECTIOUS DISEASES, Public health < INFECTIOUS DISEASES, Tuberculosis < INFECTIOUS DISEASES

SCHOLARONE™
Manuscripts

I, the Submitting Author has the right to grant and does grant on behalf of all authors of the Work (as defined in the below author licence), an exclusive licence and/or a non-exclusive licence for contributions from authors who are: i) UK Crown employees; ii) where BMJ has agreed a CC-BY licence shall apply, and/or iii) in accordance with the terms applicable for US Federal Government officers or employees acting as part of their official duties; on a worldwide, perpetual, irrevocable, royalty-free basis to BMJ Publishing Group Ltd ("BMJ") its licensees and where the relevant Journal is co-owned by BMJ to the co-owners of the Journal, to publish the Work in this journal and any other BMJ products and to exploit all rights, as set out in our licence.

The Submitting Author accepts and understands that any supply made under these terms is made by BMJ to the Submitting Author unless you are acting as an employee on behalf of your employer or a postgraduate student of an affiliated institution which is paying any applicable article publishing charge ("APC") for Open Access articles. Where the Submitting Author wishes to make the Work available on an Open Access basis (and intends to pay the relevant APC), the terms of reuse of such Open Access shall be governed by a Creative Commons licence – details of these licences and which Creative Commons licence will apply to this Work are set out in our licence referred to above.

Other than as permitted in any relevant BMJ Author's Self Archiving Policies, I confirm this Work has not been accepted for publication elsewhere, is not being considered for publication elsewhere and does not duplicate material already published. I confirm all authors consent to publication of this Work and authorise the granting of this licence.

For peer review only

Prevalence and risks of tuberculosis multimorbidity in low- and middle-income countries: a meta-review

Running title: Meta-review of TB multimorbidity

Authors

Alexander Jarde, PhD - Department of Health Sciences, University of York, York, United Kingdom.
Eugenia Romano, PhD - Department of Psychological Medicine, Institute of Psychiatry, Psychology & Neuroscience, King's College London, London, United Kingdom.
Saima Afaq, PhD - Institute of Public Health and Social Sciences, Khyber Medical University, Peshawar, Pakistan.
Asma Elsony, PhD - Epi-Lab: The Epidemiological Laboratory, Khartoum, Sudan.
Yan Lin, MD - International Union Against Tuberculosis and Lung Disease, China.
Rumana Huque, Prof - Department of Economics, University of Dhaka, Dhaka, Bangladesh.
Helen Elsey, PhD - Department of Health Sciences, University of York, and Hull York Medical School, York, United Kingdom.
Kamran Siddiqi, PhD - Department of Health Sciences, University of York, and Hull York Medical School, York, United Kingdom.
Brendon Stubbs, PhD - Department of Psychological Medicine, Institute of Psychiatry, Psychology & Neuroscience, King's College London, London, United Kingdom.
Najma Siddiqi, PhD - Department of Health Sciences, University of York, and Hull York Medical School, York, United Kingdom.

Correspondence to: Alexander Jarde: a.jarde@gmail.com; Department of Health Sciences, University of York, Heslington, York YO10 5DD, United Kingdom. Telephone: 0190432 1327

Word count: 3528

For peer review only - http://bmjopen.bmj.com/site/about/guidelines.xhtml

Abstract (264 / 300)

Objectives: Co-occurrence of tuberculosis (TB) with other chronic conditions (TB multimorbidity) increases complexity of management and adversely affects health outcomes. We aimed to map the prevalence of the co-occurrence of one or more chronic conditions in people with tuberculosis (TB) and associated health risks by systematically reviewing previously published systematic reviews.

Design: Systematic review of systematic reviews (meta-review).

Setting: Low- and middle-income countries (LMIC).

Papers: We searched in multiple major databases from inception to 23/10/2020, contacted authors, and reviewed reference lists. Pairs of independent reviewers screened titles, abstracts and full texts, extracted data, and assessed the included reviews' quality (AMSTAR2). We included systematic reviews reporting data for people in LMIC with TB multimorbidity and synthesised them narratively. We excluded reviews focused on children or specific subgroups (e.g. incarcerated people).

Primary and secondary outcome measures: Prevalence or risk of TB multimorbidity (primary); any measure of burden of disease (secondary).

Results: From the 7,557 search results, 54 were included, representing >6,296,000 people with TB. We found that the most prevalent conditions in people with TB were depression (45.19%, 95% Confidence Interval [CI] 38.04%-52.55%, 25 studies, 4,903 participants, I^2=96.28%, high quality), HIV (31.81%, 95%CI 27.83%-36.07%, 68 studies, 62,696 participants, I^2=98%, high quality), and diabetes mellitus (17.7%, 95%CI 15.1%-20.0.5%, 3 studies, 578 participants, I^2=81.4%, critically low quality).

Conclusions: We identified several chronic conditions that co-occur in a significant proportion of people with TB. Although limited by varying quality and gaps in the literature, this first meta-review of TB multimorbidity highlights the magnitude of additional ill health burden due to chronic conditions on people with TB.

Registration: Prospero CRD42020209012

Key words: Tuberculosis; Noncommunicable Diseases; Chronic Disease; Mental Disorders; Communicable Diseases; HIV; Diabetes Mellitus; Comorbidity; Multiple Chronic Conditions

Strengths and limitations of this study

- We did an extensive search strategy, including databases of grey literature and protocols.
- We summarised data synthesised at the country, regional (e.g. Eastern Sub-Saharan Africa), continental, and global (LMIC) level as long as the pooled estimate did not include data from high-income countries.
- Whenever there was an overlap between two reviews in terms of countries covered, TB comorbidities, and reported outcomes, we included the most complete one only if its quality, as assessed with the AMSTAR2 tool, was not lower than the other one.
- Although we had initially planned to re-do reported meta-analyses that included studies from high-income countries without these studies (to have pooled estimate from LMICs only), this was deemed unfeasible due to the high number of reviews where this would have been required.

For peer review only - http://bmjopen.bmj.com/site/about/guidelines.xhtml

INTRODUCTION

About 30% adults in developed countries experience multimorbidity, i.e. the co-occurrence of two or more chronic conditions (including non-communicable diseases [NCDs], chronic communicable diseases [CCDs] and mental disorders) in a single individual at one point in time. Multimorbidity is a growing global concern[1] and its prevalence is rising in low- and middle-income countries (LMICs),[2] where NCDs are increasing[3] and where CCDs such as tuberculosis (TB) and HIV remain major public health issues.[4] This is also reflected in the steady increase in the burden of disability-adjusted life years (DALYs) attributed to NCDs over the past decades,[5] reaching 34% in low-income countries, and up to 82% in middle-high-income countries in 2019.[6] TB is one of the leading cause of mortality from a single infectious disease globally[7] and contributes 1.86% of the total worldwide DALYs and 2.54% of the total worldwide years of life lost (making it the 12th and 11th highest contributor, respectively).[6,8] TB frequently co-occurs with NCDs, including diabetes mellitus (DM), depression, and cancer.[9] Depression[10] and DM[11] have been reported to be important risk factors for TB. Similarly, CCDs such as HIV and TB adversely affect each other at the molecular, cellular, individual and population levels.[12]

We defined TB multimorbidity as the co-occurrence of TB and one or more chronic conditions (NCDs or CCDs).[13] This co-occurrence increases complexity of management and adversely affects health, economic and mortality outcomes, threatening the capacity for LMIC to achieve global public health targets. The cost and access to healthcare is of particular concern in LMICs, where the high costs relating to TB multimorbidity may further burden healthcare systems already under stress, and given the high out-of-pocket expenditure, it could lead to great financial burden for patients.

Numerous systematic reviews to date have considered individual chronic conditions in people with TB (e.g.[14–17]). However, no review has synthesised the evidence on a range of chronic conditions, their prevalence in people with TB and the burden associated with such co-occurrence of conditions. Understanding the overarching literature on TB multimorbidity is essential to enable better services to be developed to identify, prevent, and manage this common situation, which presents a significant health and financial burden to people with TB and to health services. Furthermore, differences in TB multimorbidity by gender, socio-economic group and country, which could shed further light on the problem, remain unclear.

The primary aim of this comprehensive meta-review of systematic reviews was to summarise and map the prevalence and risk of chronic conditions (CCD or NCD, alone or in combination) in people with TB in LMICs compared with people without TB, and to summarise the associated health outcomes (e.g. TB treatment success and measures of disease burden) in people with TB multimorbidity, compared with people with TB only.

METHODS

We have followed the PRISMA guidelines[18] in reporting this meta-review and its protocol was registered in the international prospective register of systematic reviews (PROSPERO, CRD42020209012).

Search strategy

We ran our search strategy in Medline (Ovid), Embase (Ovid), PsycINFO (Ovid), Social Sciences Citation Index (Web of Science), Science Citation Index (Web of Science), Emerging Sources Citation Index and Conference Proceedings Citation Index (Web of Science), and the WHO Global Index Medicus from inception

For peer review only - http://bmjopen.bmj.com/site/about/guidelines.xhtml

to October 23rd, 2020. To identify unpublished studies, we also searched PROSPERO and the Open Grey database, and contacted authors of conference abstracts. Reference lists of included reviews were hand searched. We did not set any restrictions on the origin of the paper, date of publication, or language.

We used free text and controlled vocabulary (e.g., MeSH terms for Medline) for terms related to communicable, non-communicable, and mental diseases and combined them with terms for TB using Boolean operators: (CCD or NCD or mental disease) AND Tuberculosis. Supplementary Table 1 lists the search terms for Medline.

Selection criteria

We included systematic reviews reporting data for people in LMICs, with any type of TB and one or more additional chronic conditions. This included, but was not limited to, heart disease, DM, arthritis, chronic obstructive pulmonary disease (COPD), HIV, Hepatitis B (HBV) and Hepatitis C (HCV), depression, and anxiety disorders (as defined by review authors). As there is no clear and widely used definition of what constitutes a chronic condition,[19] whenever there were doubts, four of the authors with clinical/research expertise (KS, NS, HE, BS) decided by consensus if a disorder was to be considered as a chronic condition. Conditions arising because of TB treatment were not considered chronic conditions for this review.

After registering the protocol, the following additional changes were made: First, we decided to limit our population of interest to the general TB population, excluding studies that stated focusing on children. Second, we decided to exclude studies focused on specific subgroups (e.g., incarcerated people, health care workers, etc.), focussing on populations for which results are more readily generalisable. Studies in patients with a specific type of TB (e.g., extra-pulmonary TB) were, however, considered eligible.

Included systematic reviews had to report either pooled or individual study data for at least one of our primary or secondary outcomes. Narrative, non-systematic reviews and systematic reviews focused only on high-income countries (HIC) were excluded.

Primary outcomes

The co-primary outcomes included prevalence (or incidence) of each chronic condition (or combination of more than one condition) in people with TB, and odds ratios (or other comparative statistic) of having a chronic condition (or combination of conditions) in people with TB compared to those without TB.

Secondary outcomes

Secondary outcomes included any measure of disease burden in people with TB multimorbidity, such as mortality, loss to follow up (treatment interrupted for two consecutive months or more), treatment failure (sputum smear or culture remained positive at month five or later during treatment), treatment completion (without evidence of failure, but with no record of being cured), cured (smear- or culture-negative patients in the last month of treatment and on at least one previous occasion), successful treatment (patients who were cured or who completed treatment), or unsuccessful treatment (patients who were lost to follow-up, had treatment failure, or died).[20,21] Other secondary outcomes of interest included years of life lived with disability, years of life lost, DALYs, outcomes related to the additional chronic conditions, and any other reported measure of disease burden.

Study selection

For peer review only - http://bmjopen.bmj.com/site/about/guidelines.xhtml

Multiple authors (ER, SA, AJ, NS) contributed to the screening and data extraction procedures, with titles and abstracts of all deduplicated search results screened independently by at least two reviewers. The full text of potentially eligible papers was reviewed against our inclusion and exclusion criteria independently by two reviewers. Disagreements were resolved by discussion, with a third reviewer available as an arbitrator if necessary. We used the online software Rayyan (https://rayyan.ai/) to manage the study selection process.

Data extraction and quality assessment

Two reviewers used a piloted form (Google Form) developed for the review to independently extract data regarding review characteristics, characteristics of included primary studies, and outcome data. If clarifications were needed, we contacted the corresponding authors.

The quality of included systematic reviews was assessed by two reviewers (ER and SA, with discrepancies resolved by agreement or a third independent assessor, AJ) using the AMSTAR2 tool, which classifies the overall confidence in the results of each review as critically low, low, moderate, or high.[22]

Data synthesis

The following steps were followed to synthesise the evidence: First, all included systematic reviews were described in a summary table. Second, the results (primary and secondary outcomes) for each combination of conditions were summarised, including the pooled estimates, the number of studies, pooled sample size, a measure of heterogeneity, range of pooled effect sizes, and quality assessment. Third, the results were stratified by age, gender, socio-economic group, type of TB and region, where possible. We had initially planned to extract and pool individual study data for LMICs when such studies had been pooled together with data from HICs, or when individual study data were reported but not pooled in a meta-analysis. However, such an approach was deemed unfeasible due to the high number of reviews where this would have been required. In these cases, we reported the study characteristics and the range of study effect sizes from LMICs.

Patient and Public Involvement

Patients or the public were not involved in the design, or conduct, or reporting, or dissemination plans of our research.

The funder of the study had no role in study design, data collection, data analysis, data interpretation, or writing of the report.

RESULTS

Our search strategy identified 7,557 results, of which 2,200 were duplicates and were removed. Of the 221 results remaining after screening titles and abstracts, 130 were excluded for not meeting eligibility criteria. Supplementary Table 2 specifies the reasons for exclusion. The full text corresponding to 34 protocols or conference abstracts could not be obtained. We contacted the authors of these references (with a follow-up email two weeks later), seven of which replied confirming that no full article had been published. Three journal articles, related to coronary heart disease, head and neck TB, and HBV, could not be assessed in full text despite our efforts (no institutional access and no response from authors[23–25]). The full text of one additional

For peer review only - http://bmjopen.bmj.com/site/about/guidelines.xhtml

study[23] could not be obtained, but the pooled relative risk of coronary heart disease was reported in the abstract and was therefore included. Ultimately, 54 studies were included in our review (Figure 1).

Study and participant characteristics

Overall, there were over 6,296,000 people with TB across the 54 included systematic reviews, covering 85 LMICs (Appendix 1). Of these, 23 reported a pooled estimate of interest to our review,[S1–S23] while the remainder reported outcomes of interest for individual studies, but either did not pool them in a meta-analysis or pooled them with data from HICs. Among the 23 reviews reporting pooled outcomes, even when they assessed the same combination of TB and chronic condition(s), there was limited overlap between them with regards to geographical region and/or reported outcomes (Supplementary Table 3). Supplementary Table 4 details outcome information reported by each review.

Most of the included systematic reviews reported data on TB without specifying a particular type of TB;[S1–S4,S7–S10,S14,S16,S17,S20–S46] nine focused on drug-resistant TB (DR-TB),[S6] multidrug-resistant TB (MDR-TB),[S5,S12,S19,S47–S50] or extensively drug-resistant TB (XDR-TB);[S12,S47] three focused on pulmonary TB (PTB),[S15,S18,S51] three on TB meningitis,[S13,S52,S53] and one on TB lymphadenitis.[S11] The chronic conditions most often considered were HIV (31 reviews),[S1,S2,S5–S13,S24–S36,S47–S50,S52–S54] DM (14 reviews),[S4,S14–S18,S37–S43,S51] and mental illness (5 reviews).[S3,S19–S21,S44] Supplementary Table 5 lists what conditions were considered or not a chronic condition for this review.

Most of the identified systematic reviews were assessed as low or critically low quality according to AMSTAR2 (n=42). Only seven reviews were assessed as moderate (n=2)[S7,S22] or high (n=5)[S6,S10,S11,S20,S42] quality, six of which reported a pooled estimate of interest. The critical domains that failed most often were regarding risk of bias assessment (37 studies) and protocol registration (29 studies). Supplementary Table 6 details the AMSTAR2 assessment for each study.

For peer review only - http://bmjopen.bmj.com/site/about/guidelines.xhtml

Summary of results

TB and HIV

Of the 31 reviews reporting data on TB and HIV (>3,017,000 participants from 72 countries),[S1,S2,S5–S13,S24–S36,S47–S50,S52–S54] 11 focused on specific types of TB,[S5,S6,S11–S13,S47–S50,S52,S53] and 11 reported at least one pooled outcome of interest (Supplementary Table 3).[S1,S2,S5–S13]

One review[S9] reported the pooled prevalence for Latin America (25%, 95%CI 19.3%-30.8%, 7 studies, critically low quality) and Africa (31.2%, 95%CI 19.3%-43.2%, 17 studies, critically low quality). Prevalence estimates for sub-continental regions were also reported in other reviews, ranging from 25% in Western Sub-Saharan Africa (SSA, high quality) to 44% in Southern SSA (high quality), as well as for China, Ethiopia, and Iran (Table 1).

One review[S5] reported a reduced odds of treatment success (OR 0.87, 95%CI 0.79-0.96, 6 studies, critically low quality) in people with TB and HIV compared to people with only TB, in SSA.

Table 1 also summarises the results of systematic reviews reporting data for specific types of TB (DR-TB, and MDR-TB, PTB, TB meningitis, and TB lymphadenitis).

Table 1: Prevalence of HIV and effect on outcomes in people with tuberculosis

Region	Outcome (quality¶)	Effect size (95% CI)	Range	Number of studies (number of participants)	I^2	Year
Latin America	Prevalence (Critically low)[S9]	25% (19.3% - 30.8%)	NR	7 (NR)	95.2%	2013
Africa	Prevalence(Critically low)[S9]	31.2% (19.3% - 43.2%)	NR	17 (NR)	99.6%	2013
SSA	Prevalence (High)[S10]	31.81% (27.83% -36.07%)	6.03%-72.25%	68 (62,696)	98%	2019
	Successful treatment (Critically low)[S5]	OR 0.87 (0.79 - 0.96)	0.75-1.26	6 (NR)	NR	2019
Western SSA	Prevalence (High)[S10]	25.48% (19.70%-32.27%)	10.26%-72.13%	21 (16,145)	98%	2019
Eastern SSA	Prevalence (High)[S10]	31.14% (25.39%-37.54%)	6.03%-60.51%	32 (33,637)	98%	2019
Southern SSA	Prevalence (High)[S10]	43.67% (35.05%-52.69%)	23.84%-72.25%	12 (11,148)	99%	2019
Central SSA	Prevalence (High)[S10]	41.33% (30.39%-53.19%)	31.29%-51.56%	3 (2,039)	96%	2019
China	Prevalence(Critically low)[S8]	0.9% (0.6%–1.4%)	0.1%-4.5%	18 (NR)	92.21%	2010
	Prevalence in Women (Critically low)[S8]	0.6% (0.3%-1.1%)	NR	9 (NR)	71.8%	2010
	Prevalence in Men (Critically low)[S8]	1.1% (0.6%-2.0%)	NR	9 (NR)	94.7%	2010
Ethiopia	Prevalence (Moderate)[S7]	23.40% (19.56%-27.24%)	9.50%-52.10%	13 (19,212)	97.6%	2019
	Successful treatment (Critically low)[S2]	67% (56%-79%)	NR	NR (NR)	NR	2018
	Unsuccessful treatment (Critically low)[S2]	33% (21%-44%)	NR	NR (NR)	NR	2018
	Unsuccessful treatment (Critically low)[S2]	OR 1.98 (1.56-2.52)	0.82-14.31	20 (NR)	81.0%	2018
India	Case-fatality rate during treatment (Critically low)[S1]	10.91% (7.68%-15.50%)*	NR	35 (NR)	Tau2=0.90†	2020
	Case-fatality rate after treatment (Critically low)[S1]	4.15% (1.06% to 16.24%)	NR	5 (NR)	Tau2=1.90†	2020
Iran	Prevalence (Critically low) [S28]	14% (12% - 15%)	0%-54%	48 (21,388)	97.93%	2019

For peer review only - http://bmjopen.bmj.com/site/about/guidelines.xhtml

Drug resistant TB + HIV						
SSA	Unsuccessful treatment (High)[S6]	RR 1.18 (1.07-1.30)‡,§	0.71-2.37	19 (8,301)	48%	2020
	Treatment failure (High)[S6]	RR 0.66 (0.38-1.13) ‡	0.15-2.40	10 (5,474)	73%	2020
	Loss to follow-up (High)[S6]	RR 0.82 (0.74-0.92) ‡	0.49-2.61	14 (7,051)	0%	2020
	Mortality (High)[S6]	RR 1.50 (1.30-1.74) ‡	0.73-2.18	16 (7,365)	39%	2020
Western SSA	Unsuccessful treatment (High)[S6]	RR 1.42 (0.95-2.13)	1.31-2.37	2 (790)	12%	2020
	Mortality (High)[S6]	RR 1.42 (0.96-2.09)	NA	1 (588)	NA	2020
Eastern SSA	Unsuccessful treatment (High)[S6]	RR 1.47 (1.23-1.75)	1.14-1.77	6 (1,970)	0%	2020
	Mortality (High)[S6]	RR 1.52 (1.19–1.93)	1.20-2.18	5 (1,442)	0%	2020
Southern SSA	Unsuccessful treatment (High)[S6]	RR 1.09 (0.98-1.20) ‡	0.71-1.41	11 (5,541)	43%	2020
	Mortality (High)[S6]	RR 1.49 (1.21-1.83) ‡	0.73-1.47	10 (5,335)	60%	2020
Multidrug resistant TB + HIV						
LMIC	Unsuccessful treatment (Low)[S12]	RR 1.34 (1.04-1.72)	0.55-3.33	13 (5816)	88%	2018
Low income countries	Unsuccessful treatment (Low)[S12]	RR 2.23 (1.60 - 3.11)	0.67-3.33	7 (NR)	41%	2018
	Treatment failure (Low)[S12]	RR 0.75 (0.44-1.29)	0.32-2.40	7 (5,930)	55%	2018
SSA	Successful treatment (Critically low)[S5]	OR 0.87 (0.79 - 0.96)	0.75 - 1.26	6 (NR)	NR	2019
	Mortality (Critically low)[S5]	18% (14%-23%)	9%-31%	9 (NR)	91.1%	2019
	Cured (Critically low)[S5]	34% (22%-45%)	3%-60%	9 (NR)	98.9%	2019
Pulmonary TB + HIV						
Ethiopia	Prevalence (Moderate)[S7]	22.08% (14.36%-29.81%)	4.97%-28.60%	3 (1079)	89.9%	2019
TB meningitis + HIV						
LMIC	Prevalence (Critically low)[S13]	10.6% (4.2%–24.6%)	NR	NR (NR)	NR	2019
	Mortality (Critically low)[S13]	53.4% (42.4%–64.1%)	NR	7 (547)	2.1%	2019
TB lymphadenitis + HIV						
Africa	Prevalence (High)[S11]	52% (33%-71%)	6%-91%	14 (NR)	99.2%	2019
Ethiopia	Prevalence (High)[S11]	21% (12%-30%)	6%-67%	6 (NR)	92.9%	2019

Notes: SSA: Sub-Saharan Africa; LMIC: Low and middle-income countries; NA: Not applicable; RR: Risk ratio; NR: Not reported; OR: Odds ratio.

* Including only 7 high quality studies: 12.17% (95% CI 5.68%-26.11%)

† In the original review they considered this value low if it was <4

‡ Includes one study focused on children

§ Excluding one study focused on children: RR 1.12 (0.96-1.30, I2=69%)

¶ Quality rating represents the overall confidence in the results of the review, as assessed with the AMSTAR2 tool

TB and DM

Of the 14 reviews reporting data on TB and DM (>2,878,000 participants from 48 countries),[S4,S13–S17,S36–S42,S50] three focused on specific types of TB,[S14,S17,S50] and six reported at least one pooled outcome of interest (Supplementary Table 3).[S4,S14–S18]

For peer review only - http://bmjopen.bmj.com/site/about/guidelines.xhtml

One review[S17] reported the pooled prevalence separately for low-income countries (7.9%, 95%CI 4.9%-11.5%, 15 studies, 9,434 participants, critically low quality), lower-middle income countries (17.7%, 95%CI 15.1%-20.5%, 48 studies, 48,036 participants, critically low quality), and upper-middle income countries (14.4%, 95%CI 12.8%-16.0%, 75 studies, 1,994,027 participants, critically low quality). The same review also reported the prevalence of DM in people with TB in Africa (8.0%, 95%CI, 5.9%-10.4%, 119 studies, 474,944 participants, critically low quality, Table 3). Pooled prevalences in other continents were also reported, but were excluded from our review, as they included data from HICs. Other reviews reported prevalence estimates for sub-continental regions, ranging from 9% in SSA (low quality)[S14] to 21% in South Asia (low quality),[S4] as well as for multiple individual countries (Figure 2, Table 3).

One review[S16] reported an increased odds of mortality (OR 1.80, 95%CI 1.35-2.40, 34 studies, low quality) and treatment failure or death (OR 1.90, 95%CI 1.43-2.53, 22 studies, low quality) in people with TB and DM compared to people with only TB, in LMICs overall.

Table 3 also summarises the results of systematic reviews focused on (or reporting data for) specific types of TB (MDR-TB and PTB).

Table 3: Prevalence of diabetes mellitus and effect on outcomes in people with tuberculosis

**Region**	**Outcome (quality*)**	**Effect size (95% CI)**	**Range**	**Number of studies (number of participants)**	**I²**	**Year**
LMIC	Mortality (Low)[S16]	OR 1.80 (1.35-2.40)	0.45-29.22	34 (NR)	91.1%	2019
	Treatment failure or death (Low)[S16]	OR 1.90 (1.43-2.53)	0.73-11.75	22 (NR)	87.3%	2019
World (Low-income countries)	Prevalence (Critically low)[S17]	7.9% (4.9%-11.5%)	NR	15 (9,434)	96.8%	2019
World (Lower-Middle income countries)	Prevalence (Critically low)[S17]	17.7% (15.1%-20.5%)	NR	48 (48,036)	98.3%	2019
World (Upper-Middle income countries)	Prevalence (Critically low)[S17]	14.4% (12.8%-16.0%)	NR	75 (1,994,027)	99.9%	2019
Africa	Prevalence (Critically low)[S17]	8.0% (5.9%-10.4%)	1.9%-32.4%	119 (474,944)	99.8%	2019
Southeast Asia	Prevalence (Critically low)[S17]	19.0% (16.2%-21.9%)	5.1%-54.1%	30 (30,382)	97.0%	2019
South Asia	Prevalence (Low)[S4]	21% (18%-23%)	4%-66%	65 (NR)	98.28%	2021
SSA	Prevalence (Low)[S14]	9% (6%-12%)	2%-38%	16 (13,286)	97.48%	2019
Bangladesh	Prevalence (Critically low)[S17]	10.6% (7.2%-14.5%)	8.3%-12.8%	3 (3,010)	85.9%	2019
Benin	Prevalence (Critically low)[S17]	1.9% (0.2%-4.7%)	NA	1 (159)	NA	2019
Brazil	Prevalence (Critically low)[S17]	7.2% (6.3%-8.1%)	3.3%-33.1%	12 (1,726,436)	99.7%	2019
China	Prevalence (Critically low)[S17]	14.5% (10.5%-19.0%	2.7%-30.1%	14 (19,529)	98.4%	2019
Egypt	Prevalence (Critically low)[S17]	22.8% (15.2%-31.4%)	15.8%-27.7%	3 (578)	81.4%	2019
Ethiopia	Prevalence (Critically low)[S17]	18.8% (1.9%-47.1%)	8.3%-32.4%	2 (1,749)	99.2%	2019
Fiji	Prevalence (Critically low)[S17]	10.1% (4.4%-17.7%)	5.2-13.7	3 (1,139)	91.8%	2019
Georgia	Prevalence (Critically low)[S17]	12.4% (7.4%-18.5%)	NA	1 (137)	NA	2019
Guinea-Bissau	Prevalence (Critically low)[S17]	2.7% (0.3%-6.8%)	NA	1 (110)	NA	2019
Guyana	Prevalence (Critically low)[S17]	14.0% (7.8%-21.6%)	NA	1 (100)	NA	2019

For peer review only - http://bmjopen.bmj.com/site/about/guidelines.xhtml

India	Prevalence (Low)[S4]	22.0% (19.0%–25.0%)	NR	47 (NR)	97.92%	2021
	Mortality (Low)[S4]	OR 1.74 (1.21-2.51)	NR	5 (NR)	19.43%	2021
	Treatment failure (Low)[S4]	OR 1.65 (1.12-2.44)	NR	5 (NR)	49,63%	2021
	Recurrence (Low)[S4]	OR 0.53 (0.32-0.87)	NA	1 (NR)	NA	2021
	Cured (Low)[S4]	OR 0.32 (0.10-1.05)	NA	1 (NR)	NA	2021
Indonesia	Prevalence (Critically low)[S17]	14.8% (12.2%-17.7%)	NA	1 (634)	NA	2019
Iran	Prevalence (Critically low)[S17]	17.8% (12.5%-23.8%)	5.5%-40.0%	11 (3,134)	93.3%	2019
Kazakhstan	Prevalence (Critically low)[S17]	7.1% (5.1%-9.4%)	NA	1 (562)	NA	2019
Kiribati	Prevalence (Critically low)[S17]	36.7% (31.1%-42.5%)	NA	1 (275)	NA	2019
Libya	Prevalence (Critically low)[S17]	6.1% (3.5%-9.4%)	NA	1 (262)	NA	2019
Malaysia	Prevalence (Critically low)[S17]	26.9% (17.8%-37.0%)	15.4%-39.0%	5 (23,438)	98.1%	2019
Marshall Islands	Prevalence (Critically low)[S17]	45.2% (32.9%-57.7%)	NA	1 (62)	NA	2019
Mexico	Prevalence (Critically low)[S17]	30.8% (26.4%-35.3%)	19.3%-54.4%	10 (192,420)	97.9%	2019
Nepal	Prevalence (Low)[S4]	12.0% (4.0%–20.0%)	NR	4 (NR)	97.6%	2021
Nigeria	Prevalence (Critically low)[S17]	7.8% (4.4%-12.0%)	4.8%-12.0%	4 (9,821)	97.8%	2019
Pakistan	Prevalence (Low)[S4]	19.0% (11.0%–27.0%)	NR	10 (NR)	99.18%	2021
Peru	Prevalence (Critically low)[S17]	4.8% (1.7%-9.5%	2.5%-11.1%	4 (3,983)	96.8%	2019
Romania	Prevalence (Critically low)[S17]	18.4% (13.6%-23.7%)	NA	1 (228)	NA	2019
Senegal	Prevalence (Critically low)[S17]	4.9% (2.2%-8.5%)	3.8%-7.0%	2 (2,848)	75.1%	2019
South Africa	Prevalence (Critically low)[S17]	9.4% (7.6%-11.3%)	NA	1 (947)	NA	2019
Sri Lanka	Prevalence (Low)[S4]	24.0% (21.0%–27.0%)	NR	2 (NR)	NR	2021
Tanzania	Prevalence (Critically low)[S17]	8.5% (4.8%-13.0%)	2.6%-16.7%	7 (4,178)	95.1%	2019
Thailand	Prevalence (Critically low)[S17]	7.5% (6.2%-8.8%)	6.0%-16.3%	5 (17,862)	81.6%	2019
Tunisia	Prevalence (Critically low)[S17]	7.6% (5.9%-9.6%)	NA	1 (788)	NA	2019
Turkey	Prevalence (Critically low)[S17]	7.8% (6.8%-8.8%)	7.9%-8.6%	3 (2,773)	0%	2019
Uganda	Prevalence (Critically low)[S17]	7.3% (4.7%-10.3%)	5.4%-8.5%	2 (390)	9.9%	2019
Yemen	Prevalence (Critically low)[S17]	9.5% (6.0%-13.8%)	NA	1 (220)	NA	2019
Multidrug resistant TB + DM						
LMIC	Unsuccessful treatment (Low)[S12]	RR 0.90 (0.65-1.23)	0.23-0.98	3 (687)	19%	2018
Pulmonary TB + DM						
China	Prevalence (Critically low)[S15]	7.20% (6.01%-8.39%	2.08%-16.16%	22 (56,805)	NR	2013
	Retreatment (Critically low)[S18]	OR 2.05 (1.30-3.22)	NR	3 (499)	0%	2016
	Retreatment (Critically low)[S18]	aOR 3.38 (1.56-7.29)	NR	2 (NR)	75%	2016

Notes: LMIC: Low and middle-income countries; OR: Odds ratio; aOR: adjusted odds ratio; NR: Not reported; NA: Not applicable
[*] Quality rating represents the overall confidence in the results of the review, as assessed with the AMSTAR2 tool

TB and mental disorders

For peer review only - http://bmjopen.bmj.com/site/about/guidelines.xhtml

TB and mental disorders (pooled as a composite outcome)

We found one systematic review considering a composite outcome for mental disorders,[S21] as well as several other reviews looking at individual mental disorders such as depression, anxiety, and psychosis. The review[S21] that reported the effect of mental disorders (defined as a composite variable including depression, psychological distress, PTSD, or mental disorder) on unsuccessful treatment (a composite measure combining some or all of treatment failure, loss to follow-up, and death), loss to follow-up, and non-adherence, found no evidence of a significant increase in the odds of these outcomes in people with TB and mental disorders, compared to people with only TB (Table 4).

TB and depression

Of the four reviews reporting data on TB and depression (>21,770 participants from 33 countries),[S3,S19,S20,S44] three[S3,S20,S44] reported at least one pooled outcome of interest (Supplementary Table 3). One systematic review[S20] of 25 studies reported the prevalence of depression in people with TB in LMICs as 45.19% (95%CI 38.04%-52.55%, 25 studies, 4,903 participants, high quality). None of the included reviews reported this outcome at a continental, regional or country level (Table 4). One systematic review[S3] reported an increased odds of mortality (OR 2.85, 95%CI 1.52-5.36, 2 studies, 1,303 participants, critically low quality) and other adverse outcomes in people with TB and depression compared to people with only TB (Table 4).Table 4 summarises the results of systematic reviews focused on MDR-TB. According to these results, the prevalence of depression in people with MDR-TB is 52% (95%CI 38%-66%, 5 studies, high quality).[S20]

TB and anxiety

Of the two[S19,S44] reviews reporting data on TB and anxiety (>7,500 participants from 31 countries), only one,[S19] focused on MDR-TB, reported any pooled outcome of interest: the prevalence of anxiety overall (24%, 95%CI 2%-57%, 3 studies, 209 participants, critically low quality) and in the regions of Southeast Asia and the Americas (Table 4).

TB and psychosis

One systematic review (7518 participants from 17 countries), focused on MDR-TB, reported the prevalence of psychosis in Africa (12%, 95%CI 8%-17%, 5 studies, critically low quality) and in several subcontinental regions (Table 4).[S19]

Table 4: Prevalence and effect on outcomes in people with tuberculosis (TB) and mental disorders: as a composite outcome and separately for depression, anxiety, and psychosis.

Region	Outcome (quality‡)	Effect size (95% CI)	Range	Number of studies (number of participants)	I^2	Year
TB + Mental disorders (composite measure)*						
LMIC	Unsuccessful treatment† (Critically low)[S21]	OR 2.13 (0.85-5.37)	0.80-4.25	4 (1,196)	82%	2020
	Loss to follow-up (Critically low)[S21]	OR 1.90 (0.33-10.91)	0.88-5.33	2 (1,139)	78%	2020
	Non-adherence to treatment (Critically low)[S21]	OR 1.60 (0.84-3.02)	0.94-3.67	4 (10,851)	86%	2020

For peer review only - http://bmjopen.bmj.com/site/about/guidelines.xhtml

TB + Depression						
LMIC	Prevalence (High)[S20]	45.19% (38.04%-52.55%)	15.56%-80.00%)	25 (4,903)	96.28%	2020
	Prevalence in Women (High)[S20]	51.54% (40.34%–62.60%)	NR	17 (NR)	92.55%	2020
	Prevalence in Men (High)[S20]	45.25% (35.19%–55.71%)	NR	17 (NR)	95.09%	2020
	Mortality or loss to follow-up (Critically low)[S3]	OR 4.26 (2.33–7.79)	3.65-4.88	2 (1303)	0%	2020
	Mortality (Critically low)[S3]	OR 2.85 (1.52-5.36)	1.76-2.99	2 (973)	0%	2020
	Loss to follow up (Critically low)[S3]	OR 8.70 (4.95-9.09)	4.95-9.09	2 (973)	0%	2020
	Non-adherence to TB treatment (Critically low)[S3]	OR 1.38 (0.70-2.72)	0.92-3.67	3 (9,349)	94.36%	2020
Multidrug resistant TB + Depression						
LMIC	Prevalence (High)[S20]	52.34% (38.09%-66.22%)	NR	5 (NR)	92.55%	2020
Africa	Prevalence (Critically low)[S19]	16% (9%-24%)	NA	3 (NR)	NA	2018
The Americas Region	Prevalence (Critically low)[S19]	36% (23-50%)	NR	3 (NR)	NR	2018
European Region	Prevalence (Critically low)[S19]	11% (4%-21%)	NR	3 (NR)	NR	2018
Eastern Mediterranean Region	Prevalence (Critically low)[S19]	73% (64%-81%)	NR	2 (NR)	NR	2018
Western Pacific Region	Prevalence (Critically low)[S19]	5% (1%-12%)	NA	1 (NR)	NA	2018
Southeast Asia	Prevalence (Critically low)[S19]	22% (0%-60%)	NA	3 (NR)	NA	2018
Multidrug resistant TB + Anxiety						
LMIC	Prevalence (Critically low)[S19]	24% (2%-57%)	12%-56%	3 (209)	95%	2018
The Americas Region	Prevalence (Critically low)[S19]	14% (9%-21%)	NR	2 (NR)	NR	2018
Southeast Asia	Prevalence (Critically low)[S19]	56% (45%-66%)	NR	1 (NR)	NR	2018
Multidrug resistant TB + Psychosis						
Africa	Prevalence (Critically low)[S19]	12% (8%-17%)	NR	5 (NR)	NR	2018
The Americas Region	Prevalence (Critically low)[S19]	11% (7%-17%)	NR	2 (NR)	NR	2018
European Region	Prevalence (Critically low)[S19]	6% (0%-17%)	NR	2 (NR)	NR	2018
Eastern Mediterranean Region	Prevalence (Critically low)[S19]	7% (1%-17%)	NA	1 (NR)	NA	2018
Southeast Asia	Prevalence (Critically low)[S19]	10% (5%-17%)	NA	2 (NR)	NA	2018

Notes: LMIC: Low and middle-income countries; OR: Odds ratio; NR: Not reported; NA: Not applicable.
* Includes depression, psychological distress, PTSD, or mental disorder as a composite variable.
† Composite measure combining some or all of: treatment failure, loss to follow-up, and death.
‡ Quality rating represents the overall confidence in the results of the review, as assessed with the AMSTAR2 tool

TB and HCV

One systematic review estimated the prevalence of HCV in people with TB in Africa to be 11% (95%CI 1%-23%, 3 studies, 327 participants, I^2=93.9%, moderate quality).[S22]

Risk of cancer in people with TB

One systematic review[S23] reported the risk of different types of cancer in people with TB in upper-middle income countries, including lung cancer (RR 1.53, 95%CI 1.25-1.87, 9 studies, low quality), non-Hodgkin's

For peer review only - http://bmjopen.bmj.com/site/about/guidelines.xhtml

lymphoma (RR 1.70, 95%CI 1.13-2.56, 1 study, low quality) and leukaemia (RR 1.61, 95%CI 1.13-2.29, 1 study, low quality)(Table 5).

Risk of coronary heart disease in people with TB

One systematic review reported (in their abstract) an increased risk of coronary heart disease in people with TB in LMICs (RR 1.76, 95%CI 1.05–2.95)(Table 5).[S55]

Table 5: Risk of cancer and coronary heart disease in people with tuberculosis

Region	Outcome (quality*)	Effect size (95% CI)	Range	Number of studies (number of participants)	I^2	Year
Upper-Middle income countries	Lung cancer (Low)[S23]	RR 1.53 (1.25-1.87)	NR	9 (NR)	94.6%	2020
	non-Hodgkin's lymphoma (Low)[S23]	RR 1.70 (1.13-2.56)	NA	1 (NR)	NA	2020
	Leukaemia (Low)[S23]	RR 1.61 (1.13-2.29)	NA	1 (NR)	NA	2020
LMIC	Coronary heart disease † [S55]	RR 1.76 (1.05–2.95)	NA	4 (NR)	97%	2020

Notes: LMIC: Low and middle-income countries; RR: Risk ratio; NR: Not reported; NA: Not applicable.
* Quality rating represents the overall confidence in the results of the review, as assessed with the AMSTAR2 tool
† Data extracted from abstract only, as we could not obtain the full text article.

Subgroup analyses

Regarding our planned subgroup analyses, we could only find data stratified by gender reported for the prevalence of HIV in people with TB in China (Women: 0.6%, 95%CI 0.3%-1.1%, 9 studies, critically low quality; Men: 1.1% (95%CI 0.6%-2.0%; 9 studies, critically low quality)(Table 1)[S8] and the prevalence of depression in people with TB (Women: 51.54%, 95%CI 40.34%-62.60%, 17 studies, high quality; Men: 45.25%, 95%CI 35.19%–55.71%, 17 studies, high quality)(Table 4).[S20] We did not find any pooled results stratified by age.

DISCUSSION

In this first meta-review to identify and map out the co-occurrence of CCDs and NCDs in people with TB in LMICs. Although the geographical regions covered by the included reviews varied, we found that the most prevalent chronic conditions were depression, HIV, and DM. We also found some evidence that people with TB and these chronic conditions had significantly increased odds of adverse outcomes such as death and treatment failure. No systematic review pooled the prevalence of two or more additional chronic conditions in people with TB.

Our findings offer an overview of TB multimorbidity to see comorbid conditions in relation to each other. For instance, despite the known synergistic relationship between TB and HIV,[12] our review suggests that the negative impact of HIV on TB treatment outcomes is less severe than the impact of depression, which not only had higher odds of adverse outcomes, but also was more prevalent among people with TB. This apparent smaller impact of HIV than depression in people with TB could partially be explained by the disparity – in attention and resources – between HIV and depression, and illustrates how an integrated approach, such as the

For peer review only - http://bmjopen.bmj.com/site/about/guidelines.xhtml

one received by at least some patients with TB and HIV, could reduce the negative impact of other chronic conditions, such as depression, in TB patients. This also illustrates how the results of our review could be used when planning for new services. Moreover, it highlights the importance of screening for mental health in areas where mental health services need improvement.[26,27]

Our meta-review highlights the many gaps in the literature on TB multimorbidity in LMIC. For example, while the meta-analysis of the prevalence of TB and depression included 25 studies,[28] the meta-analysis for treatment outcomes in this group included only two studies,[16] reflecting the lack of evidence for the impact of TB multimorbidity on TB treatment outcomes. Data stratified by gender or age was also minimal, which is particularly important when women might have different healthcare seeking behaviours and limited voice in decision making. In addition to the gaps in the literature with regards to primary studies, our meta-review also highlights the lack of systematic reviews focused on people with TB and more than one additional chronic condition, which is an increasingly likely scenario as the prevalence of NCDs in LMICs grows.[2] Considering the potential multiple-way synergies between multiple chronic conditions, a systematic review of the literature on this topic is sorely needed. This evidence gap is addressed in a complementary review by our group.[29]

Strengths

Our review has several strengths, such as an extensive search strategy, including databases of grey literature and protocols. Considering that PROSPERO is the main registry for systematic reviews and our efforts to contact authors of potentially relevant protocols, we are confident in the coverage of our search strategy. Another strength of our review is our focus on LMICs, making sure that data from HICs was not included, as the differences in risk factors, resources and treatment opportunities would make the results less applicable to LMICs.

Limitations

Our review has several limitations as well. First, most of the meta-analyses had very high heterogeneity and should therefore be interpreted with caution. Second, more than half of the studies summarised in our results had low or critically low quality. Third, despite the large number of systematic reviews identified in our review, our focus on LMIC excluded many results reported in them. Finally, we found little evidence regarding the burden of TB multimorbidity, which was one of the goals of this review. This highlights gaps in the body of evidence of systematic reviews, suggesting new future lines of research.

Conclusion

Given the fact that multimorbidity is common in LMICs[30,31] and is associated with a wide range of adverse outcomes for the individual, family, and society, and poses challenges for healthcare systems, particularly in LMICs, our results are important.[32,33] TB multimorbidity appears to be common and to have additional burdensome impact, deserving urgent attention.[13,34] Research is needed to identify early at-risk populations and ultimately prevent the onset of TB multimorbidity and to develop effective treatments and clinical pathways to care for this heterogeneous and burdensome group of people.[13] The high prevalence of TB multimorbidity in LMICs is a triple challenge, as these regions already have the highest (and growing) number of people with multimorbidity generally, the highest levels of TB, and health and social care systems which are stretched/sparse

For peer review only - http://bmjopen.bmj.com/site/about/guidelines.xhtml

and unable to deal with these complexities.[13] Thus, urgent research is needed to better address this clearly prevalent, burdensome, and important issue.

Acknowledgments

This review is part of the TB Multimorbidity Network project (https://www.impactsouthasia.com/tbmm/), which is funded by the Medical Research Council (MRC Grant reference MC_PC_MR/T037806/1).

Competing interests

All authors declare no competing interests

Ethics statement

Ethical approval was not needed as this was a systematic review of published literature.

Funding

This work was supported by Medical Research Council UK, grant number MC_PC_MR/T037806/1.

Contributions

NS, BS, KS, and HE conceptualised the study; AJ, ER, AE, YL, RH, HE, KS, BS, and NS contributed to the design of the study; AJ, ER and NS contributed to the titles and abstracts screening; AJ, ER, and SA contributed to the full text screening and data extraction, ER and SA contributed to the quality assessment; AJ summarised the results and wrote the first draft of the report with input from BS and NS; ER, SA, AE, YL, and KS contributed to the interpretation of the results and revised the manuscript. All authors had full access to all the data in the study and had final responsibility for the decision to submit for publication.

Data sharing

All the collected data is in the public domain.

Figure legends

Figure 1: Flow diagram of the search results and screening process.

Figure 2: Prevalence of diabetes mellitus in people with TB in each country

For peer review only

References

1 Foguet-Boreu Q, Violan C, Roso-Llorach A, *et al.* Impact of multimorbidity: acute morbidity, area of residency and use of health services across the life span in a region of south Europe. *BMC Fam Pract* 2014;**15**:55. doi:10.1186/1471-2296-15-55

2 France EF, Wyke S, Gunn JM, *et al.* Multimorbidity in primary care: a systematic review of prospective cohort studies. *Br J Gen Pract* 2012;**62**:e297–307. doi:10.3399/bjgp12X636146

3 Roche S, Vries E de. Multimorbidity in a large district hospital: A descriptive cross-sectional study. *S Afr Med J* 2017;**107**:1110–5.

4 World Health Organization. HIV/AIDS. https://www.who.int/news-room/fact-sheets/detail/hiv-aids (accessed 22 Sep 2021).

5 Murray CJL, Vos T, Lozano R, *et al.* Disability-adjusted life years (DALYs) for 291 diseases and injuries in 21 regions, 1990–2010: a systematic analysis for the Global Burden of Disease Study 2010. *The Lancet* 2012;**380**:2197–223. doi:10.1016/S0140-6736(12)61689-4

6 GBD Compare | IHME Viz Hub. http://vizhub.healthdata.org/gbd-compare (accessed 22 Sep 2021).

7 World Health Organization. *Global tuberculosis report*. Geneva: : World Health Organization 2020. https://apps.who.int/iris/handle/10665/336069 (accessed 4 Oct 2021).

8 Roth GA, Abate D, Abate KH, *et al.* Global, regional, and national age-sex-specific mortality for 282 causes of death in 195 countries and territories, 1980–2017: a systematic analysis for the Global Burden of Disease Study 2017. *The Lancet* 2018;**392**:1736–88. doi:10.1016/S0140-6736(18)32203-7

9 Marais BJ, Lönnroth K, Lawn SD, *et al.* Tuberculosis comorbidity with communicable and non-communicable diseases: integrating health services and control efforts. *Lancet Infect Dis* 2013;**13**:436–48. doi:10.1016/S1473-3099(13)70015-X

10 Oh KH, Choi H, Kim EJ, *et al.* Depression and risk of tuberculosis: a nationwide population-based cohort study. *Int J Tuberc Lung Dis* 2017;**21**:804–9. doi:10.5588/ijtld.17.0038

11 Niazi AK, Kalra S. Diabetes and tuberculosis: a review of the role of optimal glycemic control. *J Diabetes Metab Disord* 2012;**11**:28. doi:10.1186/2251-6581-11-28

12 Kwan CK, Ernst JD. HIV and Tuberculosis: a Deadly Human Syndemic. *Clin Microbiol Rev* 2011;**24**:351–76. doi:10.1128/CMR.00042-10

13 Siddiqi K, Stubbs B, Lin Y, *et al.* TB multimorbidity: a global health challenge demanding urgent attention. *Int J Tuberc Lung Dis* 2021;**25**:87–90. doi:10.5588/ijtld.20.0751

14 Huddart S, Svadzian A, Nafade V, *et al.* Tuberculosis case fatality in India: a systematic review and meta-analysis. *BMJ Glob Health* 2020;**5**:e002080. doi:10.1136/bmjgh-2019-002080

15 Eshetie S, Gizachew M, Alebel A, *et al.* Tuberculosis treatment outcomes in Ethiopia from 2003 to 2016, and impact of HIV co-infection and prior drug exposure: A systematic

For peer review only - http://bmjopen.bmj.com/site/about/guidelines.xhtml

review and meta-analysis. *PloS One* 2018;**13**:e0194675. doi:10.1371/journal.pone.0194675

16 Ruiz-Grosso P, Cachay R, de la Flor A, *et al.* Association between tuberculosis and depression on negative outcomes of tuberculosis treatment: A systematic review and meta-analysis. *PloS One* 2020;**15**:e0227472. doi:10.1371/journal.pone.0227472

17 Gautam S, Shrestha N, Mahato S, *et al.* Diabetes among tuberculosis patients and its impact on tuberculosis treatment in South Asia: a systematic review and meta-analysis. *Sci Rep* 2021;**11**:2113. doi:10.1038/s41598-021-81057-2

18 Moher D, Shamseer L, Clarke M, *et al.* Preferred reporting items for systematic review and meta-analysis protocols (PRISMA-P) 2015 statement. *Syst Rev* 2015;**4**:1.

19 Bernell S, Howard SW. Use Your Words Carefully: What Is a Chronic Disease? *Front Public Health* 2016;**4**. doi:10.3389/fpubh.2016.00159

20 Laserson KF, Thorpe LE, Leimane V, *et al.* Speaking the same language: treatment outcome definitions for multidrug-resistant tuberculosis. *Int J Tuberc Lung Dis* 2005;**9**:640–5.

21 World Health Organization, others. Definitions and reporting framework for tuberculosis–2013 revision: updated December 2014 and January 2020. World Health Organization 2013.

22 Shea BJ, Reeves BC, Wells G, *et al.* AMSTAR 2: a critical appraisal tool for systematic reviews that include randomised or non-randomised studies of healthcare interventions, or both. *BMJ* 2017;**358**. doi:10.1136/bmj.j4008

23 Wongtrakul W, Charoenngam N, Ungprasert P. Tuberculosis and risk of coronary heart disease: A systematic review and meta-analysis. *Indian J Tuberc* 2020;**67**:182–8. doi:10.1016/j.ijtb.2020.01.008

24 Pang P., Sun C., Duan W., *et al.* A review on global head and neck tuberculosis cases from 980 papers and 5881 patients. *Int J Oral Maxillofac Surg* 2019;**48**:182. doi:10.1016/j.ijom.2019.03.563

25 Yixiang ZHENG, Shujuan MA, Deming TAN, *et al.* A meta-analysis of liver lesions in hepatitis B patients undergoing anti-tuberculosis therapy. *Chin J Hepatol* 2014;:585–9.

26 Keynejad RC, Dua T, Barbui C, *et al.* WHO Mental Health Gap Action Programme (mhGAP) Intervention Guide: a systematic review of evidence from low and middle-income countries. *Evid Based Ment Health* 2018;**21**:30–4.

27 Eaton J, McCay L, Semrau M, *et al.* Scale up of services for mental health in low-income and middle-income countries. *The Lancet* 2011;**378**:1592–603.

28 Duko B, Bedaso A, Ayano G. The prevalence of depression among patients with tuberculosis: a systematic review and meta-analysis. *Ann Gen Psychiatry* 2020;**19**:30. doi:10.1186/s12991-020-00281-8

29 Alexander Jarde BS Ruimin Ma, Eugenia Romano, Helen Elsey, Najma Siddiqi, Kamran Siddiqi. Prevalence, clusters and burden of multimorbidity in people with tuberculosis: a systematic review and meta-analysis.

For peer review only - http://bmjopen.bmj.com/site/about/guidelines.xhtml

30 Vancampfort D, Koyanagi A, Ward PB, *et al.* Perceived Stress and Its Relationship With Chronic Medical Conditions and Multimorbidity Among 229,293 Community-Dwelling Adults in 44 Low- and Middle-Income Countries. *Am J Epidemiol* 2017;**186**:979–89. doi:10.1093/aje/kwx159

31 Vancampfort D, Koyanagi A, Ward PB, *et al.* Chronic physical conditions, multimorbidity and physical activity across 46 low- and middle-income countries. *Int J Behav Nutr Phys Act* 2017;**14**:6. doi:10.1186/s12966-017-0463-5

32 Vancampfort D, Koyanagi A, Hallgren M, *et al.* The relationship between chronic physical conditions, multimorbidity and anxiety in the general population: A global perspective across 42 countries. *Gen Hosp Psychiatry* 2017;**45**:1–6. doi:10.1016/j.genhosppsych.2016.11.002

33 Jacob L, Oh H, Shin JI, *et al.* Informal Caregiving, Chronic Physical Conditions, and Physical Multimorbidity in 48 Low- and Middle-Income Countries. *J Gerontol A Biol Sci Med Sci* 2020;**75**:1572–8. doi:10.1093/gerona/glaa017

34 Stubbs B, Siddiqi K, Elsey H, *et al.* Tuberculosis and Non-Communicable Disease Multimorbidity: An Analysis of the World Health Survey in 48 Low- and Middle-Income Countries. *Int J Environ Res Public Health* 2021;**18**:2439. doi:10.3390/ijerph18052439

For peer review only

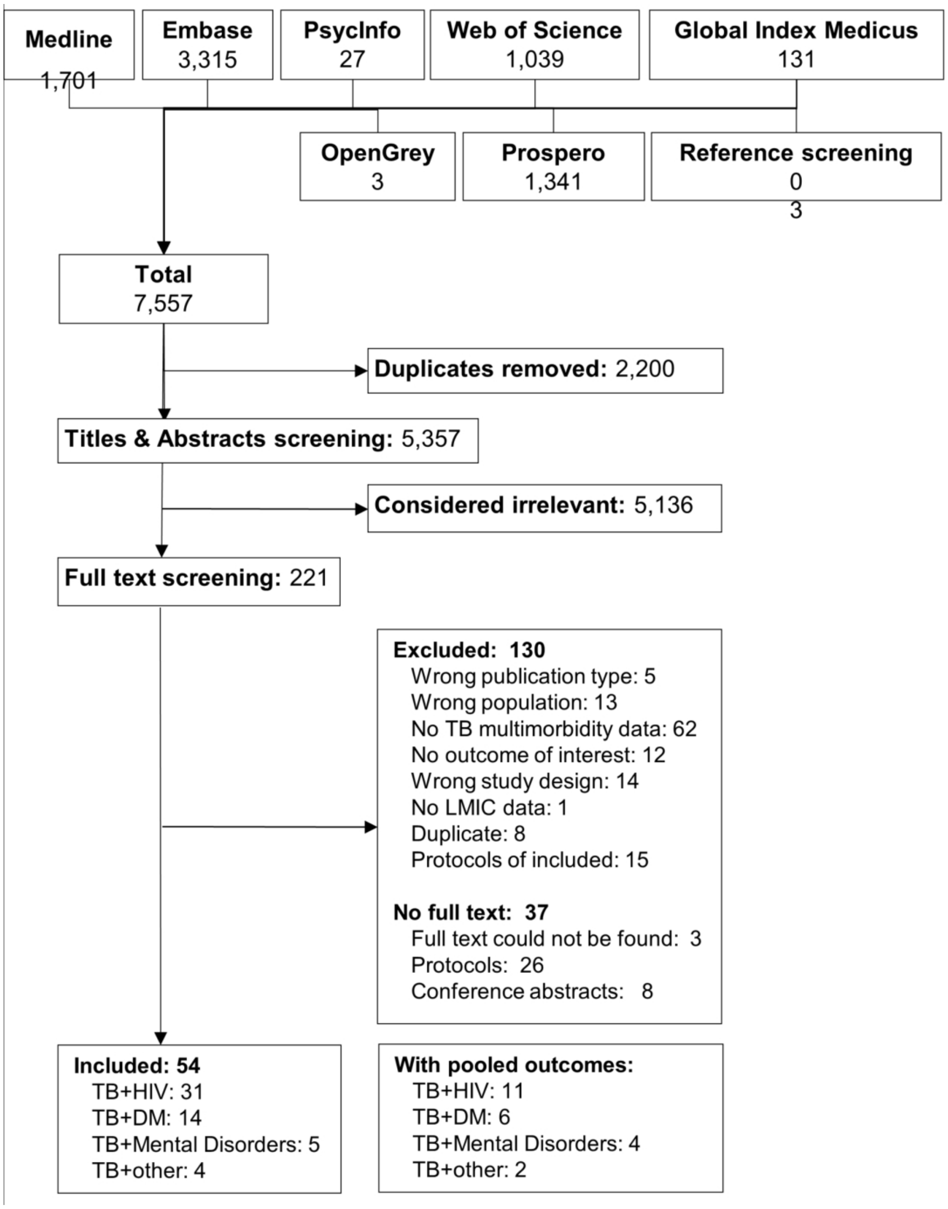

Flow diagram of the search results and screening process.

157x194mm (300 x 300 DPI)

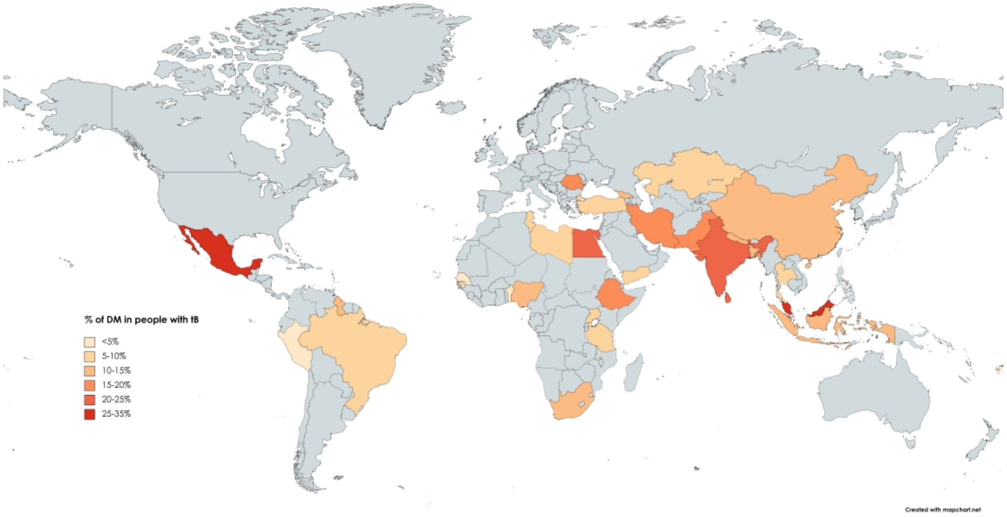

Prevalence of diabetes mellitus in people with TB in each country

546x288mm (300 x 300 DPI)

APPENDIX 1: References of included studies

S1 Huddart S, Svadzian A, Nafade V, *et al.* Tuberculosis case fatality in India: a systematic review and meta-analysis. *BMJ Glob Health* 2020;**5**:e002080. doi:10.1136/bmjgh-2019-002080

S2 Eshetie S, Gizachew M, Alebel A, *et al.* Tuberculosis treatment outcomes in Ethiopia from 2003 to 2016, and impact of HIV co-infection and prior drug exposure: A systematic review and meta-analysis. *PloS One* 2018;**13**:e0194675. doi:10.1371/journal.pone.0194675

S3 Ruiz-Grosso P, Cachay R, de la Flor A, *et al.* Association between tuberculosis and depression on negative outcomes of tuberculosis treatment: A systematic review and meta-analysis. *PloS One* 2020;**15**:e0227472. doi:10.1371/journal.pone.0227472

S4 Gautam S, Shrestha N, Mahato S, *et al.* Diabetes among tuberculosis patients and its impact on tuberculosis treatment in South Asia: a systematic review and meta-analysis. *Sci Rep* 2021;**11**:2113. doi:10.1038/s41598-021-81057-2

S5 Chem ED, Van Hout MC, Hope V. Treatment outcomes and antiretroviral uptake in multidrug-resistant tuberculosis and HIV co-infected patients in Sub Saharan Africa: a systematic review and meta-analysis. *BMC Infect Dis* 2019;**19**:723. doi:10.1186/s12879-019-4317-4

S6 Edessa D, Sisay M, Dessie Y. Unfavorable outcomes to second-line tuberculosis therapy among HIV-infected versus HIV-uninfected patients in sub-Saharan Africa: A systematic review and meta-analysis. *PloS One* 2020;**15**:e0237534. doi:10.1371/journal.pone.0237534

S7 Endalamaw A, Ambachew S, Geremew D, *et al.* HIV infection and unknown HIV status among tuberculosis patients in Ethiopia: a systematic review and meta-analysis. *Int J Tuberc Lung Dis Off J Int Union Tuberc Lung Dis* 2019;**23**:187–94. doi:10.5588/ijtld.18.0363

S8 Gao L, Zhou F, Li X, *et al.* HIV/TB co-infection in mainland China: a meta-analysis. *PloS One* 2010;**5**:e10736. doi:10.1371/journal.pone.0010736

S9 Gao J, Zheng P, Fu H. Prevalence of TB/HIV co-infection in countries except China: a systematic review and meta-analysis. *PloS One* 2013;**8**:e64915. doi:10.1371/journal.pone.0064915

S10 Gelaw YA, Williams G, Soares Magalhaes RJ, *et al.* HIV Prevalence Among Tuberculosis Patients in Sub-Saharan Africa: A Systematic Review and Meta-analysis. *AIDS Behav* 2019;**23**:1561–75. doi:10.1007/s10461-018-02386-4

S11 Mekonnen D, Derbie A, Abeje A, *et al.* Epidemiology of tuberculous lymphadenitis in Africa: A systematic review and meta-analysis. *PloS One* 2019;**14**:e0215647. doi:10.1371/journal.pone.0215647

S12 Samuels JP, Sood A, Campbell JR, *et al.* Comorbidities and treatment outcomes in multidrug resistant tuberculosis: a systematic review and meta-analysis. *Sci Rep* 2018;**8**:4980. doi:10.1038/s41598-018-23344-z

S13 Wang M-G, Luo L, Zhang Y, *et al.* Treatment outcomes of tuberculous meningitis in adults: a systematic review and meta-analysis. *BMC Pulm Med* 2019;**19**:200. doi:10.1186/s12890-019-0966-8

S14 Alebel A, Wondemagegn AT, Tesema C, *et al.* Prevalence of diabetes mellitus among tuberculosis patients in Sub-Saharan Africa: a systematic review and meta-analysis of observational studies. *BMC Infect Dis* 2019;**19**:254. doi:10.1186/s12879-019-3892-8

S15 Chen H, Liu M, Gu F. [Meta-analysis on the co-morbidity rate between tuberculosis and diabetes mellitus in China]. *Zhonghua Liu Xing Bing Xue Za Zhi Zhonghua Liuxingbingxue Zazhi* 2013;**34**:1128–33.

For peer review only - http://bmjopen.bmj.com/site/about/guidelines.xhtml

S16 Huangfu P, Ugarte-Gil C, Golub J, *et al.* The effects of diabetes on tuberculosis treatment outcomes: an updated systematic review and meta-analysis. *Int J Tuberc Lung Dis Off J Int Union Tuberc Lung Dis* 2019;**23**:783–96. doi:10.5588/ijtld.18.0433

S17 Noubiap JJ, Nansseu JR, Nyaga UF, *et al.* Global prevalence of diabetes in active tuberculosis: a systematic review and meta-analysis of data from 2.3 million patients with tuberculosis. *Lancet Glob Health* 2019;**7**:e448–60. doi:10.1016/S2214-109X(18)30487-X

S18 S.H Y, M.-L D, D J, *et al.* Risk factors of retreatment pulmonary tuberculosis patients with unfavorable treatment outcome in China: A meta-analysis. *Chin J Evid-Based Med* 2016;**16**:795–801.

S19 Alene KA, Clements ACA, McBryde ES, *et al.* Mental health disorders, social stressors, and health-related quality of life in patients with multidrug-resistant tuberculosis: A systematic review and meta-analysis. *J Infect* 2018;**77**:357–67. doi:10.1016/j.jinf.2018.07.007

S20 Duko B, Bedaso A, Ayano G. The prevalence of depression among patients with tuberculosis: a systematic review and meta-analysis. *Ann Gen Psychiatry* 2020;**19**:30. doi:10.1186/s12991-020-00281-8

S21 Ga Eun Lee JS Jerome Galea, Sanghyuk Shin, Annika Sweetland, Elizabeth Magill. Impact of mental illness on active tuberculosis (TB) treatment outcome: a systematic review and meta-analysis.

S22 Behzadifar M, Heydarvand S, Behzadifar M, *et al.* Prevalence of Hepatitis C Virus in Tuberculosis Patients: A Systematic Review and Meta-Analysis. *Ethiop J Health Sci* 2019;**29**:945–56. doi:10.4314/ejhs.v29i1.17

S23 C.Y L, H.-L H, M.M R, *et al.* Cancer incidence attributable to tuberculosis in 2015: Global, regional, and national estimates. *BMC Cancer* 2020;**20**:412.

S24 Arega B, Minda A, Mengistu G, *et al.* Unknown HIV status and the TB/HIV collaborative control program in Ethiopia: systematic review and meta-analysis. *BMC Public Health* 2020;**20**:1021. doi:10.1186/s12889-020-09117-2

S25 Bastos SH, Taminato M, Fernandes H, *et al.* Sociodemographic and health profile of TB/HIV co-infection in Brazil: a systematic review. *Rev Bras Enferm* 2019;**72**:1389–96. doi:10.1590/0034-7167-2018-0285

S26 Lukoye D, Ssengooba W, Musisi K, *et al.* Variation and risk factors of drug resistant tuberculosis in sub-Saharan Africa: a systematic review and meta-analysis. *BMC Public Health* 2015;**15**:291. doi:10.1186/s12889-015-1614-8

S27 McMurry HS, Mendenhall E, Rajendrakumar A, *et al.* Coprevalence of type 2 diabetes mellitus and tuberculosis in low-income and middle-income countries: A systematic review. *Diabetes Metab Res Rev* 2019;**35**:e3066. doi:10.1002/dmrr.3066

S28 Pourakbari B, Mamishi S, Banar M, *et al.* Prevalence of TB/ HIV co-infection in Iran: a systematic review and meta-analysis. *Ann Ig Med Prev E Comunita* 2019;**31**:333–48. doi:10.7416/ai.2019.2295

S29 Rajendran M, Zaki RA, Aghamohammadi N. Contributing risk factors towards the prevalence of multidrug-resistant tuberculosis in Malaysia: A systematic review. *Tuberc Edinb Scotl* 2020;**122**:101925. doi:10.1016/j.tube.2020.101925

S30 Reddy EA, Shaw AV, Crump JA. Community-acquired bloodstream infections in Africa: a systematic review and meta-analysis. *Lancet Infect Dis* 2010;**10**:417–32. doi:10.1016/S1473-3099(10)70072-4

S31 Seid MA, Ayalew MB, Muche EA, *et al.* Drug-susceptible tuberculosis treatment success and associated factors in Ethiopia from 2005 to 2017: a systematic review and meta-analysis. *BMJ Open* 2018;**8**:e022111. doi:10.1136/bmjopen-2018-022111

For peer review only

S32 Straetemans M, Glaziou P, Bierrenbach AL, *et al.* Assessing tuberculosis case fatality ratio: a meta-analysis. *PloS One* 2011;**6**:e20755. doi:10.1371/journal.pone.0020755

S33 Tesfaye B, Alebel A, Gebrie A, *et al.* The twin epidemics: Prevalence of TB/HIV co-infection and its associated factors in Ethiopia; A systematic review and meta-analysis. *PloS One* 2018;**13**:e0203986. doi:10.1371/journal.pone.0203986

S34 Teweldemedhin M, Asres N, Gebreyesus H, *et al.* Tuberculosis-Human Immunodeficiency Virus (HIV) co-infection in Ethiopia: a systematic review and meta-analysis. *BMC Infect Dis* 2018;**18**:676. doi:10.1186/s12879-018-3604-9

S35 Uchida S, Komiya K, Honjo K, *et al.* A mini systematic review of prognostic factors in elderly patients with tuberculosis. *Respir Investig* 2019;**57**:207–12. doi:10.1016/j.resinv.2018.12.004

S36 Waitt CJ, Squire SB. A systematic review of risk factors for death in adults during and after tuberculosis treatment. *Int J Tuberc Lung Dis Off J Int Union Tuberc Lung Dis* 2011;**15**:871–85. doi:10.5588/ijtld.10.0352

S37 Baker MA, Harries AD, Jeon CY, *et al.* The impact of diabetes on tuberculosis treatment outcomes: a systematic review. *BMC Med* 2011;**9**:81. doi:10.1186/1741-7015-9-81

S38 Han X, Wang Q, Wang Y, *et al.* The impact of diabetes on tuberculosis treatment outcomes: evidence based on a cumulative meta-analysis. *Int J DIABETES Dev Ctries* 2016;**36**:490–507. doi:10.1007/s13410-016-0514-5

S39 Huang D, Wang Y, Wang Y, *et al.* The impact of diabetes mellitus on drug resistance in patients with newly diagnosed tuberculosis: a systematic review and meta-analysis. *Ann Palliat Med* 2020;**9**:152–62. doi:10.21037/apm.2020.02.16

S40 Jeon CY, Harries AD, Baker MA, *et al.* Bi-directional screening for tuberculosis and diabetes: a systematic review. *Trop Med Int Health TM IH* 2010;**15**:1300–14. doi:10.1111/j.1365-3156.2010.02632.x

S41 Lutfiana NC, van Boven JFM, Masoom Zubair MA, *et al.* Diabetes mellitus comorbidity in patients enrolled in tuberculosis drug efficacy trials around the world: A systematic review. *Br J Clin Pharmacol* 2019;**85**:1407–17. doi:10.1111/bcp.13935

S42 Tegegne BS, Mengesha MM, Teferra AA, *et al.* Association between diabetes mellitus and multi-drug-resistant tuberculosis: evidence from a systematic review and meta-analysis. *Syst Rev* 2018;**7**:161. doi:10.1186/s13643-018-0828-0

S43 Workneh MH, Bjune GA, Yimer SA. Prevalence and associated factors of tuberculosis and diabetes mellitus comorbidity: A systematic review. *PloS One* 2017;**12**:e0175925. doi:10.1371/journal.pone.0175925

S44 A JVR, A D, R C, *et al.* Comorbidities between tuberculosis and common mental disorders: A scoping review of epidemiological patterns and person-centred care interventions from low-to-middle income and BRICS countries. *Infect Dis Poverty* 2020;**9**:4.

S45 Basham CA, Smith SJ, Romanowski K, *et al.* Cardiovascular morbidity and mortality among persons diagnosed with tuberculosis: A systematic review and meta-analysis. *PloS One* 2020;**15**:e0235821. doi:10.1371/journal.pone.0235821

S46 Rehm J, Samokhvalov AV, Neuman MG, *et al.* The association between alcohol use, alcohol use disorders and tuberculosis (TB). A systematic review. *BMC Public Health* 2009;**9**:450. doi:10.1186/1471-2458-9-450

S47 Sotgiu G, Ferrara G, Matteelli A, *et al.* Epidemiology and clinical management of XDR-TB: a systematic review by TBNET. *Eur Respir J* 2009;**33**:871–81. doi:10.1183/09031936.00168008

For peer review only

S48 Bisson GP, Bastos M, Campbell JR, *et al.* Mortality in adults with multidrug-resistant tuberculosis and HIV by antiretroviral therapy and tuberculosis drug use: an individual patient data meta-analysis. *Lancet Lond Engl* 2020;**396**:402–11. doi:10.1016/S0140-6736(20)31316-7

S49 Mesfin YM, Hailemariam D, Biadgilign S, *et al.* Association between HIV/AIDS and multi-drug resistance tuberculosis: a systematic review and meta-analysis. *PloS One* 2014;**9**:e82235. doi:10.1371/journal.pone.0082235

S50 Wu S, Zhang Y, Sun F, *et al.* Adverse Events Associated With the Treatment of Multidrug-Resistant Tuberculosis: A Systematic Review and Meta-analysis. *Am J Ther* 2016;**23**:e521-30. doi:10.1097/01.mjt.0000433951.09030.5a

S51 de Almeida CPB, Ziegelmann PK, Couban R, *et al.* Predictors of In-Hospital Mortality among Patients with Pulmonary Tuberculosis: A Systematic Review and Meta-analysis. *Sci Rep* 2018;**8**:7230. doi:10.1038/s41598-018-25409-5

S52 Pormohammad A, Nasiri MJ, Riahi SM, *et al.* Human immunodeficiency virus in patients with tuberculous meningitis: systematic review and meta-analysis. *Trop Med Int Health TM IH* 2018;**23**:589–95. doi:10.1111/tmi.13059

S53 Purmohamad A, Azimi T, Nasiri MJ, *et al.* HIV-Tuberculous Meningitis Co-infection: A Systematic Review and Meta-analysis. *Curr Pharm Biotechnol* Published Online First: 2020. doi:10.2174/1389201021666200730143906

S54 Alemu A, Bitew ZW, Worku T. Poor treatment outcome and its predictors among drug-resistant tuberculosis patients in Ethiopia: A systematic review and meta-analysis. *Int J Infect Dis IJID Off Publ Int Soc Infect Dis* 2020;**98**:420–39. doi:10.1016/j.ijid.2020.05.087

S55 Wongtrakul W, Charoenngam N, Ungprasert P. Tuberculosis and risk of coronary heart disease: A systematic review and meta-analysis. *Indian J Tuberc* 2020;**67**:182–8. doi:10.1016/j.ijtb.2020.01.008

For peer review only

Medline (October 23rd 2020)

# ▲	Searches	Results
1	exp Noncommunicable Diseases/ or ((Non-communicable or Noncommunicable or Non-infectious) adj (disease* or condition* or illness*)).mp.	11685
2	exp Chronic Disease/ or ((chronic or long-term) adj (disease* or condition* or illness*)).mp.	343000
3	exp Heart Diseases/ or (heart adj (disease* or disorder* or failure)).mp. or (cardiac adj (disease* or disorder* or failure)).mp.	1252363
4	exp Cardiovascular Diseases/ or (cardiovascular adj (disease* or disorder* or failure)).mp.	2482639
5	exp Coronary Disease/ or (coronary adj (disease* or disorder* or failure)).mp.	224638
6	exp Cerebrovascular Disorders/ or (cerebrovascular adj (disease* or disorder* or insufficienc* or occlusion*)).mp. or (vascular adj (disease* or disorder*)).mp. or (carotid* adj (disease* or disorder*)).mp.	460609
7	exp Peripheral Arterial Disease/ or (arter* adj (disease* or disorder*)).mp.	172699
8	exp Rheumatic Heart Disease/ or exp Heart Defects, Congenital/ or (heart adj3 (malform* or defect* or congeni*)).mp.	182266
9	exp Venous Thrombosis/ or ((deep vein or deep venous) adj thrombos*).mp. or phlebothrombos*.mp.	68358
10	exp Pulmonary Embolism/ or (pulmonar* adj (thromboembolism* or embolism* or disease* or disorder*)).mp.	138141
11	exp Stroke/ or stroke.mp.	320701
12	exp Neoplasms/ or Cancer*.mp. or neoplas*.mp. or tumor*.mp.	4416189
13	exp Lung Diseases/ or exp Respiratory Tract Diseases/ or exp Lung Diseases, Obstructive/ or ((lung* or respiratory or pulmonar* or airflow or airway) adj2 (disease* or obstruct* or hypersensitiv*)).mp. or exp Asthma/ or asthma*.mp. or exp Pulmonary Disease, Chronic Obstructive/ or exp Respiratory Hypersensitivity/	1474479
14	exp Diabetes Mellitus/ or diabet*.mp.	710026
15	exp Autoimmune Diseases/ or ((autoimmun* or auto immun* or autoaggress* or auto aggress*) adj (disorder* or disease*)).mp.	516672
16	exp Metabolic Syndrome/ or exp Metabolic Diseases/ or ((metabolic or insulin resistance) adj (disorder* or disease* or syndrome*)).mp.	1067250
17	exp Obesity/ or obes*.mp.	365459
18	exp Osteoporosis/ or osteoporo*.mp. or bone loss.mp. or exp osteolysis/ or osteolysis.mp. or bone resorption.mp.	153480

19	exp Parkinson disease/ or parkinson*.mp. or paralysis agitans.mp.	131499
20	exp Arthritis/ or arthriti*.mp. or polyarthriti*.mp. or rheumarthriti*.mp.	321229
21	exp Kidney Diseases/ or (kidney adj (disease* or disorder*)).mp.	544657
22	exp Liver Diseases/ or (liver adj (disease* or disorder* or dysfunction*)).mp.	591670
23	exp Hypertension/ or high blood pressure*.mp. or hypertens*.mp.	522886
24	exp Hyperlipidemias/ or hyperlipem*.mp. or hyperlipidem*.mp. or lipem*.mp. or lipidem*.mp.	84750
25	exp Hypercholesterolemia/ or ((high* or elevat*) adj cholesterol*).mp. or hypercholesterem*.mp. or hypercholesterolem*.mp.	50873
26	exp Hypertriglyceridemia/ or hypertriglyceridem*.mp.	15254
27	exp Thyroid Diseases/ or (thyroid adj (disease* or disorder*)).mp. or exp Hyperthyroidism/ or hyperthyroid*.mp. or exp Hypothyroidism/ or hypothyroid*.mp. or ((thyroid-stimulating hormone* or tsh) adj deficien*).mp.	166497
28	exp Motor Neuron Disease/ or motor neuron* disease*.mp. or lateral scleros*.mp. or motor system disease*.mp.	38409
29	exp Multiple Sclerosis/ or multiple sclerosis.mp. or disseminated sclerosis.mp.	84720
30	exp Emphysema/ or emphysema*.mp.	38522
31	exp Bronchitis/ or bronchit*.mp.	41201
32	1 or 2 or 3 or 4 or 5 or 6 or 7 or 8 or 9 or 10 or 11 or 12 or 13 or 14 or 15 or 16 or 17 or 18 or 19 or 20 or 21 or 22 or 23 or 24 or 25 or 26 or 27 or 28 or 29 or 30 or 31	10292435
33	exp Mental Disorders/ or exp Psychotic Disorders/ or ((mental* or psychiatr* or psycho*) adj (disorder* or disease* or illness*)).mp.	1313460
34	exp Depressive Disorder, Major/ or exp Depression/ or Depress*.mp. or MDD.mp.	552268
35	exp Anxiety Disorders/ or exp Anxiety/ or anxi*.mp.	289411
36	exp Phobic Disorders/ or phobi*.mp.	17668
37	exp Schizophrenia/ or schizophreni*.mp. or hebephreni*.mp.	150158
38	exp Somatoform Disorders/ or ((somatoform* or somati* or medically unexplained or briquet or pain) adj (disorder* or syndrome* or symptom*)).mp. or exp Medically Unexplained Symptoms/	49713
39	exp Dissociative Disorders/ or (dissociative adj (disorder* or hysteri* or reaction*)).mp. or dissociation*.mp.	115459
40	exp Hysteria/ or hysteri*.mp.	6074
41	exp Mood Disorders/ or ((affective* or mood*) adj (disorder* or disease* or illness* or symptom*)).mp.	158894

42	exp Stress Disorders, Post-Traumatic/ or PTSD.mp. or ((post trauma* or posttrauma*) adj (stress* or neurose*)).mp. or combat disorder*.mp. or war disorder*.mp.	48359
43	exp Cognition Disorders/ or ((cognitive or cognition or mental or neurocognitive) adj (dysfunction* or decline* or impairment* or deterioration* or disorder* or illness* or disease*)).mp.	377493
44	exp Personality Disorders/ or personality disorder*.mp.	48830
45	exp "Disruptive, Impulse Control, and Conduct Disorders"/ or impulse control disorder*.mp. or intermittent explosive disorder*.mp.	9781
46	exp "Feeding and Eating Disorders"/ or ((eating or appetite or feeding) adj disorder*).mp.	38361
47	exp Bipolar Disorder/ or ((bipolar or mani*) adj (disorder* or illness* or disease*)).mp.	51927
48	exp Obsessive-Compulsive Disorder/ or OCD*.mp. or ((obsess*-compulsi* or obsess* or compulsi*) adj (disorder* or illness* or disease* or neuros*)).mp.	22060
49	exp Panic Disorder/ or (panic adj (attack* or disorder*)).mp.	12638
50	exp Agoraphobia/ or agoraphobi*.mp.	4210
51	exp Neurotic Disorders/ or neuros*.mp. or neurotic disorder*.mp. or psychoneuros*.mp.	190989
52	33 or 34 or 35 or 36 or 37 or 38 or 39 or 40 or 41 or 42 or 43 or 44 or 45 or 46 or 47 or 48 or 49 or 50 or 51	2107749
53	exp Communicable Diseases/ or ((communic* or contag* or transmi* or infect*) adj (disease* or infection* or illness*)).mp.	212955
54	exp Bacterial Infections/ or bacteri* infection*.mp.	916393
55	exp Conjunctivitis/ or conjunctivitis.mp.	24690
56	exp HIV/ or hiv.mp. or Human immuno deficiency virus.mp.	362332
57	exp Acquired Immunodeficiency Syndrome/ or AIDS.mp. or immunodeficiency associated virus.mp. or immun* deficiency associated virus.mp. or acquired immunodeficiency syndrome*.mp. or acquired immun* deficiency syndrome*.mp.	226679
58	exp Buruli Ulcer/ or Bairnsdale.mp. or Buruli.mp.	1104
59	exp Onchocerciasis/ or onchocer*.mp.	5978
60	hepatitis.mp. or exp Hepatitis B/ or exp Hepatitis C/	256450
61	exp Leishmaniasis/ or leishmania*.mp.	37940
62	exp Leprosy/ or lepros*.mp. or hansen*.mp.	31193
63	exp Elephantiasis, Filarial/ or elephantias*.mp. or filaria*.mp.	14354
64	exp Trachoma/ or egyptian ophthalmia*.mp. or trachoma*.mp.	20432
65	exp Chikungunya Fever/ or chickungunya.mp. or chikungunya.mp.	5766

66	exp Taeniasis/ or taenia*.mp.	12366
67	exp Cysticercosis/ or cysticercos*.mp.	7146
68	exp Echinococcosis/ or hydatid*.mp. or echinococc*.mp.	29913
69	exp Chagas Disease/ or trypanosom*.mp. or chagas.mp.	44250
70	exp Trypanosomiasis/ or sleeping sickness.mp.	23416
71	exp Encephalitis, Japanese/ or (japanese adj3 encephalitis).mp.	5847
72	exp Syphilis/ or syphilis.mp.	37477
73	53 or 54 or 55 or 56 or 57 or 58 or 59 or 60 or 61 or 62 or 63 or 64 or 65 or 66 or 67 or 68 or 69 or 70 or 71 or 72	1872806
74	exp Tuberculosis/	192915
75	Tuberculos*.mp.	256334
76	TB.mp.	56951
77	koch*.mp.	9469
78	exp Tuberculosis/ or Tuberculos*.mp. or TB.mp. or koch*.mp.	283447
79	(multiple adj (ill* or disease* or condition* or syndrom* or disorder*)).mp.	5522
80	((Cooccur* or co-occur* or coexist* or co-exist* or multipl* or concord* or discord* or long-term or physical*) adj3 (disease* or ill* or care or condition* or disorder* or health* or medication* or symptom* or syndrom*)).mp.	269814
81	(comorbid* or multimorbid* or co-occurren* or co-morbid* or Multidisease* or multi-disease*).mp.	268450
82	(comorbid* or multimorbid* or co-occurren* or co-morbid* or multi-morbid* or Multidisease* or multi-disease*).mp.	268954
83	exp Comorbidity/ or exp Multimorbidity/ or exp Multiple Chronic Conditions/	111238
84	79 or 80 or 81 or 82 or 83	519584
85	exp "Systematic Review"/	137409
86	"systematic review*".m_titl.	134940
87	exp Meta-Analysis/	121268
88	"meta-analys*".m_titl.	115466
89	exp "Systematic Review"/ or "systematic review*".m_titl. or exp Meta-Analysis/ or "meta-analys*".m_titl.	260449
90	32 or 52 or 73	13060984
91	(32 or 52 or 73) and 78	228989
92	(32 or 52 or 73) and 78 and 84	4072

93	(32 or 52 or 73) and 78 and 84 and 89	89
94	exp Animals/ not exp Humans/	4747614
95	((32 or 52 or 73) and 78 and 84) not 94	4004
96	((32 or 52 or 73) and 78 and 84 and 89) not 94	89
97	((32 or 52 or 73) and 78 and 89) not 94	1701

For peer review only

Embase (October 23rd 2020)

# ▲	Searches	Results
1	exp non communicable disease/ or ((Non-communicable or Noncommunicable or Non-infectious) adj (disease* or condition* or illness*)).mp.	16547
2	exp chronic disease/ or ((chronic or long-term) adj (disease* or condition* or illness*)).mp.	268915
3	exp heart disease/ or (heart adj (disease* or disorder* or failure)).mp. or (cardiac adj (disease* or disorder* or failure)).mp.	1935434
4	exp cardiovascular disease/ or (cardiovascular adj (disease* or disorder* or failure)).mp.	4171750
5	exp coronary artery disease/ or (coronary adj (disease* or disorder* or failure)).mp.	338836
6	exp cerebrovascular disease/ or (cerebrovascular adj (disease* or disorder* or insufficienc* or occlusion*)).mp. or (vascular adj (disease* or disorder*)).mp. or (carotid* adj (disease* or disorder*)).mp.	760319
7	exp peripheral occlusive artery disease/ or (arter* adj (disease* or disorder*)).mp.	427607
8	exp rheumatic heart disease/ or exp congenital heart malformation/ or (heart adj3 (malform* or defect* or congeni*)).mp.	187115
9	exp vein thrombosis/ or ((deep vein or deep venous) adj thrombos*).mp. or phlebothrombos*.mp.	137063
10	exp lung embolism/ or (pulmonar* adj (thromboembolism* or embolism* or disease* or disorder*)).mp.	205531
11	exp cerebrovascular accident/ or stroke.mp.	513128
12	exp neoplasm/ or Cancer*.mp. or neoplas*.mp. or tumor*.mp.	5810559
13	exp lung disease/ or exp respiratory tract disease/ or exp chronic obstructive lung disease/ or ((lung* or respiratory or pulmonar* or airflow or airway) adj2 (disease* or obstruct* or hypersensitiv*)).mp. or exp asthma/ or asthma*.mp. or exp chronic obstructive lung disease/ or exp respiratory tract allergy/	2529309
14	exp diabetes mellitus/ or diabet*.mp.	1165861
15	exp autoimmune disease/ or ((autoimmun* or auto immun* or autoaggress* or auto aggress*) adj (disorder* or disease*)).mp.	628015
16	exp metabolic syndrome X/ or exp metabolic disorder/ or ((metabolic or insulin resistance) adj (disorder* or disease* or syndrome*)).mp.	2728282
17	exp obesity/ or obes*.mp.	632387
18	exp osteoporosis/ or osteoporo*.mp. or bone loss.mp. or exp osteolysis/ or osteolysis.mp. or bone resorption.mp.	237604
19	exp Parkinson disease/ or parkinson*.mp. or paralysis agitans.mp.	212471

20	exp arthritis/ or arthriti*.mp. or polyarthriti*.mp. or rheumarthriti*.mp.	510423
21	exp kidney disease/ or (kidney adj (disease* or disorder*)).mp.	973568
22	exp liver disease/ or (liver adj (disease* or disorder* or dysfunction*)).mp.	1021173
23	exp hypertension/ or high blood pressure*.mp. or hypertens*.mp.	1010094
24	exp hyperlipidemia/ or hyperlipem*.mp. or hyperlipidem*.mp. or lipem*.mp. or lipidem*.mp.	170635
25	exp hypercholesterolemia/ or ((high* or elevat*) adj cholesterol*).mp. or hypercholesterem*.mp. or hypercholesterolem*.mp.	90843
26	exp hypertriglyceridemia/ or hypertriglyceridem*.mp.	31673
27	exp thyroid disease/ or (thyroid adj (disease* or disorder*)).mp. or exp hyperthyroidism/ or hyperthyroid*.mp. or exp hypothyroidism/ or hypothyroid*.mp. or ((thyroid-stimulating hormone* or tsh) adj deficien*).mp.	238358
28	exp motor neuron disease/ or motor neuron* disease*.mp. or lateral scleros*.mp. or motor system disease*.mp.	52581
29	exp multiple sclerosis/ or multiple sclerosis.mp. or disseminated sclerosis.mp.	143653
30	exp emphysema/ or emphysema*.mp.	52663
31	exp bronchitis/ or bronchit*.mp.	70185
32	1 or 2 or 3 or 4 or 5 or 6 or 7 or 8 or 9 or 10 or 11 or 12 or 13 or 14 or 15 or 16 or 17 or 18 or 19 or 20 or 21 or 22 or 23 or 24 or 25 or 26 or 27 or 28 or 29 or 30 or 31	13930977
33	exp mental disease/ or exp psychosis/ or ((mental* or psychiatr* or psycho*) adj (disorder* or disease* or illness*)).mp.	2252075
34	exp major depression/ or exp depression/ or Depress*.mp. or MDD.mp.	837980
35	exp anxiety disorder/ or exp anxiety/ or anxi*.mp.	518902
36	exp phobia/ or phobi*.mp.	35665
37	exp schizophrenia/ or schizophreni*.mp. or hebephreni*.mp.	209868
38	exp somatoform disorder/ or ((somatoform* or somati* or medically unexplained or briquet or pain) adj (disorder* or syndrome* or symptom*)).mp. or exp medically unexplained symptom/	82640
39	exp dissociative disorder/ or (dissociative adj (disorder* or hysteri* or reaction*)).mp. or dissociation*.mp.	142193
40	exp hysteria/ or hysteri*.mp.	7294
41	exp mood disorder/ or ((affective* or mood*) adj (disorder* or disease* or illness* or symptom*)).mp.	539796
42	exp posttraumatic stress disorder/ or PTSD.mp. or ((post trauma* or posttrauma*) adj (stress* or neurose*)).mp. or combat disorder*.mp. or war disorder*.mp.	68828

43	exp cognitive defect/ or ((cognitive or cognition or mental or neurocognitive) adj (dysfunction* or decline* or impairment* or deterioration* or disorder* or illness* or disease*)).mp.	802224
44	exp personality disorder/ or personality disorder*.mp.	64830
45	exp impulse control disorder/ or impulse control disorder*.mp. or intermittent explosive disorder*.mp.	12514
46	exp eating disorder/ or ((eating or appetite or feeding) adj disorder*).mp.	64625
47	exp bipolar disorder/ or ((bipolar or mani*) adj (disorder* or illness* or disease*)).mp.	73910
48	exp obsessive compulsive disorder/ or OCD*.mp. or ((obsess*-compulsi* or obsess* or compulsi*) adj (disorder* or illness* or disease* or neuros*)).mp.	46156
49	exp panic/ or (panic adj (attack* or disorder*)).mp.	25651
50	exp agoraphobia/ or agoraphobi*.mp.	7128
51	exp neurosis/ or neuros*.mp. or neurotic disorder*.mp. or psychoneuros*.mp.	284790
52	33 or 34 or 35 or 36 or 37 or 38 or 39 or 40 or 41 or 42 or 43 or 44 or 45 or 46 or 47 or 48 or 49 or 50 or 51	3032495
53	exp communicable disease/ or ((communic* or contag* or transmi* or infect*) adj (disease* or infection* or illness*)).mp.	227165
54	exp bacterial infection/ or bacteri* infection*.mp.	875706
55	exp conjunctivitis/ or conjunctivitis.mp.	42120
56	exp Human immunodeficiency virus/ or hiv.mp. or Human immuno deficiency virus.mp.	440016
57	exp acquired immune deficiency syndrome/ or AIDS.mp. or immunodeficiency associated virus.mp. or immun* deficiency associated virus.mp. or acquired immunodeficiency syndrome*.mp. or acquired immun* deficiency syndrome*.mp.	245085
58	exp Buruli ulcer/ or Bairnsdale.mp. or Buruli.mp.	1384
59	exp onchocerciasis/ or onchocer*.mp.	7089
60	hepatitis.mp. or exp hepatitis B/ or exp hepatitis C/	401827
61	exp leishmaniasis/ or leishmania*.mp.	45114
62	exp leprosy/ or lepros*.mp. or hansen*.mp.	32759
63	exp elephantiasis/ or elephantias*.mp. or filaria*.mp.	17315
64	exp trachoma/ or egyptian ophthalmia*.mp. or trachoma*.mp.	25318
65	exp chikungunya/ or chickungunya.mp. or chikungunya.mp.	8172
66	exp taeniasis/ or taenia*.mp.	13801
67	exp Cysticercosis/ or cysticercos*.mp.	5977

68	exp echinococcosis/ or hydatid*.mp. or echinococc*.mp.	30362
69	exp Chagas disease/ or trypanosom*.mp. or chagas.mp.	47764
70	exp trypanosomiasis/ or sleeping sickness.mp.	25238
71	exp Japanese encephalitis/ or (japanese adj3 encephalitis).mp.	7060
72	exp syphilis/ or syphilis.mp.	32633
73	53 or 54 or 55 or 56 or 57 or 58 or 59 or 60 or 61 or 62 or 63 or 64 or 65 or 66 or 67 or 68 or 69 or 70 or 71 or 72	2039904
74	exp tuberculosis/	193084
75	Tuberculos*.mp.	245295
76	TB.mp.	72736
77	koch*.mp.	12432
78	exp tuberculosis/ or Tuberculos*.mp. or TB.mp. or koch*.mp.	284028
79	(multiple adj (ill* or disease* or condition* or syndrom* or disorder*)).mp.	7515
80	((Cooccur* or co-occur* or coexist* or co-exist* or multipl* or concord* or discord* or long-term or physical*) adj3 (disease* or ill* or care or condition* or disorder* or health* or medication* or symptom* or syndrom*)).mp.	468523
81	(comorbid* or multimorbid* or co-occurren* or co-morbid* or Multidisease* or multi-disease*).mp.	461825
82	(comorbid* or multimorbid* or co-occurren* or co-morbid* or multi-morbid* or Multidisease* or multi-disease*).mp.	462635
83	exp Comorbidity/ or exp Multimorbidity/ or exp Multiple Chronic Conditions/	281021
84	79 or 80 or 81 or 82 or 83	895311
85	exp "systematic review"/	268161
86	"systematic review* ".m_titl.	163162
87	exp meta analysis/	200254
88	"meta-analys* ".m_titl.	144571
89	exp "systematic review"/ or "systematic review* ".m_titl. or exp meta analysis/ or "meta-analys* ".m_titl.	411674
90	32 or 52 or 73	16955591
91	(32 or 52 or 73) and 78	237676
92	(32 or 52 or 73) and 78 and 84	9231
93	(32 or 52 or 73) and 78 and 84 and 89	271
94	exp animal/ not exp human/	4710933

95	((32 or 52 or 73) and 78 and 84) not 94	9130
96	((32 or 52 or 73) and 78 and 84 and 89) not 94	271
97	((32 or 52 or 73) and 78 and 89) not 94	3315

For peer review only

PsycINFO (October 23rd 2020)

# ▲	Searches	Results
1	((Non-communicable or Noncommunicable or Non-infectious) adj (disease* or condition* or illness*)).mp.	1054
2	exp Chronic Illness/ or ((chronic or long-term) adj (disease* or condition* or illness*)).mp.	48942
3	exp Heart Disorders/ or (heart adj (disease* or disorder* or failure)).mp. or (cardiac adj (disease* or disorder* or failure)).mp.	22461
4	exp Cardiovascular Disorders/ or (cardiovascular adj (disease* or disorder* or failure)).mp.	68973
5	(coronary adj (disease* or disorder* or failure)).mp.	450
6	exp Cerebrovascular Disorders/ or (cerebrovascular adj (disease* or disorder* or insufficienc* or occlusion*)).mp. or (vascular adj (disease* or disorder*)).mp. or (carotid* adj (disease* or disorder*)).mp.	31536
7	(arter* adj (disease* or disorder*)).mp.	2605
8	(heart adj3 (malform* or defect* or congeni*)).mp.	1007
9	exp Thromboses/ or ((deep vein or deep venous) adj thrombos*).mp. or phlebothrombos*.mp.	1070
10	exp Embolisms/ or (pulmonar* adj (thromboembolism* or embolism* or disease* or disorder*)).mp.	3511
11	exp Cerebrovascular Accidents/ or stroke.mp.	36761
12	exp Neoplasms/ or Cancer*.mp. or neoplas*.mp. or tumor*.mp.	83903
13	exp Lung Disorders/ or exp Respiratory Tract Disorders/ or ((lung* or respiratory or pulmonar* or airflow or airway) adj2 (disease* or obstruct* or hypersensitiv*)).mp. or exp Asthma/ or asthma*.mp. or exp Chronic Obstructive Pulmonary Disease/	20498
14	exp Diabetes Mellitus/ or diabet*.mp.	33566
15	((autoimmun* or auto immun* or autoaggress* or auto aggress*) adj (disorder* or disease*)).mp.	2453
16	exp Metabolic Syndrome/ or ((metabolic or insulin resistance) adj (disorder* or disease* or syndrome*)).mp.	5856

17	exp Obesity/ or obes*.mp.	45201
18	exp Osteoporosis/ or osteoporo*.mp. or bone loss.mp. or osteolysis.mp. or bone resorption.mp.	2582
19	exp Parkinson's Disease/ or parkinson*.mp. or paralysis agitans.mp.	36257
20	exp Arthritis/ or arthriti*.mp. or polyarthriti*.mp. or rheumarthriti*.mp.	7176
21	exp Kidney Diseases/ or (kidney adj (disease* or disorder*)).mp.	2912
22	exp Liver Disorders/ or (liver adj (disease* or disorder* or dysfunction*)).mp.	5371
23	exp Hypertension/ or high blood pressure*.mp. or hypertens*.mp.	20357
24	(hyperlipem* or hyperlipidem* or lipem* or lipidem*).mp.	1314
25	(((high* or elevat*) adj cholesterol*) or hypercholesterem* or hypercholesterolem*).mp.	1637
26	hypertriglyceridem*.mp.	293
27	exp Thyroid Disorders/ or (thyroid adj (disease* or disorder*)).mp. or exp Hyperthyroidism/ or hyperthyroid*.mp. or exp Hypothyroidism/ or hypothyroid*.mp. or ((thyroid-stimulating hormone* or tsh) adj deficien*).mp.	2924
28	exp Nervous System Disorders/ or motor neuron* disease*.mp. or lateral scleros*.mp. or motor system disease*.mp.	319159
29	exp Multiple Sclerosis/ or multiple sclerosis.mp. or disseminated sclerosis.mp.	16491
30	exp Pulmonary Emphysema/ or emphysema*.mp.	285
31	exp Bronchial Disorders/ or bronchit*.mp.	476
32	1 or 2 or 3 or 4 or 5 or 6 or 7 or 8 or 9 or 10 or 11 or 12 or 13 or 14 or 15 or 16 or 17 or 18 or 19 or 20 or 21 or 22 or 23 or 24 or 25 or 26 or 27 or 28 or 29 or 30 or 31	567022
33	exp Mental Disorders/ or exp Psychosis/ or ((mental* or psychiatr* or psycho*) adj (disorder* or disease* or illness*)).mp.	921435
34	exp Major Depression/ or exp "Depression (Emotion)"/ or Depress*.mp. or MDD.mp.	374034
35	exp Anxiety Disorders/ or exp Anxiety/ or anxi*.mp.	273241

36	exp Phobias/ or phobi*.mp.	24242
37	exp Schizophrenia/ or schizophreni*.mp. or hebephreni*.mp.	140371
38	exp Somatoform Disorders/ or ((somatoform* or somati* or medically unexplained or briquet or pain) adj (disorder* or syndrome* or symptom*)).mp.	27313
39	exp Dissociative Disorders/ or (dissociative adj (disorder* or hysteri* or reaction*)).mp. or dissociation*.mp.	24759
40	exp Hysteria/ or hysteri*.mp.	8253
41	exp Affective Disorders/ or ((affective* or mood*) adj (disorder* or disease* or illness* or symptom*)).mp.	170875
42	exp Posttraumatic Stress Disorder/ or PTSD.mp. or ((post trauma* or posttrauma*) adj (stress* or neurose*)).mp. or combat disorder*.mp. or war disorder*.mp.	51508
43	exp Cognitive Impairment/ or ((cognitive or cognition or mental or neurocognitive) adj (dysfunction* or decline* or impairment* or deterioration* or disorder* or illness* or disease*)).mp.	221483
44	exp Personality Disorders/ or personality disorder*.mp.	49598
45	exp Impulse Control Disorders/ or impulse control disorder*.mp. or intermittent explosive disorder*.mp.	2232
46	exp Eating Disorders/ or ((eating or appetite or feeding) adj disorder*).mp.	39055
47	exp Bipolar Disorder/ or ((bipolar or mani*) adj (disorder* or illness* or disease*)).mp.	38332
48	exp Obsessive Compulsive Disorder/ or OCD*.mp. or ((obsess*-compulsi* or obsess* or compulsi*) adj (disorder* or illness* or disease* or neuros*)).mp.	21451
49	exp Panic Disorder/ or (panic adj (attack* or disorder*)).mp.	13643
50	exp Agoraphobia/ or agoraphobi*.mp.	6095
51	exp Neurosis/ or neuros*.mp. or neurotic disorder*.mp. or psychoneuros*.mp.	81072
52	33 or 34 or 35 or 36 or 37 or 38 or 39 or 40 or 41 or 42 or 43 or 44 or 45 or 46 or 47 or 48 or 49 or 50 or 51	1284709

53	exp Infectious Disorders/ or ((communic* or contag* or transmi* or infect*) adj (disease* or infection* or illness*)).mp.	70725
54	exp Bacterial Disorders/ or bacteri* infection*.mp.	2731
55	exp Eye Disorders/ or conjunctivitis.mp.	4950
56	exp HIV/ or hiv.mp. or Human immuno deficiency virus.mp.	57416
57	exp AIDS/ or AIDS.mp. or immunodeficiency associated virus.mp. or immun* deficiency associated virus.mp. or acquired immunodeficiency syndrome*.mp. or acquired immun* deficiency syndrome*.mp.	48739
58	(Bairnsdale or Buruli).mp.	7
59	onchocer*.mp.	47
60	hepatitis.mp. or exp Hepatitis/	4736
61	leishmania*.mp.	61
62	(lepros* or hansen*).mp.	1155
63	(elephantias* or filaria*).mp.	75
64	(egyptian ophthalmia* or trachoma*).mp.	309
65	(chickungunya or chikungunya).mp.	39
66	taenia*.mp.	160
67	cysticercos*.mp.	102
68	(hydatid* or echinococc*).mp.	39
69	(trypanosom* or chagas).mp.	217
70	sleeping sickness.mp.	50
71	(japanese adj3 encephalitis).mp.	73
72	exp Syphilis/ or syphilis.mp.	1870

73	53 or 54 or 55 or 56 or 57 or 58 or 59 or 60 or 61 or 62 or 63 or 64 or 65 or 66 or 67 or 68 or 69 or 70 or 71 or 72	108493
74	exp Tuberculosis/	1223
75	Tuberculos*.mp.	2795
76	TB.mp.	1354
77	koch*.mp.	1045
78	exp Tuberculosis/ or Tuberculos*.mp. or TB.mp. or koch*.mp.	4450
79	(multiple adj (ill* or disease* or condition* or syndrom* or disorder*)).mp.	913
80	((Cooccur* or co-occur* or coexist* or co-exist* or multipl* or concord* or discord* or long-term or physical*) adj3 (disease* or ill* or care or condition* or disorder* or health* or medication* or symptom* or syndrom*)).mp.	116177
81	(comorbid* or multimorbid* or co-occurren* or co-morbid* or multi-morbid* or Multidisease* or multi-disease*).mp.	88147
82	exp Comorbidity/	33088
83	79 or 80 or 81 or 82	192590
84	exp "Systematic Review"/	460
85	"systematic review*".m_titl.	21652
86	exp Meta Analysis/	4825
87	"meta-analys*".m_titl.	18645
88	exp "Systematic Review"/ or "systematic review*".m_titl. or exp Meta Analysis/ or "meta-analys*".m_titl.	37279
89	32 or 52 or 73	1665397
90	(32 or 52 or 73) and 78	3046
91	(32 or 52 or 73) and 78 and 83	362

92	(32 or 52 or 73) and 78 and 83 and 88	6
93	(32 or 52 or 73) and 78 and 88	27

For peer review only

Web of Science (October 23rd 2020)

#90	1,039	(#74 OR #52 OR #32) AND #82 AND #85<br>*Indexes=SCI-EXPANDED, SSCI, A&HCI, CPCI-S, CPCI-SSH, ESCI Timespan=All years*
#89	62	(#74 OR #52 OR #32) AND #82 AND #78 AND #85<br>*Indexes=SCI-EXPANDED, SSCI, A&HCI, CPCI-S, CPCI-SSH, ESCI Timespan=All years*
#88	3,336	(#74 OR #52 OR #32) AND #82 AND #78<br>*Indexes=SCI-EXPANDED, SSCI, A&HCI, CPCI-S, CPCI-SSH, ESCI Timespan=All years*
#87	87,846	(#74 OR #52 OR #32) AND #82<br>*Indexes=SCI-EXPANDED, SSCI, A&HCI, CPCI-S, CPCI-SSH, ESCI Timespan=All years*
#86	11,086,931	#74 OR #52 OR #32<br>*Indexes=SCI-EXPANDED, SSCI, A&HCI, CPCI-S, CPCI-SSH, ESCI Timespan=All years*
#85	226,528	#84 OR #83<br>*Indexes=SCI-EXPANDED, SSCI, A&HCI, CPCI-S, CPCI-SSH, ESCI Timespan=All years*
#84	134,872	TI="Meta-analysis"<br>*Indexes=SCI-EXPANDED, SSCI, A&HCI, CPCI-S, CPCI-SSH, ESCI Timespan=All years*
#83	147,319	TI = "Systematic Review"<br>*Indexes=SCI-EXPANDED, SSCI, A&HCI, CPCI-S, CPCI-SSH, ESCI Timespan=All years*
#82	234,479	#81 OR #80 OR #79<br>*Indexes=SCI-EXPANDED, SSCI, A&HCI, CPCI-S, CPCI-SSH, ESCI Timespan=All years*
#81	19,329	TS=koch*<br>*Indexes=SCI-EXPANDED, SSCI, A&HCI, CPCI-S, CPCI-SSH, ESCI Timespan=All years*
#80	80,779	TS=TB<br>*Indexes=SCI-EXPANDED, SSCI, A&HCI, CPCI-S, CPCI-SSH, ESCI Timespan=All years*

#79	**170,591**	TS=Tuberculos*<br>*Indexes=SCI-EXPANDED, SSCI, A&HCI, CPCI-S, CPCI-SSH, ESCI Timespan=All years*
#78	**659,174**	#77 OR #76 OR #75<br>*Indexes=SCI-EXPANDED, SSCI, A&HCI, CPCI-S, CPCI-SSH, ESCI Timespan=All years*
#77	**238,429**	TS=(comorbid* or multimorbid* or co-occurren* or co-morbid* or multi-morbid* or Multidisease* or multi-disease*)<br>*Indexes=SCI-EXPANDED, SSCI, A&HCI, CPCI-S, CPCI-SSH, ESCI Timespan=All years*
#76	**325,588**	TS=((Cooccur* or co-occur* or coexist* or co-exist* or multipl* or concord* or discord* or long-term or physical*) NEAR/3 (disease* or ill* or care or condition* or disorder* or health* or medication* or symptom* or syndrom*))<br>*Indexes=SCI-EXPANDED, SSCI, A&HCI, CPCI-S, CPCI-SSH, ESCI Timespan=All years*
#75	**191,473**	TS=(multiple NEAR (ill* or disease* or condition* or syndrom* or disorder*))<br>*Indexes=SCI-EXPANDED, SSCI, A&HCI, CPCI-S, CPCI-SSH, ESCI Timespan=All years*
#74	**2,677,956**	#73 OR #72 OR #71 OR #70 OR #69 OR #68 OR #67 OR #66 OR #65 OR #64 OR #63 OR #62 OR #61 OR #60 OR #59 OR #58 OR #57 OR #56 OR #55 OR #54 OR #53<br>*Indexes=SCI-EXPANDED, SSCI, A&HCI, CPCI-S, CPCI-SSH, ESCI Timespan=All years*
#73	**23,389**	TS=syphilis<br>*Indexes=SCI-EXPANDED, SSCI, A&HCI, CPCI-S, CPCI-SSH, ESCI Timespan=All years*
#72	**5,981**	TS=(japanese NEAR/3 encephalitis)<br>*Indexes=SCI-EXPANDED, SSCI, A&HCI, CPCI-S, CPCI-SSH, ESCI Timespan=All years*
#71	**4,257**	TS=sleeping sickness<br>*Indexes=SCI-EXPANDED, SSCI, A&HCI, CPCI-S, CPCI-SSH, ESCI Timespan=All years*
#70	**50,013**	TS=trypanosom* or TS=chagas<br>*Indexes=SCI-EXPANDED, SSCI, A&HCI, CPCI-S, CPCI-SSH, ESCI Timespan=All years*
#69	**19,785**	TS=hydatid* OR TS=echinococc*<br>*Indexes=SCI-EXPANDED, SSCI, A&HCI, CPCI-S, CPCI-SSH, ESCI Timespan=All years*

# 68	4,657	TS=cysticercos*<br>*Indexes=SCI-EXPANDED, SSCI, A&HCI, CPCI-S, CPCI-SSH, ESCI Timespan=All years*
# 67	8,257	TS=taenia*<br>*Indexes=SCI-EXPANDED, SSCI, A&HCI, CPCI-S, CPCI-SSH, ESCI Timespan=All years*
# 66	6,629	TS=chickungunya Or TS=chikungunya<br>*Indexes=SCI-EXPANDED, SSCI, A&HCI, CPCI-S, CPCI-SSH, ESCI Timespan=All years*
# 65	19,987	TS=trachoma*<br>*Indexes=SCI-EXPANDED, SSCI, A&HCI, CPCI-S, CPCI-SSH, ESCI Timespan=All years*
# 64	5	TS=egyptian ophthalmia*<br>*Indexes=SCI-EXPANDED, SSCI, A&HCI, CPCI-S, CPCI-SSH, ESCI Timespan=All years*
# 63	11,257	TS=elephantias* OR TS=filaria*<br>*Indexes=SCI-EXPANDED, SSCI, A&HCI, CPCI-S, CPCI-SSH, ESCI Timespan=All years*
# 62	30,101	TS=lepros* OR TS=hansen*<br>*Indexes=SCI-EXPANDED, SSCI, A&HCI, CPCI-S, CPCI-SSH, ESCI Timespan=All years*
# 61	41,800	TS=leishmania*<br>*Indexes=SCI-EXPANDED, SSCI, A&HCI, CPCI-S, CPCI-SSH, ESCI Timespan=All years*
# 60	267,930	TS=hepatitis<br>*Indexes=SCI-EXPANDED, SSCI, A&HCI, CPCI-S, CPCI-SSH, ESCI Timespan=All years*
# 59	6,453	TS=onchocer*<br>*Indexes=SCI-EXPANDED, SSCI, A&HCI, CPCI-S, CPCI-SSH, ESCI Timespan=All years*
# 58	1,120	TS=Bairnsdale OR TS=Buruli<br>*Indexes=SCI-EXPANDED, SSCI, A&HCI, CPCI-S, CPCI-SSH, ESCI Timespan=All years*
# 57	640,388	TS=AIDS OR TS=immunodeficiency associated virus OR TS=immun* deficiency associated virus OR TS=acquired immunodeficiency syndrome* OR TS=acquired immun* deficiency syndrome*

		Indexes=SCI-EXPANDED, SSCI, A&HCI, CPCI-S, CPCI-SSH, ESCI Timespan=All years
#56	374,418	TS=hiv OR TS=Human immuno deficiency virus<br>*Indexes=SCI-EXPANDED, SSCI, A&HCI, CPCI-S, CPCI-SSH, ESCI Timespan=All years*
#55	10,545	TS=conjunctivitis<br>*Indexes=SCI-EXPANDED, SSCI, A&HCI, CPCI-S, CPCI-SSH, ESCI Timespan=All years*
#54	240,625	TS=bacteri* infection*<br>*Indexes=SCI-EXPANDED, SSCI, A&HCI, CPCI-S, CPCI-SSH, ESCI Timespan=All years*
#53	1,710,573	TS=((communic* or contag* or transmi* or infect*) NEAR (disease* or infection* or illness*))<br>*Indexes=SCI-EXPANDED, SSCI, A&HCI, CPCI-S, CPCI-SSH, ESCI Timespan=All years*
#52	1,891,336	#51 OR #50 OR #49 OR #48 OR #47 OR #46 OR #45 OR #44 OR #43 OR #42 OR #41 OR #40 OR #39 OR #38 OR #37 OR #36 OR #35 OR #34 OR #33<br>*Indexes=SCI-EXPANDED, SSCI, A&HCI, CPCI-S, CPCI-SSH, ESCI Timespan=All years*
#51	155,801	TS=neuros* OR TS=neurotic disorder* OR TS=psychoneuros*<br>*Indexes=SCI-EXPANDED, SSCI, A&HCI, CPCI-S, CPCI-SSH, ESCI Timespan=All years*
#50	5,165	TS=agoraphobi*<br>*Indexes=SCI-EXPANDED, SSCI, A&HCI, CPCI-S, CPCI-SSH, ESCI Timespan=All years*
#49	19,459	TS=(panic NEAR (attack* or disorder*))<br>*Indexes=SCI-EXPANDED, SSCI, A&HCI, CPCI-S, CPCI-SSH, ESCI Timespan=All years*
#48	32,551	TS=OCD* OR TS=((obsess*-compulsi* or obsess* or compulsi*) NEAR (disorder* or illness* or disease* or neuros*))<br>*Indexes=SCI-EXPANDED, SSCI, A&HCI, CPCI-S, CPCI-SSH, ESCI Timespan=All years*
#47	137,039	TS=((bipolar or mani*) NEAR (disorder* or illness* or disease*))<br>*Indexes=SCI-EXPANDED, SSCI, A&HCI, CPCI-S, CPCI-SSH, ESCI Timespan=All years*
#46	35,573	TS=((eating or appetite or feeding) NEAR disorder*)<br>*Indexes=SCI-EXPANDED, SSCI, A&HCI, CPCI-S, CPCI-SSH, ESCI Timespan=All years*

# 45	4,535	TS=impulse control disorder* OR TS=intermittent explosive disorder*<br>*Indexes=SCI-EXPANDED, SSCI, A&HCI, CPCI-S, CPCI-SSH, ESCI Timespan=All years*
# 44	52,384	TS=personality disorder*<br>*Indexes=SCI-EXPANDED, SSCI, A&HCI, CPCI-S, CPCI-SSH, ESCI Timespan=All years*
# 43	325,105	TS=((cognitive or cognition or mental or neurocognitive) NEAR (dysfunction* or decline* or impairment* or deterioration* or disorder* or illness* or disease*))<br>*Indexes=SCI-EXPANDED, SSCI, A&HCI, CPCI-S, CPCI-SSH, ESCI Timespan=All years*
# 42	69,002	TS=PTSD OR TS=(((post NEAR trauma*) or posttrauma*) NEAR (stress* or neurose*)) OR TS=combat disorder* OR TS=war disorder*<br>*Indexes=SCI-EXPANDED, SSCI, A&HCI, CPCI-S, CPCI-SSH, ESCI Timespan=All years*
# 41	73,280	TS=((affective* or mood*) NEAR (disorder* or disease* or illness* or symptom*))<br>*Indexes=SCI-EXPANDED, SSCI, A&HCI, CPCI-S, CPCI-SSH, ESCI Timespan=All years*
# 40	6,468	TS=hysteri*<br>*Indexes=SCI-EXPANDED, SSCI, A&HCI, CPCI-S, CPCI-SSH, ESCI Timespan=All years*
# 39	215,870	TS=(dissociative NEAR (disorder* or hysteri* or reaction*)) OR TS=dissociation*<br>*Indexes=SCI-EXPANDED, SSCI, A&HCI, CPCI-S, CPCI-SSH, ESCI Timespan=All years*
# 38	122,031	TS=((somatoform* or somati* or (medically NEAR unexplained) or briquet or pain) NEAR (disorder* or syndrome* or symptom*))<br>*Indexes=SCI-EXPANDED, SSCI, A&HCI, CPCI-S, CPCI-SSH, ESCI Timespan=All years*
# 37	200,099	TS=schizophreni* OR TS=hebephreni*<br>*Indexes=SCI-EXPANDED, SSCI, A&HCI, CPCI-S, CPCI-SSH, ESCI Timespan=All years*
# 36	19,359	TS=phobi*<br>*Indexes=SCI-EXPANDED, SSCI, A&HCI, CPCI-S, CPCI-SSH, ESCI Timespan=All years*
# 35	300,816	TS=anxi*<br>*Indexes=SCI-EXPANDED, SSCI, A&HCI, CPCI-S, CPCI-SSH, ESCI Timespan=All years*
# 34	645,434	TS=Depress* OR TS=MDD<br>*Indexes=SCI-EXPANDED, SSCI, A&HCI, CPCI-S, CPCI-SSH, ESCI Timespan=All years*

# 33	283,212	TS=((mental* or psychiatr* or psycho*) NEAR (disorder* or disease* or illness*))<br>*Indexes=SCI-EXPANDED, SSCI, A&HCI, CPCI-S, CPCI-SSH, ESCI Timespan=All years*
# 32	7,479,780	#31 OR #30 OR #29 OR #28 OR #27 OR #26 OR #25 OR #24 OR #23 OR #22 OR #21 OR #20 OR #19 OR #18 OR #17 OR #16 OR #15 OR #14 OR #13 OR #12 OR #11 OR #10 OR #9 OR #8 OR #7 OR #6 OR #5 OR #4 OR #3 OR #2 OR #1<br>*Indexes=SCI-EXPANDED, SSCI, A&HCI, CPCI-S, CPCI-SSH, ESCI Timespan=All years*
# 31	20,591	TS=bronchit*<br>*Indexes=SCI-EXPANDED, SSCI, A&HCI, CPCI-S, CPCI-SSH, ESCI Timespan=All years*
# 30	26,727	TS=emphysema*<br>*Indexes=SCI-EXPANDED, SSCI, A&HCI, CPCI-S, CPCI-SSH, ESCI Timespan=All years*
# 29	129,081	TS=multiple sclerosis OR TS=disseminated sclerosis<br>*Indexes=SCI-EXPANDED, SSCI, A&HCI, CPCI-S, CPCI-SSH, ESCI Timespan=All years*
# 28	67,922	TS=motor neuron* disease* OR TS=lateral scleros* OR TS=motor system disease*<br>*Indexes=SCI-EXPANDED, SSCI, A&HCI, CPCI-S, CPCI-SSH, ESCI Timespan=All years*
# 27	71,105	TS=(thyroid NEAR (disease* or disorder*)) OR TS=hyperthyroid* OR TS=hypothyroid* OR TS=(((thyroid-stimulating NEAR hormone*) or tsh) NEAR deficien*)<br>*Indexes=SCI-EXPANDED, SSCI, A&HCI, CPCI-S, CPCI-SSH, ESCI Timespan=All years*
# 26	14,111	TS=hypertriglyceridem*<br>*Indexes=SCI-EXPANDED, SSCI, A&HCI, CPCI-S, CPCI-SSH, ESCI Timespan=All years*
# 25	113,697	TS=((high* or elevat*) NEAR cholesterol*) Or TS=hypercholesterem* OR TS=hypercholesterolem*<br>*Indexes=SCI-EXPANDED, SSCI, A&HCI, CPCI-S, CPCI-SSH, ESCI Timespan=All years*
# 24	36,981	TS=hyperlipem* OR TS=hyperlipidem* or TS=lipem* OR TS=lipidem*<br>*Indexes=SCI-EXPANDED, SSCI, A&HCI, CPCI-S, CPCI-SSH, ESCI Timespan=All years*
# 23	613,541	TS=high blood pressure* OR TS=hypertens*<br>*Indexes=SCI-EXPANDED, SSCI, A&HCI, CPCI-S, CPCI-SSH, ESCI Timespan=All years*

# 22	161,103	TS=(liver NEAR (disease* or disorder* or dysfunction*))<br>*Indexes=SCI-EXPANDED, SSCI, A&HCI, CPCI-S, CPCI-SSH, ESCI Timespan=All years*
# 21	125,492	TS=(kidney NEAR (disease* or disorder*))<br>*Indexes=SCI-EXPANDED, SSCI, A&HCI, CPCI-S, CPCI-SSH, ESCI Timespan=All years*
# 20	278,897	TS=arthriti* OR TS=polyarthriti* OR TS=rheumarthriti*<br>*Indexes=SCI-EXPANDED, SSCI, A&HCI, CPCI-S, CPCI-SSH, ESCI Timespan=All years*
# 19	181,735	TS=parkinson* OR TS=paralysis agitans<br>*Indexes=SCI-EXPANDED, SSCI, A&HCI, CPCI-S, CPCI-SSH, ESCI Timespan=All years*
# 18	195,021	TS=osteoporo* OR TS=bone loss OR TS=osteolysis or TS=bone resorption<br>*Indexes=SCI-EXPANDED, SSCI, A&HCI, CPCI-S, CPCI-SSH, ESCI Timespan=All years*
# 17	426,882	TS=obes*<br>*Indexes=SCI-EXPANDED, SSCI, A&HCI, CPCI-S, CPCI-SSH, ESCI Timespan=All years*
# 16	174,058	TS=((metabolic or (insulin near resistance)) NEAR (disorder* or disease* or syndrome*))<br>*Indexes=SCI-EXPANDED, SSCI, A&HCI, CPCI-S, CPCI-SSH, ESCI Timespan=All years*
# 15	109,133	TS=((autoimmun* or (auto NEAR immun*) or autoaggress* or (auto NEAR aggress*)) NEAR (disorder* or disease*))<br>*Indexes=SCI-EXPANDED, SSCI, A&HCI, CPCI-S, CPCI-SSH, ESCI Timespan=All years*
# 14	771,913	TS=diabet*<br>*Indexes=SCI-EXPANDED, SSCI, A&HCI, CPCI-S, CPCI-SSH, ESCI Timespan=All years*
# 13	385,258	TS=((lung* or respiratory or pulmonar* or airflow or airway) NEAR/2 (disease* or obstruct* or hypersensitiv*)) OR TS=asthma*<br>*Indexes=SCI-EXPANDED, SSCI, A&HCI, CPCI-S, CPCI-SSH, ESCI Timespan=All years*
# 12	3,520,631	TS=Cancer* or TS=neoplas* OR TS=tumor*<br>*Indexes=SCI-EXPANDED, SSCI, A&HCI, CPCI-S, CPCI-SSH, ESCI Timespan=All years*
# 11	356,775	TS=stroke<br>*Indexes=SCI-EXPANDED, SSCI, A&HCI, CPCI-S, CPCI-SSH, ESCI Timespan=All years*

#10	164,438	TS=(pulmonar* NEAR (thromboembolism* or embolism* or disease* or disorder*))<br>*Indexes=SCI-EXPANDED, SSCI, A&HCI, CPCI-S, CPCI-SSH, ESCI Timespan=All years*
#9	34,725	TS=(("deep vein" or "deep venous") NEAR thrombos*) OR TS=phlebothrombos*<br>*Indexes=SCI-EXPANDED, SSCI, A&HCI, CPCI-S, CPCI-SSH, ESCI Timespan=All years*
#8	52,910	TS=(heart NEAR/3 (malform* or defect* or congeni*))<br>*Indexes=SCI-EXPANDED, SSCI, A&HCI, CPCI-S, CPCI-SSH, ESCI Timespan=All years*
#7	223,717	TS=(arter* NEAR (disease* or disorder*))<br>*Indexes=SCI-EXPANDED, SSCI, A&HCI, CPCI-S, CPCI-SSH, ESCI Timespan=All years*
#6	131,935	TS=(cerebrovascular NEAR (disease* or disorder* or insufficienc* or occlusion*)) OR TS=(vascular NEAR (disease* or disorder*)) OR TS=(carotid* NEAR (disease* or disorder*))<br>*Indexes=SCI-EXPANDED, SSCI, A&HCI, CPCI-S, CPCI-SSH, ESCI Timespan=All years*
#5	260,403	TS=(coronary NEAR (disease* or disorder* or failure))<br>*Indexes=SCI-EXPANDED, SSCI, A&HCI, CPCI-S, CPCI-SSH, ESCI Timespan=All years*
#4	286,220	TS=(cardiovascular NEAR (disease* or disorder* or failure))<br>*Indexes=SCI-EXPANDED, SSCI, A&HCI, CPCI-S, CPCI-SSH, ESCI Timespan=All years*
#3	561,756	TS=(heart NEAR (disease* or disorder* or failure)) OR TS=(cardiac NEAR (disease* or disorder* or failure))<br>*Indexes=SCI-EXPANDED, SSCI, A&HCI, CPCI-S, CPCI-SSH, ESCI Timespan=All years*
#2	500,606	TS=((chronic or long-term) NEAR (disease* or condition* or illness*))<br>*Indexes=SCI-EXPANDED, SSCI, A&HCI, CPCI-S, CPCI-SSH, ESCI Timespan=All years*
#1	12,403	TS=((Non-communicable or Noncommunicable or Non-infectious) NEAR/1 (disease* or condition* or illness*))<br>*Indexes=SCI-EXPANDED, SSCI, A&HCI, CPCI-S, CPCI-SSH, ESCI Timespan=All years*

Global Index Medicus (October 23rd 2020)

(((("Noncommunicable Diseases" OR ((("Non-communicable" or Noncommunicable or "Non-infectious") AND (disease* or condition* or illness*)))) OR ("Chronic Disease" OR (((chronic or "long-term") AND (disease* or condition* or illness*)))) OR ("Heart Diseases" OR ((heart AND (disease* or disorder* or failure)) or (cardiac AND (disease* or disorder* or failure)))) OR ("Cardiovascular Diseases" OR ((cardiovascular AND (disease* or disorder* or failure)))) OR ("Coronary Disease" OR (coronary AND (disease* or disorder* or failure))) OR ("Cerebrovascular Disorders" OR ((cerebrovascular AND (disease* or disorder* or insufficienc* or occlusion*)) or (vascular AND (disease* or disorder*)) or (carotid* AND (disease* or disorder*)))) OR ("Peripheral Arterial Disease" OR ((arter* AND (disease* or disorder*)))) OR ("Rheumatic Heart Disease" OR "Heart Defects, Congenital" OR ((heart AND (malform* or defect* or congeni*)))) OR ("Venous Thrombosis" OR ((("deep vein" or "deep venous") AND thrombos*) or phlebothrombos*)) OR ("Pulmonary Embolism" OR ((pulmonar* AND (thromboembolism* or embolism* or disease* or disorder*)))) OR ("Stroke" OR (stroke)) OR ("Neoplasms" OR (Cancer* or neoplas* or tumor*)) OR ("Lung Diseases" OR "Respiratory Tract Diseases" OR "Lung Diseases, Obstructive" OR (((lung* or respiratory or pulmonar* or airflow or airway) AND (disease* or obstruct* or hypersensitiv*))) OR "Asthma" OR asthma* OR "Pulmonary Disease, Chronic Obstructive" OR "Respiratory Hypersensitivity") OR ("Diabetes Mellitus" or diabet*) OR ("Autoimmune Diseases" OR ((autoimmun* or (auto AND immun*) or autoaggress* or (auto AND aggress*)) AND (disorder* or disease*))) OR ("Metabolic Syndrome" OR "Metabolic Diseases" OR (((metabolic or "insulin resistance") AND (disorder* or disease* or syndrome*)))) OR ("Obesity" OR obes*) OR ("Osteoporosis" OR (osteoporo* or "bone loss" or "Osteolysis" or osteolysis or "bone resorption")) OR ("Parkinson Disease" OR (parkinson* or "paralysis agitans")) OR ("Arthritis" OR (arthriti* or polyarthriti* or rheumarthriti*)) OR ("Kidney Diseases" OR ((kidney AND (disease* or disorder*)))) OR ("Liver Diseases" OR (liver AND (disease* or disorder* or dysfunction*))) OR ("Hypertension" OR ("high blood pressure" or hypertens*)) OR ("Hyperlipidemias" OR (hyperlipem* or hyperlipidem* or lipem* or lipidem*)) OR ("Hypercholesterolemia" OR (((high* or elevat*) AND cholesterol*) or hypercholesterem* or hypercholesterolem*)) OR ("Hypertriglyceridemia " OR (hypertriglyceridem*)) OR ("Thyroid Diseases" OR (thyroid AND (disease* or disorder*)) OR "Hyperthyroidism " OR (hyperthyroid*) OR "Hypothyroidism" OR (hypothyroid*) OR ((("thyroid-stimulating hormone" or tsh) AND deficien*))) OR ("Motor Neuron Disease" OR ("motor neuron disease") OR (lateral AND scleros*) OR ("motor system disease")) OR ("Multiple Sclerosis" OR ("multiple sclerosis" or "disseminated sclerosis")) OR ("Emphysema" OR emphysema*) OR ("Bronchitis" OR bronchit*)) OR (("Mental Disorders" OR "Psychotic Disorders" OR (((mental* or psychiatr* or psycho*) AND (disorder* or disease* or illness*)))) OR ("Depressive Disorder, Major" OR "Depression" OR (Depress* or MDD)) OR ("Anxiety Disorders" OR "Anxiety" OR anxi*) OR ("Phobic Disorders" OR phobi*) OR ("Schizophrenia" Or (schizophreni* or hebephreni*)) OR ("Somatoform Disorders" OR "Medically Unexplained Symptoms" OR (((somatoform* or somati* or (medically AND unexplained) or briquet or pain) AND (disorder* or syndrome* or symptom*)))) OR ("Dissociative Disorders" OR (dissociative AND (disorder* or hysteri* or reaction*)) or dissociation*) OR ("Hysteria" or hysteri*) OR ("Mood Disorders" OR ((affective* or mood*) AND (disorder* or disease* or illness* or symptom*))) OR ("Stress Disorders, Post-Traumatic" OR (PTSD or ((posttrauma* or trauma*) AND (stress* or neurose*)) or "combat disorder" or "war disorder")) OR ("Cognition Disorders" OR (((cognitive or cognition or mental or neurocognitive) AND (dysfunction* or decline* or impairment* or deterioration* or disorder* or illness* or disease*)))) OR ("Personality Disorders" OR "personality disorder") OR ("Disruptive, Impulse Control, and Conduct Disorders" OR ("impulse control disorder" or "intermittent explosive disorder")) OR ("Feeding and Eating Disorders" OR ((eating or appetite or feeding) AND disorder*)) OR ("Bipolar Disorder" OR ((bipolar or mani*) AND (disorder* or illness* or disease*))) OR ("Obsessive-Compulsive Disorder" OR OCD* or ((obsess* or compulsi*) AND (disorder* or illness* or disease*

For peer review only

or neuros*))) OR ("Panic Disorder" OR ((panic AND (attack* or disorder*)))) OR ("Agoraphobia" OR agoraphobi*) OR ("Neurotic Disorders" OR (neuros* or "neurotic disorder" or psychoneuros*))) OR (("Communicable Diseases" OR (((communic* or contag* or transmi* or infect*) AND (disease* or infection* or illness*)))) OR ("Bacterial Infections" OR (bacteri* infection*)) OR ("Conjunctivitis") OR ("HIV" OR "Human immuno deficiency virus") OR ("Acquired Immunodeficiency Syndrome" OR (AIDS or "immunodeficiency associated virus" or (immun* deficiency associated virus) or (acquired immunodeficiency syndrome*) or (acquired immun* deficiency syndrome*))) OR ("Buruli Ulcer" OR (Bairnsdale or Buruli)) OR ("Onchocerciasis" OR (onchocer*)) OR ("Hepatitis B" OR "Hepatitis C" OR (hepatitis)) OR ("Leishmaniasis" OR (leishmania*)) OR ("Leprosy" OR (lepros* or hansen*)) OR ("Elephantiasis, Filarial" OR (elephantias* or filaria*)) OR ("Trachoma" OR ((egyptian ophthalmia*) or trachoma*)) OR ("Chikungunya Fever" OR (chickungunya or chikungunya)) OR ("Taeniasis" OR taenia*) OR ("Cysticercosis" OR cysticercos*) OR ("Echinococcosis" OR (hydatid or echinococc*)) OR ("Chagas Disease" OR (trypanosom* or chagas)) OR ("Trypanosomiasis" OR ("sleeping sickness")) OR ("Encephalitis, Japanese" OR (japanese encephalitis)) OR ("Syphilis"))) AND ("Tuberculosis" OR Tuberculos* OR TB OR koch*)) AND ("Systematic Review" OR (systematic AND review*) OR "Meta-Analysis" OR meta-analys*)
131

OpenGrey (23/10/2020)

(((("Noncommunicable Diseases" OR ((("Non-communicable" or Noncommunicable or "Non-infectious") AND (disease* or condition* or illness*)))) OR ("Chronic Disease" OR (((chronic or "long-term") AND (disease* or condition* or illness*)))) OR ("Heart Diseases" OR ((heart AND (disease* or disorder* or failure)) or (cardiac AND (disease* or disorder* or failure)))) OR ("Cardiovascular Diseases" OR ((cardiovascular AND (disease* or disorder* or failure)))) OR ("Coronary Disease" OR (coronary AND (disease* or disorder* or failure))) OR ("Cerebrovascular Disorders" OR ((cerebrovascular AND (disease* or disorder* or insufficienc* or occlusion*)) or (vascular AND (disease* or disorder*)) or (carotid* AND (disease* or disorder*)))) OR ("Peripheral Arterial Disease" OR ((arter* AND (disease* or disorder*)))) OR ("Rheumatic Heart Disease" OR "Heart Defects, Congenital" OR ((heart AND (malform* or defect* or congeni*)))) OR ("Venous Thrombosis" OR ((("deep vein" or "deep venous") AND thrombos*) or phlebothrombos*)) OR ("Pulmonary Embolism" OR ((pulmonar* AND (thromboembolism* or embolism* or disease* or disorder*)))) OR ("Stroke" OR (stroke)) OR ("Neoplasms" OR (Cancer* or neoplas* or tumor*)) OR ("Lung Diseases" OR "Respiratory Tract Diseases" OR "Lung Diseases, Obstructive" OR (((lung* or respiratory or pulmonar* or airflow or airway) AND (disease* or obstruct* or hypersensitiv*))) OR "Asthma" OR asthma* OR "Pulmonary Disease, Chronic Obstructive" OR "Respiratory Hypersensitivity") OR ("Diabetes Mellitus" or diabet*) OR ("Autoimmune Diseases" OR ((autoimmun* or (auto AND immun*) or autoaggress* or (auto AND aggress*)) AND (disorder* or disease*))) OR ("Metabolic Syndrome" OR "Metabolic Diseases" OR (((metabolic or "insulin resistance") AND (disorder* or disease* or syndrome*)))) OR ("Obesity" OR obes*) OR ("Osteoporosis" OR (osteoporo* or "bone loss" or "Osteolysis" or osteolysis or "bone resorption")) OR ("Parkinson Disease" OR (parkinson* or "paralysis agitans")) OR ("Arthritis" OR (arthriti* or polyarthriti* or rheumarthriti*)) OR ("Kidney Diseases" OR ((kidney AND (disease* or disorder*)))) OR ("Liver Diseases" OR (liver AND (disease* or disorder* or dysfunction*))) OR ("Hypertension" OR ("high blood pressure" or hypertens*)) OR ("Hyperlipidemias" OR (hyperlipem* or hyperlipidem* or lipem* or lipidem*)) OR ("Hypercholesterolemia" OR (((high* or elevat*) AND cholesterol*) or hypercholesterem* or hypercholesterolem*)) OR ("Hypertriglyceridemia " OR (hypertriglyceridem*)) OR ("Thyroid Diseases" OR (thyroid AND (disease* or disorder*)) OR

"Hyperthyroidism " OR (hyperthyroid*) OR "Hypothyroidism" OR (hypothyroid*) OR ((("thyroid-stimulating hormone" or tsh) AND deficien*))) OR ("Motor Neuron Disease" OR ("motor neuron disease") OR (lateral AND scleros*) OR ("motor system disease")) OR ("Multiple Sclerosis" OR ("multiple sclerosis" or "disseminated sclerosis")) OR ("Emphysema" OR emphysema*) OR ("Bronchitis" OR bronchit*)) OR (("Mental Disorders" OR "Psychotic Disorders" OR (((mental* or psychiatr* or psycho*) AND (disorder* or disease* or illness*)))) OR ("Depressive Disorder, Major" OR "Depression" OR (Depress* or MDD)) OR ("Anxiety Disorders" OR "Anxiety" OR anxi*) OR ("Phobic Disorders" OR phobi*) OR ("Schizophrenia" Or (schizophreni* or hebephreni*)) OR ("Somatoform Disorders" OR "Medically Unexplained Symptoms" OR (((somatoform* or somati* or (medically AND unexplained) or briquet or pain) AND (disorder* or syndrome* or symptom*)))) OR ("Dissociative Disorders" OR (dissociative AND (disorder* or hysteri* or reaction*)) or dissociation*) OR ("Hysteria" or hysteri*) OR ("Mood Disorders" OR ((affective* or mood*) AND (disorder* or disease* or illness* or symptom*))) OR ("Stress Disorders, Post-Traumatic" OR (PTSD or ((posttrauma* or trauma*) AND (stress* or neurose*)) or "combat disorder" or "war disorder")) OR ("Cognition Disorders" OR (((cognitive or cognition or mental or neurocognitive) AND (dysfunction* or decline* or impairment* or deterioration* or disorder* or illness* or disease*)))) OR ("Personality Disorders" OR "personality disorder") OR ("Disruptive, Impulse Control, and Conduct Disorders" OR ("impulse control disorder" or "intermittent explosive disorder")) OR ("Feeding and Eating Disorders" OR ((eating or appetite or feeding) AND disorder*)) OR ("Bipolar Disorder" OR ((bipolar or mani*) AND (disorder* or illness* or disease*))) OR ("Obsessive-Compulsive Disorder" OR OCD* or ((obsess* or compulsi*) AND (disorder* or illness* or disease* or neuros*))) OR ("Panic Disorder" OR ((panic AND (attack* or disorder*)))) OR ("Agoraphobia" OR agoraphobi*) OR ("Neurotic Disorders" OR (neuros* or "neurotic disorder" or psychoneuros*))) OR (("Communicable Diseases" OR (((communic* or contag* or transmi* or infect*) AND (disease* or infection* or illness*)))) OR ("Bacterial Infections" OR (bacteri* infection*)) OR ("Conjunctivitis") OR ("HIV" OR "Human immuno deficiency virus") OR ("Acquired Immunodeficiency Syndrome" OR (AIDS or "immunodeficiency associated virus" or (immun* deficiency associated virus) or (acquired immunodeficiency syndrome*) or (acquired immun* deficiency syndrome*))) OR ("Buruli Ulcer" OR (Bairnsdale or Buruli)) OR ("Onchocerciasis" OR (onchocer*)) OR ("Hepatitis B" OR "Hepatitis C" OR (hepatitis)) OR ("Leishmaniasis" OR (leishmania*)) OR ("Leprosy" OR (lepros* or hansen*)) OR ("Elephantiasis, Filarial" OR (elephantias* or filaria*)) OR ("Trachoma" OR ((egyptian ophthalmia*) or trachoma*)) OR ("Chikungunya Fever" OR (chickungunya or chikungunya)) OR ("Taeniasis" OR taenia*) OR ("Cysticercosis" OR cysticercos*) OR ("Echinococcosis" OR (hydatid or echinococc*)) OR ("Chagas Disease" OR (trypanosom* or chagas)) OR ("Trypanosomiasis" OR ("sleeping sickness")) OR ("Encephalitis, Japanese" OR (japanese encephalitis)) OR ("Syphilis"))) AND ("Tuberculosis" OR Tuberculos* OR TB OR koch*)) AND ("Systematic Review" OR (systematic AND review*) OR "Meta-Analysis" OR meta-analys*)
3

For peer review only

PROSPERO (07/10/2020)

(((((Non-communicable or Noncommunicable or Non-infectious) AnD (disease* or condition* or illness*))) oR ((MeSH DESCRIPTOR Chronic Disease EXPLODE ALL TREES) or ((chronic or long-term) AnD (disease* or condition* or illness*))) oR ((MeSH DESCRIPTOR Heart Diseases EXPLODE ALL TREES) or ((heart AnD (disease* or disorder* or failure)) or (cardiac AnD (disease* or disorder* or failure)))) oR ((MeSH DESCRIPTOR Cardiovascular Diseases EXPLODE ALL TREES) OR ((cardiovascular AnD (disease* or disorder* or failure)))) oR ((MeSH DESCRIPTOR Coronary Disease EXPLODE ALL TREES) OR ((coronary AnD (disease* or disorder* or failure)))) oR ((MeSH DESCRIPTOR Cerebrovascular Disorders EXPLODE ALL TREES) OR ((cerebrovascular AnD (disease* or disorder* or insufficienc* or occlusion*))) OR ((vascular AnD (disease* or disorder*))) OR ((carotid* AnD (disease* or disorder*)))) oR ((MeSH DESCRIPTOR Peripheral Arterial Disease EXPLODE ALL TREES) OR ((arter* AnD (disease* or disorder*)))) oR ((MeSH DESCRIPTOR Rheumatic Heart Disease EXPLODE ALL TREES) OR (MeSH DESCRIPTOR Heart Defects, Congenital EXPLODE ALL TREES) or ((heart AnD (malform* or defect* or congeni*)))) oR ((MeSH DESCRIPTOR Venous Thrombosis EXPLODE ALL TREES) OR (((deep vein or deep venous) AnD thrombos*)) OR (phlebothrombos*)) oR ((MeSH DESCRIPTOR Pulmonary Embolism EXPLODE ALL TREES) OR ((pulmonar* AnD (thromboembolism* or embolism* or disease* or disorder*)))) oR ((MeSH DESCRIPTOR Stroke EXPLODE ALL TREES) OR (stroke)) oR ((MeSH DESCRIPTOR Neoplasms EXPLODE ALL TREES) OR (Cancer*) OR (neoplas*) OR (tumor*)) oR ((MeSH DESCRIPTOR Lung Diseases EXPLODE ALL TREES) OR (MeSH DESCRIPTOR Respiratory Tract Diseases EXPLODE ALL TREES) OR (MeSH DESCRIPTOR Lung Diseases, Obstructive EXPLODE ALL TREES) OR (((lung* or respiratory or pulmonar* or airflow or airway) AnD (disease* or obstruct* or hypersensitiv*))) OR (MeSH DESCRIPTOR Asthma EXPLODE ALL TREES) Or (asthma*) OR (MeSH DESCRIPTOR Pulmonary Disease, Chronic Obstructive EXPLODE ALL TREES) OR (MeSH DESCRIPTOR Respiratory Hypersensitivity EXPLODE ALL TREES)) oR ((MeSH DESCRIPTOR Diabetes Mellitus EXPLODE ALL TREES) OR (diabet*)) oR ((MeSH DESCRIPTOR Autoimmune Diseases EXPLODE ALL TREES) OR (((autoimmun* or auto immun* or autoaggress* or auto aggress*) AnD (disorder* or disease*)))) oR ((MeSH DESCRIPTOR Metabolic Syndrome X EXPLODE ALL TREES) OR (MeSH DESCRIPTOR Metabolic Diseases EXPLODE ALL TREES) OR (((metabolic or insulin resistance) AnD (disorder* or disease* or syndrome*)))) oR ((MeSH DESCRIPTOR Obesity EXPLODE ALL TREES) OR (obes*)) oR ((MeSH DESCRIPTOR Osteoporosis EXPLODE ALL TREES) OR (osteoporo* or bone loss) OR (MeSH DESCRIPTOR Osteolysis EXPLODE ALL TREES) OR (osteolysis or "bone resorption")) oR ((MeSH DESCRIPTOR Parkinson Disease EXPLODE ALL TREES) OR (parkinson* or "paralysis agitans")) oR ((MeSH DESCRIPTOR Arthritis EXPLODE ALL TREES) OR (arthriti* or polyarthriti* or rheumarthriti*)) oR ((MeSH DESCRIPTOR Kidney Diseases EXPLODE ALL TREES) OR ((kidney AnD (disease* or disorder*)))) oR ((MeSH DESCRIPTOR Liver Diseases EXPLODE ALL TREES) OR ((liver AnD (disease* or disorder* or dysfunction*)))) oR ((MeSH DESCRIPTOR Hypertension EXPLODE ALL TREES) OR (high blood pressure* or hypertens*)) oR ((MeSH DESCRIPTOR Hyperlipidemias EXPLODE ALL TREES) OR (hyperlipem* or hyperlipidem* or lipem* or lipidem*)) oR ((MeSH DESCRIPTOR Hypercholesterolemia EXPLODE ALL TREES) OR (((high* or elevat*) AnD cholesterol*)) OR (hypercholesterem* or hypercholesterolem*)) oR ((MeSH DESCRIPTOR Hypertriglyceridemia EXPLODE ALL TREES) OR (hypertriglyceridem*)) oR ((MeSH DESCRIPTOR Thyroid Diseases EXPLODE ALL TREES) OR ((thyroid AnD (disease* or disorder*))) OR (MeSH DESCRIPTOR Hyperthyroidism EXPLODE ALL TREES) OR (hypothyroid*) OR (((thyroid-stimulating hormone* or tsh) AnD deficien*))) oR ((MeSH DESCRIPTOR Motor Neuron Disease EXPLODE ALL TREES) OR (motor neuron* disease*) Or (lateral scleros*) OR (motor system disease*)) oR ((MeSH DESCRIPTOR Multiple Sclerosis EXPLODE ALL TREES) OR ("multiple sclerosis" or

For peer review only

"disseminated sclerosis")) oR ((MeSH DESCRIPTOR Emphysema EXPLODE ALL TREES) OR (emphysema*)) oR ((MeSH DESCRIPTOR Bronchitis EXPLODE ALL TREES) OR (bronchit*))) OR (((MeSH DESCRIPTOR Mental Disorders EXPLODE ALL TREES) OR (MeSH DESCRIPTOR Psychotic Disorders EXPLODE ALL TREES) OR (((mental* or psychiatr* or psycho*) AnD (disorder* or disease* or illness*)))) oR ((MeSH DESCRIPTOR Depressive Disorder, Major EXPLODE ALL TREES) OR (MeSH DESCRIPTOR Depression EXPLODE ALL TREES) OR (Depress* or MDD)) oR ((MeSH DESCRIPTOR Anxiety Disorders EXPLODE ALL TREES) oR (MeSH DESCRIPTOR Anxiety EXPLODE ALL TREES) OR (anxi*)) oR ((MeSH DESCRIPTOR Phobic Disorders EXPLODE ALL TREES) OR (phobi*)) oR ((MeSH DESCRIPTOR Schizophrenia EXPLODE ALL TREES) OR (schizophreni* or hebephreni*)) oR ((MeSH DESCRIPTOR Somatoform Disorders EXPLODE ALL TREES) OR (((somatoform* or somati* or "medically unexplained" or briquet or pain) AnD (disorder* or syndrome* or symptom*))) or (MeSH DESCRIPTOR Medically Unexplained Symptoms EXPLODE ALL TREES)) oR ((MeSH DESCRIPTOR Dissociative Disorders EXPLODE ALL TREES) OR ((dissociative AnD (disorder* or hysteri* or reaction*))) OR (dissociation*)) oR ((MeSH DESCRIPTOR Hysteria EXPLODE ALL TREES) OR (hysteri*)) oR ((MeSH DESCRIPTOR Mood Disorders EXPLODE ALL TREES) OR (((affective* or mood*) AnD (disorder* or disease* or illness* or symptom*)))) oR ((MeSH DESCRIPTOR Stress Disorders, Post-Traumatic EXPLODE ALL TREES) or (PTSD) OR (((post trauma* or posttrauma*) AnD (stress* or neurose*))) or ("combat disorder") OR ("war disorder")) oR ((MeSH DESCRIPTOR Cognition Disorders EXPLODE ALL TREES) OR (((cognitive or cognition or mental or neurocognitive) AnD (dysfunction* or decline* or impairment* or deterioration* or disorder* or illness* or disease*)))) oR ((MeSH DESCRIPTOR Personality Disorders EXPLODE ALL TREES) OR ("personality disorder") OR ("personality disorders")) oR ((impulse control disorder*) OR (intermittent explosive disorder*)) oR ((MeSH DESCRIPTOR Feeding and Eating Disorders EXPLODE ALL TREES) OR (((eating or appetite or feeding) AnD disorder*))) oR ((MeSH DESCRIPTOR Bipolar Disorder EXPLODE ALL TREES) OR (((bipolar or mani*) AnD (disorder* or illness* or disease*)))) oR ((MeSH DESCRIPTOR Obsessive-Compulsive Disorder EXPLODE ALL TREES) OR (OCD*) OR (((obsess*-compulsi* or obsess* or compulsi*) AnD (disorder* or illness* or disease* or neuros*)))) oR ((MeSH DESCRIPTOR Panic Disorder EXPLODE ALL TREES) OR ((panic AnD (attack* or disorder*)))) oR ((MeSH DESCRIPTOR Agoraphobia EXPLODE ALL TREES) OR (agoraphobi*)) oR ((MeSH DESCRIPTOR Neurotic Disorders EXPLODE ALL TREES) OR (neuros* or "neurotic disorder" or psychoneuros*))) OR (((MeSH DESCRIPTOR Communicable Diseases EXPLODE ALL TREES) OR (((communic* or contag* or transmi* or infect*) AnD (disease* or infection* or illness*)))) oR ((MeSH DESCRIPTOR Bacterial Infections EXPLODE ALL TREES) OR (bacteri* infection*)) oR ((MeSH DESCRIPTOR Conjunctivitis EXPLODE ALL TREES) OR (conjunctivitis)) oR ((MeSH DESCRIPTOR HIV EXPLODE ALL TREES) OR (hiv or "Human immuno deficiency virus")) oR ((MeSH DESCRIPTOR Acquired Immunodeficiency Syndrome EXPLODE ALL TREES) OR (AIDS) OR ("immunodeficiency associated virus") OR (immun* AND "deficiency associated virus") OR ("acquired immunodeficiency syndrome")) oR ((MeSH DESCRIPTOR Buruli Ulcer EXPLODE ALL TREES) OR (Bairnsdale or Buruli)) oR ((MeSH DESCRIPTOR Onchocerciasis EXPLODE ALL TREES) OR (onchocer*)) oR ((MeSH DESCRIPTOR Hepatitis B EXPLODE ALL TREES) OR (MeSH DESCRIPTOR Hepatitis C EXPLODE ALL TREES) OR (hepatitis)) oR ((MeSH DESCRIPTOR Leishmaniasis EXPLODE ALL TREES) OR (leishmania*)) oR ((MeSH DESCRIPTOR Leprosy EXPLODE ALL TREES) OR (lepros* or hansen*)) oR ((MeSH DESCRIPTOR Elephantiasis, Filarial EXPLODE ALL TREES) OR (elephantias* or filaria*)) oR ((MeSH DESCRIPTOR Trachoma EXPLODE ALL TREES) OR ("egyptian ophthalmia" or trachoma*)) oR ((MeSH DESCRIPTOR Chikungunya Fever EXPLODE ALL TREES) OR (chickungunya or chikungunya)) oR ((MeSH DESCRIPTOR Taeniasis EXPLODE ALL TREES) OR taenia*) oR ((MeSH DESCRIPTOR Cysticercosis EXPLODE ALL TREES) OR (cysticercos*)) oR ((MeSH DESCRIPTOR Echinococcosis EXPLODE ALL TREES)

For peer review only

OR (hydatid* or echinococc*)) oR ((MeSH DESCRIPTOR Chagas Disease EXPLODE ALL TREES) OR (trypanosom* or chagas)) oR ((MeSH DESCRIPTOR Trypanosomiasis EXPLODE ALL TREES) OR ("sleeping sickness")) oR ((MeSH DESCRIPTOR Encephalitis, Japanese EXPLODE ALL TREES) OR (japanese AnD encephalitis)) oR ((MeSH DESCRIPTOR Syphilis EXPLODE ALL TREES) OR (syphilis)))) AND ((MeSH DESCRIPTOR Tuberculosis EXPLODE ALL TREES) OR (Tuberculos*) OR (TB) OR (koch*))
1341

For peer review only

Supplemental material

Supplementary Table 1: Search terms used in Medline (run on October 23rd, 2020)

	Searches
1	exp Noncommunicable Diseases/ or ((Non-communicable or Noncommunicable or Non-infectious) adj (disease* or condition* or illness*)).mp.
2	exp Chronic Disease/ or ((chronic or long-term) adj (disease* or condition* or illness*)).mp.
3	exp Heart Diseases/ or (heart adj (disease* or disorder* or failure)).mp. or (cardiac adj (disease* or disorder* or failure)).mp.
4	exp Cardiovascular Diseases/ or (cardiovascular adj (disease* or disorder* or failure)).mp.
5	exp Coronary Disease/ or (coronary adj (disease* or disorder* or failure)).mp.
6	exp Cerebrovascular Disorders/ or (cerebrovascular adj (disease* or disorder* or insufficienc* or occlusion*)).mp. or (vascular adj (disease* or disorder*)).mp. or (carotid* adj (disease* or disorder*)).mp.
7	exp Peripheral Arterial Disease/ or (arter* adj (disease* or disorder*)).mp.
8	exp Rheumatic Heart Disease/ or exp Heart Defects, Congenital/ or (heart adj3 (malform* or defect* or congeni*)).mp.
9	exp Venous Thrombosis/ or ((deep vein or deep venous) adj thrombos*).mp. or phlebothrombos*.mp.
10	exp Pulmonary Embolism/ or (pulmonar* adj (thromboembolism* or embolism* or disease* or disorder*)).mp.
11	exp Stroke/ or stroke.mp.
12	exp Neoplasms/ or Cancer*.mp. or neoplas*.mp. or tumor*.mp.
13	exp Lung Diseases/ or exp Respiratory Tract Diseases/ or exp Lung Diseases, Obstructive/ or ((lung* or respiratory or pulmonar* or airflow or airway) adj2 (disease* or obstruct* or hypersensitiv*)).mp. or exp Asthma/ or asthma*.mp. or exp Pulmonary Disease, Chronic Obstructive/ or exp Respiratory Hypersensitivity/
14	exp Diabetes Mellitus/ or diabet*.mp.
15	exp Autoimmune Diseases/ or ((autoimmun* or auto immun* or autoaggress* or auto aggress*) adj (disorder* or disease*)).mp.
16	exp Metabolic Syndrome/ or exp Metabolic Diseases/ or ((metabolic or insulin resistance) adj (disorder* or disease* or syndrome*)).mp.
17	exp Obesity/ or obes*.mp.
18	exp Osteoporosis/ or osteoporo*.mp. or bone loss.mp. or exp osteolysis/ or osteolysis.mp. or bone resorption.mp.
19	exp Parkinson disease/ or parkinson*.mp. or paralysis agitans.mp.
20	exp Arthritis/ or arthriti*.mp. or polyarthriti*.mp. or rheumarthriti*.mp.
21	exp Kidney Diseases/ or (kidney adj (disease* or disorder*)).mp.
22	exp Liver Diseases/ or (liver adj (disease* or disorder* or dysfunction*)).mp.
23	exp Hypertension/ or high blood pressure*.mp. or hypertens*.mp.
24	exp Hyperlipidemias/ or hyperlipem*.mp. or hyperlipidem*.mp. or lipem*.mp. or lipidem*.mp.
25	exp Hypercholesterolemia/ or ((high* or elevat*) adj cholesterol*).mp. or hypercholesterem*.mp. or hypercholesterolem*.mp.
26	exp Hypertriglyceridemia/ or hypertriglyceridem*.mp.
27	exp Thyroid Diseases/ or (thyroid adj (disease* or disorder*)).mp. or exp Hyperthyroidism/ or hyperthyroid*.mp. or exp Hypothyroidism/ or hypothyroid*.mp. or ((thyroid-stimulating hormone* or tsh) adj deficien*).mp.
28	exp Motor Neuron Disease/ or motor neuron* disease*.mp. or lateral scleros*.mp. or motor system disease*.mp.
29	exp Multiple Sclerosis/ or multiple sclerosis.mp. or disseminated sclerosis.mp.
30	exp Emphysema/ or emphysema*.mp.
31	exp Bronchitis/ or bronchit*.mp.
32	1 or 2 or 3 or 4 or 5 or 6 or 7 or 8 or 9 or 10 or 11 or 12 or 13 or 14 or 15 or 16 or 17 or 18 or 19 or 20 or 21 or 22 or 23 or 24 or 25 or 26 or 27 or 28 or 29 or 30 or 31
33	exp Mental Disorders/ or exp Psychotic Disorders/ or ((mental* or psychiatr* or psycho*) adj (disorder* or disease* or illness*)).mp.
34	exp Depressive Disorder, Major/ or exp Depression/ or Depress*.mp. or MDD.mp.
35	exp Anxiety Disorders/ or exp Anxiety/ or anxi*.mp.

36	exp Phobic Disorders/ or phobi*.mp.
37	exp Schizophrenia/ or schizophreni*.mp. or hebephreni*.mp.
38	exp Somatoform Disorders/ or ((somatoform* or somati* or medically unexplained or briquet or pain) adj (disorder* or syndrome* or symptom*)).mp. or exp Medically Unexplained Symptoms/
39	exp Dissociative Disorders/ or (dissociative adj (disorder* or hysteri* or reaction*)).mp. or dissociation*.mp.
40	exp Hysteria/ or hysteri*.mp.
41	exp Mood Disorders/ or ((affective* or mood*) adj (disorder* or disease* or illness* or symptom*)).mp.
42	exp Stress Disorders, Post-Traumatic/ or PTSD.mp. or ((post trauma* or posttrauma*) adj (stress* or neurose*)).mp. or combat disorder*.mp. or war disorder*.mp.
43	exp Cognition Disorders/ or ((cognitive or cognition or mental or neurocognitive) adj (dysfunction* or decline* or impairment* or deterioration* or disorder* or illness* or disease*)).mp.
44	exp Personality Disorders/ or personality disorder*.mp.
45	exp "Disruptive, Impulse Control, and Conduct Disorders"/ or impulse control disorder*.mp. or intermittent explosive disorder*.mp.
46	exp "Feeding and Eating Disorders"/ or ((eating or appetite or feeding) adj disorder*).mp.
47	exp Bipolar Disorder/ or ((bipolar or mani*) adj (disorder* or illness* or disease*)).mp.
48	exp Obsessive-Compulsive Disorder/ or OCD*.mp. or ((obsess*-compulsi* or obsess* or compulsi*) adj (disorder* or illness* or disease* or neuros*)).mp.
49	exp Panic Disorder/ or (panic adj (attack* or disorder*)).mp.
50	exp Agoraphobia/ or agoraphobi*.mp.
51	exp Neurotic Disorders/ or neuros*.mp. or neurotic disorder*.mp. or psychoneuros*.mp.
52	33 or 34 or 35 or 36 or 37 or 38 or 39 or 40 or 41 or 42 or 43 or 44 or 45 or 46 or 47 or 48 or 49 or 50 or 51
53	exp Communicable Diseases/ or ((communic* or contag* or transmi* or infect*) adj (disease* or infection* or illness*)).mp.
54	exp Bacterial Infections/ or bacteri* infection*.mp.
55	exp Conjunctivitis/ or conjunctivitis.mp.
56	exp HIV/ or hiv.mp. or Human immuno deficiency virus.mp.
57	exp Acquired Immunodeficiency Syndrome/ or AIDS.mp. or immunodeficiency associated virus.mp. or immun* deficiency associated virus.mp. or acquired immunodeficiency syndrome*.mp. or acquired immun* deficiency syndrome*.mp.
58	exp Buruli Ulcer/ or Bairnsdale.mp. or Buruli.mp.
59	exp Onchocerciasis/ or onchocer*.mp.
60	hepatitis.mp. or exp Hepatitis B/ or exp Hepatitis C/
61	exp Leishmaniasis/ or leishmania*.mp.
62	exp Leprosy/ or lepros*.mp. or hansen*.mp.
63	exp Elephantiasis, Filarial/ or elephantias*.mp. or filaria*.mp.
64	exp Trachoma/ or egyptian ophthalmia*.mp. or trachoma*.mp.
65	exp Chikungunya Fever/ or chickungunya.mp. or chikungunya.mp.
66	exp Taeniasis/ or taenia*.mp.
67	exp Cysticercosis/ or cysticercos*.mp.
68	exp Echinococcosis/ or hydatid*.mp. or echinococc*.mp.
69	exp Chagas Disease/ or trypanosom*.mp. or chagas.mp.
70	exp Trypanosomiasis/ or sleeping sickness.mp.
71	exp Encephalitis, Japanese/ or (japanese adj3 encephalitis).mp.
72	exp Syphilis/ or syphilis.mp.
73	53 or 54 or 55 or 56 or 57 or 58 or 59 or 60 or 61 or 62 or 63 or 64 or 65 or 66 or 67 or 68 or 69 or 70 or 71 or 72
74	exp Tuberculosis/
75	Tuberculos*.mp.
76	TB.mp.

For peer review only - http://bmjopen.bmj.com/site/about/guidelines.xhtml

77	koch*.mp.
78	exp Tuberculosis/ or Tuberculos*.mp. or TB.mp. or koch*.mp.
79	(multiple adj (ill* or disease* or condition* or syndrom* or disorder*)).mp.
80	((Cooccur* or co-occur* or coexist* or co-exist* or multipl* or concord* or discord* or long-term or physical*) adj3 (disease* or ill* or care or condition* or disorder* or health* or medication* or symptom* or syndrom*)).mp.
81	(comorbid* or multimorbid* or co-occurren* or co-morbid* or Multidisease* or multi-disease*).mp.
82	(comorbid* or multimorbid* or co-occurren* or co-morbid* or multi-morbid* or Multidisease* or multi-disease*).mp.
83	exp Comorbidity/ or exp Multimorbidity/ or exp Multiple Chronic Conditions/
84	79 or 80 or 81 or 82 or 83
85	exp "Systematic Review"/
86	"systematic review*".m_titl.
87	exp Meta-Analysis/
88	"meta-analys*".m_titl.
89	exp "Systematic Review"/ or "systematic review*".m_titl. or exp Meta-Analysis/ or "meta-analys*".m_titl.
90	32 or 52 or 73
91	(32 or 52 or 73) and 78
92	(32 or 52 or 73) and 78 and 84
93	(32 or 52 or 73) and 78 and 84 and 89
94	exp Animals/ not exp Humans/
95	((32 or 52 or 73) and 78 and 84) not 94
96	((32 or 52 or 73) and 78 and 84 and 89) not 94
97	((32 or 52 or 73) and 78 and 89) not 94

review only

Supplementary Table 2. Reasons for exclusion of references assessed in full text.

**Wrong population**	
1.	Shivakoti, Rupak and Sharma, Davina and Mamoon, Gabeena and Pham, Kiemanh. Association of HIV infection with extrapulmonary tuberculosis: a systematic review. Infection, 2017; 45(1):44501.
2.	Nasiri, Mohammad Javad and Dabiri, Hossein and Darban-Sarokhalil, Davood and Hashemi Shahraki, Abdolrazagh. Prevalence of Non-Tuberculosis Mycobacterial Infections among Tuberculosis Suspects in Iran: Systematic Review and Meta-Analysis. PloS one, 2015; 10(6):e0129073.
3.	Yaghoubi A. and Salehabadi S. and Abdeahad H. and Hasanian S.M. and Avan A. and Yousefi M. and Jamehdar S.A. and Ferns G.A. and Khazaei M. and Soleimanpour S. Tuberculosis, human immunodeficiency viruses and TB/HIV co-infection in pregnant women: A meta-analysis. Clinical Epidemiology and Global Health, 2020; 8(4):1312-1320.
4.	Manrique, RD and CastaÃ±eda, HL. RelaciÃ³n entre haber tenido tuberculosis y desarrollar enfermedad pulmonar obstructiva crÃ³nica. RevisiÃ³n sistemÃ¡tica y meta anÃ¡lisis. Med. U.P.B, 2015; 34(2):115-125.
5.	Fadaee, Manouchehr and Rashedi, Jalil and Arabi, Sepideh and Poor, Behroz Mahdavi and Kafil, Hossein Samadi and Pourostadi, Mahya and Kazemi, Abdolhassan and Asgharzadeh, Mohammad. Stopping of the Downtrend of Tuberculosis in Iran, a Systematic Review of Associated Risk Factors. Infectious disorders drug targets, 2020; 20(3):367-373.
6.	Bailey, S L and Ayles, H. Association between diabetes mellitus and active tuberculosis in Africa and the effect of HIV. Tropical medicine & international health : TM & IH, 2017; 22(3):261-268.
7.	Berhan, Asres and Berhan, Yifru and Yizengaw, Desalegn. A meta-analysis of drug resistant tuberculosis in Sub-Saharan Africa: how strongly associated with previous treatment and HIV co-infection?. Ethiopian journal of health sciences, 2013; 23(3):271-82.
8.	Gao Y. and Liu M. and Chen Y. and Shi S. and Geng J. and Tian J. Association between tuberculosis and COVID-19 severity and mortality: A rapid systematic review and meta-analysis. Journal of Medical Virology, 2020; ():.
9.	Berhan A. and Berhan Y. and Yizengaw D. A meta-analysis of drug resistant tuberculosis in Sub-Saharan Africa: how strongly associated with previous treatment and HIV co-infection?. Ethiopian journal of health sciences, 2013; 23(3):271-282.
10.	Judith Udeh, Jabulani Ncayiyana, Sibusiso Mkwananzi. The prevalence of latent tuberculosis infection and associated risk factors among young people in South Africa: a systematic review and meta-analysis.
11.	Demeke Geremew. Tuberculosis and its association with ART initiation in HIV patients in Ethiopia.
12.	Eleni Seyoum, Alemayehu Worku. The effect of co-infection (hepatitis b or c viruses & tuberculosis) on the outcome of antiretroviral therapy in resource limited countries: systematic review and meta-analysis.
13.	Diana Ramos, Ana Carolina Fragoso Motta, Alan Grupioni LourenÃ§o, Lara Maria Alencar Ramos Innocentini, Maria ConceiÃ§Ã£o Pereira Saraiva. Influence of HIV and tuberculosis co-infection on the occurrence of oral candidiasis: a systematic review and meta analysis.
**Does not report data on people with TB+1 chronic condition**	
14.	Oga-Omenka, Charity and Tseja-Akinrin, Azhee and Sen, Paulami and Mac-Seing, Muriel and Agbaje, Aderonke and Menzies, Dick and Zarowsky, Christina. Factors influencing diagnosis and treatment initiation for multidrug-resistant/rifampicin-resistant tuberculosis in six sub-Saharan African countries: a mixed-methods systematic review. BMJ global health, 2020; 5(7):.
15.	Tamuzi, Jacques L and Ayele, Birhanu T and Shumba, Constance S and Adetokunboh, Olatunji O and Uwimana-Nicol, Jeannine and Haile, Zelalem T and Inugu, Joseph and Nyasulu, Peter S. Implications of COVID-19 in high burden countries for HIV/TB: A systematic review of evidence. BMC infectious diseases, 2020; 20(1):744.
16.	Walaza, Sibongile and Cohen, Cheryl and Tempia, Stefano and Moyes, Jocelyn and Nguweneza, Athermon and Madhi, Shabir A and McMorrow, Meredith and Cohen, Adam L. Influenza and tuberculosis co-infection: A systematic review. Influenza and other respiratory viruses, 2020; 14(1):77-91.
17.	Tola, Habteyes Hailu and Holakouie-Naieni, Kourosh and Lejisa, Tadesse and Mansournia, Mohammad Ali and Yaseri, Mehdi and Tesfaye, Ephrem and Mola, Million. Is hypothyroidism rare in multidrug resistance tuberculosis patients on treatment? A systematic review and meta-analysis. PloS one, 2019; 14(6):e0218487.
18.	Nliwasa, Marriott and MacPherson, Peter and Gupta-Wright, Ankur and Mwapasa, Mphatso and Horton, Katherine and Odland, Jon O and Flach, Clare and Corbett, Elizabeth L. High HIV and active tuberculosis prevalence and increased mortality risk in adults with symptoms of TB: a systematic review and meta-analyses. Journal of the International AIDS Society, 2018; 21(7):e25162.
19.	Seegert, Anneline Borchsenius and Rudolf, Frauke and Wejse, Christian and Neupane, Dinesh. Tuberculosis and hypertension-a systematic review of the literature. International journal of infectious diseases : IJID : official publication of the International Society for Infectious Diseases, 2017; 56():54-61.
20.	Wu, Shan-shan and Zhang, Yue-lun and Wang, Wei-wei and Chen, Ru and Sun, Feng and Zhan, Si-yan. [Liver injury associated with treatment of multidrug-resistant tuberculosis: a systematic review and meta-analysis]. Beijing da xue xue bao. Yi xue ban = Journal of Peking University. Health sciences, 2014; 46(3):417-23.
21.	Patra, Jayadeep and Jha, Prabhat and Rehm, Jurgen and Suraweera, Wilson. Tobacco smoking, alcohol drinking, diabetes, low body mass index and the risk of self-reported symptoms of active tuberculosis: individual participant data (IPD) meta-analyses of 72,684 individuals in 14 high tuberculosis burden countries. PloS one, 2014; 9(5):e96433.
22.	Li, Xin-Xu and Zhou, Xiao-Nong. Co-infection of tuberculosis and parasitic diseases in humans: a systematic review. Parasites & vectors, 2013; 6(101462774):79.
23.	Sathiyamoorthy R. and Kalaivani M. and Aggarwal P. and Gupta S.K. Prevalence of pulmonary tuberculosis in India: A systematic review and meta-analysis. Lung India, 2020; 37(1):45-52.
24.	Zaidi A.K.M. and Awasthi S. and DeSilva H.J. Burden of infectious diseases in South Asia. British Medical Journal, 2004; 328(7443):811-815.
25.	Mortazavi, Hamed and Ghazalibina, Mehran and Mansouri, Shamseddin and Khaledi, Azad and Saburi, Ehsan. Pulmonary Fungal Co-Infection Prevalence among Iranian Patients with Pulmonary Tuberculosis: A Systematic Review and Meta-Analysis. SAINS MALAYSIANA, 2019; 48(12):2717-2725.
26.	Tanya Diefenbach-Elstob, Patricia Graves, Emma McBryde, David Plummer, Jeffrey Warner. Extrapulmonary tuberculosis in a global context: a systematic review of epidemiology and risk factors.
27.	Worku Jimma, Ahmed Abdulahi. Prevalence and risk factors of multidrug-resistant tuberculosis in Iran and its neighboring countries: systematic review and meta-analysis.

For peer review only - http://bmjopen.bmj.com/site/about/guidelines.xhtml

28.	Simon Collin, Dominik Zenner, Ibrahim Abubakar, Marieke van der Werf, Sarah Anderson, Gerard de Vries, Knut Lonnroth, Emily Newton. Effectiveness of interventions for TB control and prevention in countries of low and medium TB incidence: a systematic review of reviews.
29.	Yohannes Gezahagn, Yasin Mohammed, Alemseged Abdissa, Sophia Hussien, Guday Emire, Mirgissa Kaba. Delays in the diagnosis and treatment of tuberculosis in Ethiopia: a systematic review.
30.	Victor Vega, Sharon Rodriguez, Larissa Otero, Carlos Seas. Systematic review and meta-analysis on recurrent tuberculosis and associated risk factors.
31.	Mahdi Afshari, Bagheri, Saeed Barzegari. A protocol for estimating the risk factors of treatment default among patients with tuberculosis using systematic review and meta-analysis.
32.	Nguyen Tien Huy, Hieu Truong Hong, Mohammad Rashidul Hashan, Hazem Faraj, Tran Thuy Huong Quynh, Ahmed Saber Abdelrahman, Ahmad Morad, Khaled Mohy Ismaeil, Mohamed Gomaa Kamel. Hyponatremia in tubercular meningitis: a systematic review and meta-analysis.
33.	Trent Herdman, Justine Zhang, Sumona Datta, Matthew Saunders, Marco Tovar, Rosario Montoya, Carlton Evans. Clinical characteristics of unrecognised pulmonary tuberculosis detected through comprehensive population-based prevalence surveys: a systematic review.
34.	Olena Ivanova, Verena Hoffmann, Celso Khosa, Jule Witzleb, Michael Hoelscher, Andrea Rachow. Pulmonary outcomes in patients with tuberculosis: systematic review and meta-analysis.
35.	Shamanthi Jayasooriya, Caroline Mitchell. A systematic review of what proportion of adult patients attending TB treatment programmes in Africa have been identified as not having active tuberculosis?.
36.	Petrus Kosmas, Elize Pietersen, Jabulani Ncayiyana, Mark Engel. Extensively drug resistant tuberculosis in Africa; prevalence and factors associated; a systematic review and meta-analysis.
37.	Rodney Ehrlich, Paula Akugizibwe, Nandi Siegfried, David Rees. Systematic review of association between silica/silicosis and tuberculosis.
38.	Saravanan N, Silambhu Chelvi Ramesh, Rajesh Mondal, Banu Rekha, Basilea Watson, Rajendran Krishnan, Kannan Thiruvengadam. Nutritional status of patients with tuberculosis among the tribal population in India.
39.	Philippe Armel Awana, Celestin Danwang, Joel Noutakdie Tochie, Jean Joel Bigna. Global epidemiology of venous thromboembolism in people with active tuberculosis: a systematic review and meta-analysis.
40.	Sally Hayward, Rachel Wittenberg, Lisa Stockdale. Systematic review of the relationship between Herpesviridae infections and tuberculosis.
41.	Linh Nguyen, Quyen Bui. The incidence rate of multidrug-resistant Tuberculosis/ Rifampicin-resitant Tuberculosis and associated factors: a meta-analysis.
42.	Mpho Refilwe Disang, Christine Campbell, David Weller. Prevalence and patterns of communicable and noncommunicable diseases multimorbidity in sub-Saharan Africa; a protocol for systematic review.
43.	Tamuzi Lukenze Jacques, Peter Nyasulu, Ayele Birhanu. COVID-19 impact on HIV and tuberculosis programmes: a systematic review and meta-synthesis of evidence.
44.	Sara Hussein, May Al-Asmar, Ahmed Awaisu, Yaw Owusu, Alaa Soliman, Sara Murshid, Muna Al-Maslmani, Faraj Howady. Prevalence and Risk Factors of Drug Resistant Tuberculosis in the Eastern Mediterranean Region: A Systematic Review.
45.	Nannan Wang, Jia Zhu, Wenpei Liu, Miaomiao Yang, Honggang Yi, Shaowen Tang. Incidence, temporal trend and factors associated with anti-tuberculosis drug-induced liver injury: A systematic review and meta-analysis.
46.	Tasmiya Ira, Elizabeth Ojewole, Pravina Laljeeth, Richard Beharilal. Determinants of adverse drug reactions due to tuberculosis therapy in African countries: a systematic review.
47.	Danwang, Celestin and Bigna, Jean Joel and Awana, Armel Philippe and Nzalie, Rolf Nyah-Tuku and Robert, Annie. Global epidemiology of venous thromboembolism in people with active tuberculosis: a systematic review and meta-analysis. Journal of thrombosis and thrombolysis, 2020; ():.
48.	Dessie, Getenet and Negesse, Ayenew and Wagnew, Fasil and Amare, Desalegne and Tiruneh, Balew Zeleke and Mulugeta, Henok and Mekonen, Berhanu Abebaw and Haile, Dessalegn and Ayalew, Tilksew and Habtewold, Tesfa Dejenie. Intestinal parasites and HIV in Ethiopian tuberclosis patients: A systematic review and meta-analysis. Current therapeutic research, clinical and experimental, 2020; 93():100603.
49.	Hadadi-Fishani, Mehdi and Shakerimoghaddam, Ali and Khaledi, Azad. Candida coinfection among patients with pulmonary tuberculosis in Asia and Africa; A systematic review and meta-analysis of cross-sectional studies. Microbial pathogenesis, 2020; 139():103898.
50.	Alemu, Ayinalem and Bitew, Zebenay Workneh and Worku, Teshager. Intestinal parasites co-infection among tuberculosis patients in Ethiopia: a systematic review and meta-analysis. BMC infectious diseases, 2020; 20(1):510.
51.	Barzegari, Saeed and Afshari, Mahdi and Movahednia, Mahtab and Moosazadeh, Mahmood. Prevalence of anemia among patients with tuberculosis: A systematic review and meta-analysis. The Indian journal of tuberculosis, 2019; 66(2):299-307.
52.	Girum, Tadele and Muktar, Ebrahim and Lentiro, Kifle and Wondiye, Habtamu and Shewangizaw, Misgun. Epidemiology of multidrug-resistant tuberculosis (MDR-TB) in Ethiopia: a systematic review and meta-analysis of the prevalence, determinants and treatment outcome. Tropical diseases, travel medicine and vaccines, 2018; 4(101674442):5.
53.	Keflie, Tibebeselassie Seyoum and Nolle, Nils and Lambert, Christine and Nohr, Donatus and Biesalski, Hans Konrad. Vitamin D deficiencies among tuberculosis patients in Africa: A systematic review. Nutrition (Burbank, Los Angeles County, Calif.), 2015; 31(10):1204-12.
54.	Allwood, Brian W and Myer, Landon and Bateman, Eric D. A systematic review of the association between pulmonary tuberculosis and the development of chronic airflow obstruction in adults. Respiration; international review of thoracic diseases, 2013; 86(1):76-85.
55.	Azzeri A. and Ching G.H. and Jaafar H. and Noor M.I.M. and Razi N.A. and Then A.Y.-H. and Suhaimi J. and Kari F. and Dahlui M. A review of published literature regarding health issues of coastal communities in Sabah, Malaysia. International Journal of Environmental Research and Public Health, 2020; 17(5):1533.
56.	Hosseini M. and Shakerimoghaddam A. and Ghazalibina M. and Khaledi A. Aspergillus coinfection among patients with pulmonary tuberculosis in Asia and Africa countries; A systematic review and meta-analysis of cross-sectional studies. Microbial Pathogenesis, 2020; 141():104018.
57.	Cormier M. and Schwartzman K. and N'Diaye D.S. and Boone C.E. and dos Santos A.M. and Gaspar J. and Cazabon D. and Ghiasi M. and Kahn R. and Uppal A. and Morris M. and Oxlade O. Proximate determinants of tuberculosis in Indigenous peoples worldwide: a systematic review. The Lancet Global Health, 2019; 7(1):e68-e80.

For peer review only - http://bmjopen.bmj.com/site/about/guidelines.xhtml

58.	Azhar G. DOTS for TB relapse in India: A systematic review. Lung India, 2012; 29(2):147-153.
59.	Flannery E. and Rosenthal M. and Roberts E.R. and Henderson J.J. and Davis B.M. and Foxman B. Synergy and anergy in the context of infectious disease epidemiology: The role of co-infection. American Journal of Epidemiology, 2011; 173():S234.
60.	Khadija Ibrahim, Nan Shwe Htun, Karim Manji, Peter Odermatt, Marcel Tanner, JÃ¼rg Utzinger, Nicole Probst Hensch, Lukas Fenner. Does the association between helminths and tuberculosis have influence on each other?.
61.	Katherine Horton, Peter MacPherson, Richard White, Rein Houben, Liz Corbett. Gender differences in tuberculosis prevalence in low- and middle-income countries.
62.	Dalya Eltayeb, Elize Pietersen, Mark Engel, Leila Abdullahi. Factors associated with patient and health system delay in diagnosis and treatment for pulmonary tuberculosis in Middle East and North Africa (MENA).
63.	Erick Bunyasi, Leila Abdullahi, Bey-Marrie Schmidt, Hennie Geldenhuys, Robin Wood, Mark Hatherill. A systematic review on the prevalence and incidence of tuberculosis disease and infection prevention and control measures among high school students in 22 high tuberculosis disease burden countries defined by the World Health Organisation.
64.	Anthony Byrne, Ben Marais, Carole Mitnick, Leonid Lecca, Guy Marks. Tuberculosis and asthma: a systematic review.
65.	Amare Tariku, Destaw Fetene. The effect of under-nutrition on non-adherence to anti-TB drugs in TB patients in Sub-Saharan Africa: a systematic review and meta-analysis.
66.	Habteyes Tola, Kourosh Holakouie-Naieni, Ephrem Tesfaye, Mohammad Mansournia, Mehdi Yaseri. Tuberculosis treatment interruption and its associated factors in Ethiopia: a systematic review and meta-analysis.
67.	Ahmed Hossain, Zeeba Sultana, Farhana Hoque. Association between HIV infection and multidrug-resistant tuberculosis in Africa and Asia: a systematic review and meta-analysis.
68.	Ayinalem Alemu Shitie, Zebenay Workneh Bitew, Teshager Worku. Intestinal parasites co-infection among tuberculosis patients in Ethiopia: a systematic review and meta-analysis.
69.	Birhan Alemnew, Setegn Eshetie, Asmamaw Demis. The prevalence and its associated risk factors of extra pulmonary tuberculosis in Ethiopia; systematic review and meta-analysis.
70.	Getu Diriba, Habteyes Tola, Ayinalem Alemu, Abebaw Kebede. Prevalence of drug resistance and its risk factors among extra pulmonary tuberculosis patients in Ethiopia: a systematic review and meta-analysis.
71.	Balew Arega. Prevalence rate of undiagnosed tuberculosis in the community in Ethiopia from 2001 to 2014: systematic review and meta-analysis.
72.	Kerri Viney, Luis Furuya-Kanamori, Kefyalew Addis Alene, Kinley Wangdi, Anthony Byrne, Justin Clark. Types and burden of disabilities associated with tuberculosis: protocol for a systematic review.
73.	Getahun Molla Kassa, Atalay Goshu Muluneh, Dawit Tefera Fentie, Mehari Woldemariam Merid. Magnitude and effect of anemia on treatment outcome of drug-resistance tuberculosis in Sub-Sahara Africa: systematic review and meta-analysis.
74.	Adam Wondmieneh, Getnet Gedefaw, Addisu Getie, Asmamaw Demis. Undernutrition among adult tuberculosis patients in Ethiopia: A Systematic review and meta- analysis.
75.	Badawi A. and Gregg B. and Vasileva D.. Systematic analysis for the relationship between obesity and tuberculosis. Public Health, 2020; 186():246-256.
**Does not report data on any of our outcomes of interest**	
76.	Lohiya, Ayush and Suliankatchi Abdulkader, Rizwan and Rath, Rama Shankar and Jacob, Olivia and Chinnakali, Palanivel and Goel, Akhil Dhanesh and Agrawal, Sumita. Prevalence and patterns of drug resistant pulmonary tuberculosis in India-A systematic review and meta-analysis. Journal of global antimicrobial resistance, 2020; 22(101622459):308-316.
77.	Noykhovich, Ekaterina and Mookherji, Sangeeta and Roess, Amira. The Risk of Tuberculosis among Populations Living in Slum Settings: a Systematic Review and Meta-analysis. Journal of urban health : bulletin of the New York Academy of Medicine, 2019; 96(2):262-275.
78.	Tola, Habteyes Hailu and Tol, Azar and Shojaeizadeh, Davoud and Garmaroudi, Gholamreza. Tuberculosis Treatment Non-Adherence and Lost to Follow Up among TB Patients with or without HIV in Developing Countries: A Systematic Review. Iranian journal of public health, 2015; 44(1):44501.
79.	Naing, Cho and Mak, Joon Wah and Maung, Mala and Wong, Shew Fung and Kassim, Ani Izzuani Binti Mohd. Meta-analysis: the association between HIV infection and extrapulmonary tuberculosis. Lung, 2013; 191(1):27-34.
80.	Tengan F.M. and Figueiredo G.M. and Leite O.H.M. and Nunes A.K.S. and Manchiero C. and Dantas B.P. and Magri M.C. and Barone A.A. and Bernardo W.M. Prevalence of multidrug-resistant tuberculosis in Latin America and the Caribbean: a systematic review and meta-analysis. Tropical Medicine and International Health, 2020; 25(9):1065-1078.
81.	Xia Y.Y. and Zhan S.Y. Systematic review of anti-tuberculosis drug induced adverse reactions in China. Zhonghua jie he he hu xi za zhi = Zhonghua jiehe he huxi zazhi = Chinese journal of tuberculosis and respiratory diseases, 2007; 30(6):419-423.
82.	Perez-Guzman C. and Vargas M.H. and Torres-Cruz A. and Villarreal-Velarde H. Does aging modify pulmonary tuberculosis?: A meta-analytical review. Chest, 1999; 116(4):961-967.
83.	Mhlengi Vella Ncube, Tivani Mashamba-Thompson. Evidence on implementation of HIV/AIDS, STIs and TB related point of care diagnostics in low and medium income countries.
84.	Marie Varughese, Michael Li, Courtney Heffernan. Time to diagnosis and treatment of pulmonary tuberculosis in indigenous peoples: a systematic review.
85.	Md Asiful Islam, Mahfuza Marzan, Shoumik Kundu. Prevalence of antibiotic-resistant pulmonary tuberculosis in Bangladesh: A systematic review and meta-analysis.
86.	Balewgizie Sileshi Tegegne, Tesfa Dejenie Habtewold, Melkamu Merid Mengesha, Hans Burgerhof. Diabetes mellitus and multi-drug resistant tuberculosis: a protocol for a systematic review and meta-analysis.
87.	Benjamin Momo Kadia, Fongwen Noah Takah, Christian Akem Dimala, Victoria Simms. A systematic review and meta-analysis of the association between integrated tuberculosis and human immuno-deficiency virus therapy and tuberculosis treatment outcomes in sub-Saharan Africa.
**Wrong study design**	
88.	Ruslami, Rovina and Aarnoutse, Rob E and Alisjahbana, Bachti and van der Ven, Andre J A M and van Crevel, Reinout. Implications of the global increase of diabetes for tuberculosis control and patient care. Tropical medicine & international health : TM & IH, 2010; 15(11):1289-99.
89.	Sarkar M. and Srinivasa and Madabhavi I. and Kumar K. Tuberculosis associated chronic obstructive pulmonary disease. Clinical Respiratory Journal, 2017; 11(3):285-295.

For peer review only - http://bmjopen.bmj.com/site/about/guidelines.xhtml

90. Pablo-Villamor M.P. and Benedicto J.P. and Benedicto M.T.J.U. and Perez V.M. Screening for diabetes mellitus in patients diagnosed with pulmonary tuberculosis. Phillippine Journal of Internal Medicine, 2014; 52(4):44440.

91. Sharma P. and Visnegarwala F. and Tripathi V. Burgeoning double burden of tuberculosis and diabetes in India: Magnitude of the problem - Strategies and solutions. Clinical Epidemiology and Global Health, 2014; 2(3):107-116.

92. Wenjie Fang, Min Chen, Jia Liu, Weihua Pan, Wanqing Liao. Tuberculosis/Cryptococcosis co-infection in China from 1965 to 2016: a probably underestimated challenge.

93. Kouemo Motse, Dorgelesse F and Nsagha, Dickson Shey and Adiogo, Dieudonne and Kojom Foko, Loick P and Teyim, Pride M and Chichom-Mefire, Alain and Nguedia Assob, Jules C. Tuberculosis Chemotherapy Outcome in the Littoral Region of Cameroon: A Meta-analysis of Treatment Success Rate between 2014 and 2016. BioMed research international, 2020; 2020(101600173):8298291.

94. Cheng, Jun and Zhang, Hui and Zhao, Yan Lin and Wang, Li Xia and Chen, Ming Ting. Mutual Impact of Diabetes Mellitus and Tuberculosis in China. Biomedical and environmental sciences : BES, 2017; 30(5):384-389.

95. Balinda I.G. and Sugrue D.D. and Ivers L.C. More Than Malnutrition: A Review of the Relationship between Food Insecurity and Tuberculosis. Open Forum Infectious Diseases, 2019; 6(4):.

96. Guto J.A. and Bii C.C. and Denning D.W. Estimated burden of fungal infections in Kenya. Journal of Infection in Developing Countries, 2016; 10(8):777-784.

97. Doherty A.M. and Kelly J. and McDonald C. and O'Dywer A.M. and Keane J. and Cooney J. A review of the interplay between tuberculosis and mental health. General Hospital Psychiatry, 2013; 35(4):398-406.

98. Creswell J. and Raviglione M. and Ottmani S. and Migliori G.B. and Uplekar M. and Blanc L. and Sotgiu G. and Lonnroth K. Series: "Update on tuberculosis" - Tuberculosis and noncommunicable diseases: Neglected links and missed opportunities. European Respiratory Journal, 2011; 37(5):1269-1282.

99. Agnihotram R.V. Reviewing disease burden among rural Indian women. Online Journal of Health and Allied Sciences, 2004; 3(2):.

100. Baziz A. Miliary tuberculosis associated with adult respiratory distress syndrome. Annales de Medecine Interne, 1995; 146(2):114-122.

101. CedeÃ±o-Burbano, Anuar Alonso and CerÃ³n-Ortega, Ronal Fredy and Pacichana-Agudelo, Carlos Eberth and MuÃ±oz-GarcÃa, David AndrÃ©s and Galeano-TriviÃ±o, Gerardo Alfonso and Cardona-GÃ³mez, Diana Catalina and Manquillo-Arias, William AndrÃ©s and Plaza-Rivera, Regina Victoria. Parasitismo intestinal y tuberculosis. Rev. Fac. Med. (BogotÃ¡), 2017; 65(4):673-677.

No data from LMICs

102. Lonnroth, Knut and Williams, Brian G and Cegielski, Peter and Dye, Christopher. A consistent log-linear relationship between tuberculosis incidence and body mass index. International journal of epidemiology, 2010; 39(1):149-55.

Duplicated reference

103. Noubiap, Jean Jacques and Nansseu, Jobert Richie and Nyaga, Ulrich Flore and Nkeck, Jan Rene and Endomba, Francky Teddy and Kaze, Arnaud D and Agbor, Valirie N and Bigna, Jean Joel. Global prevalence of diabetes in active tuberculosis: a systematic review and meta-analysis of data from 2.3 million patients with tuberculosis. The Lancet. Global health, 2019; 7(4):e448-e460.

104. Zheng, Yixiang and Ma, Shujuan and Tan, Deming and Lu, Menghou. [A meta-analysis of liver lesions in hepatitis B patients undergoing anti-tuberculosis therapy]. Zhonghua gan zang bing za zhi = Zhonghua ganzangbing zazhi = Chinese journal of hepatology, 2014; 22(8):585-9.

105. Liu, Jiao and Lu, Bing and Yan, Yan. [Meta analysis on the co-infection between Mycobacterium tuberculosis and HIV/AIDS in China]. Zhonghua liu xing bing xue za zhi = Zhonghua liuxingbingxue zazhi, 2013; 34(1):85-90.

106. Pang P. and Sun C. and Duan W. and Liu S. and Bai S. and Ma Y. and Li R. and Liu F. A review on global head and neck tuberculosis cases from 980 papers and 5881 patients. International Journal of Oral and Maxillofacial Surgery, 2019; 48():185.

107. Liu J. and Lu B. and Yan Y. [Meta analysis on the co-infection between Mycobacterium tuberculosis and HIV/AIDS in China]. Zhonghua liu xing bing xue za zhi = Zhonghua liuxingbingxue zazhi, 2013; 34(1):85-90.

108. Carlos Almeida, Rachel Couban, Sun Kallyth, Li Wang, Jason Busse, Denise Silva. Predictors of in-hospital mortality among patients with pulmonary tuberculosis: systematic review and meta-analysis of observational studies.

109. Bisson G.P. and Bastos M. and Campbell J.R. and Bang D. and Brust J.C. and Isaakadis P. and Lange C. and Menzies D. and Migliori G.B. and Pape J.W. and Palmero D. and Baghei P. and Tabarsi P. and Viiklepp P. and Vilbrun S. and Walsh J. and Marks S.M. Mortality in adults with multidrug-resistant tuberculosis and HIV by antiretroviral therapy and tuberculosis drug use: an individual patient data meta-analysis. The Lancet, 2020; 396(10248):402-411.

110. Hong-Guang CHEN and Min LIU and Fang-Hui GU. Meta-analysis on the co-morbidity rate between tuberculosis and diabetes mellitus in China. Chinese Journal of Epidemiology, 2013; (12):1128-1133.

Protocol of an already included study

111. Tankeu, Aurel T and Bigna, Jean Joel and Nansseu, Jobert Richie and Endomba, Francky Teddy A and Wafeu, Guy Sadeu and Kaze, Arnaud D and Noubiap, Jean Jacques. Global prevalence of diabetes mellitus in patients with tuberculosis: a systematic review and meta-analysis protocol. BMJ open, 2017; 7(6):e015170.

112. Marriott Nliwasa, Elizabeth Corbett, Peter MacPherson, Ankur-Gupta Wright, Katherine Horton. The Prevalence of HIV and Risk of Early Mortality in Adults with Suspected Tuberculosis in Low- and Middle- Income Countries: A Systematic Literature Review.

113. Tamuzi Lukenze Jacques, Birhanu Bayele. Covid-19 implications in high burden HIV/TB countries: a systematic review of evidence.

114. sanju gautam. Prevalence of diabetes mellitus among tuberculosis patients and itâ€™s impact on treatment outcome in South Asian Region: a systematic review and meta-analysis.

115. Cesar Ugarte-Gil, Fiona Pearson. Systematic review and meta-analysis on the diabetes mellitus role on tuberculosis treatment outcomes.

116. Aurel. T. Tankeu, Jean JoÃ«l R. Bigna, Jobert Richie N. Nansseu, Francky Teddy A. Endomba, Guy Sadeu Wafeu, Arnaud D. Kaze, Jean Jacques N. Noubiap. Global prevalence of diabetes mellitus in patients with tuberculosis: a systematic review and meta-analysis protocol.

117. Chi Yan Leung, Hsi Lan Huang, Md. Mizanur Rahman, Sarah Krull Abe, Stuart Gilmour, Kenji Shibuya. Pulmonary and extra-pulmonary tuberculosis infection and the risk of pulmonary and extra-pulmonary malignancies: a systematic review and meta-analysis.

For peer review only - http://bmjopen.bmj.com/site/about/guidelines.xhtml

118.	Animut Alebel, Amsalu Taye Wendemagen, Cheru Tesema, Getiye Dejenu, Fasil Wagnew, Fasil Wagnew, Pammla Petrucka, Setegn Eshetie. Prevalence of diabetes mellitus among tuberculosis patients in Sub-Saharan Africa, and the impact of HIV infection: a systematic review and meta-analysis.
119.	Hasan Abolghasem Gorji, Nicola Luigi Bragazzi. A systematic review and meta-analysis of the prevalence of Hepatitis C virus (HCV) among tuberculosis patients.
120.	Aklilu Endalamaw, Setegn Eshetie, Sintayehu Ambachew, Demeke Geremew, Tesfa Dejenie Habtewold. HIV-infection and unknown HIV status of tuberculosis patients in Ethiopia: a systematic review and meta-analysis.
121.	C. Andrew Basham, Sarah Smith, James C. Johnston. Tuberculosis (TB) and cardiovascular disease (CVD) risk: a systematic review and meta-analysis.
122.	Abraham Assefa, Awoke Derbie, Abebe Shumet, Abaineh Munshae, Endalkachew Nibret, Fantahun Biadglegne. Epidemiology of Tuberculosis Lymphadenitis in Africa: a systematic reviews and meta-analysis.
123.	Habteyes Tola, Kourosh Holakouie-Naieni, Mohammad Mansournia, Mehdi Yaseri, Ephrem Tesfaye, Million Mola, Tadesse Lejisa Lejisa. Is hypothyroidism rare and does it require screening in multidrug-resistant tuberculosis patients on treatment? A systematic review and meta-analysis.
124.	Alvaro Schwalb, CÃ©sar Ugarte, Rodrigo Cachay, Paulo Ruiz, Adriana De la Flor. The association between tuberculosis and depression on poor outcomes of tuberculosis treatment: a systematic review.
125.	Dumessa Edessa, Moti Tolera, Bisrat Hagos. Poor outcomes to second-line tuberculosis treatment among multidrug-resistant patients in sub-Saharan Africa: a systematic review and meta-analysis.
**Full text could not be found**	
126.	Wongtrakul, Wasit and Charoenngam, Nipith and Ungprasert, Patompong. Tuberculosis and risk of coronary heart disease: A systematic review and meta-analysis. The Indian journal of tuberculosis, 2020; 67(2):182-188.
127.	Pang P. and Sun C. and Duan W. and Liu S. and Bai S. and Ma Y. and Li R. and Liu F. A review on global head and neck tuberculosis cases from 980 papers and 5881 patients. International Journal of Oral and Maxillofacial Surgery, 2019; 48():182.
128.	Yixiang ZHENG and Shujuan MA and Deming TAN and Menghou LU. A meta-analysis of liver lesions in hepatitis B patients undergoing anti-tuberculosis therapy. Chinese Journal of Hepatology, 2014; (12):585-589.
**Protocol**	
129.	Benjamin Momo Kadia, Desmond Aroke, Kevin Pene Njefi, Yves-Joel Tochie Noutakdie, Frank-Leonel Tianyi, Reine Suzanne Kadia, Christian Akem Dimala. Systematic review of therapeutic outcomes of multi-drug resistant tuberculosis and their predictors in adults receiving integrated treatment of tuberculosis and Human Immuno-deficiency Virus in low and middle-income countries: a study protocol.
130.	Noemia Siqueira-Filha. Costs of tuberculosis and HIV co-infection for the health system and families worldwide: a systematic literature review.
131.	Minmin Li. National, regional, and global prevalence of diabetes mellitus in patients with tuberculosis: a systematic review and meta-analysis.
132.	Yonggang Zhang, Linli Zheng, Lu Huang. The prevalence of depression in tuberculosis patients: a meta-analysis.
133.	Tamaryn J. Nicholson, Muhammad Osman, Rory Dunbar, Anneke. C. Hesseling, Florian Marx, Elisa Lopez Varela, James A. Seddon, Kogie Naidoo. A systematic review of risk factors for mortality among drug-susceptible and drug-resistant TB patients in South Africa (2010 â€“ 2018).
134.	Rebecca Harris, Fidel Vazquez. A systematic review and meta-analysis of the effect of HIV status on the incidence of tuberculosis disease among individuals with latent Mycobacterium tuberculosis infection.
135.	Nicolas Nunez, Aiswarya Nandakumar, Lakshmi, Aparna Menon, Boney Joseph. Risk of depression in pulmonary tuberculosis.
136.	Melese Teferi, Mekonnen Mekonnen, Hawult Adane. Determinants of tuberculosis (TB) treatment outcome in resource-limited settings: protocol for systematic review and meta-analysis.
137.	Liu Chun, yang Xie, Hulei Zhao, Jiansheng Li. Prevalence of pneumoconiosis and that complicated with pulmonary tuberculosis in China: a systemic review and meta-analysis.
138.	Melaku Kindie Yenit, Wubet Worku Takele, Achenef Asmamaw Muche, Setegn Eshetie Kebede, Dessie Abebaw Angaw. The effect of depression on Tuberculosis (TB) treatment outcome in Africa: A Protocol for systematic review and meta-analysis.
139.	Lucy Kaluvu, Asogwa Ogechukwu, Anna Marza Florensa, Daniel Boateng, Kerstin Klipstein-Grobusch. A systematic review to examine the patterns, prevalence and management of multimorbidity of communicable and non-communicable diseases in low- and middle- income countries.
140.	Marie Charmaine Sy, Adrian Espiritu, Jose Leonard Pascual. Global frequency of stroke in tuberculous meningitis: A systematic review and meta-analysis.
141.	Mogesie Necho, Mekonnen Tsehay, Asmare Belete. Prevalence and Associated Factors of Alcohol Use Disorder Among Tuberculosis Patients: A Systematic Review and Meta-analysis.
142.	Mogesie Necho, Mengesha Birkie. Psychological distress and Depression in Tuberculosis patients in Africa: A systematic Review and Meta-analysis.
143.	Javier Ogembo, Rebecca Ogembo, Paul Bain. Prevalence of drug-resistant tuberculosis in sub-Saharan Africa: systematic review and meta-analysis.
144.	Adhanom Baraki, Abel Dadi, Hanna Desyibelew. Epidemiology of depression among tuberculosis patients: a systematic review and meta-analysis of observational studies from Africa.
145.	Jing Wu, Jennifer M McGoogan, Zunyou Wu. Prevalence of HIV/TB co-infection worldwide: a systematic review and meta-analysis.
146.	Birhanie Mekuriaw, Alemayehu Molla, Zelalem Belayneh, Tsegaye Mehare. Depression and its determinants among individuals with Tuberculosis in Ethiopia: a systematic review and meta-analysis.
147.	Chalachew Adugna, Aklilu Enalamaw. Poor treatment adherence and associated factors among patients taking anti-tuberculosis drug in Ethiopia: systematic review and meta-analysis.
148.	Gebremedhin Berhe Gebregergs, Gebreamlak Gidey, Fitwi Tinsae, Gebremedhin Gebreziher, Selam Desalegn. Gender difference in co-morbid depression among tuberculosis patients in high burden countries: systematic review and meta-analysis.
149.	Belayneh Kefale, Mulugeta Molla, Amien Ewunetie, Amsalu Degu, Gobezie Temesgen. Treatment outcomes and associated factors among tuberculosis patients in Ethiopia: a systematic review and meta analysis.

For peer review only

150.	Alvaro Schwalb, Jorge InolopÃº, Eduardo Gotuzzo, Rodrigo Cachay, Fernando MejÃa, Larissa Otero, Carlos Seas, CÃ©sar Ugarte-Gil, Kristien Verdonck, Nicole Young. Clinical and epidemiological association between HTLV-1/2 and tuberculosis: systematic review.
151.	Demeke Geremew. Latent TB and its associated factors in Ethiopian: an aggregated and individual patientsâ€™ data meta-analysis.
152.	Carole Mitnick, Molly Franke, Celia Fung, Andrew Lindeborg. Clinical Outcomes of Individuals with COVID-19 and Tuberculosis Disease: a Living Systematic Review.
153.	Javier Cabrera, Vicente Cuba. Tuberculosis and lung cancer risk: a systematic review and meta-analysis.
154.	Jemal Abdela, Fuad Adem, Abraham Nigussie. Incidences and risks of drug-induced hepatotoxicity among Tuberculosis and HIV/ Tuberculosis co-infected patients in Sub Saharan Africa: a systematic review and meta-analysis.
**Conference abstract**	
155.	Schafer J.M. and Welwarth J. and Balk D.S. and Lee C. and Hardin J. and Hoffmann B. Rates of pericardial effusion in patients with HIV/tuberclosis co-infection: A systematic review and meta-analysis. Academic Emergency Medicine, 2017; 24():S97.
156.	Moniruzzaman A. and Kazanjian A. and Wong H. and Chowdhury M.M. and Elwood R.K. and Fitzgerald J.M. A systematic review on risk factors of mortality among Tb patients. American Journal of Respiratory and Critical Care Medicine, 2010; 181(1):.
157.	Yeats, J. and Patel, S. and Shete, P. B. and Cattamanchi, A. and Baker, B. J. Reactivation Risk Of Latent Tuberculosis Infection By Population Subgroup: A Systematic Review And Meta-Analysis. AMERICAN JOURNAL OF RESPIRATORY AND CRITICAL CARE MEDICINE, 2016; 193():.
158.	Huang Y.-S. Chronic hepatitis C may increase the risk of anti-tuberculosis drug-induced liver injury: A systematic review and meta-analysis. Hepatology International, 2017; 11(1):S842.
159.	Chen H. and Liu M. and Gu F. Meta-analysis on the co-morbidity rate between tuberculosis and diabetes mellitus in China. Zhonghua liu xing bing xue za zhi = Zhonghua liuxingbingxue zazhi, 2013; 34(11):1128-1133.
160.	Arruda S. and Loureiro C. and Almeida M. and Mendes D. and Grassi M.F.R. and Lapa J.R. and Kritski A. and Verdonck K. and Gotuzzo E. and Galvao-Castro B. Association between human T-Cell lymphotropic virus type 1 and 2 (HTLV 1/2) infection and tuberculosis: Systematic review and meta-analysis. Retrovirology, 2011; 8():A80.
161.	Hussain, M. S. and Siddiqui, A. N. and Najmi, A. K. Burden of diabetes mellitus among tuberculosis patients in Asia-Pacific region: Evidence from meta-analysis using real-world data. INTERNATIONAL JOURNAL OF INFECTIOUS DISEASES, 2018; 73():87.
162.	Jiao LIU and Bing LV and Yan YAN. Meta analysis on the co-infection between Mycobacterium tuberculosis and HIV/AIDS in China. Chinese Journal of Epidemiology, 2013; (12):85-90.
**Wrong publication type (letters to the editor, corrections, protocol for this meta-review)**	
163.	Pai, Madhukar and McCulloch, Michael and Colford, John M Jr. Meta-analysis of the impact of HIV on the infectiousness of tuberculosis: methodological concerns. Clinical infectious diseases : an official publication of the Infectious Diseases Society of America, 2002; 34(9):1285-7.
164.	Chen M. and Al-Hatmi A.M. and Chen Y. and Ying Y. and Fang W. and Xu J. and Hagen F. and Hong N. and Boekhout T. and Liao W. and Pan W. Cryptococcosis and tuberculosis co-infection in mainland China. Emerging Microbes and Infections, 2016; 5(9):e98.
165.	Anonymous. Correction: Association between HIV/AIDS and multi-drug resistance tuberculosis: A systematic review and meta-analysis (PLoS ONE). PLoS ONE, 2014; 9(2):e89709.
166.	Chen L. and Li N. and Liu M. and Zhang J. and Zhang H. High prevalence of multidrug-resistant tuberculosis in Zunyi, Guizhou province of China. Journal of Antimicrobial Chemotherapy, 2011; 66(10):2435-2437.
167.	Alexander Jarde, Ruimin Ma, Eugenia Romano, Helen Elsey, Kamran Siddiqi, Najma Siddiqi, Brendon Stubbs. A meta-review of systematic reviews of chronic disease multimorbidity in people with tuberculosis in low- and middle-income countries.

For peer review only

Supplementary Table 3: Study characteristics of systematic reviews reporting pooled outcomes of studies from LMICs

Lead author and year	Search dates and limits	Number of studies in LMIC/Total number of studies (countries covered)	Clusters covered	Outcomes	Quality rating
**TB + HIV**					
Alemu 2020[S54]	2009 to Jan 2020; English language, in Ethiopia	17/17 (Ethiopia)	TB +HIV	Prevalence*	Low
Arega 2020 [S24]	200 to 2019; English language, in Ethiopia	47 / 47 (Ethiopia)	TB +HIV	Prevalence*	Critically low
Bastos 2019 [S25]	period between 2008 and 2017; Brazil only	15 /15 (Brazil)	TB +HIV	Mortality*, Treatment outcomes*	Critically low
Bisson 2020 [S48]	2009 to Sep 2015	52 (South Africa, Philippines, Georgia, Russia, Latvia, Peru, India, Haiti, Pakistan, Belarus, Brazil, Thailand, Mexico, Bulgaria, Argentina, Ecuador)	MDR-TB +HIV	Prevalence (pooled individuals - no meta-analysis)	Low
Chem 2019 [S5]	2004 to May 2018; English language, in SSA countries	9 / 9 (South Africa, Lesotho, Botswana, Ethiopia)	MDR-TB +HIV	OR of treatment success and prevalence of death and cured. Prevalence*	Critically low
Edessa 2020 [S6]	up to Feb 2020; English language, in SSA countries	19/19 (South Africa, Lesotho, Ethiopia, Kenya, Tanzania, Niger, Botswana)	DR-TB +HIV	RR of unfavourable outcome (Failed treatment/Lost from treatment/died), death, treatment failure and loss to follow-up	High
Endalamaw 2019 [S7]	2003-2018; English language, in Ethiopia	13/13 (Ethiopia)	TB +HIV ; PTB +HIV	Prevalence	Moderate
Eshetie 2018 [S2]	up to 2017; English language, in Ethiopia	34 /34 (Ethiopia)	TB +HIV	Prevalence of successful and unsuccessful treatment	Critically low
Gao 2010 [S8]	up to Apr 2010; English or Chinese language, in mainland China	29/29 (China)	TB +HIV	Prevalence (also by sex)	Critically low
Gao 2013 [S9]	up to Dec 2011; English language, any country except China	31/47 (Brazil, Nigeria, Ethiopia, India, Iran, South Africa, Zambia, Zimbabwe, Cambodia, Tanzania, Thailand, Togo, Ukraine, Vietnam)	TB +HIV	Prevalence	Critically low
Gelaw 2019 [S10]	up to Sep 2017; English language in SSA countries	68/68 (Ethiopia, Tanzania, Kenya, Eritrea, Uganda, South Africa, Zambia, Zimbabwe, Angola, Malawi, Nigeria, Cote d'Ivoire, Ghana, Burkina Faso, Togo, Cameroon, Republic of Congo)	TB +HIV	Prevalence (in SSA and in the central, southern, western and eastern regions of SSA)	High
Huddart 2020 [S1]	2006 to Jan 2019; in India	212 / 212 (India)	TB +HIV	Case-fatality rate (during treatment and after treatment)	Critically low
Lukoye 2015 [S26]	2003 to 2013; SSA countries only	27/27 (SSA)	TB +HIV	Prevalence*	Critically low
McMurry 2019 [S27]	1990 to 2016.; English language, LMIC only	84/84 (India, China, Mexico, Tanzania, Ethiopia, Malaysia, Pakistan, Brazil, Nigeria, South Africa, Bangladesh, Indonesia, Marshall Islands, Turkey, Benin, Fiji, Georgia, Guinea, Guinea-Bissau, Guyana, Iran, Kazakhstan, Kiribati, Kyrgystan, Micronesia, Peru, Philippines, Sri Lanka, Thailand, Zambia)	TB +HIV ; TB +DM	Prevalence*	Low
Mekonnen 2019 [S11]	up to Mar 2018; English language, in African countries	28 / 28 (Ethiopia, Zambia, South Africa, Nigeria, Burkina Faso, Uganda, Djibouti, Mozambique, Sudan, Tunisia, Tanzania, Malawi)	TB lymphadenitis +HIV	Prevalence	High
Mesfin 2014 [S49]	up to April 2012.; English language	4/24 (Ucraine, Haiti, Georgia, South Africa)	MDR-TB +HIV	Prevalence*, OR*	Critically low
Pormohammad 2018 [S52]	1985 to Mar 2018	16 / 20 (Dominican Republic, South Africa, Indonesia, China, Vietnam, Turkey, India, Brazil, Peru, Zambia)	TB Meningitis +HIV	Prevalence*	Critically low

For peer review only - http://bmjopen.bmj.com/site/about/guidelines.xhtml

Pourakbari 2019 [S28]	up to Apr 2017; Persian and English languages, Iran only	48/48 (Iran)	TB +HIV	Prevalence*	Critically low
Purmohamad 2020 [S53]	2000 to Jan 2017; English language	22/26 (South Africa, Turkey, China, India, Egypt, Brazil, Peru, Vietnam, Indonesia)	TB Meningitis +HIV	Prevalence*	Critically low
Rajendran 2020 [S29]	2009 to Dec 2018; English language, Malaysia only	23/23 (Malaysia)	TB +HIV; TB +DM	Prevalence*	Critically low
Reddy 2010 [S30]	up to Jun 2009; African countries only	22 / 22 but only 5 provided data on TB (Tanzania, Malawi, Uganda, and Cote d'Ivoire)	TB +HIV	Prevalence (pooled individuals - no meta-analysis)	Critically low
Samuels 2018 [S12]	1980 to Jun 2016; English, French and Spanish language	39 / 48 (Ethiopia, Georgia, Russia, Latvia, South Africa, India, Cameroon, Nigeria, Pakistan, Vietnam, Uzbekistan, Moldovia, Belarus, Peru, China, Turkey, Haiti, Philippines,)	MDR/XDR-TB +HIV; MDR/XDR-TB +DM	RR of unsuccessful treatment (composite of failure, death, and default) and treatment failure. Prevalence*	Low
Seid 2018 [S31]	up to Mar 2017; English language, Ethiopia only	34 / 34 (Ethiopia)	TB +HIV	Prevalence*	Critically low
Sotgiu 2009 [S47]	2006 to Dec 2008.; English language	5 / 13 (South Africa, Russia, Peru)	MDR-TB +HIV; XDR-TB +HIV	Mortality*, Treatment outcomes*	Critically low
Straetemans 2011 [S32]	up to Mar 2011; English language	50 / 70 (studies with TB + HIV: 14 / 22) (South Africa, Ivory Coast, Uganda, Somalia, Iran, Malawi, Thailand, Zambia, Mexico, Russia, Sudan, Cambodia, Central African Republic, Guinea-Bissau, Nepal, Ivory Coast, India, Burkina Faso, Cameroon, Kenya, Zaire, China, Vietnam)	TB +HIV	Mortality*	Critically low
Tesfaye 2018 [S33]	2007 to 2016; English language, Ethiopia only	21/21 (15 for prevalence data) (Ethiopia)	TB +HIV	Prevalence*	Critically low
Teweldemedhin 2018 [S34]	1995 to Nov 2017; English language, Ethiopia only	30/30, but only 19/30 determined HIV infection among TB patients (Ethiopia)	TB +HIV	Prevalence*	Critically low
Uchida 2019 [S35]	1919 to 2017; English language	2/7 (India, Nigeria)	TB +HIV	Treatment outcomes*	Critically low
Waitt 2011 [S36]	1966 to 2010; English language	40 / 62 (China, Sudan, Brazil, Vietnam, South Africa, Gambia, Malawi, India, Russia, Thailand, Tanzania, Guinea Bissau, Peru, Mexico, Zambia, Uganda, Bolivia)	TB +HIV; TB +Non-infective comorbidities	Mortality*	Critically low
Wang 2019 [S13]	up to May 2018; English language	21 / 22 (Turkey, India, China, Vietnam, South Africa, Indonesia, Malaysia, Madagascar)	TB meningitis +HIV	Prevalence and prevalence of death	Critically low
Wu 2016 [S50]	up to Oct 2012; English or Chinese language	23/39 (Peru, India, Bangladesh, Lesotho, Iran, China, Latvia, Russia, Uzbekistan, Turkey, South Africa)	MDR-TB +HIV	Prevalence*	Critically low
**TB + DM**					
Alebel 2019 [S14]	up to Sep 2017; English language, in SSA countries	16/16 (Benin, Tanzania, Guinea-Bissau, Uganda, Nigeria, Ethiopia, Guinea, Madagascar, Kenya, Cameroon)	TB +DM	Prevalence	Low
Almeida 2018 [S51]	up to Nov 2015	2/11 (Iran, China)	PTB +DM	Mortality*	Critically low
Baker 2011 [S37]	1980 to Dec 2010	12 / 33 (Indonesia, Thailand, India, Turkey, Iran, Russia, Tunisia, Republic of the Congo, Mexico, China)	TB +DM	Prevalence*, Mortality*, Treatment outcomes*	Critically low
Chen 2013 [S15]	2000-Apr 2013; in China	22/22 (China)	PTB +DM	Prevalence	Critically low
Gautam 2021 [S4]	1980 to Jul 2020; English language, in Afghanistan, Bangladesh, Bhutan, India, Maldives, Nepal, Pakistan, Sri Lanka	65/ 74 (India, Pakistan, Nepal, Bangladesh, and Sri-Lanka)	TB +DM	Prevalence (also by countries), OR of mortality, treatment failure, culture conversion, recurrence, and MDR-TB	Low

For peer review only - http://bmjopen.bmj.com/site/about/guidelines.xhtml

Han 2016 [S38]	1980 to Jul 2015; English language	27/54 (total articles included), 27/33 (articles used for the meta-analysis) (Congo, Tunisia, Russia, Turkey, Thailand, China, India, Mexico, Iran, Kiribati, Brazil, Malaysia, Tanzania, Peru, Indonesia)	TB +DM	Mortality*, Treatment outcomes*	Critically low
Huang 2020 [S39]	1966 to Jul 2019; English only	10/13 (India, China, Mexico, Thailand, Georgia, Iran)	TB +DM	Prevalence*	Critically low
Huangfu 2019 [S16]	1980 to Jul 2018	57/104 (Congo, Indonesia, Iran, Thailand, Tunisia, Turkey, Brazil, India, Mexico, China, Malaysia, Fiji, Kiribati, Ethiopia, Argentina, Poland, Tanzania, Russia, Egypt, Uganda, Georgia, Saudi Arabia, Senegal)	TB +DM	OR of death only and treatment failure and death	Low
Jeon 2010 [S40]	up to May 2009 (databases), 2007 to 2008 (World Lung Conferences abstracts)	16 / 18 studies that met our inclusion criteria on screening for DM among patients with TB (32 studies included in total) (India, Russia, Nigeria, Guinea, Pakistan, Turkey, Indonesia, Tanzania, Mexico, Iran)	TB +DM	Prevalence*, RR*	Critically low
Lutfiana 2019 [S41]	2012 to Sep 2017; English language	32/41 (South Africa, China, India, Thailand, Bangladesh, Georgia, Brazil, Tanzania, Vietnam, Mongolia)	TB +DM	Prevalence*	Critically low
Noubiap 2019 [S17]	1986 to Jun 2017	138/ 200 (Benin, Ethiopia, Guinea-Bissau, Nigeria, Senegal, South Africa, Tanzania, Uganda, Georgia, Kazakhstan, Romania, Turkey, Egypt, Iran, Libya, Pakistan, Tunisia, Yemen, Guyana, Mexico, Brazil, Peru, Bangladesh, India, Sri Lanka, China, Fiji, Indonesia, Kiribati, Malaysia, Marshall Islands, Thailand)	TB +DM	Prevalence (by income level, regions and countries)	Critically low
Shao-hua 2016 [S18]	up to Nov 2015; in China	13 / 13 (China)	PTB +DM	OR and aOR of adverse outcomes (failure to retreatment, death, and loss)	Critically low
Tegegne 2018 [S42]	up to Jul 2018; English language	17 / 25 (Iran, Georgia, Mexico, Egypt, Thailand, Peru, China, Indonesia, Bangladesh, Turkey)	TB +DM	Prevalence*	High
Workneh 2017 [S43]	up to Mar 2016; English language	80/94 (India, China, Iran, Indonesia, Pakistan, Sri Lanka, Nepal, Thailand, Georgia, Philippines, Malaysia, Benin, Ethiopia, Tanzania, Guinea, Kenya, Ethiopia, Uganda, Nigeria, South Africa, Madagascar, Mexico, Peru, Brazil, Kiribati, Marshall Islands, Guyana, Fiji)	TB +DM	Prevalence*	Low
**TB + Mental disorders**					
Alene 2018 [S19]	up to September 2017	37/40 (1 studies on Canada and 2 on South Korea) (China, Peru, Russia, Argentina, Turkey, Iran, Lesotho, Latvia, South Africa, Tanzania, Haiti, Pakistan, Ethiopia, Vietnam, Indonesia, Nigeria, Namibia)	MDR-TB +Depression; MDR-TB +Anxiety; MDR-TB +Psychosis	Prevalence (also by regions)	Critically low
Duko 2020 [S20]	up to Dec 2019; English language	25/25 (Pakistan, Turkey, India, Brazil, China, Nigeria, Cameroon, Ethiopia)	TB +Depression; MDR-TB +Depression	Prevalence (also by sex)	High
Lee 2020 [S21]	1990 to Oct 2018; English, French, Spanish, Portuguese, and Korean languages	10/10 (South Africa, Ethiopia, Zimbabwe, Zambia, Tanzania, Peru, China)	TB +Mental Disorders	OR of poor TB treatment outcomes, loss to follow-up and non-adherence to treatment. Prevalence*, Mortality*	Critically low
Rensburg 2020 [S44]	2000 to 2019; English language	100/100 (Pakistan, South Africa, Peru, Pakistan, China, Ethiopia, Thailand, India, Sudan, Cameroon, Kazakhstan, Sri Lanka, Nigeria, Zambia, Russia, Brazil, Poland, Burkina Faso, Estonia, Angola, Romania, Ukraine)	TB +Mental illness (Depression, Anxiety, Alcohol use, and General Mental health)	Prevalence*	Critically low

For peer review only - http://bmjopen.bmj.com/site/about/guidelines.xhtml

Ruiz-Grosso 2020 [S3]	up to Aug 2019; English language	8/8 (South Africa, Peru, Ethiopia, China, Zimbabwe, Zambia, Tanzania)	TB +Depression	OR of negative outcomes (death and loss to follow-up), death, loss to follow-up and non-adherence	Critically low
**TB + HCV**					
Behzadifar 2019 [S22]	2000 to Mar 2018; English language	13/21 (Georgia, Argentina, Iran, Brazil, Egypt, Pakistan, China, Sudan, Iraq)	TB +HCV	Prevalence	Moderate
**TB + other**					
Basham 2020 [S45]	up to Jan 2020 (databases), 2013 to Dec 2019 (The International Journal of Tuberculosis and Lung Disease); English language	5/16 (Tanzania, Egypt, Peru, Russia, Estonia)	TB + cardiovascular disease	OR*, Mortality*	Critically low
Leung 2020 [S23]	up to Jun 2019; any language	14/47 (Lithuania, China, Czech Republic)	TB +Lung cancer; TB +non-Hodgkin's lymphoma; TB +Leukaemia	RR of lung cancer, non-Hodgkin's lymphoma, and leukaemia	Low
Rehm 2009 [S46]	up to Sep 2008; English language	14/53 (Russia, India , Brazil, Belarus, Kazakhstan, Romania, Slovenia)	TB+AUD (Alcohol Use Disorder)	Prevalence*	Critically low

Notes: aOR: adjsuted OR; DM: Diabetes Mellitus; DR-TB: Drug resistant TB; HCV: Hepatitis C Virus; HIV: Human immunodeficiency virus; LMIC: Low- and middle-income countries; MDR-TB: Multidrug resistant TB; OR: Odds Ratio; PTB: Pulmonary TB; RR: Relative Risk; SSA: Sub-Saharan Africa; Tuberculosis; XDR-TB: Extensively drug-resistant TB.

* Outcomes available for individual studies, but not pooled

For peer review only - http://bmjopen.bmj.com/site/about/guidelines.xhtml

Supplementary Table 4: Outcomes reported by each systematic review

Lead author and year	Clusters covered	Outcomes	Quality rating
**TB+HIV**			
Alemu 2020	TB +HIV	Outcomes available for individual studies, but not pooled: Prevalence*: range 7.1% - 30.4%	Low
Arega 2020	TB +HIV	Outcomes available for individual studies, but not pooled: Prevalence*: range 1.70%-45.80%	Critically low
Bastos 2019	TB +HIV	Outcomes available for individual studies, but not pooled: Mortality*: range 3.6% - 30.9%, Treatment outcomes*: cure (33% - 62%), abandonment of treatment (4.2% - 13.6%)	Critically low
Bisson 2020	MDR-TB +HIV	Low and lower-middle income countries: 5.1% (130 of (2421+130) pooled individuals from two studies) - no MA<br>Upper-middle: 3585/(3585+3244)= 52.5% (pooled individuals from two studies) - no MA	Low
Chem 2019	MDR-TB +HIV	Successful treatment: OR 0.87 (0.79 - 0.96), 6 studies, number of participants NR, I^2 NR, range 0.75 - 1.26)<br>Mortality: 18% (14%-23%, 9 studies, number of participants NR, I^2=91.1%, range=9%-31%)<br>Cured: 34% (22%-45%, 9 studies, number of participants NR, I^2=98.9%, range= 3%-60%)<br>Outcomes available for individual studies, but not pooled: Prevalence*: 21.73% - 100%	Critically low
Edessa 2020	DR-TB +HIV	Unsuccessful treatment: † RR 1.18 (1.07-1.30, 19 studies, 8301 participants, I^2=48%, range=0.71-2.37)<br>Unsuccessful treatment (western SSA region) : RR 1.42 (0.95-2.13, 2 studies, 790 participants, I^2=12%, range=1.31-2.37)<br>Unsuccessful treatment (eastern SSA region): RR 1.47 (95% CI: 1.23–1.75, 6 studies, 1970 participants, I^2=0%, range=1.14-1.77)<br>Unsuccessful treatment (southern SSA region): † RR 1.09 (0.98-1.20, 11 studies, 5541 participants, I^2=43%, range=0.71-1.41)<br><br>Mortality: † RR 1.50 (1.30-1.74, 16 studies, 7365 participants, I^2=39%, range=0.73-2.18)<br>Mortality (western SSA region) : RR 1.42 (0.96-2.09, 1 study, 588 participants)<br>Mortality (eastern SSA region): RR 1.52 (95% CI: 1.19–1.93, 5 studies, 1442 participants, ,I^2=0%, range=1.20-2.18)<br>Mortality (southern SSA region): † RR 1.49 (1.21-1.83, 10 studies, 5335 participants, I^2=60%, range=0.73-1.47)<br><br>Treatment failure: † RR 0.66 (0.38-1.13, 10 studies, 5474 participants, I^2=73%, range=0.15-2.40)<br>Loss to follow up: † RR 0.82 (0.74-0.92, 14 studies, 7051 participants, I^2=0%, range=0.49-2.61)	High
Endalamaw 2019	TB +HIV; PTB +HIV	Prevalence: 23.40% (95% CI 19.56%-27.24%, 13 studies, 19212 participants, I^2=97.6%, range=9.50%-52.10%)<br>PTB. 22.08% (95% CI 14.36%-29.81%, 3 studies, 1079 participants, I^2=89.9%, range 14.97%-28.60%)	Moderate
Eshetie 2018	TB +HIV	Successful treatment: prev 67% (56%-79%, number of studies NR, number of participants NR, I^2 NR, range NR)<br>Unsuccessful treatment: prev 33% (21%-44%, number of studies NR, number of participants NR, I^2 NR, range NR)<br>Unsuccessful treatment: OR (TB+HIV vs TB) 1.98 (1.56-2.52, 20 studies, number of participants NR, I^2 =81.0%, range 0.82-14.31)	Critically low
Gao 2010	TB +HIV	Prevalence: 0.9% (95% CI 0.6%–1.4%, 18 studies, number of participants NR, I^2= 92.21, range 0.1%–4.5%)<br>Men: 1.1% (95% CI 0.6% - 2.0%, 9 studies, number of participants NR, I^2=94.7%)<br>Women: 0.6% (95% CI 0.3% - 1.1%, 9 studies, number of participants NR, I^2=71.8%)	Critically low
Gao 2013	TB +HIV	Africa: 31.2% (95% CI 19.3% - 43.2%), 17 studies, number of participants NR, I^2=99.6%, range NR)<br>Latin America: 25% (95% CI 19.3% - 30.8%), 7 studies, number of participants NR, I^2=95.2%, range NR)	Critically low
Gelaw 2019	TB +HIV	SSA: prevalence 31.81% (95% CI 27.83%-36.07%; 68 studies, 62696 participants, I^2=98%, range=6.03%-72.25%)<br>Eastern region (SSA): prev. 31.14% (95% CI 25.39%-37.54%, 32 studies, 33637 participants, I^2=98%, range=6.03%-60.51%)<br>Western region (SSA): prev. 25.48% (95% CI 19.70%-32.27%), 21 studies, 16145 participants, I^2=98%, range=10.26%-72.13%)<br>Southern region (SSA): prev. 43.67% (95% CI 35.05%-52.69%, 12 studies, 11148 subject, I^2=99%, range=23.84%-72.25%)<br>Central region (SSA): prev. 41.33% (95% CI 30.39%-53.19%, 3 studies, 2039 participants, I^2=96%, range=31.29%-51.56%)	High
Huddart 2020	TB +HIV	Case-fatality rate (during treatment): 10.91% (7.68%-15.50%), 35 studies, number of participants NR, Tau^2=0.90 (considered low heterogeneity if <4, according to the authors)<br>Case-fatality rate (after treatment): 4.15% (1.06% to 16.24%), 5 studies, number of participants NR, Tau2=1.902 (considered low heterogeneity if <4, according to the authors)	Critically low

For peer review only - http://bmjopen.bmj.com/site/about/guidelines.xhtml

Lukoye 2015	TB +HIV	Outcomes available for individual studies, but not pooled: Prevalence*: range 25.4% - 79.9%	Critically low
McMurry 2018	TB +HIV; TB +DM	Outcomes available for individual studies, but not pooled: Prevalence*: range 1.9% - 45%	Low
Mekonnen 2019	TB lymphadenitis +HIV	Africa: 52% (95% CI 33%-71%, 14 studies, number of participants NR, I^2=99.2%, range 6%-91%)<br>Ethiopia: 21% (95% CI 12%-30%, 6 studies, number of participants NR, I^2=92.9%, range 6%-67%)	High
Mesfin 2014	MDR-TB +HIV	Outcomes available for individual studies, but not pooled: Prevalence*: range 3.4% - 31.6%, OR*	Critically low
Pormohammad 2018	TB Meningitis +HIV	Outcomes available for individual studies, but not pooled: Prevalence*: range 0% - 100%	Critically low
Pourakbari 2019	TB +HIV	Outcomes available for individual studies, but not pooled: Prevalence*: range 0.4% - 38%	Critically low
Purmohamad 2020	TB meningitis +HIV	Outcomes available for individual studies, but not pooled: Prevalence*: range 0% - 91%	Critically low
Rajendran 2020	TB +HIV; TB +DM	Outcomes available for individual studies, but not pooled: Prevalence*: MDR-TB+DM: 26.7% (1 study); MDR-TB+HIV: 17.6% (1 study)	Critically low
Reddy 2010	TB +HIV	161 / 166 (97%) – no meta-analysis	Critically low
Samuels 2018	MDR/XDR-TB +HIV ; MDR/XDR-TB +DM	Unsuccessful treatment:<br>MDR/XDR-TB + HIV (low gross domestic product countries): RR 2.23 (1.60-3.11, 7 studies, 2662 participants, I^2=41%, range=0.67-3.33)<br>MDR/XDR-TB + HIV (LMIC): RR 1.34 (1.04-1.72, 13 studies, 5816 participants, I^2=88%, range=0.55-3.33)<br>MDR/XDR-TB + DM (vs MDR/XDR-TB only): RR 0.90 (0.65-1.23, 3 studies, 687 participants, I^2=19%, range=0.23-0.98)<br><br>Treatment Failure (defined as 5 cultures positive within the last 12 months of therapy or any culture positivity within the last 3 cultures; alternatively, failure was defined as treatment discontinuation due to lack of appropriate response or significant adverse events):<br>MDR/XDR-TB + HIV (vs MDR/XDR-TB only): RR 0.75 (0.44-129, 7 studies, 5930 participants, I^2=55%, range=0.32-2.40)<br>Outcomes available for individual studies, but not pooled: Prevalence*:	Low
Seid 2018	TB +HIV	Outcomes available for individual studies, but not pooled: Prevalence*: range 2.0% - 100%	Critically low
Sotgiu 2009	MDR-TB +HIV; XDR-TB +HIV	Outcomes available for individual studies, but not pooled: Mortality*: , Treatment outcomes*:	Critically low
Straetemans 2011	TB +HIV	Outcomes available for individual studies, but not pooled: Mortality*: range 2.2% - 34.4%	Critically low
Tesfaye 2018	TB +HIV	Outcomes available for individual studies, but not pooled: Prevalence*: range 11.4% - 36.2%	Critically low
Teweldemedhin 2018	TB +HIV	Outcomes available for individual studies, but not pooled: Prevalence*: range 6%-52.1%	Critically low
Uchida 2019	TB +HIV	Outcomes available for individual studies, but not pooled: Treatment outcomes*: Unsuccessful outcomes (death, failure, loss to follow-up and transferred-out HIV positive): aOR 3.6 (95%CI 1.1-11.7) (1 study)	Critically low
Waitt 2011	TB +HIV; TB + Non-infective comorbidities	Outcomes available for individual studies, but not pooled: Mortality*:	Critically low
Wang 2019	TB meningitis +HIV	Prevalence: 10.6% (95% CI: 4.2%–24.6%, number of studies NR, number of participants NR, I^2 NR, range NR)<br>Mortality: 53.4% (42.4%–64.1%, 7 studies, 547 participants, I^2= 2.1%, range NR)	Critically low
Wu 2016	MDR-TB +HIV	Outcomes available for individual studies, but not pooled: Prevalence*:	Critically low
**TB+DM**			

For peer review only - http://bmjopen.bmj.com/site/about/guidelines.xhtml

Alebel 2019	TB +DM	Prevalence TB+DM: 9% (95% CI 6%-12%, 16 studies, 13286 participants, I^2= 97.48%, range: 2%-38%)<br>Nigeria: 15% (95% CI 7%-23%, 4 studies, 4998 participants, I^2 NR, range NR)<br>Ethiopia: 10% (95% CI 6%-13%, 3 studies, 1633 participants, I^2 NR, range NR)<br>Tanzania: 11% (95% CI 9%-12%, 2 studies, 1309 participants, I^2 NR, range NR)	Low
Almeida 2018	PTB +DM	Outcomes available for individual studies, but not pooled: Mortality*: OR 9.70 (95% CI 2.92-32.22)	Critically low
Baker 2011	TB +DM	Outcomes available for individual studies, but not pooled: Prevalence*: , Mortality*: range of RR 1.26 - 28.47, Treatment outcomes*: relapse: range of RR 1.88 - 5.96; remaining sputum culture positive: range of RR 0.79 - 2.17, failure/death: range of RR 1.44 - 3.13	Critically low
Chen 2013	PTB +DM	Prev: 7.20% (95% CI 6.01%-8.39%, 22 studies, 56805 participants, I^2 NR, range 2.08%-16.16%)	Critically low
Gautam 2021	TB +DM	Pooled prevalence 21% (95% CI 18%-23%, 65 studies, 49,792 patients, I^2=98.28%, range NR)<br>MDR-TB OR 1.05 (95% CI 0.63-1.74, 4 studies, number of participants NR, I^2=40,71%, range NR)<br>Among adults only: 21.0% (95% CI 18.0–23.0%, 55 studies, number of participants NR, I^2= 97.99%, range NR)<br><br>Bangladesh prevalence 11.0% (95% CI 10.0%–12.0%, 2 studies, number of participants NR, I^2 NR, range NR)<br>India prevalence 22.0% (95% CI 19.0%–25.0%, 47 studies, number of participants NR, I^2=97.92%, range NR)<br>Nepal prevalence 12.0% (95% CI 4.0%–20.0%, 4 studies, number of participants NR, I^2=96.70%, range NR)<br>Pakistan prevalence 19.0% (95% CI 11.0%–27.0%, 10 studies, number of participants NR, I^2=99.18%, range NR)<br>Sri Lanka prevalence 24.0% (95% CI 21.0%–27.0%, 2 studies, number of participants NR, I^2 NR, range NR)<br>Mortality in TB+DM vs TB: OR 1.74 (1.21-2.51, 5 studies, number of participants NR, I^2=19.43%, range 0.14-1.95)<br>Treatment failure: OR 1.65 (1.12-2.44, 5 studies, number of participants NR, I^2=49.63%, range 1.34-21.91)<br>Cured: OR 0.32, 95% CI 0.10 - 1.05, 1 study)<br>Recurrence: OR 0.53 (95% CI 0.32, 0.87, 1 study)<br>MDR-TB: OR 1.05 (0.63-1.74, 4 studies, number of participants NR, I^2=40.71%, range=0.45-4.70)	Low
Han 2016	TB +DM	Outcomes available for individual studies, but not pooled: Mortality*: range of OR 0.41 - 29.22, Treatment outcomes*: Sputum culture conversion at 2 to 3 months: range of OR 0.57 - 5.27; Failure/death: range of OR 0.86 - 18.91; Relapse: range of OR 0.97 - 6.35	Critically low
Huang 2020	TB +DM	Outcomes available for individual studies, but not pooled: Prevalence*: range 9% - 49%	Critically low
Huangfu 2019	TB +DM	Mortality: OR 1.80 (95%CI 1.35–2.40; 32 studies, number of participants NR, I^2=91%, range NR)<br>Treatment failure or death: OR 1.90 (95%CI 1.43–2.53; 22 studies, number of participants NR, I^2=87.3%, range NR)	Low
Jeon 2010	TB +DM	Outcomes available for individual studies, but not pooled: Prevalence*: , RR*	Critically low
Lutfiana 2019	TB +DM	Outcomes available for individual studies, but not pooled: Prevalence*: range 3.3%-100%	Critically low
Noubiap 2019	TB +DM	Prevalence:<br>Low income countries: 7.9% (95% CI 4.9%-11.5%, 15 studies, 9434 participants, I^2=96.8%, range NR)<br>Lower-middle income: 17.7% (95% CI 15.1%-20.5%, 48 studies, 48036 participants, I^2=98.3%, range NR)<br>Upper-middle income: 14.4% (95% CI 12.8%-16.0%, 75 studies, 1,994,027 participants, I^2=99.9%, range NR)<br><br>African region: 8.0% (95% CI 5.9%-10.4%, 119 studies, 474,944 participants, I^2=99.8%, range 1.9%-32.4%)<br>Southeast Asia: 19.0% (95% CI 16.2%-21.9%, 30 studies, 30382 participants, I^2=97.0%, range 5.1%-54.1%)<br><br>Benin: 1.9% (95% CI 0.2%-4.7%, 1 study, 159 participants)<br>Ethiopia: 18.8% (95% CI 1.9%-47.1%, 2 studies, 1749 participants, I^2=99.2%, range: 8.3%-32.4%)<br>Guinea-Bissau: 2.7% (95% CI 0.3%-6.8%, 1 study, 110 participants)<br>Nigeria: 7.8% (95% CI 4.4%-12.0%, 4 studies, 9821 participants, I^2=97.8%, range=4.8%-12.0%)<br>Senegal: 4.9% (95% CI 2.2%-8.5%, 2 studies, 2848 participants, I^2=75.1%, range=3.8%-7.0%)<br>South Africa: 9.4% (95% CI 7.6%-11.3%, 1 study, 947 participants)<br>Tanzania: 8.5% (95% CI 4.8%-13.0%, 7 studies, 4178 participants, I^2=95.1%, range=2.6%-16.7%)	Critically low

For peer review only - http://bmjopen.bmj.com/site/about/guidelines.xhtml

		Uganda: 7.3% (95% CI 4.7%-10.3%, 2 studies, 390 participants, I^2=9.9%, range=5.4%-8.5%)<br><br>Kazakhstan: 7.1% (95% CI 5.1%-9.4%, 1 study, 562 participants)<br>Romania: 18.4% (95% CI 13.6%-23.7%, 1 study, 228 participants)<br>Turkey: 7.8% (95% CI 6.8%-8.8%, 3 studies, 2773 participants, I2=0%, range=7.9%-8.6%)<br>Georgia: 12.4% (95% CI 7.4%-18.5%, 1 study, 137 participants)<br><br>Egypt: 22.8% (95% CI 15.2%-31.4%, 3 studies, 578 participants, I2=81.4%, range=15.8%-27.7%)<br>Iran: 17.8% (95% CI 12.5%-23.8%, 11 studies, 3134 participants, I2=93.3%, range=5.5%-40.0%)<br>Libya: 6.1% (95% CI 3.5%-9.4%, 1 study, 262 participants)<br>Pakistan: 22.0% (95% CI 12.8%-32.8%, 6 studies, 5201 participants, I2=98.8%, range=11.4%-39.6%)<br>Tunisia: 7.6% (95% CI 5.9%-9.6%, 1 study, 788 subject)<br>Yemen: 9.5% (95% CI 6.0%-13.8%, 1 study, 220 participants)<br><br>Guyana: 14.0% (95% CI 7.8%-21.6%, 1 study, 100 participants)<br>Mexico: 30.8% (95% CI 26.4%-35.3%, 10 studies, 192420 participants, I2=97.9%, range=19.3%-54.4%)<br><br>Brazil: 7.2% (95% CI 6.3%-8.1%, 12 study, 1726436 participants, I2=99.7%, range=3.3%-33.1%)<br>Peru: 4.8% (95% CI 1.7%-9.5%, 4 studies, 3983 participants, I2=96.8%, range=2.5%-11.1%)<br><br>Bangladesh: 10.6% (95% CI 7.2%-14.5%, 3 studies, 3010 participants, I2=85.9%, range=8.3%-12.8%)<br>India: 19.9% (95% CI 16.8%-23.2%, 26 studies, 27260 participants, I2=97.2%, range=5.1%-54.1%)<br>Sri Lanka: 24.1% (95% CI 16.6%-32.5%, 1 study, 112 participants)<br><br>China: 14.5% (95% CI 10.5%-19.0%, 14 studies, 19529 participants, I2=98.4%, range=2.7%-30.1%)<br>Fiji: 10.1% (95% CI 4.4%-17.7%, 3 studies, 1139 participants, I2=91.8%, range=5.2%-13.7%)<br>Indonesia: 14.8% (95% CI 12.2%-17.7%, 1 study, 634 participants)<br>Kiribati: 36.7% (95% CI 31.1%-42.5%, 1 study, 275 participants)<br>Malaysia: 26.9% (95% CI 17.8%-37.0%, 5 studies, 23438 participants)<br>Marshall Island: 45.2% (95% CI 32.9%-57.7%, 1 study, 62 participants)<br>Thailand: 7.5% (95% CI 6.2%-8.8%, 5 studies, 17862 participants, I2=81.6%, range=6.0%-16.3%)	
Shao-hua 2016	PTB +DM	Retreatment:<br>OR 2.05 (1.30-3.22, 3 studies, 499 participants, I^2=0%, range NR)<br>aOR 3.38 (1.56-7.29, 2 studies, n participants NR, I^2=75%, range NR)	Critically low
Tegegne 2018	TB +DM	Outcomes available for individual studies, but not pooled: Prevalence*: range 5% - 36%	High
Workneh 2017	TB +DM	Outcomes available for individual studies, but not pooled: Prevalence*: range 1.9% - 45%	Low
**TB+Mental disorders**			
Alene 2018	MDR-TB + Depression; MDR-TB +Anxiety; MDR-TB +Psychosis	Depression:<br>Overall: Prev. 25% (95% CI 14%-39%, 15 studies, n participants NR, I^2=98%, range= 3%-79%)<br>African region: 16% (95% CI 9%-24%, 3 studies, n participants NR, I^2 NR, range NR)<br>The Americas Region: 36% (95% CI 23%-50%, 3 studies, n participants NR, I^2 NR, range NR)<br>South-East Asia Region: 22% (95% CI 0%-60%, 3 studies, n participants NR, I^2 NR, range NR)<br>European region: 11% (95% CI 4%-21%, 3 studies, n participants NR, I^2 NR, range NR)<br>Eastern Mediterranean Region: 73% (95% CI 64%-81%, 2 studies, n participants NR, I^2 NR, range NR)<br>Western Pacific Region: 5% (95% CI 1%-12%, 1 study, , n participants NR, I^2 NR, range NR)	Critically low

For peer review only - http://bmjopen.bmj.com/site/about/guidelines.xhtml

		Anxiety:<br>Overall: Prev: 24% (95% CI 2%-57%, 3 studies, n participants NR, I^2=95%, range=12%-56%)<br>The Americas Region: 14% (95% CI 9%-21%, 2 studies, n participants NR, I^2 NR, range NR)<br>South-East Asia Region: 56% (95% CI 45%-66%, 1 studies, n participants NR, I^2 NR, range NR)<br><br>Psychosis:<br>(Overall includes a study from S.Korea)<br>African region: 12% (95% CI 8%-17%, 5 studies, n participants NR, I^2 NR, range NR)<br>The Americas Region: 11% (95% CI 7%-17%, 2 studies, n participants NR, I^2 NR, range NR)<br>South-East Asia Region: 10% (95% CI 5%-17%, 2 studies, n participants NR, I^2 NR, range NR)<br>European region: 6% (95% CI 0%-17%, 2 studies, n participants NR, I^2 NR, range NR)<br>Eastern Mediterranean Region: 7% (95% CI 1%-17%,1 studies, n participants NR, I^2 NR, range NR)	
Duko 2020	TB + Depression; MDR-TB +Depression	Prevalence: 45.19% (95% CI 38.04%-52.55%, 25 studies, 4903 participants, I^2=96.28%, range=15.56%-80.00%)<br>Women: 51.54% (95% CI 40.34%–62.60%, 17 studies, number of participants NR, I^2 = 92. 55%, range NR)<br>Men: 45.25% (95% CI 35.19%–55.71%, 17 studies, number of participants NR, I^2 = 95.09%, range NR)<br>MDR-TB: 52.34% (95% CI 38.09%-66.22%, 5 studies, number of participants NR, I^2=92.55%, range=NR)	High
Lee 2020	TB +Mental Disorders	Unsuccessful treatment: OR 2.13 (95%CI 0.85-5.37, 4 studies, 1196 participants, I^2=82%, range NR)<br>Loss to follow up: OR 1.90 (95%CI 0.33-10.91, 2 studies, 1139 participants, I^2=78%, range NR)<br>Non-adherence to treatment (measured by self-report, missed visits, pill count, or physiological tests): OR 1.60 (95% CI 0.84-3.02, 4 studies, 10851 participants, I^2=86%, range=0.94-3.67)<br>Outcomes available for individual studies, but not pooled: Prevalence*: Depression: range 37.5% - 53.9%; Mental disorder: range 18.9%-22.4%; Psychological distress: range 22% - 67.6%; PTSD: 29.6% (1 study), Mortality*:	Critically low
Rensburg 2020	TB +Mental illness (Depression, Anxiety, Alcohol use, and General Mental health)	Outcomes available for individual studies, but not pooled: Prevalence*: Depression: range 9.3% - 84%; Anxiety: range 2%-47.2%; Alcohol use: range 5% - 63%; Psychiatric comorbidity: range 3%; Psychological distress: range 22% - 83.6%; Poor mental quality: range 13.1% (1 study); Common mental disorder/Mental disorder: range 22.4%-38.3%	Critically low
Ruiz-Grosso 2020	TB +Depression	Mortality or loss to follow-up: OR = 4.26 (95% CI 2.33–7.79, 2 studies, 973 participants, I^2=0%, range=3.65-4.88)<br>Mortality: OR 2.85 (1.52-5.36, 2 studies, 973 participants, I^2=0%, range=1.76-2.99)<br>Loss to follow up: OR 8.70 (4.95-9.09, 2 studies, 973 participants, I^2=0%, range=4.95-9.09)<br>Non-adherence to TB treatment: OR 1.38 (0.70-2.72, 3 studies, 9349 participants, I^2=94.36%, range=0.92-3.67)	Critically low
**TB+HCV**			
Behzadifar 2019	TB +HCV	Africa: 11% (95% CI 1%-23%, 3 studies, 327 participants, I^2=93.9%, range=NR)	Moderate
**TB+other**			
Basham 2020	TB + cardiovascular disease	Outcomes available for individual studies, but not pooled: Mortality*: range of ORs 2.50 - 3.01	Critically low
Leung 2020	TB +Lung cancer; TB +non-Hodgkin's lymphoma; TB +Leukaemia	Upper middle-income countries<br>Lung cancer: RR 1.53 (95% CI 1.25-1.87, 9 studies, number of participants NR, I^2=94.6%, range NR)<br>non-Hodgkin's lymphoma: RR 1.70 (95% CI 1.13-2.56, 1 study, number of participants NR, I^2=NA)<br>leukaemia: RR 1.61 (95% CI 1.13-2.29, 1 study, number of participants NR, I^2= NA)	Low
Rehm 2009	TB+ Alcohol Use Disorder	Outcomes available for individual studies, but not pooled: Prevalence*:	Critically low

For peer review only - http://bmjopen.bmj.com/site/about/guidelines.xhtml

Note: Quality was assessed using the AMSTAR2 tool. aOR: adjusted OR; DM: Diabetes Mellitus; DR-TB: Drug resistant TB; HCV: Hepatitis C Virus; HIV: Human immunodeficiency virus; LMIC: Low- and middle-income countries; MDR-TB: Multidrug resistant TB; NR: Not reported; OR: Odds Ratio; prev.: prevalence; PTB: Pulmonary TB; RR: Relative Risk; SSA: Sub-Saharan Africa; Tuberculosis; XDR-TB: Extensively drug-resistant TB.

* Outcomes available for individual studies, but not pooled. Range of effect estimates reported.

† Includes one study focused on children

For peer review only - http://bmjopen.bmj.com/site/about/guidelines.xhtml

Supplementary Table 5: Conditions that were considered chronic (and therefore included) or not (and therefore excluded) for this review

Condition/risk factor	Should be considered as a comorbidity in this review?	Description or details
HIV	Yes	
Diabetes mellitus (DM)	Yes	
smoking	No	Would not fall under 'substance abuse', even if it is reported as 'high dependence smoking'
Drinking alcohol	No	No, unless it is something like 'harmful use of alcohol' or 'alcohol dependence'
Substance use / drug abuse	Yes	usually reserved for illicit substances
Cavitary disease	No	
Acute Respiratory Distress Syndrome	No	Acute
Leukopenia	no	
Chronic diarrhoea	no	Unless it's inflammatory bowel disorders, no.
Malnourishment	No	
Obesity	No	
BMI	No	
Anaemia	No	
drug-induced hepatotoxicity/ liver injury	No	
Intestinal parasites	No	
hypothyroidism	Yes	
hyperthyroidism	Yes	
hypokalaemia	No	
hearing defect	Yes	
Hypertension	No	
Dyslipidemia	No	
Skin conditions	no	
deep venous thrombosis	NO	
sacroiliitis	NO	
severe epistaxis	No	
seizures (cause not determined)	Yes if called epilepsy	Yes only if called epilepsy
haemorrhoids/fistula-in-ano	No	
dermatitis	No	
cerebrovascular accident	Yes	
scabies	No	
pneumothorax	No	
pancreatitis	No	Unless Long term pancreatitis and it is clearly specified
cryptococcal IRIS (immune reconstitution inflammatory syndrome) adenitis	No	
cryptoccocus	No	complications of HIV
visual impairment (reported as a symptom of DM)	No (reported as a complication)	
pulmonary fungal infection	No	complications of HIV
Candida coinfection	No	complications of HIV
Aspergilius coinfection	No	complications of HIV

For peer review only - http://bmjopen.bmj.com/site/about/guidelines.xhtml

chronic airflow obstruction	Yes	As a proxy for COPD
HTLV (Human T-Cell LymphotropicVirus)	No	complications of HIV
pneumoconiosis	Yes	
hyponatremia	No	complications of HIV
Hepatitis B virus (HBV)	Yes	
Hepatitis C virus (HCV)	Yes	
Acquired immunodeficiency syndrome (AIDS)	Yes	
Autoimmune diseases	Yes	
Arthritis	Yes	
T. pallidum	Yes	
Cancer	Yes	Any type of cancer
Heart disease / Cardiopathies	Yes	
cardiomyopathy	No	Can be acute and/or reversible
Heart failure	Yes	
Cardiovascular disease	Yes	
cerebrovascular accident	Yes	Cerebrovascular disease is a form of cardiovascular disease
Chronic kidney disease / Chronic renal failure	Yes	No if acute renal failure- Yes if chronic renal failure
Chronic liver disease / Cirrhosis / Chronic hepatic dysfunction	Yes	
Depression	Yes	Clinical diagnostic or assessed with a validated scale (e.g. PHQ-9)
Anxiety	Yes	Clinical diagnostic of an anxiety disorder or assessed with a validated scale
Panic disorder	Yes	
Obsessive compulsive disorder (OCD)	Yes	
Post-traumatic stress disorder (PTSD)	Yes	
Mental disorder	Yes	Umbrella term for conditions such as PTSD, OCD, depression, anxiety disorders, etc.
Pneumonia	No	Acute condition
Chronic obstructive pulmonary disease (COPD)	Yes	COPD is form of chronic lung disease
Chronic lung disease	Yes	
Pulmonary edema	No	Symptom
Asthma	Yes	
Cor pulmonale	Yes	
Unstable angina	Yes	
Chronic corticosteroid therapy	No	Treatment

For peer review only - http://bmjopen.bmj.com/site/about/guidelines.xhtml

Supplementary Table 6: AMSTAR2 assessment details for each study

**Study Id**	**1**	**2**	**3**	**4**	**5**	**6**	**7**	**8**	**9**	**10**	**11**	**12**	**13**	**14**	**15**	**16**	**confidence rating**
Duko 2020	Yes	**Partial Yes**	Yes	**Partial Yes**	Yes	Yes	**Partial Yes**	Yes	**Yes**	No	**Yes**	Yes	**Yes**	Yes	**Yes**	Yes	High
Alemu 2020	Yes	**Partial Yes**	Yes	**Partial Yes**	Yes	Yes	**No**	Yes	**Yes**	No	**Yes**	Yes	**Yes**	Yes	**Yes**	Yes	Low
Arega 2020	Yes	**No**		**Partial Yes**	Yes	Yes	**No**	Yes	**Yes**	No	**Yes**	Yes	**Yes**	Yes	**Yes**	Yes	Critically low
Huddart 2020	Yes	**No**	Yes	**Partial Yes**	Yes	Yes	**Partial Yes**	Partial Yes	**No**	No	**No**	Yes	**Yes**	Yes	**No**	Yes	Critically low
Huang 2020	Yes	**Partial Yes**	Yes	**No**	Yes	Yes	**No**	Yes	**Yes**	No	**Yes**	Yes	**Yes**	Yes	**Yes**	Yes	Critically low
Edessa 2020	Yes	**Partial Yes**	Yes	**Partial Yes**	Yes	Yes	**Partial Yes**	Yes	**Partial Yes**	No		Yes	**Yes**	Yes	**Yes**	Yes	High
Basham 2020	Yes	**No**	Yes	**Partial Yes**	Yes	Yes	**No**	Yes	**Partial Yes**	No	**No**	Yes	**Yes**	Yes			Critically low
Bisson 2020	Yes	**Yes**	Yes	**Partial Yes**	No	No	**Partial Yes**	No	**No**	No	**NA**	NA	**NA**	No	**NA**	Yes	Low
Huangfu 2019	Yes	**Partial Yes**	Yes	**Partial Yes**	Yes	Yes	**Partial Yes**	Partial Yes	**Partial Yes**	No	**Yes**	Yes	**Yes**	Yes	**No**	Yes	Low
Bastos 2019	Yes	**Yes**	Yes	**Partial Yes**	Yes	Yes	**No**	Partial Yes	**No**	No	**NA**	NA	**NA**	No	**NA**	Yes	Critically low
Endalamaw 2019		**Partial Yes**	Yes	**Partial Yes**	No	Yes	**Partial Yes**	Partial Yes	**Partial Yes**	No	**Yes**	Yes	**Yes**	No	**Yes**	Yes	Moderate
Chem 2019	Yes	**Partial Yes**	Yes	**Partial Yes**	Yes	No	**Partial Yes**	Yes	**Partial Yes**	Yes	**Yes**	Yes	**No**	Yes	**No**	Yes	Critically low
Alebel 2019	Yes	**Yes**	Yes	**Partial Yes**	No	Yes	**No**	Partial Yes	**Yes**	No	**Yes**	Yes	**Yes**	Yes	**Yes**	Yes	Low
Behzadifar 2019	Yes	**Partial Yes**	Yes	**Partial Yes**	Yes	No	**Partial Yes**	Partial Yes	**Yes**	No	**Yes**	Yes	**Yes**	Yes	**Yes**	Yes	Moderate
Gelaw 2019	Yes	**Partial Yes**	Yes	**Partial Yes**	Yes	Yes	**Partial Yes**	Yes	**Yes**	No	**Yes**	Yes	**Yes**	Yes	**Yes**	Yes	High
Alene 2018		**Partial Yes**	Yes	**Partial Yes**	Yes	Yes	**Partial Yes**	Yes	**No**	No	**No**	No	**No**	No	**No**	Yes	Critically low
Almeida 2018	Yes	**Partial Yes**	Yes	**Partial Yes**	Yes	Yes	**Partial Yes**	Partial Yes	**No**	No	**No**	No	**No**	No	**No**	Yes	Critically low
Eshetie 2018		**No**	Yes	**Partial Yes**	Yes	Yes	**No**	Yes	**Yes**	No	**Yes**	Yes	**Yes**	Yes	**No**	Yes	Critically low
Gao 2013	Yes	**No**	Yes	**Partial Yes**	Yes	Yes	**Partial Yes**	Partial Yes	**No**	No	**Yes**	No	**No**	Yes	**No**	Yes	Critically low
	Yes	**No**		**Partial Yes**	No	No	**Partial Yes**	Partial Yes	**No**	No	**NA**	NA	**NA**	No	**NA**	No	Critically low
Baker 2011	Yes	**No**	Yes	**Partial Yes**	No	Yes	**Partial Yes**	Partial Yes	**No**	No	**Yes**	Yes	**No**	Yes	**Yes**	Yes	Critically low
Jeon 2010	Yes	**No**	Yes	**Partial Yes**	No	Yes	**No**	Yes	**No**	No	**NA**	NA	**NA**	No	**NA**	Yes	Critically low
Gao 2010	Yes	**No**	Yes	**Partial Yes**	No	No	**Yes**	Yes	**No**	No	**No**	No	**No**	Yes	**No**	Yes	Critically low

For peer review only

	Yes	**Partial Yes**	Yes	**Partial Yes**	Yes	Yes	**Partial Yes**	Yes	**No**	No	**No**	No	**No**	Yes	**Yes**	Yes	Critically low
Leung 2020	Yes	**Partial Yes**	Yes	**Partial Yes**	Yes	Yes	**Yes**	Yes	**Partial Yes**	No	**No**	Yes	**Yes**	Yes	**Yes**	Yes	Low
Rensburg 2020	Yes	**No**	Yes	**Partial Yes**	Yes	No	**No**	Partial Yes	**No**	No	**NA**	NA	**NA**	No	**NA**	Yes	Critically low
Han 2016	Yes	**No**	Yes	**Partial Yes**	Yes	Yes	**Partial Yes**	No	**No**	No	**No**	No	**No**	Yes	**Yes**	No	Critically low
Lee 2020	Yes	**Yes**	Yes	**Partial Yes**	Yes	Yes	**Partial Yes**	Yes	**Yes**	No	**Yes**	No	**No**	No	**No**	Yes	Critically low
Purmohamad 2020	Yes	**No**	Yes	**Partial Yes**	Yes	Yes	**Partial Yes**	Partial Yes	**No**	No	**No**	No	**No**	Yes	**Yes**	Yes	Critically low
Rajendran 2020	Yes	**No**	Yes	**Partial Yes**	No	No	**Yes**	No	**No**	No	**NA**	NA	**NA**	No	**NA**	Yes	Critically low
Ruiz-Grosso 2020	Yes	**Partial Yes**	Yes	**No**	Yes	Yes	**No**	No	**No**	No	**Yes**	Yes	**No**	No	**No**	Yes	Critically low
Pourakbari 2019	Yes	**No**	Yes	**Partial Yes**	Yes	Yes	**No**	Yes	**No**	No	**Yes**	No	**Yes**	Yes	**Yes**	Yes	Critically low
Lutfiana 2019	Yes	**No**	Yes	**Partial Yes**	No	No	**Partial Yes**	Partial Yes	**No**	No	**NA**	NA	**NA**	No	**NA**	Yes	Critically low
Wang 2019		**No**	Yes	**Partial Yes**	Yes	Yes	**Partial Yes**	Yes	**No**	No	**Yes**	No	**No**	Yes	**Yes**	Yes	Critically low
Uchida 2019	Yes	**No**	Yes	**No**	Yes	No	**No**	Partial Yes	**No**	No	**NA**	NA	**NA**	No	**NA**	Yes	Critically low
Mekonnen 2019	Yes	**Partial Yes**	Yes	**Partial Yes**	Yes	Yes	**Partial Yes**	Yes	**Partial Yes**	No	**Yes**	Yes	**Yes**	Yes	**Yes**	Yes	High
Teweldemedhin 2018	Yes	**No**	Yes	**No**	Yes	No	**Yes**	Partial Yes	**No**	No	**Yes**	No	**No**	Yes	**Yes**	Yes	Critically low
Tegegne 2018	Yes	**Partial Yes**	Yes	**Partial Yes**	Yes	Yes	**Yes**	Partial Yes	**Yes**	No	**Yes**	Yes	**Yes**	Yes	**Yes**	Yes	High
Tesfaye 2018	Yes	**Partial Yes**	Yes	**Partial Yes**	Yes	Yes	**No**	Partial Yes	**No**	No	**Yes**	Yes	**No**	Yes	**Yes**	Yes	Critically low
Seid 2018	Yes	**No**	Yes	**Partial Yes**	Yes	Yes	**Partial Yes**	Partial Yes	**No**	No	**No**	No	**No**	No	**Yes**	Yes	Critically low
Pormohammad 2018	Yes	**No**	Yes	**Partial Yes**	Yes	Yes	**Partial Yes**	Partial Yes	**No**	No	**No**	Yes	**Yes**	No	**Yes**	Yes	Critically low
Samuels 2018	Yes	**Partial Yes**	Yes	**Partial Yes**	Yes	Yes	**Partial Yes**	Yes	**No**	No	**Yes**	Yes	**Yes**	Yes	**Yes**	Yes	Low
Lukoye 2015	Yes	**No**	Yes	**Partial Yes**	Yes	Yes	**Partial Yes**	Yes	**No**	No	**No**	No	**No**	No	**Yes**	Yes	Critically low
Mesfin 2014	Yes	**No**	Yes	**Partial Yes**	Yes	Yes	**Partial Yes**	Yes	**Partial Yes**	No	**No**	No	**No**	Yes	**Yes**	Yes	Critically low
Straetemans 2011	Yes	**Partial Yes**	Yes	**Partial Yes**	No	No	**Yes**	Partial Yes	**No**	No	**Yes**	No	**No**	Yes	**Yes**	Yes	Critically low
Waitt 2011	Yes	**No**	Yes	**No**	No	No	**Partial Yes**	Partial Yes	**No**	No	**No**	NA	**No**	No	**No**	No	Critically low
Reddy 2010	Yes	**No**	No	**Partial Yes**	Yes	Yes	**Yes**	No	**No**	No	**NA**	NA	**NA**	No	**NA**	Yes	Critically low
Rehm 2009	Yes	**No**	Yes	**Partial Yes**	Yes	No	**No**	Partial Yes	**No**	No	**NA**	NA	**NA**	No	**NA**	Yes	Critically low

For peer review only

Sotgiu 2009	Yes	**No**	Yes	**Partial Yes**	Yes	No	**No**	Partial Yes	**No**	No	**NA**	NA	**NA**	No	**NA**	Yes	Critically low
Noubiap 2019		**Partial Yes**	Yes	**Partial Yes**	Yes	Yes	**Partial Yes**	Partial Yes	**No**	No	**No**	Yes	**Yes**	Yes	**Yes**	Yes	Critically low
Wu 2016	Yes	**No**	Yes	**Partial Yes**	No	No	**Partial Yes**	Yes	**No**	No	**Yes**	Yes	**No**	Yes	**No**	Yes	Critically low
Gautam 2021	Yes	**Partial Yes**	Yes	**Partial Yes**	Yes	Yes	**Yes**	Partial Yes	**Partial Yes**	No	**No**	No	**Yes**	Yes	**Yes**	Yes	Low
Chen 2013	Yes	**No**	Yes	**Partial Yes**	Yes	Yes	**Partial Yes**	Partial Yes	**No**	No	**No**	Yes	**Yes**	Yes	**Yes**	No	Critically low
McMurry 2018	Yes	**Partial Yes**	Yes	**Partial Yes**	**Yes**	No	**Partial Yes**	Partial Yes	**No**	No	**NA**	NA	**NA**	No	**NA**	yes	Low
Workneh 2017	Yes	**Partial Yes**	**Yes**	**Partial Yes**	No	No	**Partial Yes**	**Partial Yes**	**No**	No	**NA**	NA	**NA**	no	**NA**	Yes	Low
Shao-hua 2016	Yes	**No**	Yes	**Partial Yes**	Yes	Yes	**Partial Yes**	Partial Yes	**No**	No	**No**	No	**No**	Yes	**No**	No	Critically low

Note: Items assessing critical domains are bolded.
1. Did the research questions and inclusion criteria for the review include the components of PICO?
2. Did the report of the review contain an explicit statement that the review methods were established prior to conduct of the review and did the report justify any significant deviations from the protocol
3. Did the review authors explain their selection of the study designs for inclusion in the review?
4. Did the review authors use a comprehensive literature search strategy?
5. Did the review authors perform study selection in duplicate?
6. Did the review authors perform data extraction in duplicate?
7. Did the review authors provide a list of excluded studies and justify the exclusions?
8. Did the review authors describe the included studies in adequate detail?
9. Did the review authors use a satisfactory technique for assessing the risk of bias (RoB) in individual studies that were included in the review?
10. Did the review authors report on the sources of funding for the studies included in the review?
11. If meta-analysis was performed did the review authors use appropriate methods for statistical combination of results?
12. If meta-analysis was performed, did the review authors assess the potential impact of RoB in individual studies on the results of the meta-analysis or other evidence synthesis?
13. Did the review authors account for RoB in individual studies when interpreting/discussing the results of the review?
14. Did the review authors provide a satisfactory explanation for, and discussion of, any heterogeneity observed in the results of the review?
15. If they performed quantitative synthesis did the review authors carry out an adequate investigation of publication bias (small study bias) and discuss its likely impact on the results of the review?
16. Did the review authors report any potential sources of conflict of interest, including any funding they received for conducting the review?

For peer review only

PRISMA 2020 Checklist

Section and Topic	Item #	Checklist item	Location where item is reported
**TITLE**			
Title	1	Identify the report as a systematic review.	
**ABSTRACT**			
Abstract	2	See the PRISMA 2020 for Abstracts checklist.	
**INTRODUCTION**			
Rationale	3	Describe the rationale for the review in the context of existing knowledge.	
Objectives	4	Provide an explicit statement of the objective(s) or question(s) the review addresses.	
**METHODS**			
Eligibility criteria	5	Specify the inclusion and exclusion criteria for the review and how studies were grouped for the syntheses.	
Information sources	6	Specify all databases, registers, websites, organisations, reference lists and other sources searched or consulted to identify studies. Specify the date when each source was last searched or consulted.	
Search strategy	7	Present the full search strategies for all databases, registers and websites, including any filters and limits used.	
Selection process	8	Specify the methods used to decide whether a study met the inclusion criteria of the review, including how many reviewers screened each record and each report retrieved, whether they worked independently, and if applicable, details of automation tools used in the process.	
Data collection process	9	Specify the methods used to collect data from reports, including how many reviewers collected data from each report, whether they worked independently, any processes for obtaining or confirming data from study investigators, and if applicable, details of automation tools used in the process.	
Data items	10a	List and define all outcomes for which data were sought. Specify whether all results that were compatible with each outcome domain in each study were sought (e.g. for all measures, time points, analyses), and if not, the methods used to decide which results to collect.	
	10b	List and define all other variables for which data were sought (e.g. participant and intervention characteristics, funding sources). Describe any assumptions made about any missing or unclear information.	
Study risk of bias assessment	11	Specify the methods used to assess risk of bias in the included studies, including details of the tool(s) used, how many reviewers assessed each study and whether they worked independently, and if applicable, details of automation tools used in the process.	
Effect measures	12	Specify for each outcome the effect measure(s) (e.g. risk ratio, mean difference) used in the synthesis or presentation of results.	
Synthesis methods	13a	Describe the processes used to decide which studies were eligible for each synthesis (e.g. tabulating the study intervention characteristics and comparing against the planned groups for each synthesis (item #5)).	
	13b	Describe any methods required to prepare the data for presentation or synthesis, such as handling of missing summary statistics, or data conversions.	
	13c	Describe any methods used to tabulate or visually display results of individual studies and syntheses.	
	13d	Describe any methods used to synthesize results and provide a rationale for the choice(s). If meta-analysis was performed, describe the model(s), method(s) to identify the presence and extent of statistical heterogeneity, and software package(s) used.	
	13e	Describe any methods used to explore possible causes of heterogeneity among study results (e.g. subgroup analysis, meta-regression).	
	13f	Describe any sensitivity analyses conducted to assess robustness of the synthesized results.	
Reporting bias assessment	14	Describe any methods used to assess risk of bias due to missing results in a synthesis (arising from reporting biases).	
Certainty assessment	15	Describe any methods used to assess certainty (or confidence) in the body of evidence for an outcome.	

For peer review only

For peer review only - http://bmjopen.bmj.com/site/about/guidelines.xhtml

PRISMA 2020 Checklist

Section and Topic	Item #	Checklist item	Location where item is reported
**RESULTS**			
Study selection	16a	Describe the results of the search and selection process, from the number of records identified in the search to the number of studies included in the review, ideally using a flow diagram.	
	16b	Cite studies that might appear to meet the inclusion criteria, but which were excluded, and explain why they were excluded.	
Study characteristics	17	Cite each included study and present its characteristics.	
Risk of bias in studies	18	Present assessments of risk of bias for each included study.	
Results of individual studies	19	For all outcomes, present, for each study: (a) summary statistics for each group (where appropriate) and (b) an effect estimate and its precision (e.g. confidence/credible interval), ideally using structured tables or plots.	
Results of syntheses	20a	For each synthesis, briefly summarise the characteristics and risk of bias among contributing studies.	
	20b	Present results of all statistical syntheses conducted. If meta-analysis was done, present for each the summary estimate and its precision (e.g. confidence/credible interval) and measures of statistical heterogeneity. If comparing groups, describe the direction of the effect.	
	20c	Present results of all investigations of possible causes of heterogeneity among study results.	
	20d	Present results of all sensitivity analyses conducted to assess the robustness of the synthesized results.	
Reporting biases	21	Present assessments of risk of bias due to missing results (arising from reporting biases) for each synthesis assessed.	
Certainty of evidence	22	Present assessments of certainty (or confidence) in the body of evidence for each outcome assessed.	
**DISCUSSION**			
Discussion	23a	Provide a general interpretation of the results in the context of other evidence.	
	23b	Discuss any limitations of the evidence included in the review.	
	23c	Discuss any limitations of the review processes used.	
	23d	Discuss implications of the results for practice, policy, and future research.	
**OTHER INFORMATION**			
Registration and protocol	24a	Provide registration information for the review, including register name and registration number, or state that the review was not registered.	
	24b	Indicate where the review protocol can be accessed, or state that a protocol was not prepared.	
	24c	Describe and explain any amendments to information provided at registration or in the protocol.	
Support	25	Describe sources of financial or non-financial support for the review, and the role of the funders or sponsors in the review.	
Competing interests	26	Declare any competing interests of review authors.	
Availability of data, code and other materials	27	Report which of the following are publicly available and where they can be found: template data collection forms; data extracted from included studies; data used for all analyses; analytic code; any other materials used in the review.	

From: Page MJ, McKenzie JE, Bossuyt PM, Boutron I, Hoffmann TC, Mulrow CD, et al. The PRISMA 2020 statement: an updated guideline for reporting systematic reviews. BMJ 2021;372:n71. doi: 10.1136/bmj.n71

For peer review only - http://bmjopen.bmj.com/site/about/guidelines.xhtml

For more information, visit: http://www.prisma-statement.org/

For peer review only

BMJ Open

Prevalence and risks of tuberculosis multimorbidity in low- and middle-income countries: a meta-review

Journal:	*BMJ Open*
Manuscript ID	bmjopen-2022-060906.R1
Article Type:	Original research
Date Submitted by the Author:	08-Aug-2022
Complete List of Authors:	Jarde, Alexander; University of York,<br>Romano, Eugenia; King's College London Institute of Psychiatry Psychology and Neuroscience, Department of Psychological Medicine<br>Afaq, Saima; Khyber Medical University, Institute of Public Health and Social Sciences; Imperial College London, Department of Epidemiology and Biostatistics<br>Elsony, Asma; Epi-Lab, Public Health<br>Lin, Yan; International Union Against Tuberculosis and Lung Disease<br>Huque, Rumana; University of Dhaka, Economics<br>Elsey, Helen ; University of York; Hull York Medical School<br>Siddiqi, Kamran; University of York, Institute of Health Sciences; Hull York Medical School<br>Stubbs, B; King's College London Institute of Psychiatry Psychology and Neuroscience, Department of Psychological Medicine<br>Siddiqi, Najma; University of York, Department of Health Sciences; Hull York Medical School
**Primary Subject Heading**:	Global health
Secondary Subject Heading:	Public health, Epidemiology
Keywords:	Epidemiology < INFECTIOUS DISEASES, Public health < INFECTIOUS DISEASES, Tuberculosis < INFECTIOUS DISEASES

SCHOLARONE™ Manuscripts

I, the Submitting Author has the right to grant and does grant on behalf of all authors of the Work (as defined in the below author licence), an exclusive licence and/or a non-exclusive licence for contributions from authors who are: i) UK Crown employees; ii) where BMJ has agreed a CC-BY licence shall apply, and/or iii) in accordance with the terms applicable for US Federal Government officers or employees acting as part of their official duties; on a worldwide, perpetual, irrevocable, royalty-free basis to BMJ Publishing Group Ltd ("BMJ") its licensees and where the relevant Journal is co-owned by BMJ to the co-owners of the Journal, to publish the Work in this journal and any other BMJ products and to exploit all rights, as set out in our licence.

The Submitting Author accepts and understands that any supply made under these terms is made by BMJ to the Submitting Author unless you are acting as an employee on behalf of your employer or a postgraduate student of an affiliated institution which is paying any applicable article publishing charge ("APC") for Open Access articles. Where the Submitting Author wishes to make the Work available on an Open Access basis (and intends to pay the relevant APC), the terms of reuse of such Open Access shall be governed by a Creative Commons licence – details of these licences and which Creative Commons licence will apply to this Work are set out in our licence referred to above.

Other than as permitted in any relevant BMJ Author's Self Archiving Policies, I confirm this Work has not been accepted for publication elsewhere, is not being considered for publication elsewhere and does not duplicate material already published. I confirm all authors consent to publication of this Work and authorise the granting of this licence.

For peer review only

Prevalence and risks of tuberculosis multimorbidity in low- and middle-income countries: a meta-review

Running title: Meta-review of TB multimorbidity

Authors

Alexander Jarde, PhD - Department of Health Sciences, University of York, York, United Kingdom.
Eugenia Romano, PhD - Department of Psychological Medicine, Institute of Psychiatry, Psychology & Neuroscience, King's College London, London, United Kingdom.
Saima Afaq, PhD - Institute of Public Health and Social Sciences, Khyber Medical University, Peshawar, Pakistan.
Asma Elsony, PhD - Epi-Lab: The Epidemiological Laboratory, Khartoum, Sudan.
Yan Lin, MD - International Union Against Tuberculosis and Lung Disease, China.
Rumana Huque, Prof - Department of Economics, University of Dhaka, Dhaka, Bangladesh.
Helen Elsey, PhD - Department of Health Sciences, University of York, and Hull York Medical School, York, United Kingdom.
Kamran Siddiqi, PhD - Department of Health Sciences, University of York, and Hull York Medical School, York, United Kingdom.
Brendon Stubbs, PhD - Department of Psychological Medicine, Institute of Psychiatry, Psychology & Neuroscience, King's College London, London, United Kingdom.
Najma Siddiqi, PhD - Department of Health Sciences, University of York, and Hull York Medical School, York, United Kingdom.

Correspondence to: Alexander Jarde: a.jarde@gmail.com; Department of Health Sciences, University of York, Heslington, York YO10 5DD, United Kingdom. Telephone: 0190432 1327

Word count: 3528

For peer review only

For peer review only - http://bmjopen.bmj.com/site/about/guidelines.xhtml

Abstract (264 / 300)

Objectives: Co-occurrence of tuberculosis (TB) with other chronic conditions (TB multimorbidity) increases complexity of management and adversely affects health outcomes. We aimed to map the prevalence of the co-occurrence of one or more chronic conditions in people with tuberculosis (TB) and associated health risks by systematically reviewing previously published systematic reviews.

Design: Systematic review of systematic reviews (meta-review).

Setting: Low- and middle-income countries (LMIC).

Papers: We searched in Medline, Embase, PsycINFO, Social Sciences Citation Index, Science Citation Index, Emerging Sources Citation Index and Conference Proceedings Citation Index, and the WHO Global Index Medicus from inception to 23/10/2020, contacted authors, and reviewed reference lists. Pairs of independent reviewers screened titles, abstracts and full texts, extracted data, and assessed the included reviews' quality (AMSTAR2). We included systematic reviews reporting data for people in LMIC with TB multimorbidity and synthesised them narratively. We excluded reviews focused on children or specific subgroups (e.g. incarcerated people).

Primary and secondary outcome measures: Prevalence or risk of TB multimorbidity (primary); any measure of burden of disease (secondary).

Results: From the 7,557 search results, 54 were included, representing >6,296,000 people with TB. We found that the most prevalent conditions in people with TB were depression (45.19%, 95% Confidence Interval [CI] 38.04%-52.55%, 25 studies, 4,903 participants, I^2=96.28%, high quality), HIV (31.81%, 95%CI 27.83%-36.07%, 68 studies, 62,696 participants, I^2=98%, high quality), and diabetes mellitus (17.7%, 95%CI 15.1%-20.0.5%, 3 studies, 578 participants, I^2=81.4%, critically low quality).

Conclusions: We identified several chronic conditions that co-occur in a significant proportion of people with TB. Although limited by varying quality and gaps in the literature, this first meta-review of TB multimorbidity highlights the magnitude of additional ill health burden due to chronic conditions on people with TB.

Registration: Prospero CRD42020209012

Key words: Tuberculosis; Noncommunicable Diseases; Chronic Disease; Mental Disorders; Communicable Diseases; HIV; Diabetes Mellitus; Comorbidity; Multiple Chronic Conditions

Strengths and limitations of this study

- We did an extensive search strategy, including databases of grey literature and protocols.
- We summarised data synthesised at the country, regional (e.g. Eastern Sub-Saharan Africa), continental, and global (LMIC) level as long as the pooled estimate did not include data from high-income countries.
- Whenever there was an overlap between two reviews in terms of countries covered, TB comorbidities, and reported outcomes, we included the most complete one only if its quality, as assessed with the AMSTAR2 tool, was not lower than the other one.
- Although we had initially planned to re-do reported meta-analyses that included studies from high-income countries without these studies (to have pooled estimates from LMICs only), this was deemed unfeasible due to the high number of reviews where this would have been required.

For peer review only - http://bmjopen.bmj.com/site/about/guidelines.xhtml

For peer review only

For peer review only - http://bmjopen.bmj.com/site/about/guidelines.xhtml

INTRODUCTION

About 30% adults in developed countries experience multimorbidity, i.e. the co-occurrence of two or more chronic conditions (including non-communicable diseases [NCDs], chronic communicable diseases [CCDs] and mental disorders) in a single individual at one point in time. Multimorbidity is a growing global concern[1] and its prevalence is rising in low- and middle-income countries (LMICs),[2] as CCDs such as tuberculosis (TB) and HIV remain major public health issues.[3] and NCDs are increasing due to major demographic shifts, urbanization, changing environmental factors, economic empowerment and accompanying lifestyle changes [4–8] This shift away from risks for CCD in children towards those for NCD in adults is also reflected in the steady increase in the burden of disability-adjusted life years (DALYs) attributed to NCDs over the past decades,[9] reaching 34% in low-income countries, and up to 82% in middle-high-income countries in 2019.[10]

TB is one of the leading causes of mortality from a single infectious disease globally[9] and contributes 1.86% of the total worldwide DALYs and 2.54% of the total worldwide years of life lost (making it the 12th and 11th highest contributor, respectively).[4,10] TB frequently co-occurs with NCDs, including diabetes mellitus (DM, 2.79% of worldwide DALYs), depression (1.84% of worldwide DALYs), and cancer (neoplasms representing 9.93% of worldwide DALYs).[4,11] Depression[12] and DM[13] have been reported to be important risk factors for TB. Similarly, CCDs such as HIV (1.88% of worldwide DALYs) and TB adversely affect each other at the molecular, cellular, individual and population levels.[4,14]

We defined TB multimorbidity as the co-occurrence of TB and one or more chronic conditions (NCDs or CCDs).[15] This co-occurrence increases complexity of management and adversely affects health, economic and mortality outcomes, threatening the capacity for LMIC to achieve global public health targets. The cost and access to healthcare is of particular concern in LMICs, where the high costs relating to TB multimorbidity may further burden healthcare systems already under stress, and given the high out-of-pocket expenditure, it could lead to great financial burden for patients.

Numerous systematic reviews to date have considered individual chronic conditions in people with TB (e.g. [16–19]). However, no review has synthesised the evidence on a range of chronic conditions, their prevalence in people with TB and the burden associated with such co-occurrence of conditions. Understanding the overarching literature on TB multimorbidity is essential to enable better services to be developed to identify, prevent, and manage this common situation, which presents a significant health and financial burden to people with TB and to health services. Furthermore, differences in TB multimorbidity by gender, socio-economic group and country, which could shed further light on the problem, remain unclear.

The primary aim of this comprehensive meta-review of systematic reviews was to summarise and map the prevalence and risk of chronic conditions (CCD or NCD, alone or in combination) in people with TB in LMICs compared with people without TB, and to summarise the associated health outcomes (e.g. TB treatment success and measures of disease burden) in people with TB multimorbidity, compared with people with TB only.

For peer review only - http://bmjopen.bmj.com/site/about/guidelines.xhtml

METHODS

We have followed the PRISMA guidelines [20] in reporting this meta-review and its protocol was registered in the international prospective register of systematic reviews (PROSPERO, CRD42020209012).

Search strategy

We ran our search strategy in Medline (Ovid), Embase (Ovid), PsycINFO (Ovid), Social Sciences Citation Index (Web of Science), Science Citation Index (Web of Science), Emerging Sources Citation Index and Conference Proceedings Citation Index (Web of Science), and the WHO Global Index Medicus from inception to October 23rd, 2020. To identify unpublished studies, we also searched PROSPERO and the Open Grey database, and contacted authors of conference abstracts. Reference lists of included reviews were hand searched. We did not set any restrictions on the origin of the paper, date of publication, or language.

We used free text and controlled vocabulary (e.g., MeSH terms for Medline) for terms related to communicable, non-communicable, and mental diseases and combined them with terms for TB using Boolean operators: (CCD or NCD or mental disease) AND Tuberculosis. Supplementary Table 1 (Appendix 1) lists the search terms for Medline and the full search strategy can be found in Appendix 2.

Selection criteria

We included systematic reviews reporting data for people in LMICs, with any type of TB and one or more additional chronic conditions. This included, but was not limited to, heart disease, DM, arthritis, chronic obstructive pulmonary disease (COPD), HIV, Hepatitis B (HBV) and Hepatitis C (HCV), depression, and anxiety disorders (as defined by review authors). As there is no clear and widely used definition of what constitutes a chronic condition,[21] whenever there were doubts, four of the authors with clinical/research expertise (KS, NS, HE, BS) decided by consensus if a disorder was to be considered as a chronic condition. Conditions considered side effects of TB medications, such as nausea or diarrhoea, were not considered chronic conditions for this review.

After registering the protocol, the following additional changes were made: First, we decided to limit our population of interest to the general TB population, excluding studies that stated focusing on children. Second, we decided to exclude studies focused on specific subgroups (e.g., incarcerated people, health care workers, etc.), focussing on populations for which results are more readily generalisable. Studies in patients with a specific type of TB (e.g., extra-pulmonary TB) were, however, considered eligible.

Included systematic reviews had to report either pooled or individual study data for at least one of our primary or secondary outcomes. Narrative, non-systematic reviews and systematic reviews focused only on high-income countries (HIC) were excluded.

Primary outcomes

The co-primary outcomes included prevalence (or incidence) of each chronic condition (or combination of more than one condition) in people with TB, and odds ratios (or other comparative statistic) of having a chronic condition (or combination of conditions) in people with TB compared to those without TB.

Secondary outcomes

For peer review only - http://bmjopen.bmj.com/site/about/guidelines.xhtml

Secondary outcomes included any measure of disease burden in people with TB multimorbidity, such as mortality, loss to follow up (treatment interrupted for two consecutive months or more), treatment failure (sputum smear or culture remained positive at month five or later during treatment), treatment completion (without evidence of failure, but with no record of being cured), cured (smear- or culture-negative patients in the last month of treatment and on at least one previous occasion), successful treatment (patients who were cured or who completed treatment), or unsuccessful treatment (patients who were lost to follow-up, had treatment failure, or died).[22,23] Other secondary outcomes of interest included years of life lived with disability, years of life lost, DALYs, outcomes related to the additional chronic conditions, and any other reported measure of disease burden.

Study selection

Multiple authors (ER, SA, AJ, NS) contributed to the screening and data extraction procedures, with titles and abstracts of all deduplicated search results screened independently by at least two reviewers. The full text of potentially eligible papers was reviewed against our inclusion and exclusion criteria independently by two reviewers. Disagreements were resolved by discussion, with a third reviewer available as an arbitrator if necessary. We used the online software Rayyan (https://rayyan.ai/) to manage the study selection process.

Data extraction and quality assessment

Two reviewers used a piloted form (Google Form) developed for the review to independently extract data regarding review characteristics, characteristics of included primary studies, and outcome data. If clarifications were needed, we contacted the corresponding authors.

The quality of included systematic reviews was assessed by two reviewers (ER and SA, with discrepancies resolved by agreement or a third independent assessor, AJ) using the AMSTAR2 tool, which classifies the overall confidence in the results of each review as critically low, low, moderate, or high.[24]

Data synthesis

The following steps were followed to synthesise the evidence: First, all included systematic reviews were described in a summary table. Second, the results (primary and secondary outcomes) for each combination of conditions were summarised, including the pooled estimates, the number of studies, pooled sample size, a measure of heterogeneity, range of pooled effect sizes, and quality assessment. Third, the results were stratified by age, gender, socio-economic group, type of TB and region, where possible. We had initially planned to extract and pool individual study data for LMICs when such studies had been pooled together with data from HICs, or when individual study data were reported but not pooled in a meta-analysis. However, such an approach was deemed unfeasible due to the high number of reviews where this would have been required. In these cases, we reported the study characteristics and the range of study effect sizes from LMICs.

Patient and Public Involvement

We asked patients' representatives for feedback on the study protocol and they will be involved in the dissemination of our results. Patients or the public were not involved in the conduct or reporting of our research.

For peer review only - http://bmjopen.bmj.com/site/about/guidelines.xhtml

The funder of the study had no role in study design, data collection, data analysis, data interpretation, or writing of the report.

RESULTS

Our search strategy identified 7,557 results, of which 2,200 were duplicates and were removed. Of the 221 results remaining after screening titles and abstracts, 130 were excluded for not meeting eligibility criteria. Supplementary Table 2 (Appendix 1) specifies the reasons for exclusion. The full text corresponding to 34 protocols or conference abstracts could not be obtained. We contacted the authors of these references (with a follow-up email two weeks later), seven of which replied confirming that no full article had been published. Three journal articles, related to coronary heart disease, head and neck TB, and HBV, could not be assessed in full text despite our efforts (no institutional access and no response from authors[25–27]). The full text of one additional study[25] could not be obtained, but the pooled relative risk of coronary heart disease was reported in the abstract and was therefore included. Ultimately, 54 studies were included in our review (Figure 1).

Study and participant characteristics

Overall, there were over 6,296,000 people with TB across the 54 included systematic reviews, covering 85 LMICs (Appendix 3). Of these, 23 reported a pooled estimate of interest to our review,[S1–S23] while the remainder reported outcomes of interest for individual studies, but either did not pool them in a meta-analysis or pooled them with data from HICs. Among the 23 reviews reporting pooled outcomes, even when they assessed the same combination of TB and chronic condition(s), there was limited overlap between them with regards to geographical region and/or reported outcomes (Supplementary Table 3 in Appendix 1). Supplementary Table 4 (Appendix 1) details outcome information reported by each review.

Most of the included systematic reviews reported data on TB without specifying a particular type of TB;[S1–S4,S7–S10,S14,S16,S17,S20–S46] nine focused on drug-resistant TB (DR-TB),[S6] multidrug-resistant TB (MDR-TB),[S5,S12,S19,S47–S50] or extensively drug-resistant TB (XDR-TB);[S12,S47] three focused on pulmonary TB (PTB),[S15,S18,S51] three on TB meningitis,[S13,S52,S53] and one on TB lymphadenitis.[S11] The chronic conditions most often considered were HIV (31 reviews),[S1,S2,S5–S13,S24–S36,S47–S50,S52–S54] DM (14 reviews),[S4,S14–S18,S37–S43,S51] and mental illness (5 reviews).[S3,S19–S21,S44] None of the systematic reviews reported results on the prevalence and/or associated risks of more than one additional chronic condition in people with TB. Supplementary Table 5 (Appendix 1) lists what conditions were considered or not a chronic condition for this review.

Most of the identified systematic reviews were assessed as low or critically low quality according to AMSTAR2 (n=42). Only seven reviews were assessed as moderate (n=2)[S7,S22] or high (n=5)[S6,S10,S11,S20,S42] quality, six of which reported a pooled estimate of interest. The critical domains that failed most often were regarding risk of bias assessment (37 studies) and protocol registration (29 studies). Supplementary Table 6 (Appendix 1) details the AMSTAR2 assessment for each study.

For peer review only - http://bmjopen.bmj.com/site/about/guidelines.xhtml

Summary of results

TB and HIV

Of the 31 reviews reporting data on TB and HIV (>3,017,000 participants from 72 countries),[S1,S2,S5–S13,S24–S36,S47–S50,S52–S54] 11 focused on specific types of TB,[S5,S6,S11–S13,S47–S50,S52,S53] and 11 reported at least one pooled outcome of interest (Supplementary Table 3 in Appendix 1).[S1,S2,S5–S13] One review[S9] reported the pooled prevalence for Latin America (25%, 95%CI 19.3%-30.8%, 7 studies, critically low quality) and Africa (31.2%, 95%CI 19.3%-43.2%, 17 studies, critically low quality). Prevalence estimates for sub-continental regions were also reported in other reviews, ranging from 25% in Western Sub-Saharan Africa (SSA, high quality) to 44% in Southern SSA (high quality), as well as for China, Ethiopia, and Iran (Table 1).

One review[S5] reported a reduced odds of treatment success (OR 0.87, 95%CI 0.79-0.96, 6 studies, critically low quality) in people with TB and HIV compared to people with only TB, in SSA.

Table 1 also summarises the results of systematic reviews reporting data for specific types of TB (DR-TB, and MDR-TB, PTB, TB meningitis, and TB lymphadenitis).

For peer review only

Table 1: Prevalence of HIV and effect on outcomes in people with tuberculosis

Region	Outcome (quality[¶])	Effect size (95% CI)	Range	Number of studies (number of participants)	I^2	Year
Latin America	Prevalence (Critically low)[S9]	25% (19.3% - 30.8%)	NR	7 (NR)	95.2%	2013
Africa	Prevalence(Critically low)[S9]	31.2% (19.3% - 43.2%)	NR	17 (NR)	99.6%	2013
SSA	Prevalence (High)[S10]	31.81% (27.83% -36.07%)	6.03%-72.25%	68 (62,696)	98%	2019
	Successful treatment (Critically low)[S5]	OR 0.87 (0.79 - 0.96)	0.75-1.26	6 (NR)	NR	2019
Western SSA	Prevalence (High)[S10]	25.48% (19.70%-32.27%)	10.26%-72.13%	21 (16,145)	98%	2019
Eastern SSA	Prevalence (High)[S10]	31.14% (25.39%-37.54%)	6.03%-60.51%	32 (33,637)	98%	2019
Southern SSA	Prevalence (High)[S10]	43.67% (35.05%-52.69%)	23.84%-72.25%	12 (11,148)	99%	2019
Central SSA	Prevalence (High)[S10]	41.33% (30.39%-53.19%)	31.29%-51.56%	3 (2,039)	96%	2019
China	Prevalence(Critically low)[S8]	0.9% (0.6%–1.4%)	0.1%-4.5%	18 (NR)	92.21%	2010
	Prevalence in Women (Critically low)[S8]	0.6% (0.3%-1.1%)	NR	9 (NR)	71.8%	2010
	Prevalence in Men (Critically low)[S8]	1.1% (0.6%-2.0%)	NR	9 (NR)	94.7%	2010
Ethiopia	Prevalence (Moderate)[S7]	23.40% (19.56%-27.24%)	9.50%-52.10%	13 (19,212)	97.6%	2019
	Successful treatment (Critically low)[S2]	67% (56%-79%)	NR	NR (NR)	NR	2018
	Unsuccessful treatment (Critically low)[S2]	33% (21%-44%)	NR	NR (NR)	NR	2018
	Unsuccessful treatment (Critically low)[S2]	OR 1.98 (1.56-2.52)	0.82-14.31	20 (NR)	81.0%	2018
India	Case-fatality rate during treatment (Critically low)[S1]	10.91% (7.68%-15.50%)[*]	NR	35 (NR)	Tau2=0.90[†]	2020
	Case-fatality rate after treatment (Critically low)[S1]	4.15% (1.06% to 16.24%)	NR	5 (NR)	Tau2=1.90[†]	2020
Iran	Prevalence (Critically low) [S28]	14% (12% - 15%)	0%-54%	48 (21,388)	97.93%	2019

Drug resistant TB + HIV						
SSA	Unsuccessful treatment (High)[S6]	RR 1.18 (1.07-1.30)‡,§	0.71-2.37	19 (8,301)	48%	2020
	Treatment failure (High)[S6]	RR 0.66 (0.38-1.13) ‡	0.15-2.40	10 (5,474)	73%	2020
	Loss to follow-up (High)[S6]	RR 0.82 (0.74-0.92) ‡	0.49-2.61	14 (7,051)	0%	2020
	Mortality (High)[S6]	RR 1.50 (1.30-1.74) ‡	0.73-2.18	16 (7,365)	39%	2020
Western SSA	Unsuccessful treatment (High)[S6]	RR 1.42 (0.95-2.13)	1.31-2.37	2 (790)	12%	2020
	Mortality (High)[S6]	RR 1.42 (0.96-2.09)	NA	1 (588)	NA	2020
Eastern SSA	Unsuccessful treatment (High)[S6]	RR 1.47 (1.23-1.75)	1.14-1.77	6 (1,970)	0%	2020
	Mortality (High)[S6]	RR 1.52 (1.19–1.93)	1.20-2.18	5 (1,442)	0%	2020
Southern SSA	Unsuccessful treatment (High)[S6]	RR 1.09 (0.98-1.20) ‡	0.71-1.41	11 (5,541)	43%	2020
	Mortality (High)[S6]	RR 1.49 (1.21-1.83) ‡	0.73-1.47	10 (5,335)	60%	2020
Multidrug resistant TB + HIV						
LMIC	Unsuccessful treatment (Low)[S12]	RR 1.34 (1.04-1.72)	0.55-3.33	13 (5816)	88%	2018
Low income countries	Unsuccessful treatment (Low)[S12]	RR 2.23 (1.60 - 3.11)	0.67-3.33	7 (NR)	41%	2018
	Treatment failure (Low)[S12]	RR 0.75 (0.44-1.29)	0.32-2.40	7 (5,930)	55%	2018
SSA	Successful treatment (Critically low)[S5]	OR 0.87 (0.79 - 0.96)	0.75 - 1.26	6 (NR)	NR	2019
	Mortality (Critically low)[S5]	18% (14%-23%)	9%-31%	9 (NR)	91.1%	2019
	Cured (Critically low)[S5]	34% (22%-45%)	3%-60%	9 (NR)	98.9%	2019
Pulmonary TB + HIV						
Ethiopia	Prevalence (Moderate)[S7]	22.08% (14.36%-29.81%)	4.97%-28.60%	3 (1079)	89.9%	2019
TB meningitis + HIV						
LMIC	Prevalence (Critically low)[S13]	10.6% (4.2%–24.6%)	NR	NR (NR)	NR	2019
	Mortality (Critically low)[S13]	53.4% (42.4%–64.1%)	NR	7 (547)	2.1%	2019
TB lymphadenitis + HIV						
Africa	Prevalence (High)[S11]	52% (33%-71%)	6%-91%	14 (NR)	99.2%	2019
Ethiopia	Prevalence (High)[S11]	21% (12%-30%)	6%-67%	6 (NR)	92.9%	2019

Notes: SSA: Sub-Saharan Africa; LMIC: Low and middle-income countries; NA: Not applicable; RR: Risk ratio; NR: Not reported; OR: Odds ratio.
* Including only 7 high quality studies: 12.17% (95% CI 5.68%-26.11%)
† In the original review they considered this value low if it was <4
‡ Includes one study focused on children
§ Excluding one study focused on children: RR 1.12 (0.96-1.30, I2=69%)
¶ Quality rating represents the overall confidence in the results of the review, as assessed with the AMSTAR2 tool

TB and DM

Of the 14 reviews reporting data on TB and DM (>2,878,000 participants from 48 countries),[S4,S13–S17,S36–S42,S50] three focused on specific types of TB,[S14,S17,S50] and six reported at least one pooled outcome of interest (Supplementary Table 3 in Appendix 1).[S4,S14–S18]

For peer review only - http://bmjopen.bmj.com/site/about/guidelines.xhtml

One review[S17] reported the pooled prevalence separately for low-income countries (7.9%, 95%CI 4.9%-11.5%, 15 studies, 9,434 participants, critically low quality), lower-middle income countries (17.7%, 95%CI 15.1%-20.5%, 48 studies, 48,036 participants, critically low quality), and upper-middle income countries (14.4%, 95%CI 12.8%-16.0%, 75 studies, 1,994,027 participants, critically low quality). The same review also reported the prevalence of DM in people with TB in Africa (8.0%, 95%CI, 5.9%-10.4%, 119 studies, 474,944 participants, critically low quality, Table 2). Pooled prevalences in other continents were also reported, but were excluded from our review, as they included data from HICs. Other reviews reported prevalence estimates for sub-continental regions, ranging from 9% in SSA (low quality)[S14] to 21% in South Asia (low quality),[S4] as well as for multiple individual countries (Figure 2, Table 2).

One review[S16] reported an increased odds of mortality (OR 1.80, 95%CI 1.35-2.40, 34 studies, low quality) and treatment failure or death (OR 1.90, 95%CI 1.43-2.53, 22 studies, low quality) in people with TB and DM compared to people with only TB, in LMICs overall.

Table 2 also summarises the results of systematic reviews focused on (or reporting data for) specific types of TB (MDR-TB and PTB).

Table 2: Prevalence of diabetes mellitus and effect on outcomes in people with tuberculosis

Region	Outcome (quality*)	Effect size (95% CI)	Range	Number of studies (number of participants)	I^2	Year
LMIC	Mortality (Low)[S16]	OR 1.80 (1.35-2.40)	0.45-29.22	34 (NR)	91.1%	2019
	Treatment failure or death (Low)[S16]	OR 1.90 (1.43-2.53)	0.73-11.75	22 (NR)	87.3%	2019
World (Low-income countries)	Prevalence (Critically low)[S17]	7.9% (4.9%-11.5%)	NR	15 (9,434)	96.8%	2019
World (Lower-Middle income countries)	Prevalence (Critically low)[S17]	17.7% (15.1%-20.5%)	NR	48 (48,036)	98.3%	2019
World (Upper-Middle income countries)	Prevalence (Critically low)[S17]	14.4% (12.8%-16.0%)	NR	75 (1,994,027)	99.9%	2019
Africa	Prevalence (Critically low)[S17]	8.0% (5.9%-10.4%)	1.9%-32.4%	119 (474,944)	99.8%	2019
Southeast Asia	Prevalence (Critically low)[S17]	19.0% (16.2%-21.9%)	5.1%-54.1%	30 (30,382)	97.0%	2019
South Asia	Prevalence (Low)[S4]	21% (18%-23%)	4%-66%	65 (NR)	98.28%	2021
SSA	Prevalence (Low)[S14]	9% (6%-12%)	2%-38%	16 (13,286)	97.48%	2019
Bangladesh	Prevalence (Critically low)[S17]	10.6% (7.2%-14.5%)	8.3%-12.8%	3 (3,010)	85.9%	2019
Benin	Prevalence (Critically low)[S17]	1.9% (0.2%-4.7%)	NA	1 (159)	NA	2019
Brazil	Prevalence (Critically low)[S17]	7.2% (6.3%-8.1%)	3.3%-33.1%	12 (1,726,436)	99.7%	2019
China	Prevalence (Critically low)[S17]	14.5% (10.5%-19.0%	2.7%-30.1%	14 (19,529)	98.4%	2019
Egypt	Prevalence (Critically low)[S17]	22.8% (15.2%-31.4%)	15.8%-27.7%	3 (578)	81.4%	2019
Ethiopia	Prevalence (Critically low)[S17]	18.8% (1.9%-47.1%)	8.3%-32.4%	2 (1,749)	99.2%	2019
Fiji	Prevalence (Critically low)[S17]	10.1% (4.4%-17.7%)	5.2-13.7	3 (1,139)	91.8%	2019
Georgia	Prevalence (Critically low)[S17]	12.4% (7.4%-18.5%)	NA	1 (137)	NA	2019
Guinea-Bissau	Prevalence (Critically low)[S17]	2.7% (0.3%-6.8%)	NA	1 (110)	NA	2019
Guyana	Prevalence (Critically low)[S17]	14.0% (7.8%-21.6%)	NA	1 (100)	NA	2019

For peer review only - http://bmjopen.bmj.com/site/about/guidelines.xhtml

India	Prevalence (Low)[S4]	22.0% (19.0%–25.0%)	NR	47 (NR)	97.92%	2021
	Mortality (Low)[S4]	OR 1.74 (1.21-2.51)	NR	5 (NR)	19.43%	2021
	Treatment failure (Low)[S4]	OR 1.65 (1.12-2.44)	NR	5 (NR)	49,63%	2021
	Recurrence (Low)[S4]	OR 0.53 (0.32-0.87)	NA	1 (NR)	NA	2021
	Cured (Low)[S4]	OR 0.32 (0.10-1.05)	NA	1 (NR)	NA	2021
Indonesia	Prevalence (Critically low)[S17]	14.8% (12.2%-17.7%)	NA	1 (634)	NA	2019
Iran	Prevalence (Critically low)[S17]	17.8% (12.5%-23.8%)	5.5%-40.0%	11 (3,134)	93.3%	2019
Kazakhstan	Prevalence (Critically low)[S17]	7.1% (5.1%-9.4%)	NA	1 (562)	NA	2019
Kiribati	Prevalence (Critically low)[S17]	36.7% (31.1%-42.5%)	NA	1 (275)	NA	2019
Libya	Prevalence (Critically low)[S17]	6.1% (3.5%-9.4%)	NA	1 (262)	NA	2019
Malaysia	Prevalence (Critically low)[S17]	26.9% (17.8%-37.0%)	15.4%-39.0%	5 (23,438)	98.1%	2019
Marshall Islands	Prevalence (Critically low)[S17]	45.2% (32.9%-57.7%)	NA	1 (62)	NA	2019
Mexico	Prevalence (Critically low)[S17]	30.8% (26.4%-35.3%)	19.3%-54.4%	10 (192,420)	97.9%	2019
Nepal	Prevalence (Low)[S4]	12.0% (4.0%–20.0%)	NR	4 (NR)	97.6%	2021
Nigeria	Prevalence (Critically low)[S17]	7.8% (4.4%-12.0%)	4.8%-12.0%	4 (9,821)	97.8%	2019
Pakistan	Prevalence (Low)[S4]	19.0% (11.0%–27.0%)	NR	10 (NR)	99.18%	2021
Peru	Prevalence (Critically low)[S17]	4.8% (1.7%-9.5%	2.5%-11.1%	4 (3,983)	96.8%	2019
Romania	Prevalence (Critically low)[S17]	18.4% (13.6%-23.7%)	NA	1 (228)	NA	2019
Senegal	Prevalence (Critically low)[S17]	4.9% (2.2%-8.5%)	3.8%-7.0%	2 (2,848)	75.1%	2019
South Africa	Prevalence (Critically low)[S17]	9.4% (7.6%-11.3%)	NA	1 (947)	NA	2019
Sri Lanka	Prevalence (Low)[S4]	24.0% (21.0%–27.0%)	NR	2 (NR)	NR	2021
Tanzania	Prevalence (Critically low)[S17]	8.5% (4.8%-13.0%)	2.6%-16.7%	7 (4,178)	95.1%	2019
Thailand	Prevalence (Critically low)[S17]	7.5% (6.2%-8.8%)	6.0%-16.3%	5 (17,862)	81.6%	2019
Tunisia	Prevalence (Critically low)[S17]	7.6% (5.9%-9.6%)	NA	1 (788)	NA	2019
Turkey	Prevalence (Critically low)[S17]	7.8% (6.8%-8.8%)	7.9%-8.6%	3 (2,773)	0%	2019
Uganda	Prevalence (Critically low)[S17]	7.3% (4.7%-10.3%)	5.4%-8.5%	2 (390)	9.9%	2019
Yemen	Prevalence (Critically low)[S17]	9.5% (6.0%-13.8%)	NA	1 (220)	NA	2019
Multidrug resistant TB + DM						
LMIC	Unsuccessful treatment (Low)[S12]	RR 0.90 (0.65-1.23)	0.23-0.98	3 (687)	19%	2018
Pulmonary TB + DM						
China	Prevalence (Critically low)[S15]	7.20% (6.01%-8.39%	2.08%-16.16%	22 (56,805)	NR	2013
	Retreatment (Critically low)[S18]	OR 2.05 (1.30-3.22)	NR	3 (499)	0%	2016
	Retreatment (Critically low)[S18]	aOR 3.38 (1.56-7.29)	NR	2 (NR)	75%	2016

Notes: LMIC: Low and middle-income countries; OR: Odds ratio; aOR: adjusted odds ratio; NR: Not reported; NA: Not applicable

* Quality rating represents the overall confidence in the results of the review, as assessed with the AMSTAR2 tool

TB and mental disorders

For peer review only - http://bmjopen.bmj.com/site/about/guidelines.xhtml

TB and mental disorders (pooled as a composite outcome)

We found one systematic review considering a composite outcome for mental disorders,[S21] as well as several other reviews looking at individual mental disorders such as depression, anxiety, and psychosis. The review[S21] that reported the effect of mental disorders (defined as a composite variable including depression, psychological distress, PTSD, or mental disorder) on unsuccessful treatment (a composite measure combining some or all of treatment failure, loss to follow-up, and death), loss to follow-up, and non-adherence, found no evidence of a significant increase in the odds of these outcomes in people with TB and mental disorders, compared to people with only TB (Table 3).

TB and depression

Of the four reviews reporting data on TB and depression (>21,770 participants from 33 countries),[S3,S19,S20,S44] three[S3,S20,S44] reported at least one pooled outcome of interest (Supplementary Table 3 in Appendix 1). One systematic review[S20] of 25 studies reported the prevalence of depression in people with TB in LMICs as 45.19% (95%CI 38.04%-52.55%, 25 studies, 4,903 participants, high quality). None of the included reviews reported this outcome at a continental, regional or country level (Table 3). One systematic review[S3] reported an increased odds of mortality (OR 2.85, 95%CI 1.52-5.36, 2 studies, 1,303 participants, critically low quality) and other adverse outcomes in people with TB and depression compared to people with only TB (Table 3).Table 3 summarises the results of systematic reviews focused on MDR-TB. According to these results, the prevalence of depression in people with MDR-TB is 52% (95%CI 38%-66%, 5 studies, high quality).[S20]

TB and anxiety

Of the two[S19,S44] reviews reporting data on TB and anxiety (>7,500 participants from 31 countries), only one,[S19] focused on MDR-TB, reported any pooled outcome of interest: the prevalence of anxiety overall (24%, 95%CI 2%-57%, 3 studies, 209 participants, critically low quality) and in the regions of Southeast Asia and the Americas (Table 3).

TB and psychosis

One systematic review (7518 participants from 17 countries), focused on MDR-TB, reported the prevalence of psychosis in Africa (12%, 95%CI 8%-17%, 5 studies, critically low quality) and in several subcontinental regions (Table 3).[S19]

Table 3: Prevalence and effect on outcomes in people with tuberculosis (TB) and mental disorders: as a composite outcome and separately for depression, anxiety, and psychosis.

Region	Outcome (quality‡)	Effect size (95% CI)	Range	Number of studies (number of participants)	I^2	Year
TB + Mental disorders (composite measure)*						
LMIC	Unsuccessful treatment† (Critically low)[S21]	OR 2.13 (0.85-5.37)	0.80-4.25	4 (1,196)	82%	2020
	Loss to follow-up (Critically low)[S21]	OR 1.90 (0.33-10.91)	0.88-5.33	2 (1,139)	78%	2020

For peer review only - http://bmjopen.bmj.com/site/about/guidelines.xhtml

	Non-adherence to treatment (Critically low)[S21]	OR 1.60 (0.84-3.02)	0.94-3.67	4 (10,851)	86%	2020
TB + Depression						
LMIC	Prevalence (High)[S20]	45.19% (38.04%-52.55%)	15.56%-80.00%)	25 (4,903)	96.28%	2020
	Prevalence in Women (High)[S20]	51.54% (40.34%–62.60%)	NR	17 (NR)	92.55%	2020
	Prevalence in Men (High)[S20]	45.25% (35.19%–55.71%)	NR	17 (NR)	95.09%	2020
	Mortality or loss to follow-up (Critically low)[S3]	OR 4.26 (2.33–7.79)	3.65-4.88	2 (1303)	0%	2020
	Mortality (Critically low)[S3]	OR 2.85 (1.52-5.36)	1.76-2.99	2 (973)	0%	2020
	Loss to follow up (Critically low)[S3]	OR 8.70 (4.95-9.09)	4.95-9.09	2 (973)	0%	2020
	Non-adherence to TB treatment (Critically low)[S3]	OR 1.38 (0.70-2.72)	0.92-3.67	3 (9,349)	94.36%	2020
Multidrug resistant TB + Depression						
LMIC	Prevalence (High)[S20]	52.34% (38.09%-66.22%)	NR	5 (NR)	92.55%	2020
Africa	Prevalence (Critically low)[S19]	16% (9%-24%)	NA	3 (NR)	NA	2018
The Americas Region	Prevalence (Critically low)[S19]	36% (23-50%)	NR	3 (NR)	NR	2018
European Region	Prevalence (Critically low)[S19]	11% (4%-21%)	NR	3 (NR)	NR	2018
Eastern Mediterranean Region	Prevalence (Critically low)[S19]	73% (64%-81%)	NR	2 (NR)	NR	2018
Western Pacific Region	Prevalence (Critically low)[S19]	5% (1%-12%)	NA	1 (NR)	NA	2018
Southeast Asia	Prevalence (Critically low)[S19]	22% (0%-60%)	NA	3 (NR)	NA	2018
Multidrug resistant TB + Anxiety						
LMIC	Prevalence (Critically low)[S19]	24% (2%-57%)	12%-56%	3 (209)	95%	2018
The Americas Region	Prevalence (Critically low)[S19]	14% (9%-21%)	NR	2 (NR)	NR	2018
Southeast Asia	Prevalence (Critically low)[S19]	56% (45%-66%)	NR	1 (NR)	NR	2018
Multidrug resistant TB + Psychosis						
Africa	Prevalence (Critically low)[S19]	12% (8%-17%)	NR	5 (NR)	NR	2018
The Americas Region	Prevalence (Critically low)[S19]	11% (7%-17%)	NR	2 (NR)	NR	2018
European Region	Prevalence (Critically low)[S19]	6% (0%-17%)	NR	2 (NR)	NR	2018
Eastern Mediterranean Region	Prevalence (Critically low)[S19]	7% (1%-17%)	NA	1 (NR)	NA	2018
Southeast Asia	Prevalence (Critically low)[S19]	10% (5%-17%)	NA	2 (NR)	NA	2018

Notes: LMIC: Low and middle-income countries; OR: Odds ratio; NR: Not reported; NA: Not applicable.
* Includes depression, psychological distress, PTSD, or mental disorder as a composite variable.
† Composite measure combining some or all of: treatment failure, loss to follow-up, and death.
‡ Quality rating represents the overall confidence in the results of the review, as assessed with the AMSTAR2 tool

TB and HCV

One systematic review estimated the prevalence of HCV in people with TB in Africa to be 11% (95%CI 1%-23%, 3 studies, 327 participants, I^2=93.9%, moderate quality).[S22]

Risk of cancer in people with TB

For peer review only - http://bmjopen.bmj.com/site/about/guidelines.xhtml

One systematic review[S23] reported the risk of different types of cancer in people with TB in upper-middle income countries, including lung cancer (RR 1.53, 95%CI 1.25-1.87, 9 studies, low quality), non-Hodgkin's lymphoma (RR 1.70, 95%CI 1.13-2.56, 1 study, low quality) and leukaemia (RR 1.61, 95%CI 1.13-2.29, 1 study, low quality)(Table 4).

Risk of coronary heart disease in people with TB

One systematic review reported (in their abstract) an increased risk of coronary heart disease in people with TB in LMICs (RR 1.76, 95%CI 1.05–2.95)(Table 4).[S55]

Table 4 : Risk of cancer and coronary heart disease in people with tuberculosis

Region	Outcome (quality*)	Effect size (95% CI)	Range	Number of studies (number of participants)	I^2	Year
Upper-Middle income countries	Lung cancer (Low)[S23]	RR 1.53 (1.25-1.87)	NR	9 (NR)	94.6%	2020
	non-Hodgkin's lymphoma (Low)[S23]	RR 1.70 (1.13-2.56)	NA	1 (NR)	NA	2020
	Leukaemia (Low)[S23]	RR 1.61 (1.13-2.29)	NA	1 (NR)	NA	2020
LMIC	Coronary heart disease † [S55]	RR 1.76 (1.05–2.95)	NA	4 (NR)	97%	2020

Notes: LMIC: Low and middle-income countries; RR: Risk ratio; NR: Not reported; NA: Not applicable.
* Quality rating represents the overall confidence in the results of the review, as assessed with the AMSTAR2 tool
† Data extracted from abstract only, as we could not obtain the full text article.

Subgroup analyses

Regarding our planned subgroup analyses, we could only find data stratified by gender reported for the prevalence of HIV in people with TB in China (Women: 0.6%, 95%CI 0.3%-1.1%, 9 studies, critically low quality; Men: 1.1% (95%CI 0.6%-2.0%; 9 studies, critically low quality)(Table 1)[S8] and the prevalence of depression in people with TB (Women: 51.54%, 95%CI 40.34%-62.60%, 17 studies, high quality; Men: 45.25%, 95%CI 35.19%–55.71%, 17 studies, high quality)(Table 3).[S20] We did not find any pooled results stratified by age.

DISCUSSION

T his was the first meta-review to identify and map out the co-occurrence of CCDs and NCDs in people with TB in LMICs. Although the geographical regions covered by the included reviews varied, we found that the most prevalent chronic conditions were depression, HIV, and DM. We also found some evidence that people with TB and these chronic conditions had significantly increased odds of adverse outcomes such as death and treatment failure. No systematic review pooled the prevalence of two or more additional chronic conditions in people with TB and differences between people with TB and a single chronic condition vs multiple additional chronic conditions could not be explored.

While HIV, DM and depression are well-known comorbidities of TB, our review highlights that their prevalence can vary, in some cases substantially, between different countries or regions. Such regional differences should

For peer review only - http://bmjopen.bmj.com/site/about/guidelines.xhtml

be taken into account when designing interventions, illustrating how a one-size-fits-all approach is unlikely to succeed.

Our findings offer an overview of TB multimorbidity to see comorbid conditions in relation to each other. For instance, despite the known synergistic relationship between TB and HIV,[14] our review suggests that the negative impact of HIV on TB treatment outcomes is less severe than the impact of depression, which not only had higher odds of adverse outcomes, but also was more prevalent among people with TB. This apparent smaller impact of HIV than depression in people with TB could partially be explained by the disparity – in attention and resources – between HIV and depression, and illustrates how an integrated approach, such as the one received by at least some patients with TB and HIV, could reduce the negative impact of other chronic conditions, such as depression, in TB patients. This also illustrates how the results of our review could be used when planning for new services. Moreover, it highlights the importance of screening for mental health in areas where mental health services need improvement.[28,29]

Our meta-review highlights the many gaps in the literature on TB multimorbidity in LMIC. For example, while the meta-analysis of the prevalence of TB and depression included 25 studies,[30] the meta-analysis for treatment outcomes in this group included only two studies,[18] reflecting the lack of evidence for the impact of TB multimorbidity on TB treatment outcomes. Data stratified by gender or age was also minimal, which is particularly important when women might have different healthcare seeking behaviours and limited voice in decision making. In addition to the gaps in the literature with regards to primary studies, our meta-review also highlights the lack of systematic reviews focused on people with TB and more than one additional chronic condition, which is an increasingly likely scenario as the prevalence of NCDs in LMICs grows.[2] In this regard, several cohort studies have assessed the impact of multimorbidity on TB treatment outcomes, such as in China or Brazil [31,32] finding worse outcomes among patients with multiple additional chronic conditions. Furthermore, Chen et al [31] results' highlight that some combinations of comorbidities, such as the group with cardiovascular morbidity with complications, increase the risk of negative TB treatment outcomes more than others. Considering the potential multiple-way synergies between multiple chronic conditions, a systematic review of the literature on this topic is sorely needed. This evidence gap is addressed in a complementary review by our group.[33]

We did not find any systematic reviews focusing on CCDs and NCDs in people with zoonotic TB (zTB). While this type of TB was estimated to represent 1.4% of all TB cases in 2019, this number is likely to be an underestimate, as there are poor surveillance programmes, under-reporting and lack of laboratory confirmation of the causative agent [34]. It is therefore not surprising that we could not find any systematic reviews synthesising studies reporting on the prevalence of comorbidities specifically in zTB.

In addition to the gaps in the literature, our meta-review also highlights the need for systematic reviews of higher quality, as most of the identified systematic reviews were assessed as low or critically low quality according to AMSTAR2, limiting the certainty we can have in their results. The systematic reviews with high or moderate quality that we have identified reported prevalence of TB+HIV in SSA (and Ethiopia), the effect of HIV in people with DR-TB in SSA, the prevalence of HIV in people with TB lymphadenitis in Africa (and Ethiopia), and the prevalence of depression in people with TB and with MDR-TB in LMIC.

For peer review only - http://bmjopen.bmj.com/site/about/guidelines.xhtml

Strengths

Our review has several strengths, such as an extensive search strategy, including databases of grey literature and protocols. Considering that PROSPERO is the main registry for systematic reviews and our efforts to contact authors of potentially relevant protocols, we are confident in the coverage of our search strategy. Another strength of our review is our focus on LMICs, making sure that data from HICs was not included, as the differences in risk factors, resources and treatment opportunities would make the results less applicable to LMICs.

Limitations

Our review has several limitations as well. First, most of the meta-analyses had very high heterogeneity and should therefore be interpreted with caution. This was the case even in systematic reviews focused on a single country. While part of this heterogeneity could be explained by methodological differences between the included studies (e.g. differences in the definitions and measurement of comorbidities), it could also reflect variation, inside a country, in how TB treatment strategies are adapted to local needs, their cultural acceptance and funding limitations. As risk factors for specific CCDs and NCDs are also heterogeneous between regions (e.g. prevalence of HIV in the community, smoking habits, access to treatment, etc.), the pattern of TB comorbidities is also likely to vary both between and within countries. Second, more than half of the studies summarised in our results had low or critically low quality. Third, despite the large number of systematic reviews identified in our review, our focus on LMIC excluded many results reported in them. Finally, we found little evidence regarding the burden of TB multimorbidity, which was one of the goals of this review. This highlights gaps in the body of evidence of systematic reviews, suggesting new future lines of research.

Conclusion

Given the fact that multimorbidity is common in LMICs[35,36] and is associated with a wide range of adverse outcomes for the individual, family, and society, and poses challenges for healthcare systems, particularly in LMICs, our results are important.[37,38] TB multimorbidity appears to be common and to have additional burdensome impact, deserving urgent attention.[15,39] Research is needed to identify early at-risk populations and ultimately prevent the onset of TB multimorbidity and to develop effective treatments and clinical pathways to care for this heterogeneous and burdensome group of people.[15] The high prevalence of TB multimorbidity in LMICs is a triple challenge, as these regions already have the highest (and growing) number of people with multimorbidity generally, the highest levels of TB, and health and social care systems which are stretched/sparse and unable to deal with these complexities.[15] Thus, urgent research is needed to better address this clearly prevalent, burdensome, and important issue.

Acknowledgments

This review is part of the TB Multimorbidity Network project (https://www.impactsouthasia.com/tbmm/), which is funded by the Medical Research Council (MRC Grant reference MC_PC_MR/T037806/1).

Competing interests

All authors declare no competing interests

For peer review only - http://bmjopen.bmj.com/site/about/guidelines.xhtml

Ethics statement

Ethical approval was not needed as this was a systematic review of published literature.

Funding

This work was supported by Medical Research Council UK, grant number MC_PC_MR/T037806/1.

Contributions

NS, BS, KS, and HE conceptualised the study; AJ, ER, AE, YL, RH, HE, KS, BS, and NS contributed to the design of the study; AJ, ER and NS contributed to the titles and abstracts screening; AJ, ER, and SA contributed to the full text screening and data extraction, ER and SA contributed to the quality assessment; AJ summarised the results and wrote the first draft of the report with input from BS and NS; ER, SA, AE, YL, and KS contributed to the interpretation of the results and revised the manuscript. All authors had full access to all the data in the study and had final responsibility for the decision to submit for publication.

Data sharing

All data relevant to the study are included in the article or uploaded as supplementary information.

Figure legends

Figure 1: Flow diagram of the search results and screening process.

Figure 2: Prevalence of diabetes mellitus in people with TB in each country

For peer review only

For peer review only - http://bmjopen.bmj.com/site/about/guidelines.xhtml

References

1 Foguet-Boreu Q, Violan C, Roso-Llorach A, *et al.* Impact of multimorbidity: acute morbidity, area of residency and use of health services across the life span in a region of south Europe. *BMC Fam Pract* 2014;**15**:55. doi:10.1186/1471-2296-15-55

2 France EF, Wyke S, Gunn JM, *et al.* Multimorbidity in primary care: a systematic review of prospective cohort studies. *Br J Gen Pract* 2012;**62**:e297–307. doi:10.3399/bjgp12X636146

3 Roche S, Vries E de. Multimorbidity in a large district hospital: A descriptive cross-sectional study. *S Afr Med J* 2017;**107**:1110–5.

4 GBD Compare | IHME Viz Hub. http://vizhub.healthdata.org/gbd-compare (accessed 8 Sep 2021).

5 World Health Organisation. HIV/AIDS. https://www.who.int/news-room/fact-sheets/detail/hiv-aids (accessed 21 Sep 2020).

6 Murray CJL, Vos T, Lozano R, *et al.* Disability-adjusted life years (DALYs) for 291 diseases and injuries in 21 regions, 1990–2010: a systematic analysis for the Global Burden of Disease Study 2010. *The Lancet* 2012;**380**:2197–223. doi:10.1016/S0140-6736(12)61689-4

7 Bates M, Marais BJ, Zumla A. Tuberculosis Comorbidity with Communicable and Noncommunicable Diseases. *Cold Spring Harb Perspect Med* 2015;**5**:a017889. doi:10.1101/cshperspect.a017889

8 Lim SS, Vos T, Flaxman AD, *et al.* A comparative risk assessment of burden of disease and injury attributable to 67 risk factors and risk factor clusters in 21 regions, 1990–2010: a systematic analysis for the Global Burden of Disease Study 2010. *The Lancet* 2012;**380**:2224–60. doi:10.1016/S0140-6736(12)61766-8

9 World Health Organization. *Global tuberculosis report 2020*. Geneva: : World Health Organization 2020. https://apps.who.int/iris/handle/10665/336069 (accessed 18 Jul 2022).

10 Roth GA, Abate D, Abate KH, *et al.* Global, regional, and national age-sex-specific mortality for 282 causes of death in 195 countries and territories, 1980–2017: a systematic analysis for the Global Burden of Disease Study 2017. *The Lancet* 2018;**392**:1736–88. doi:10.1016/S0140-6736(18)32203-7

11 Marais BJ, Lönnroth K, Lawn SD, *et al.* Tuberculosis comorbidity with communicable and non-communicable diseases: integrating health services and control efforts. *Lancet Infect Dis* 2013;**13**:436–48. doi:10.1016/S1473-3099(13)70015-X

12 Oh KH, Choi H, Kim EJ, *et al.* Depression and risk of tuberculosis: a nationwide population-based cohort study. *Int J Tuberc Lung Dis* 2017;**21**:804–9. doi:10.5588/ijtld.17.0038

13 Niazi AK, Kalra S. Diabetes and tuberculosis: a review of the role of optimal glycemic control. *J Diabetes Metab Disord* 2012;**11**:28. doi:10.1186/2251-6581-11-28

14 Kwan CK, Ernst JD. HIV and Tuberculosis: a Deadly Human Syndemic. *Clin Microbiol Rev* 2011;**24**:351–76. doi:10.1128/CMR.00042-10

15 Siddiqi K, Stubbs B, Lin Y, *et al.* TB multimorbidity: a global health challenge demanding urgent attention. *Int J Tuberc Lung Dis* 2021;**25**:87–90.

16 Huddart S, Svadzian A, Nafade V, *et al.* Tuberculosis case fatality in India: a systematic review and meta-analysis. *BMJ Glob Health* 2020;**5**:e002080. doi:10.1136/bmjgh-2019-002080

17 Eshetie S, Gizachew M, Alebel A, *et al.* Tuberculosis treatment outcomes in Ethiopia from 2003 to 2016, and impact of HIV co-infection and prior drug exposure: A systematic review and meta-analysis. *PloS One* 2018;**13**:e0194675. doi:10.1371/journal.pone.0194675

For peer review only - http://bmjopen.bmj.com/site/about/guidelines.xhtml

18 Ruiz-Grosso P, Cachay R, de la Flor A, *et al.* Association between tuberculosis and depression on negative outcomes of tuberculosis treatment: A systematic review and meta-analysis. *PloS One* 2020;**15**:e0227472.

19 Gautam S, Shrestha N, Mahato S, *et al.* Diabetes among tuberculosis patients and its impact on tuberculosis treatment in South Asia: a systematic review and meta-analysis. *Sci Rep* 2021;**11**:2113. doi:10.1038/s41598-021-81057-2

20 Moher D, Shamseer L, Clarke M, *et al.* Preferred reporting items for systematic review and meta-analysis protocols (PRISMA-P) 2015 statement. *Syst Rev* 2015;**4**:1.

21 Bernell S, Howard SW. Use Your Words Carefully: What Is a Chronic Disease? *Front Public Health* 2016;**4**. doi:10.3389/fpubh.2016.00159

22 Laserson KF, Thorpe LE, Leimane V, *et al.* Speaking the same language: treatment outcome definitions for multidrug-resistant tuberculosis. *Int J Tuberc Lung Dis* 2005;**9**:640–5.

23 Organization WH, others. Definitions and reporting framework for tuberculosis–2013 revision: updated December 2014 and January 2020. World Health Organization 2013.

24 Shea BJ, Reeves BC, Wells G, *et al.* AMSTAR 2: a critical appraisal tool for systematic reviews that include randomised or non-randomised studies of healthcare interventions, or both. *BMJ* 2017;**358**. doi:10.1136/bmj.j4008

25 Wongtrakul W, Charoenngam N, Ungprasert P. Tuberculosis and risk of coronary heart disease: A systematic review and meta-analysis. *Indian J Tuberc* 2020;**67**:182–8. doi:10.1016/j.ijtb.2020.01.008

26 Pang P., Sun C., Duan W., *et al.* A review on global head and neck tuberculosis cases from 980 papers and 5881 patients. *Int J Oral Maxillofac Surg* 2019;**48**:182. doi:10.1016/j.ijom.2019.03.563

27 Yixiang ZHENG, Shujuan MA, Deming TAN, *et al.* A meta-analysis of liver lesions in hepatitis B patients undergoing anti-tuberculosis therapy. *Chin J Hepatol* 2014;:585–9.

28 Keynejad RC, Dua T, Barbui C, *et al.* WHO Mental Health Gap Action Programme (mhGAP) Intervention Guide: a systematic review of evidence from low and middle-income countries. *Evid Based Ment Health* 2018;**21**:30–4.

29 Eaton J, McCay L, Semrau M, *et al.* Scale up of services for mental health in low-income and middle-income countries. *The Lancet* 2011;**378**:1592–603.

30 Duko B, Bedaso A, Ayano G. The prevalence of depression among patients with tuberculosis: a systematic review and meta-analysis. *Ann Gen Psychiatry* 2020;**19**:30. doi:10.1186/s12991-020-00281-8

31 Chen Q, Che Y, Xiao Y, *et al.* Impact of Multimorbidity Subgroups on the Health Care Use and Clinical Outcomes of Patients With Tuberculosis: A Population-Based Cohort Analysis. *Front Public Health* 2021;**9**:756717. doi:10.3389/fpubh.2021.756717

32 Soares LN, Spagnolo LM de L, Tomberg JO, *et al.* Relationship between multimorbidity and the outcome of the treatment for pulmonary tuberculosis. *Rev Gaúcha Enferm* 2020;**41**.

33 Alexander Jarde BS Ruimin Ma, Eugenia Romano, Helen Elsey, Najma Siddiqi, Kamran Siddiqi. Prevalence, clusters and burden of multimorbidity in people with tuberculosis: a systematic review and meta-analysis.

34 Luciano SA, Roess A. Human zoonotic tuberculosis and livestock exposure in low- and middle-income countries: A systematic review identifying challenges in laboratory diagnosis. *Zoonoses Public Health* 2020;**67**:97–111. doi:10.1111/zph.12684

For peer review only - http://bmjopen.bmj.com/site/about/guidelines.xhtml

35 Vancampfort D, Koyanagi A, Ward PB, *et al.* Perceived Stress and Its Relationship With Chronic Medical Conditions and Multimorbidity Among 229,293 Community-Dwelling Adults in 44 Low- and Middle-Income Countries. *Am J Epidemiol* 2017;**186**:979–89. doi:10.1093/aje/kwx159

36 Vancampfort D, Koyanagi A, Ward PB, *et al.* Chronic physical conditions, multimorbidity and physical activity across 46 low-and middle-income countries. *Int J Behav Nutr Phys Act* 2017;**14**:6.

37 Vancampfort D, Koyanagi A, Hallgren M, *et al.* The relationship between chronic physical conditions, multimorbidity and anxiety in the general population: A global perspective across 42 countries. *Gen Hosp Psychiatry* 2017;**45**:1–6. doi:10.1016/j.genhosppsych.2016.11.002

38 Jacob L, Oh H, Shin JI, *et al.* Informal Caregiving, Chronic Physical Conditions, and Physical Multimorbidity in 48 Low- and Middle-Income Countries. *J Gerontol A Biol Sci Med Sci* 2020;**75**:1572–8. doi:10.1093/gerona/glaa017

39 Stubbs B, Siddiqi K, Elsey H, *et al.* Tuberculosis and Non-Communicable Disease Multimorbidity: An Analysis of the World Health Survey in 48 Low- and Middle-Income Countries. *Int J Environ Res Public Health* 2021;**18**:2439. doi:10.3390/ijerph18052439

For peer review only

For peer review only - http://bmjopen.bmj.com/site/about/guidelines.xhtml

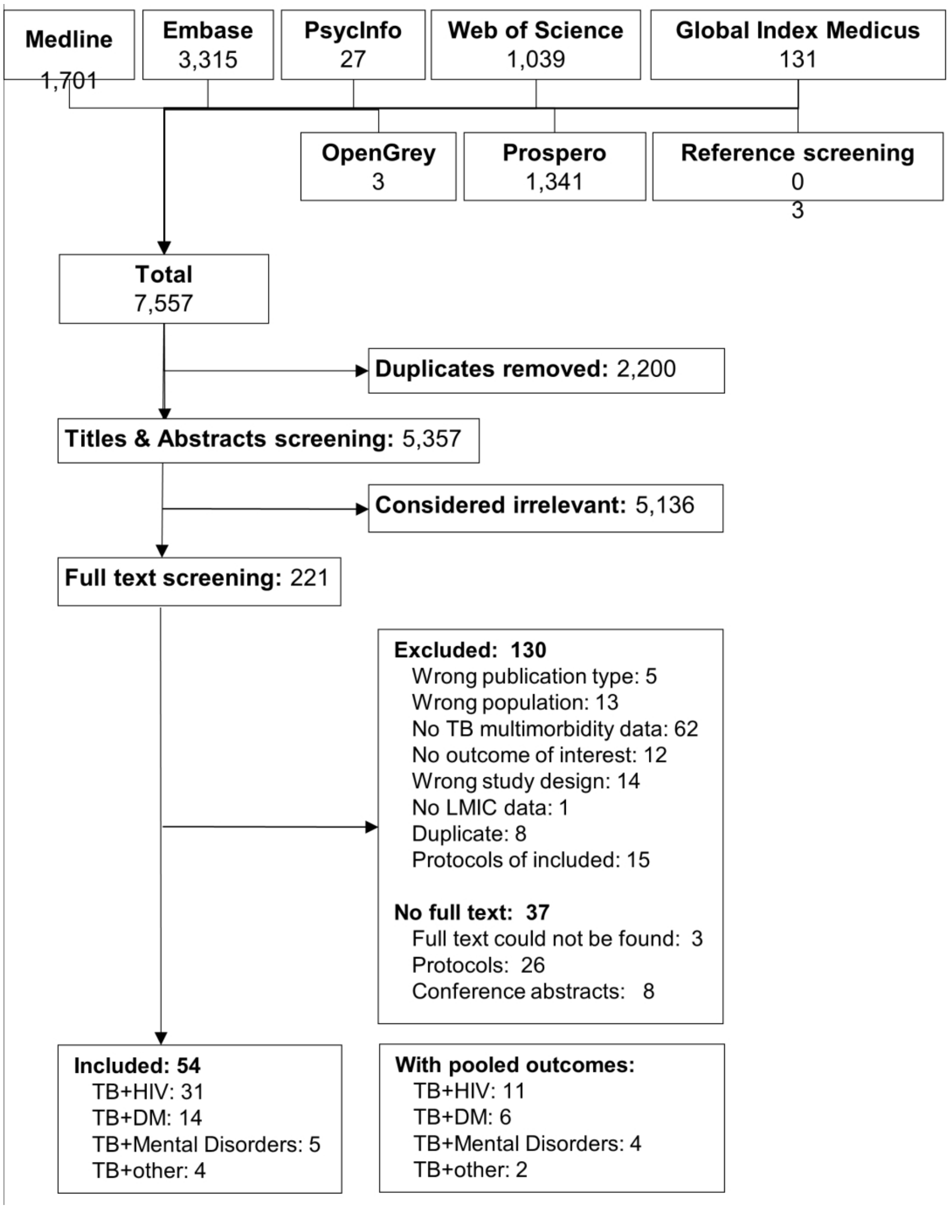

Flow diagram of the search results and screening process.

157x194mm (330 x 330 DPI)

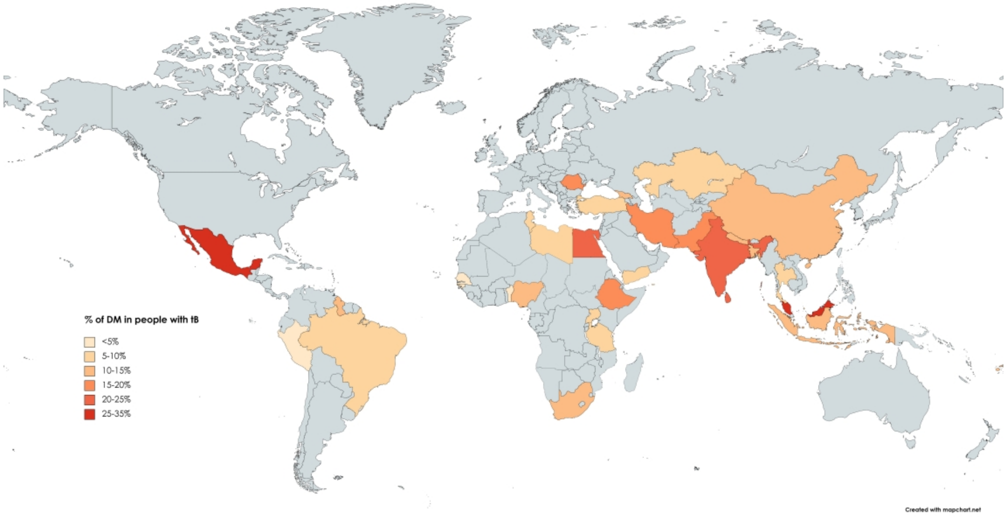

Prevalence of diabetes mellitus in people with TB in each country

546x288mm (300 x 300 DPI)

Supplemental material

Supplementary Table 1: Search terms used in Medline (run on October 23rd, 2020)

	Searches
1	exp Noncommunicable Diseases/ or ((Non-communicable or Noncommunicable or Non-infectious) adj (disease* or condition* or illness*)).mp.
2	exp Chronic Disease/ or ((chronic or long-term) adj (disease* or condition* or illness*)).mp.
3	exp Heart Diseases/ or (heart adj (disease* or disorder* or failure)).mp. or (cardiac adj (disease* or disorder* or failure)).mp.
4	exp Cardiovascular Diseases/ or (cardiovascular adj (disease* or disorder* or failure)).mp.
5	exp Coronary Disease/ or (coronary adj (disease* or disorder* or failure)).mp.
6	exp Cerebrovascular Disorders/ or (cerebrovascular adj (disease* or disorder* or insufficienc* or occlusion*)).mp. or (vascular adj (disease* or disorder*)).mp. or (carotid* adj (disease* or disorder*)).mp.
7	exp Peripheral Arterial Disease/ or (arter* adj (disease* or disorder*)).mp.
8	exp Rheumatic Heart Disease/ or exp Heart Defects, Congenital/ or (heart adj3 (malform* or defect* or congeni*)).mp.
9	exp Venous Thrombosis/ or ((deep vein or deep venous) adj thrombos*).mp. or phlebothrombos*.mp.
10	exp Pulmonary Embolism/ or (pulmonar* adj (thromboembolism* or embolism* or disease* or disorder*)).mp.
11	exp Stroke/ or stroke.mp.
12	exp Neoplasms/ or Cancer*.mp. or neoplas*.mp. or tumor*.mp.
13	exp Lung Diseases/ or exp Respiratory Tract Diseases/ or exp Lung Diseases, Obstructive/ or ((lung* or respiratory or pulmonar* or airflow or airway) adj2 (disease* or obstruct* or hypersensitiv*)).mp. or exp Asthma/ or asthma*.mp. or exp Pulmonary Disease, Chronic Obstructive/ or exp Respiratory Hypersensitivity/
14	exp Diabetes Mellitus/ or diabet*.mp.
15	exp Autoimmune Diseases/ or ((autoimmun* or auto immun* or autoaggress* or auto aggress*) adj (disorder* or disease*)).mp.
16	exp Metabolic Syndrome/ or exp Metabolic Diseases/ or ((metabolic or insulin resistance) adj (disorder* or disease* or syndrome*)).mp.
17	exp Obesity/ or obes*.mp.
18	exp Osteoporosis/ or osteoporo*.mp. or bone loss.mp. or exp osteolysis/ or osteolysis.mp. or bone resorption.mp.
19	exp Parkinson disease/ or parkinson*.mp. or paralysis agitans.mp.
20	exp Arthritis/ or arthriti*.mp. or polyarthriti*.mp. or rheumarthriti*.mp.
21	exp Kidney Diseases/ or (kidney adj (disease* or disorder*)).mp.
22	exp Liver Diseases/ or (liver adj (disease* or disorder* or dysfunction*)).mp.
23	exp Hypertension/ or high blood pressure*.mp. or hypertens*.mp.
24	exp Hyperlipidemias/ or hyperlipem*.mp. or hyperlipidem*.mp. or lipem*.mp. or lipidem*.mp.
25	exp Hypercholesterolemia/ or ((high* or elevat*) adj cholesterol*).mp. or hypercholesterem*.mp. or hypercholesterolem*.mp.
26	exp Hypertriglyceridemia/ or hypertriglyceridem*.mp.
27	exp Thyroid Diseases/ or (thyroid adj (disease* or disorder*)).mp. or exp Hyperthyroidism/ or hyperthyroid*.mp. or exp Hypothyroidism/ or hypothyroid*.mp. or ((thyroid-stimulating hormone* or tsh) adj deficien*).mp.
28	exp Motor Neuron Disease/ or motor neuron* disease*.mp. or lateral scleros*.mp. or motor system disease*.mp.
29	exp Multiple Sclerosis/ or multiple sclerosis.mp. or disseminated sclerosis.mp.
30	exp Emphysema/ or emphysema*.mp.
31	exp Bronchitis/ or bronchit*.mp.
32	1 or 2 or 3 or 4 or 5 or 6 or 7 or 8 or 9 or 10 or 11 or 12 or 13 or 14 or 15 or 16 or 17 or 18 or 19 or 20 or 21 or 22 or 23 or 24 or 25 or 26 or 27 or 28 or 29 or 30 or 31
33	exp Mental Disorders/ or exp Psychotic Disorders/ or ((mental* or psychiatr* or psycho*) adj (disorder* or disease* or illness*)).mp.
34	exp Depressive Disorder, Major/ or exp Depression/ or Depress*.mp. or MDD.mp.
35	exp Anxiety Disorders/ or exp Anxiety/ or anxi*.mp.

36	exp Phobic Disorders/ or phobi*.mp.
37	exp Schizophrenia/ or schizophreni*.mp. or hebephreni*.mp.
38	exp Somatoform Disorders/ or ((somatoform* or somati* or medically unexplained or briquet or pain) adj (disorder* or syndrome* or symptom*)).mp. or exp Medically Unexplained Symptoms/
39	exp Dissociative Disorders/ or (dissociative adj (disorder* or hysteri* or reaction*)).mp. or dissociation*.mp.
40	exp Hysteria/ or hysteri*.mp.
41	exp Mood Disorders/ or ((affective* or mood*) adj (disorder* or disease* or illness* or symptom*)).mp.
42	exp Stress Disorders, Post-Traumatic/ or PTSD.mp. or ((post trauma* or posttrauma*) adj (stress* or neurose*)).mp. or combat disorder*.mp. or war disorder*.mp.
43	exp Cognition Disorders/ or ((cognitive or cognition or mental or neurocognitive) adj (dysfunction* or decline* or impairment* or deterioration* or disorder* or illness* or disease*)).mp.
44	exp Personality Disorders/ or personality disorder*.mp.
45	exp "Disruptive, Impulse Control, and Conduct Disorders"/ or impulse control disorder*.mp. or intermittent explosive disorder*.mp.
46	exp "Feeding and Eating Disorders"/ or ((eating or appetite or feeding) adj disorder*).mp.
47	exp Bipolar Disorder/ or ((bipolar or mani*) adj (disorder* or illness* or disease*)).mp.
48	exp Obsessive-Compulsive Disorder/ or OCD*.mp. or ((obsess*-compulsi* or obsess* or compulsi*) adj (disorder* or illness* or disease* or neuros*)).mp.
49	exp Panic Disorder/ or (panic adj (attack* or disorder*)).mp.
50	exp Agoraphobia/ or agoraphobi*.mp.
51	exp Neurotic Disorders/ or neuros*.mp. or neurotic disorder*.mp. or psychoneuros*.mp.
52	33 or 34 or 35 or 36 or 37 or 38 or 39 or 40 or 41 or 42 or 43 or 44 or 45 or 46 or 47 or 48 or 49 or 50 or 51
53	exp Communicable Diseases/ or ((communic* or contag* or transmi* or infect*) adj (disease* or infection* or illness*)).mp.
54	exp Bacterial Infections/ or bacteri* infection*.mp.
55	exp Conjunctivitis/ or conjunctivitis.mp.
56	exp HIV/ or hiv.mp. or Human immuno deficiency virus.mp.
57	exp Acquired Immunodeficiency Syndrome/ or AIDS.mp. or immunodeficiency associated virus.mp. or immun* deficiency associated virus.mp. or acquired immunodeficiency syndrome*.mp. or acquired immun* deficiency syndrome*.mp.
58	exp Buruli Ulcer/ or Bairnsdale.mp. or Buruli.mp.
59	exp Onchocerciasis/ or onchocer*.mp.
60	hepatitis.mp. or exp Hepatitis B/ or exp Hepatitis C/
61	exp Leishmaniasis/ or leishmania*.mp.
62	exp Leprosy/ or lepros*.mp. or hansen*.mp.
63	exp Elephantiasis, Filarial/ or elephantias*.mp. or filaria*.mp.
64	exp Trachoma/ or egyptian ophthalmia*.mp. or trachoma*.mp.
65	exp Chikungunya Fever/ or chickungunya.mp. or chikungunya.mp.
66	exp Taeniasis/ or taenia*.mp.
67	exp Cysticercosis/ or cysticercos*.mp.
68	exp Echinococcosis/ or hydatid*.mp. or echinococc*.mp.
69	exp Chagas Disease/ or trypanosom*.mp. or chagas.mp.
70	exp Trypanosomiasis/ or sleeping sickness.mp.
71	exp Encephalitis, Japanese/ or (japanese adj3 encephalitis).mp.
72	exp Syphilis/ or syphilis.mp.
73	53 or 54 or 55 or 56 or 57 or 58 or 59 or 60 or 61 or 62 or 63 or 64 or 65 or 66 or 67 or 68 or 69 or 70 or 71 or 72
74	exp Tuberculosis/
75	Tuberculos*.mp.
76	TB.mp.

For peer review only - http://bmjopen.bmj.com/site/about/guidelines.xhtml

77	koch*.mp.
78	exp Tuberculosis/ or Tuberculos*.mp. or TB.mp. or koch*.mp.
79	(multiple adj (ill* or disease* or condition* or syndrom* or disorder*)).mp.
80	((Cooccur* or co-occur* or coexist* or co-exist* or multipl* or concord* or discord* or long-term or physical*) adj3 (disease* or ill* or care or condition* or disorder* or health* or medication* or symptom* or syndrom*)).mp.
81	(comorbid* or multimorbid* or co-occurren* or co-morbid* or Multidisease* or multi-disease*).mp.
82	(comorbid* or multimorbid* or co-occurren* or co-morbid* or multi-morbid* or Multidisease* or multi-disease*).mp.
83	exp Comorbidity/ or exp Multimorbidity/ or exp Multiple Chronic Conditions/
84	79 or 80 or 81 or 82 or 83
85	exp "Systematic Review"/
86	"systematic review*".m_titl.
87	exp Meta-Analysis/
88	"meta-analys*".m_titl.
89	exp "Systematic Review"/ or "systematic review*".m_titl. or exp Meta-Analysis/ or "meta-analys*".m_titl.
90	32 or 52 or 73
91	(32 or 52 or 73) and 78
92	(32 or 52 or 73) and 78 and 84
93	(32 or 52 or 73) and 78 and 84 and 89
94	exp Animals/ not exp Humans/
95	((32 or 52 or 73) and 78 and 84) not 94
96	((32 or 52 or 73) and 78 and 84 and 89) not 94
97	((32 or 52 or 73) and 78 and 89) not 94

For peer review only - http://bmjopen.bmj.com/site/about/guidelines.xhtml

Supplementary Table 2. Reasons for exclusion of references assessed in full text.

**Wrong population**	
1.	Shivakoti, Rupak and Sharma, Davina and Mamoon, Gabeena and Pham, Kiemanh. Association of HIV infection with extrapulmonary tuberculosis: a systematic review. Infection, 2017; 45(1):44501.
2.	Nasiri, Mohammad Javad and Dabiri, Hossein and Darban-Sarokhalil, Davood and Hashemi Shahraki, Abdolrazagh. Prevalence of Non-Tuberculosis Mycobacterial Infections among Tuberculosis Suspects in Iran: Systematic Review and Meta-Analysis. PloS one, 2015; 10(6):e0129073.
3.	Yaghoubi A. and Salehabadi S. and Abdeahad H. and Hasanian S.M. and Avan A. and Yousefi M. and Jamehdar S.A. and Ferns G.A. and Khazaei M. and Soleimanpour S. Tuberculosis, human immunodeficiency viruses and TB/HIV co-infection in pregnant women: A meta-analysis. Clinical Epidemiology and Global Health, 2020; 8(4):1312-1320.
4.	Manrique, RD and CastaÃ±eda, HL. RelaciÃ³n entre haber tenido tuberculosis y desarrollar enfermedad pulmonar obstructiva crÃ³nica. RevisiÃ³n sistemÃ¡tica y meta anÃ¡lisis. Med. U.P.B, 2015; 34(2):115-125.
5.	Fadaee, Manouchehr and Rashedi, Jalil and Arabi, Sepideh and Poor, Behroz Mahdavi and Kafil, Hossein Samadi and Pourostadi, Mahya and Kazemi, Abdolhassan and Asgharzadeh, Mohammad. Stopping of the Downtrend of Tuberculosis in Iran, a Systematic Review of Associated Risk Factors. Infectious disorders drug targets, 2020; 20(3):367-373.
6.	Bailey, S L and Ayles, H. Association between diabetes mellitus and active tuberculosis in Africa and the effect of HIV. Tropical medicine & international health : TM & IH, 2017; 22(3):261-268.
7.	Berhan, Asres and Berhan, Yifru and Yizengaw, Desalegn. A meta-analysis of drug resistant tuberculosis in Sub-Saharan Africa: how strongly associated with previous treatment and HIV co-infection?. Ethiopian journal of health sciences, 2013; 23(3):271-82.
8.	Gao Y. and Liu M. and Chen Y. and Shi S. and Geng J. and Tian J. Association between tuberculosis and COVID-19 severity and mortality: A rapid systematic review and meta-analysis. Journal of Medical Virology, 2020; ():.
9.	Berhan A. and Berhan Y. and Yizengaw D. A meta-analysis of drug resistant tuberculosis in Sub-Saharan Africa: how strongly associated with previous treatment and HIV co-infection?. Ethiopian journal of health sciences, 2013; 23(3):271-282.
10.	Judith Udeh, Jabulani Ncayiyana, Sibusiso Mkwananzi. The prevalence of latent tuberculosis infection and associated risk factors among young people in South Africa: a systematic review and meta-analysis.
11.	Demeke Geremew. Tuberculosis and its association with ART initiation in HIV patients in Ethiopia.
12.	Eleni Seyoum, Alemayehu Worku. The effect of co-infection (hepatitis b or c viruses & tuberculosis) on the outcome of antiretroviral therapy in resource limited countries: systematic review and meta-analysis.
13.	Diana Ramos, Ana Carolina Fragoso Motta, Alan Grupioni LourenÃ§o, Lara Maria Alencar Ramos Innocentini, Maria ConceiÃ§Ã£o Pereira Saraiva. Influence of HIV and tuberculosis co-infection on the occurrence of oral candidiasis: a systematic review and meta analysis.
**Does not report data on people with TB+1 chronic condition**	
14.	Oga-Omenka, Charity and Tseja-Akinrin, Azhee and Sen, Paulami and Mac-Seing, Muriel and Agbaje, Aderonke and Menzies, Dick and Zarowsky, Christina. Factors influencing diagnosis and treatment initiation for multidrug-resistant/rifampicin-resistant tuberculosis in six sub-Saharan African countries: a mixed-methods systematic review. BMJ global health, 2020; 5(7):.
15.	Tamuzi, Jacques L and Ayele, Birhanu T and Shumba, Constance S and Adetokunboh, Olatunji O and Uwimana-Nicol, Jeannine and Haile, Zelalem T and Inugu, Joseph and Nyasulu, Peter S. Implications of COVID-19 in high burden countries for HIV/TB: A systematic review of evidence. BMC infectious diseases, 2020; 20(1):744.
16.	Walaza, Sibongile and Cohen, Cheryl and Tempia, Stefano and Moyes, Jocelyn and Nguweneza, Athermon and Madhi, Shabir A and McMorrow, Meredith and Cohen, Adam L. Influenza and tuberculosis co-infection: A systematic review. Influenza and other respiratory viruses, 2020; 14(1):77-91.
17.	Tola, Habteyes Hailu and Holakouie-Naieni, Kourosh and Lejisa, Tadesse and Mansournia, Mohammad Ali and Yaseri, Mehdi and Tesfaye, Ephrem and Mola, Million. Is hypothyroidism rare in multidrug resistance tuberculosis patients on treatment? A systematic review and meta-analysis. PloS one, 2019; 14(6):e0218487.
18.	Nliwasa, Marriott and MacPherson, Peter and Gupta-Wright, Ankur and Mwapasa, Mphatso and Horton, Katherine and Odland, Jon O and Flach, Clare and Corbett, Elizabeth L. High HIV and active tuberculosis prevalence and increased mortality risk in adults with symptoms of TB: a systematic review and meta-analyses. Journal of the International AIDS Society, 2018; 21(7):e25162.
19.	Seegert, Anneline Borchsenius and Rudolf, Frauke and Wejse, Christian and Neupane, Dinesh. Tuberculosis and hypertension-a systematic review of the literature. International journal of infectious diseases : IJID : official publication of the International Society for Infectious Diseases, 2017; 56():54-61.
20.	Wu, Shan-shan and Zhang, Yue-lun and Wang, Wei-wei and Chen, Ru and Sun, Feng and Zhan, Si-yan. [Liver injury associated with treatment of multidrug-resistant tuberculosis: a systematic review and meta-analysis]. Beijing da xue xue bao. Yi xue ban = Journal of Peking University. Health sciences, 2014; 46(3):417-23.
21.	Patra, Jayadeep and Jha, Prabhat and Rehm, Jurgen and Suraweera, Wilson. Tobacco smoking, alcohol drinking, diabetes, low body mass index and the risk of self-reported symptoms of active tuberculosis: individual participant data (IPD) meta-analyses of 72,684 individuals in 14 high tuberculosis burden countries. PloS one, 2014; 9(5):e96433.
22.	Li, Xin-Xu and Zhou, Xiao-Nong. Co-infection of tuberculosis and parasitic diseases in humans: a systematic review. Parasites & vectors, 2013; 6(101462774):79.
23.	Sathiyamoorthy R. and Kalaivani M. and Aggarwal P. and Gupta S.K. Prevalence of pulmonary tuberculosis in India: A systematic review and meta-analysis. Lung India, 2020; 37(1):45-52.
24.	Zaidi A.K.M. and Awasthi S. and DeSilva H.J. Burden of infectious diseases in South Asia. British Medical Journal, 2004; 328(7443):811-815.
25.	Mortazavi, Hamed and Ghazalibina, Mehran and Mansouri, Shamseddin and Khaledi, Azad and Saburi, Ehsan. Pulmonary Fungal Co-Infection Prevalence among Iranian Patients with Pulmonary Tuberculosis: A Systematic Review and Meta-Analysis. SAINS MALAYSIANA, 2019; 48(12):2717-2725.
26.	Tanya Diefenbach-Elstob, Patricia Graves, Emma McBryde, David Plummer, Jeffrey Warner. Extrapulmonary tuberculosis in a global context: a systematic review of epidemiology and risk factors.
27.	Worku Jimma, Ahmed Abdulahi. Prevalence and risk factors of multidrug-resistant tuberculosis in Iran and its neighboring countries: systematic review and meta-analysis.

For peer review only - http://bmjopen.bmj.com/site/about/guidelines.xhtml

28.	Simon Collin, Dominik Zenner, Ibrahim Abubakar, Marieke van der Werf, Sarah Anderson, Gerard de Vries, Knut Lonnroth, Emily Newton. Effectiveness of interventions for TB control and prevention in countries of low and medium TB incidence: a systematic review of reviews.
29.	Yohannes Gezahagn, Yasin Mohammed, Alemseged Abdissa, Sophia Hussien, Guday Emire, Mirgissa Kaba. Delays in the diagnosis and treatment of tuberculosis in Ethiopia: a systematic review.
30.	Victor Vega, Sharon Rodriguez, Larissa Otero, Carlos Seas. Systematic review and meta-analysis on recurrent tuberculosis and associated risk factors.
31.	Mahdi Afshari, Bagheri, Saeed Barzegari. A protocol for estimating the risk factors of treatment default among patients with tuberculosis using systematic review and meta-analysis.
32.	Nguyen Tien Huy, Hieu Truong Hong, Mohammad Rashidul Hashan, Hazem Faraj, Tran Thuy Huong Quynh, Ahmed Saber Abdelrahman, Ahmad Morad, Khaled Mohy Ismaeil, Mohamed Gomaa Kamel. Hyponatremia in tubercular meningitis: a systematic review and meta-analysis.
33.	Trent Herdman, Justine Zhang, Sumona Datta, Matthew Saunders, Marco Tovar, Rosario Montoya, Carlton Evans. Clinical characteristics of unrecognised pulmonary tuberculosis detected through comprehensive population-based prevalence surveys: a systematic review.
34.	Olena Ivanova, Verena Hoffmann, Celso Khosa, Jule Witzleb, Michael Hoelscher, Andrea Rachow. Pulmonary outcomes in patients with tuberculosis: systematic review and meta-analysis.
35.	Shamanthi Jayasooriya, Caroline Mitchell. A systematic review of what proportion of adult patients attending TB treatment programmes in Africa have been identified as not having active tuberculosis?.
36.	Petrus Kosmas, Elize Pietersen, Jabulani Ncayiyana, Mark Engel. Extensively drug resistant tuberculosis in Africa; prevalence and factors associated; a systematic review and meta-analysis.
37.	Rodney Ehrlich, Paula Akugizibwe, Nandi Siegfried, David Rees. Systematic review of association between silica/silicosis and tuberculosis.
38.	Saravanan N, Silambhu Chelvi Ramesh, Rajesh Mondal, Banu Rekha, Basilea Watson, Rajendran Krishnan, Kannan Thiruvengadam. Nutritional status of patients with tuberculosis among the tribal population in India.
39.	Philippe Armel Awana, Celestin Danwang, Joel Noutakdie Tochie, Jean Joel Bigna. Global epidemiology of venous thromboembolism in people with active tuberculosis: a systematic review and meta-analysis.
40.	Sally Hayward, Rachel Wittenberg, Lisa Stockdale. Systematic review of the relationship between Herpesviridae infections and tuberculosis.
41.	Linh Nguyen, Quyen Bui. The incidence rate of multidrug-resistant Tuberculosis/ Rifampicin-resitant Tuberculosis and associated factors: a meta-analysis.
42.	Mpho Refilwe Disang, Christine Campbell, David Weller. Prevalence and patterns of communicable and noncommunicable diseases multimorbidity in sub-Saharan Africa; a protocol for systematic review.
43.	Tamuzi Lukenze Jacques, Peter Nyasulu, Ayele Birhanu. COVID-19 impact on HIV and tuberculosis programmes: a systematic review and meta-synthesis of evidence.
44.	Sara Hussein, May Al-Asmar, Ahmed Awaisu, Yaw Owusu, Alaa Soliman, Sara Murshid, Muna Al-Maslmani, Faraj Howady. Prevalence and Risk Factors of Drug Resistant Tuberculosis in the Eastern Mediterranean Region: A Systematic Review.
45.	Nannan Wang, Jia Zhu, Wenpei Liu, Miaomiao Yang, Honggang Yi, Shaowen Tang. Incidence, temporal trend and factors associated with anti-tuberculosis drug-induced liver injury: A systematic review and meta-analysis.
46.	Tasmiya Ira, Elizabeth Ojewole, Pravina Laljeeth, Richard Beharilal. Determinants of adverse drug reactions due to tuberculosis therapy in African countries: a systematic review.
47.	Danwang, Celestin and Bigna, Jean Joel and Awana, Armel Philippe and Nzalie, Rolf Nyah-Tuku and Robert, Annie. Global epidemiology of venous thromboembolism in people with active tuberculosis: a systematic review and meta-analysis. Journal of thrombosis and thrombolysis, 2020; ():.
48.	Dessie, Getenet and Negesse, Ayenew and Wagnew, Fasil and Amare, Desalegne and Tiruneh, Balew Zeleke and Mulugeta, Henok and Mekonen, Berhanu Abebaw and Haile, Dessalegn and Ayalew, Tilksew and Habtewold, Tesfa Dejenie. Intestinal parasites and HIV in Ethiopian tuberclosis patients: A systematic review and meta-analysis. Current therapeutic research, clinical and experimental, 2020; 93():100603.
49.	Hadadi-Fishani, Mehdi and Shakerimoghaddam, Ali and Khaledi, Azad. Candida coinfection among patients with pulmonary tuberculosis in Asia and Africa; A systematic review and meta-analysis of cross-sectional studies. Microbial pathogenesis, 2020; 139():103898.
50.	Alemu, Ayinalem and Bitew, Zebenay Workneh and Worku, Teshager. Intestinal parasites co-infection among tuberculosis patients in Ethiopia: a systematic review and meta-analysis. BMC infectious diseases, 2020; 20(1):510.
51.	Barzegari, Saeed and Afshari, Mahdi and Movahednia, Mahtab and Moosazadeh, Mahmood. Prevalence of anemia among patients with tuberculosis: A systematic review and meta-analysis. The Indian journal of tuberculosis, 2019; 66(2):299-307.
52.	Girum, Tadele and Muktar, Ebrahim and Lentiro, Kifle and Wondiye, Habtamu and Shewangizaw, Misgun. Epidemiology of multidrug-resistant tuberculosis (MDR-TB) in Ethiopia: a systematic review and meta-analysis of the prevalence, determinants and treatment outcome. Tropical diseases, travel medicine and vaccines, 2018; 4(101674442):5.
53.	Keflie, Tibebeselassie Seyoum and Nolle, Nils and Lambert, Christine and Nohr, Donatus and Biesalski, Hans Konrad. Vitamin D deficiencies among tuberculosis patients in Africa: A systematic review. Nutrition (Burbank, Los Angeles County, Calif.), 2015; 31(10):1204-12.
54.	Allwood, Brian W and Myer, Landon and Bateman, Eric D. A systematic review of the association between pulmonary tuberculosis and the development of chronic airflow obstruction in adults. Respiration; international review of thoracic diseases, 2013; 86(1):76-85.
55.	Azzeri A. and Ching G.H. and Jaafar H. and Noor M.I.M. and Razi N.A. and Then A.Y.-H. and Suhaimi J. and Kari F. and Dahlui M. A review of published literature regarding health issues of coastal communities in Sabah, Malaysia. International Journal of Environmental Research and Public Health, 2020; 17(5):1533.
56.	Hosseini M. and Shakerimoghaddam A. and Ghazalibina M. and Khaledi A. Aspergillus coinfection among patients with pulmonary tuberculosis in Asia and Africa countries; A systematic review and meta-analysis of cross-sectional studies. Microbial Pathogenesis, 2020; 141():104018.
57.	Cormier M. and Schwartzman K. and N'Diaye D.S. and Boone C.E. and dos Santos A.M. and Gaspar J. and Cazabon D. and Ghiasi M. and Kahn R. and Uppal A. and Morris M. and Oxlade O. Proximate determinants of tuberculosis in Indigenous peoples worldwide: a systematic review. The Lancet Global Health, 2019; 7(1):e68-e80.

For peer review only - http://bmjopen.bmj.com/site/about/guidelines.xhtml

58.	Azhar G. DOTS for TB relapse in India: A systematic review. Lung India, 2012; 29(2):147-153.
59.	Flannery E. and Rosenthal M. and Roberts E.R. and Henderson J.J. and Davis B.M. and Foxman B. Synergy and anergy in the context of infectious disease epidemiology: The role of co-infection. American Journal of Epidemiology, 2011; 173():S234.
60.	Khadija Ibrahim, Nan Shwe Htun, Karim Manji, Peter Odermatt, Marcel Tanner, JÃ¼rg Utzinger, Nicole Probst Hensch, Lukas Fenner. Does the association between helminths and tuberculosis have influence on each other?.
61.	Katherine Horton, Peter MacPherson, Richard White, Rein Houben, Liz Corbett. Gender differences in tuberculosis prevalence in low- and middle-income countries.
62.	Dalya Eltayeb, Elize Pietersen, Mark Engel, Leila Abdullahi. Factors associated with patient and health system delay in diagnosis and treatment for pulmonary tuberculosis in Middle East and North Africa (MENA).
63.	Erick Bunyasi, Leila Abdullahi, Bey-Marrie Schmidt, Hennie Geldenhuys, Robin Wood, Mark Hatherill. A systematic review on the prevalence and incidence of tuberculosis disease and infection prevention and control measures among high school students in 22 high tuberculosis disease burden countries defined by the World Health Organisation.
64.	Anthony Byrne, Ben Marais, Carole Mitnick, Leonid Lecca, Guy Marks. Tuberculosis and asthma: a systematic review.
65.	Amare Tariku, Destaw Fetene. The effect of under-nutrition on non-adherence to anti-TB drugs in TB patients in Sub-Saharan Africa: a systematic review and meta-analysis.
66.	Habteyes Tola, Kourosh Holakouie-Naieni, Ephrem Tesfaye, Mohammad Mansournia, Mehdi Yaseri. Tuberculosis treatment interruption and its associated factors in Ethiopia: a systematic review and meta-analysis.
67.	Ahmed Hossain, Zeeba Sultana, Farhana Hoque. Association between HIV infection and multidrug-resistant tuberculosis in Africa and Asia: a systematic review and meta-analysis.
68.	Ayinalem Alemu Shitie, Zebenay Workneh Bitew, Teshager Worku. Intestinal parasites co-infection among tuberculosis patients in Ethiopia: a systematic review and meta-analysis.
69.	Birhan Alemnew, Setegn Eshetie, Asmamaw Demis. The prevalence and its associated risk factors of extra pulmonary tuberculosis in Ethiopia; systematic review and meta-analysis.
70.	Getu Diriba, Habteyes Tola, Ayinalem Alemu, Abebaw Kebede. Prevalence of drug resistance and its risk factors among extra pulmonary tuberculosis patients in Ethiopia: a systematic review and meta-analysis.
71.	Balew Arega. Prevalence rate of undiagnosed tuberculosis in the community in Ethiopia from 2001 to 2014: systematic review and meta-analysis.
72.	Kerri Viney, Luis Furuya-Kanamori, Kefyalew Addis Alene, Kinley Wangdi, Anthony Byrne, Justin Clark. Types and burden of disabilities associated with tuberculosis: protocol for a systematic review.
73.	Getahun Molla Kassa, Atalay Goshu Muluneh, Dawit Tefera Fentie, Mehari Woldemariam Merid. Magnitude and effect of anemia on treatment outcome of drug-resistance tuberculosis in Sub-Sahara Africa: systematic review and meta-analysis.
74.	Adam Wondmieneh, Getnet Gedefaw, Addisu Getie, Asmamaw Demis. Undernutrition among adult tuberculosis patients in Ethiopia: A Systematic review and meta- analysis.
75.	Badawi A. and Gregg B. and Vasileva D.. Systematic analysis for the relationship between obesity and tuberculosis. Public Health, 2020; 186():246-256.
**Does not report data on any of our outcomes of interest**	
76.	Lohiya, Ayush and Suliankatchi Abdulkader, Rizwan and Rath, Rama Shankar and Jacob, Olivia and Chinnakali, Palanivel and Goel, Akhil Dhanesh and Agrawal, Sumita. Prevalence and patterns of drug resistant pulmonary tuberculosis in India-A systematic review and meta-analysis. Journal of global antimicrobial resistance, 2020; 22(101622459):308-316.
77.	Noykhovich, Ekaterina and Mookherji, Sangeeta and Roess, Amira. The Risk of Tuberculosis among Populations Living in Slum Settings: a Systematic Review and Meta-analysis. Journal of urban health : bulletin of the New York Academy of Medicine, 2019; 96(2):262-275.
78.	Tola, Habteyes Hailu and Tol, Azar and Shojaeizadeh, Davoud and Garmaroudi, Gholamreza. Tuberculosis Treatment Non-Adherence and Lost to Follow Up among TB Patients with or without HIV in Developing Countries: A Systematic Review. Iranian journal of public health, 2015; 44(1):44501.
79.	Naing, Cho and Mak, Joon Wah and Maung, Mala and Wong, Shew Fung and Kassim, Ani Izzuani Binti Mohd. Meta-analysis: the association between HIV infection and extrapulmonary tuberculosis. Lung, 2013; 191(1):27-34.
80.	Tengan F.M. and Figueiredo G.M. and Leite O.H.M. and Nunes A.K.S. and Manchiero C. and Dantas B.P. and Magri M.C. and Barone A.A. and Bernardo W.M. Prevalence of multidrug-resistant tuberculosis in Latin America and the Caribbean: a systematic review and meta-analysis. Tropical Medicine and International Health, 2020; 25(9):1065-1078.
81.	Xia Y.Y. and Zhan S.Y. Systematic review of anti-tuberculosis drug induced adverse reactions in China. Zhonghua jie he he hu xi za zhi = Zhonghua jiehe he huxi zazhi = Chinese journal of tuberculosis and respiratory diseases, 2007; 30(6):419-423.
82.	Perez-Guzman C. and Vargas M.H. and Torres-Cruz A. and Villarreal-Velarde H. Does aging modify pulmonary tuberculosis?: A meta-analytical review. Chest, 1999; 116(4):961-967.
83.	Mhlengi Vella Ncube, Tivani Mashamba-Thompson. Evidence on implementation of HIV/AIDS, STIs and TB related point of care diagnostics in low and medium income countries.
84.	Marie Varughese, Michael Li, Courtney Heffernan. Time to diagnosis and treatment of pulmonary tuberculosis in indigenous peoples: a systematic review.
85.	Md Asiful Islam, Mahfuza Marzan, Shoumik Kundu. Prevalence of antibiotic-resistant pulmonary tuberculosis in Bangladesh: A systematic review and meta-analysis.
86.	Balewgizie Sileshi Tegegne, Tesfa Dejenie Habtewold, Melkamu Merid Mengesha, Hans Burgerhof. Diabetes mellitus and multi-drug resistant tuberculosis: a protocol for a systematic review and meta-analysis.
87.	Benjamin Momo Kadia, Fongwen Noah Takah, Christian Akem Dimala, Victoria Simms. A systematic review and meta-analysis of the association between integrated tuberculosis and human immuno-deficiency virus therapy and tuberculosis treatment outcomes in sub-Saharan Africa.
**Wrong study design**	
88.	Ruslami, Rovina and Aarnoutse, Rob E and Alisjahbana, Bachti and van der Ven, Andre J A M and van Crevel, Reinout. Implications of the global increase of diabetes for tuberculosis control and patient care. Tropical medicine & international health : TM & IH, 2010; 15(11):1289-99.
89.	Sarkar M. and Srinivasa and Madabhavi I. and Kumar K. Tuberculosis associated chronic obstructive pulmonary disease. Clinical Respiratory Journal, 2017; 11(3):285-295.

For peer review only - http://bmjopen.bmj.com/site/about/guidelines.xhtml

90.	Pablo-Villamor M.P. and Benedicto J.P. and Benedicto M.T.J.U. and Perez V.M. Screening for diabetes mellitus in patients diagnosed with pulmonary tuberculosis. Phillippine Journal of Internal Medicine, 2014; 52(4):44440.
91.	Sharma P. and Visnegarwala F. and Tripathi V. Burgeoning double burden of tuberculosis and diabetes in India: Magnitude of the problem - Strategies and solutions. Clinical Epidemiology and Global Health, 2014; 2(3):107-116.
92.	Wenjie Fang, Min Chen, Jia Liu, Weihua Pan, Wanqing Liao. Tuberculosis/Cryptococcosis co-infection in China from 1965 to 2016: a probably underestimated challenge.
93.	Kouemo Motse, Dorgelesse F and Nsagha, Dickson Shey and Adiogo, Dieudonne and Kojom Foko, Loick P and Teyim, Pride M and Chichom-Mefire, Alain and Nguedia Assob, Jules C. Tuberculosis Chemotherapy Outcome in the Littoral Region of Cameroon: A Meta-analysis of Treatment Success Rate between 2014 and 2016. BioMed research international, 2020; 2020(101600173):8298291.
94.	Cheng, Jun and Zhang, Hui and Zhao, Yan Lin and Wang, Li Xia and Chen, Ming Ting. Mutual Impact of Diabetes Mellitus and Tuberculosis in China. Biomedical and environmental sciences : BES, 2017; 30(5):384-389.
95.	Balinda I.G. and Sugrue D.D. and Ivers L.C. More Than Malnutrition: A Review of the Relationship between Food Insecurity and Tuberculosis. Open Forum Infectious Diseases, 2019; 6(4):.
96.	Guto J.A. and Bii C.C. and Denning D.W. Estimated burden of fungal infections in Kenya. Journal of Infection in Developing Countries, 2016; 10(8):777-784.
97.	Doherty A.M. and Kelly J. and McDonald C. and O'Dywer A.M. and Keane J. and Cooney J. A review of the interplay between tuberculosis and mental health. General Hospital Psychiatry, 2013; 35(4):398-406.
98.	Creswell J. and Raviglione M. and Ottmani S. and Migliori G.B. and Uplekar M. and Blanc L. and Sotgiu G. and Lonnroth K. Series: "Update on tuberculosis" - Tuberculosis and noncommunicable diseases: Neglected links and missed opportunities. European Respiratory Journal, 2011; 37(5):1269-1282.
99.	Agnihotram R.V. Reviewing disease burden among rural Indian women. Online Journal of Health and Allied Sciences, 2004; 3(2):.
100.	Baziz A. Miliary tuberculosis associated with adult respiratory distress syndrome. Annales de Medecine Interne, 1995; 146(2):114-122.
101.	CedeÃ±o-Burbano, Anuar Alonso and CerÃ³n-Ortega, Ronal Fredy and Pacichana-Agudelo, Carlos Eberth and MuÃ±oz-GarcÃa, David AndrÃ©s and Galeano-TriviÃ±o, Gerardo Alfonso and Cardona-GÃ³mez, Diana Catalina and Manquillo-Arias, William AndrÃ©s and Plaza-Rivera, Regina Victoria. Parasitismo intestinal y tuberculosis. Rev. Fac. Med. (BogotÃ¡), 2017; 65(4):673-677.
**No data from LMICs**	
102.	Lonnroth, Knut and Williams, Brian G and Cegielski, Peter and Dye, Christopher. A consistent log-linear relationship between tuberculosis incidence and body mass index. International journal of epidemiology, 2010; 39(1):149-55.
**Duplicated reference**	
103.	Noubiap, Jean Jacques and Nansseu, Jobert Richie and Nyaga, Ulrich Flore and Nkeck, Jan Rene and Endomba, Francky Teddy and Kaze, Arnaud D and Agbor, Valirie N and Bigna, Jean Joel. Global prevalence of diabetes in active tuberculosis: a systematic review and meta-analysis of data from 2.3 million patients with tuberculosis. The Lancet. Global health, 2019; 7(4):e448-e460.
104.	Zheng, Yixiang and Ma, Shujuan and Tan, Deming and Lu, Menghou. [A meta-analysis of liver lesions in hepatitis B patients undergoing anti-tuberculosis therapy]. Zhonghua gan zang bing za zhi = Zhonghua ganzangbing zazhi = Chinese journal of hepatology, 2014; 22(8):585-9.
105.	Liu, Jiao and Lu, Bing and Yan, Yan. [Meta analysis on the co-infection between Mycobacterium tuberculosis and HIV/AIDS in China]. Zhonghua liu xing bing xue za zhi = Zhonghua liuxingbingxue zazhi, 2013; 34(1):85-90.
106.	Pang P. and Sun C. and Duan W. and Liu S. and Bai S. and Ma Y. and Li R. and Liu F. A review on global head and neck tuberculosis cases from 980 papers and 5881 patients. International Journal of Oral and Maxillofacial Surgery, 2019; 48():185.
107.	Liu J. and Lu B. and Yan Y. [Meta analysis on the co-infection between Mycobacterium tuberculosis and HIV/AIDS in China]. Zhonghua liu xing bing xue za zhi = Zhonghua liuxingbingxue zazhi, 2013; 34(1):85-90.
108.	Carlos Almeida, Rachel Couban, Sun Kallyth, Li Wang, Jason Busse, Denise Silva. Predictors of in-hospital mortality among patients with pulmonary tuberculosis: systematic review and meta-analysis of observational studies.
109.	Bisson G.P. and Bastos M. and Campbell J.R. and Bang D. and Brust J.C. and Isaakadis P. and Lange C. and Menzies D. and Migliori G.B. and Pape J.W. and Palmero D. and Baghei P. and Tabarsi P. and Viiklepp P. and Vilbrun S. and Walsh J. and Marks S.M. Mortality in adults with multidrug-resistant tuberculosis and HIV by antiretroviral therapy and tuberculosis drug use: an individual patient data meta-analysis. The Lancet, 2020; 396(10248):402-411.
110.	Hong-Guang CHEN and Min LIU and Fang-Hui GU. Meta-analysis on the co-morbidity rate between tuberculosis and diabetes mellitus in China. Chinese Journal of Epidemiology, 2013; (12):1128-1133.
**Protocol of an already included study**	
111.	Tankeu, Aurel T and Bigna, Jean Joel and Nansseu, Jobert Richie and Endomba, Francky Teddy A and Wafeu, Guy Sadeu and Kaze, Arnaud D and Noubiap, Jean Jacques. Global prevalence of diabetes mellitus in patients with tuberculosis: a systematic review and meta-analysis protocol. BMJ open, 2017; 7(6):e015170.
112.	Marriott Nliwasa, Elizabeth Corbett, Peter MacPherson, Ankur-Gupta Wright, Katherine Horton. The Prevalence of HIV and Risk of Early Mortality in Adults with Suspected Tuberculosis in Low- and Middle- Income Countries: A Systematic Literature Review.
113.	Tamuzi Lukenze Jacques, Birhanu Bayele. Covid-19 implications in high burden HIV/TB countries: a systematic review of evidence.
114.	sanju gautam. Prevalence of diabetes mellitus among tuberculosis patients and itâ€™s impact on treatment outcome in South Asian Region: a systematic review and meta-analysis.
115.	Cesar Ugarte-Gil, Fiona Pearson. Systematic review and meta-analysis on the diabetes mellitus role on tuberculosis treatment outcomes.
116.	Aurel. T. Tankeu, Jean JoÃ«l R. Bigna, Jobert Richie N. Nansseu, Francky Teddy A. Endomba, Guy Sadeu Wafeu, Arnaud D. Kaze, Jean Jacques N. Noubiap. Global prevalence of diabetes mellitus in patients with tuberculosis: a systematic review and meta-analysis protocol.
117.	Chi Yan Leung, Hsi Lan Huang, Md. Mizanur Rahman, Sarah Krull Abe, Stuart Gilmour, Kenji Shibuya. Pulmonary and extra-pulmonary tuberculosis infection and the risk of pulmonary and extra-pulmonary malignancies: a systematic review and meta-analysis.

For peer review only - http://bmjopen.bmj.com/site/about/guidelines.xhtml

118.	Animut Alebel, Amsalu Taye Wendemagen, Cheru Tesema, Getiye Dejenu, Fasil Wagnew, Fasil Wagnew, Pammla Petrucka, Setegn Eshetie. Prevalence of diabetes mellitus among tuberculosis patients in Sub-Saharan Africa, and the impact of HIV infection: a systematic review and meta-analysis.
119.	Hasan Abolghasem Gorji, Nicola Luigi Bragazzi. A systematic review and meta-analysis of the prevalence of Hepatitis C virus (HCV) among tuberculosis patients.
120.	Aklilu Endalamaw, Setegn Eshetie, Sintayehu Ambachew, Demeke Geremew, Tesfa Dejenie Habtewold. HIV-infection and unknown HIV status of tuberculosis patients in Ethiopia: a systematic review and meta-analysis.
121.	C. Andrew Basham, Sarah Smith, James C. Johnston. Tuberculosis (TB) and cardiovascular disease (CVD) risk: a systematic review and meta-analysis.
122.	Abraham Assefa, Awoke Derbie, Abebe Shumet, Abaineh Munshae, Endalkachew Nibret, Fantahun Biadglegne. Epidemiology of Tuberculosis Lymphadenitis in Africa: a systematic reviews and meta-analysis.
123.	Habteyes Tola, Kourosh Holakouie-Naieni, Mohammad Mansournia, Mehdi Yaseri, Ephrem Tesfaye, Million Mola, Tadesse Lejisa Lejisa. Is hypothyroidism rare and does it require screening in multidrug-resistant tuberculosis patients on treatment? A systematic review and meta-analysis.
124.	Alvaro Schwalb, CÃ©sar Ugarte, Rodrigo Cachay, Paulo Ruiz, Adriana De la Flor. The association between tuberculosis and depression on poor outcomes of tuberculosis treatment: a systematic review.
125.	Dumessa Edessa, Moti Tolera, Bisrat Hagos. Poor outcomes to second-line tuberculosis treatment among multidrug-resistant patients in sub-Saharan Africa: a systematic review and meta-analysis.
**Full text could not be found**	
126.	Wongtrakul, Wasit and Charoenngam, Nipith and Ungprasert, Patompong. Tuberculosis and risk of coronary heart disease: A systematic review and meta-analysis. The Indian journal of tuberculosis, 2020; 67(2):182-188.
127.	Pang P. and Sun C. and Duan W. and Liu S. and Bai S. and Ma Y. and Li R. and Liu F. A review on global head and neck tuberculosis cases from 980 papers and 5881 patients. International Journal of Oral and Maxillofacial Surgery, 2019; 48():182.
128.	Yixiang ZHENG and Shujuan MA and Deming TAN and Menghou LU. A meta-analysis of liver lesions in hepatitis B patients undergoing anti-tuberculosis therapy. Chinese Journal of Hepatology, 2014; (12):585-589.
**Protocol**	
129.	Benjamin Momo Kadia, Desmond Aroke, Kevin Pene Njefi, Yves-Joel Tochie Noutakdie, Frank-Leonel Tianyi, Reine Suzanne Kadia, Christian Akem Dimala. Systematic review of therapeutic outcomes of multi-drug resistant tuberculosis and their predictors in adults receiving integrated treatment of tuberculosis and Human Immuno-deficiency Virus in low and middle-income countries: a study protocol.
130.	Noemia Siqueira-Filha. Costs of tuberculosis and HIV co-infection for the health system and families worldwide: a systematic literature review.
131.	Minmin Li. National, regional, and global prevalence of diabetes mellitus in patients with tuberculosis: a systematic review and meta-analysis.
132.	Yonggang Zhang, Linli Zheng, Lu Huang. The prevalence of depression in tuberculosis patients: a meta-analysis.
133.	Tamaryn J. Nicholson, Muhammad Osman, Rory Dunbar, Anneke. C. Hesseling, Florian Marx, Elisa Lopez Varela, James A. Seddon, Kogie Naidoo. A systematic review of risk factors for mortality among drug-susceptible and drug-resistant TB patients in South Africa (2010 â€“ 2018).
134.	Rebecca Harris, Fidel Vazquez. A systematic review and meta-analysis of the effect of HIV status on the incidence of tuberculosis disease among individuals with latent Mycobacterium tuberculosis infection.
135.	Nicolas Nunez, Aiswarya Nandakumar, Lakshmi, Aparna Menon, Boney Joseph. Risk of depression in pulmonary tuberculosis.
136.	Melese Teferi, Mekonnen Mekonnen, Hawult Adane. Determinants of tuberculosis (TB) treatment outcome in resource-limited settings: protocol for systematic review and meta-analysis.
137.	Liu Chun, yang Xie, Hulei Zhao, Jiansheng Li. Prevalence of pneumoconiosis and that complicated with pulmonary tuberculosis in China: a systemic review and meta-analysis.
138.	Melaku Kindie Yenit, Wubet Worku Takele, Achenef Asmamaw Muche, Setegn Eshetie Kebede, Dessie Abebaw Angaw. The effect of depression on Tuberculosis (TB) treatment outcome in Africa: A Protocol for systematic review and meta-analysis.
139.	Lucy Kaluvu, Asogwa Ogechukwu, Anna Marza Florensa, Daniel Boateng, Kerstin Klipstein-Grobusch. A systematic review to examine the patterns, prevalence and management of multimorbidity of communicable and non-communicable diseases in low- and middle- income countries.
140.	Marie Charmaine Sy, Adrian Espiritu, Jose Leonard Pascual. Global frequency of stroke in tuberculous meningitis: A systematic review and meta-analysis.
141.	Mogesie Necho, Mekonnen Tsehay, Asmare Belete. Prevalence and Associated Factors of Alcohol Use Disorder Among Tuberculosis Patients: A Systematic Review and Meta-analysis.
142.	Mogesie Necho, Mengesha Birkie. Psychological distress and Depression in Tuberculosis patients in Africa: A systematic Review and Meta-analysis.
143.	Javier Ogembo, Rebecca Ogembo, Paul Bain. Prevalence of drug-resistant tuberculosis in sub-Saharan Africa: systematic review and meta-analysis.
144.	Adhanom Baraki, Abel Dadi, Hanna Desyibelew. Epidemiology of depression among tuberculosis patients: a systematic review and meta-analysis of observational studies from Africa.
145.	Jing Wu, Jennifer M McGoogan, Zunyou Wu. Prevalence of HIV/TB co-infection worldwide: a systematic review and meta-analysis.
146.	Birhanie Mekuriaw, Alemayehu Molla, Zelalem Belayneh, Tsegaye Mehare. Depression and its determinants among individuals with Tuberculosis in Ethiopia: a systematic review and meta-analysis.
147.	Chalachew Adugna, Aklilu Enalamaw. Poor treatment adherence and associated factors among patients taking anti-tuberculosis drug in Ethiopia: systematic review and meta-analysis.
148.	Gebremedhin Berhe Gebregergs, Gebreamlak Gidey, Fitwi Tinsae, Gebremedhin Gebreziher, Selam Desalegn. Gender difference in co-morbid depression among tuberculosis patients in high burden countries: systematic review and meta-analysis.
149.	Belayneh Kefale, Mulugeta Molla, Amien Ewunetie, Amsalu Degu, Gobezie Temesgen. Treatment outcomes and associated factors among tuberculosis patients in Ethiopia: a systematic review and meta analysis.

For peer review only - http://bmjopen.bmj.com/site/about/guidelines.xhtml

150.	Alvaro Schwalb, Jorge InolopÃº, Eduardo Gotuzzo, Rodrigo Cachay, Fernando MejÃa, Larissa Otero, Carlos Seas, CÃ©sar Ugarte-Gil, Kristien Verdonck, Nicole Young. Clinical and epidemiological association between HTLV-1/2 and tuberculosis: systematic review.
151.	Demeke Geremew. Latent TB and its associated factors in Ethiopian: an aggregated and individual patientsâ€™ data meta-analysis.
152.	Carole Mitnick, Molly Franke, Celia Fung, Andrew Lindeborg. Clinical Outcomes of Individuals with COVID-19 and Tuberculosis Disease: a Living Systematic Review.
153.	Javier Cabrera, Vicente Cuba. Tuberculosis and lung cancer risk: a systematic review and meta-analysis.
154.	Jemal Abdela, Fuad Adem, Abraham Nigussie. Incidences and risks of drug-induced hepatotoxicity among Tuberculosis and HIV/ Tuberculosis co-infected patients in Sub Saharan Africa: a systematic review and meta-analysis.
**Conference abstract**	
155.	Schafer J.M. and Welwarth J. and Balk D.S. and Lee C. and Hardin J. and Hoffmann B. Rates of pericardial effusion in patients with HIV/tuberclosis co-infection: A systematic review and meta-analysis. Academic Emergency Medicine, 2017; 24():S97.
156.	Moniruzzaman A. and Kazanjian A. and Wong H. and Chowdhury M.M. and Elwood R.K. and Fitzgerald J.M. A systematic review on risk factors of mortality among Tb patients. American Journal of Respiratory and Critical Care Medicine, 2010; 181(1):.
157.	Yeats, J. and Patel, S. and Shete, P. B. and Cattamanchi, A. and Baker, B. J. Reactivation Risk Of Latent Tuberculosis Infection By Population Subgroup: A Systematic Review And Meta-Analysis. AMERICAN JOURNAL OF RESPIRATORY AND CRITICAL CARE MEDICINE, 2016; 193():.
158.	Huang Y.-S. Chronic hepatitis C may increase the risk of anti-tuberculosis drug-induced liver injury: A systematic review and meta-analysis. Hepatology International, 2017; 11(1):S842.
159.	Chen H. and Liu M. and Gu F. Meta-analysis on the co-morbidity rate between tuberculosis and diabetes mellitus in China. Zhonghua liu xing bing xue za zhi = Zhonghua liuxingbingxue zazhi, 2013; 34(11):1128-1133.
160.	Arruda S. and Loureiro C. and Almeida M. and Mendes D. and Grassi M.F.R. and Lapa J.R. and Kritski A. and Verdonck K. and Gotuzzo E. and Galvao-Castro B. Association between human T-Cell lymphotropic virus type 1 and 2 (HTLV 1/2) infection and tuberculosis: Systematic review and meta-analysis. Retrovirology, 2011; 8():A80.
161.	Hussain, M. S. and Siddiqui, A. N. and Najmi, A. K. Burden of diabetes mellitus among tuberculosis patients in Asia-Pacific region: Evidence from meta-analysis using real-world data. INTERNATIONAL JOURNAL OF INFECTIOUS DISEASES, 2018; 73():87.
162.	Jiao LIU and Bing LV and Yan YAN. Meta analysis on the co-infection between Mycobacterium tuberculosis and HIV/AIDS in China. Chinese Journal of Epidemiology, 2013; (12):85-90.
**Wrong publication type (letters to the editor, corrections, protocol for this meta-review)**	
163.	Pai, Madhukar and McCulloch, Michael and Colford, John M Jr. Meta-analysis of the impact of HIV on the infectiousness of tuberculosis: methodological concerns. Clinical infectious diseases : an official publication of the Infectious Diseases Society of America, 2002; 34(9):1285-7.
164.	Chen M. and Al-Hatmi A.M. and Chen Y. and Ying Y. and Fang W. and Xu J. and Hagen F. and Hong N. and Boekhout T. and Liao W. and Pan W. Cryptococcosis and tuberculosis co-infection in mainland China. Emerging Microbes and Infections, 2016; 5(9):e98.
165.	Anonymous. Correction: Association between HIV/AIDS and multi-drug resistance tuberculosis: A systematic review and meta-analysis (PLoS ONE). PLoS ONE, 2014; 9(2):e89709.
166.	Chen L. and Li N. and Liu M. and Zhang J. and Zhang H. High prevalence of multidrug-resistant tuberculosis in Zunyi, Guizhou province of China. Journal of Antimicrobial Chemotherapy, 2011; 66(10):2435-2437.
167.	Alexander Jarde, Ruimin Ma, Eugenia Romano, Helen Elsey, Kamran Siddiqi, Najma Siddiqi, Brendon Stubbs. A meta-review of systematic reviews of chronic disease multimorbidity in people with tuberculosis in low- and middle-income countries.

For peer review only - http://bmjopen.bmj.com/site/about/guidelines.xhtml

Supplementary Table 3: Study characteristics of systematic reviews reporting pooled outcomes of studies from LMICs

Lead author and year	Search dates and limits	Number of studies in LMIC/Total number of studies (countries covered)	Clusters covered	Outcomes	Quality rating
**TB + HIV**					
Alemu 2020[S54]	2009 to Jan 2020; English language, in Ethiopia	17/17 (Ethiopia)	TB +HIV	Prevalence*	Low
Arega 2020 [S24]	200 to 2019; English language, in Ethiopia	47 / 47 (Ethiopia)	TB +HIV	Prevalence*	Critically low
Bastos 2019 [S25]	period between 2008 and 2017; Brazil only	15 /15 (Brazil)	TB +HIV	Mortality*, Treatment outcomes*	Critically low
Bisson 2020 [S48]	2009 to Sep 2015	52 (South Africa, Philippines, Georgia, Russia, Latvia, Peru, India, Haiti, Pakistan, Belarus, Brazil, Thailand, Mexico, Bulgaria, Argentina, Ecuador)	MDR-TB +HIV	Prevalence (pooled individuals - no meta-analysis)	Low
Chem 2019 [S5]	2004 to May 2018; English language, in SSA countries	9 / 9 (South Africa, Lesotho, Botswana, Ethiopia)	MDR-TB +HIV	OR of treatment success and prevalence of death and cured. Prevalence*	Critically low
Edessa 2020 [S6]	up to Feb 2020; English language, in SSA countries	19/19 (South Africa, Lesotho, Ethiopia, Kenya, Tanzania, Niger, Botswana)	DR-TB +HIV	RR of unfavourable outcome (Failed treatment/Lost from treatment/died), death, treatment failure and loss to follow-up	High
Endalamaw 2019 [S7]	2003-2018; English language, in Ethiopia	13/13 (Ethiopia)	TB +HIV ; PTB +HIV	Prevalence	Moderate
Eshetie 2018 [S2]	up to 2017; English language, in Ethiopia	34 /34 (Ethiopia)	TB +HIV	Prevalence of successful and unsuccessful treatment	Critically low
Gao 2010 [S8]	up to Apr 2010; English or Chinese language, in mainland China	29/29 (China)	TB +HIV	Prevalence (also by sex)	Critically low
Gao 2013 [S9]	up to Dec 2011; English language, any country except China	31/47 (Brazil, Nigeria, Ethiopia, India, Iran, South Africa, Zambia, Zimbabwe, Cambodia, Tanzania, Thailand, Togo, Ukraine, Vietnam)	TB +HIV	Prevalence	Critically low
Gelaw 2019 [S10]	up to Sep 2017; English language in SSA countries	68/68 (Ethiopia, Tanzania, Kenya, Eritrea, Uganda, South Africa, Zambia, Zimbabwe, Angola, Malawi, Nigeria, Cote d'Ivoire, Ghana, Burkina Faso, Togo, Cameroon, Republic of Congo)	TB +HIV	Prevalence (in SSA and in the central, southern, western and eastern regions of SSA)	High
Huddart 2020 [S1]	2006 to Jan 2019; in India	212 / 212 (India)	TB +HIV	Case-fatality rate (during treatment and after treatment)	Critically low
Lukoye 2015 [S26]	2003 to 2013; SSA countries only	27/27 (SSA)	TB +HIV	Prevalence*	Critically low
McMurry 2019 [S27]	1990 to 2016.; English language, LMIC only	84/84 (India, China, Mexico, Tanzania, Ethiopia, Malaysia, Pakistan, Brazil, Nigeria, South Africa, Bangladesh, Indonesia, Marshall Islands, Turkey, Benin, Fiji, Georgia, Guinea, Guinea-Bissau, Guyana, Iran, Kazakhstan, Kiribati, Kyrgystan, Micronesia, Peru, Philippines, Sri Lanka, Thailand, Zambia)	TB +HIV ; TB +DM	Prevalence*	Low
Mekonnen 2019 [S11]	up to Mar 2018; English language, in African countries	28 / 28 (Ethiopia, Zambia, South Africa, Nigeria, Burkina Faso, Uganda, Djibouti, Mozambique, Sudan, Tunisia, Tanzania, Malawi)	TB lymphadenitis +HIV	Prevalence	High
Mesfin 2014 [S49]	up to April 2012.; English language	4/24 (Ucraine, Haiti, Georgia, South Africa)	MDR-TB +HIV	Prevalence*, OR*	Critically low
Pormohammad 2018 [S52]	1985 to Mar 2018	16 / 20 (Dominican Republic, South Africa, Indonesia, China, Vietnam, Turkey, India, Brazil, Peru, Zambia)	TB Meningitis +HIV	Prevalence*	Critically low

For peer review only

Pourakbari 2019 [S28]	up to Apr 2017; Persian and English languages, Iran only	48/48 (Iran)	TB +HIV	Prevalence*	Critically low
Purmohamad 2020 [S53]	2000 to Jan 2017; English language	22/26 (South Africa, Turkey, China, India, Egypt, Brazil, Peru, Vietnam, Indonesia)	TB Meningitis +HIV	Prevalence*	Critically low
Rajendran 2020 [S29]	2009 to Dec 2018; English language, Malaysia only	23/23 (Malaysia)	TB +HIV; TB +DM	Prevalence*	Critically low
Reddy 2010 [S30]	up to Jun 2009; African countries only	22 / 22 but only 5 provided data on TB (Tanzania, Malawi, Uganda, and Cote d'Ivoire)	TB +HIV	Prevalence (pooled individuals - no meta-analysis)	Critically low
Samuels 2018 [S12]	1980 to Jun 2016; English, French and Spanish language	39 / 48 (Ethiopia, Georgia, Russia, Latvia, South Africa, India, Cameroon, Nigeria, Pakistan, Vietnam, Uzbekistan, Moldovia, Belarus, Peru, China, Turkey, Haiti, Philippines,)	MDR/XDR-TB +HIV; MDR/XDR-TB +DM	RR of unsuccessful treatment (composite of failure, death, and default) and treatment failure. Prevalence*	Low
Seid 2018 [S31]	up to Mar 2017; English language, Ethiopia only	34 / 34 (Ethiopia)	TB +HIV	Prevalence*	Critically low
Sotgiu 2009 [S47]	2006 to Dec 2008.; English language	5 / 13 (South Africa, Russia, Peru)	MDR-TB +HIV; XDR-TB +HIV	Mortality*, Treatment outcomes*	Critically low
Straetemans 2011 [S32]	up to Mar 2011; English language	50 / 70 (studies with TB + HIV: 14 / 22) (South Africa, Ivory Coast, Uganda, Somalia, Iran, Malawi, Thailand, Zambia, Mexico, Russia, Sudan, Cambodia, Central African Republic, Guinea-Bissau, Nepal, Ivory Coast, India, Burkina Faso, Cameroon, Kenya, Zaire, China, Vietnam)	TB +HIV	Mortality*	Critically low
Tesfaye 2018 [S33]	2007 to 2016; English language, Ethiopia only	21/21 (15 for prevalence data) (Ethiopia)	TB +HIV	Prevalence*	Critically low
Teweldemedhin 2018 [S34]	1995 to Nov 2017; English language, Ethiopia only	30/30, but only 19/30 determined HIV infection among TB patients (Ethiopia)	TB +HIV	Prevalence*	Critically low
Uchida 2019 [S35]	1919 to 2017; English language	2/7 (India, Nigeria)	TB +HIV	Treatment outcomes*	Critically low
Waitt 2011 [S36]	1966 to 2010; English language	40 / 62 (China, Sudan, Brazil, Vietnam, South Africa, Gambia, Malawi, India, Russia, Thailand, Tanzania, Guinea Bissau, Peru, Mexico, Zambia, Uganda, Bolivia)	TB +HIV; TB +Non-infective comorbidities	Mortality*	Critically low
Wang 2019 [S13]	up to May 2018; English language	21 / 22 (Turkey, India, China, Vietnam, South Africa, Indonesia, Malaysia, Madagascar)	TB meningitis +HIV	Prevalence and prevalence of death	Critically low
Wu 2016 [S50]	up to Oct 2012; English or Chinese language	23/39 (Peru, India, Bangladesh, Lesotho, Iran, China, Latvia, Russia, Uzbekistan, Turkey, South Africa)	MDR-TB +HIV	Prevalence*	Critically low
**TB + DM**					
Alebel 2019 [S14]	up to Sep 2017; English language, in SSA countries	16/16 (Benin, Tanzania, Guinea-Bissau, Uganda, Nigeria, Ethiopia, Guinea, Madagascar, Kenya, Cameroon)	TB +DM	Prevalence	Low
Almeida 2018 [S51]	up to Nov 2015	2/11 (Iran, China)	PTB +DM	Mortality*	Critically low
Baker 2011 [S37]	1980 to Dec 2010	12 / 33 (Indonesia, Thailand, India, Turkey, Iran, Russia, Tunisia, Republic of the Congo, Mexico, China)	TB +DM	Prevalence*, Mortality*, Treatment outcomes*	Critically low
Chen 2013 [S15]	2000-Apr 2013; in China	22/22 (China)	PTB +DM	Prevalence	Critically low
Gautam 2021 [S4]	1980 to Jul 2020; English language, in Afghanistan, Bangladesh, Bhutan, India, Maldives, Nepal, Pakistan, Sri Lanka	65/ 74 (India, Pakistan, Nepal, Bangladesh, and Sri-Lanka)	TB +DM	Prevalence (also by countries), OR of mortality, treatment failure, culture conversion, recurrence, and MDR-TB	Low

For peer review only - http://bmjopen.bmj.com/site/about/guidelines.xhtml

Han 2016 [S38]	1980 to Jul 2015; English language	27/54 (total articles included), 27/33 (articles used for the meta-analysis) (Congo, Tunisia, Russia, Turkey, Thailand, China, India, Mexico, Iran, Kiribati, Brazil, Malaysia, Tanzania, Peru, Indonesia)	TB +DM	Mortality*, Treatment outcomes*	Critically low
Huang 2020 [S39]	1966 to Jul 2019; English only	10/13 (India, China, Mexico, Thailand, Georgia, Iran)	TB +DM	Prevalence*	Critically low
Huangfu 2019 [S16]	1980 to Jul 2018	57/104 (Congo, Indonesia, Iran, Thailand, Tunisia, Turkey, Brazil, India, Mexico, China, Malaysia, Fiji, Kiribati, Ethiopia, Argentina, Poland, Tanzania, Russia, Egypt, Uganda, Georgia, Saudi Arabia, Senegal)	TB +DM	OR of death only and treatment failure and death	Low
Jeon 2010 [S40]	up to May 2009 (databases), 2007 to 2008 (World Lung Conferences abstracts)	16 / 18 studies that met our inclusion criteria on screening for DM among patients with TB (32 studies included in total) (India, Russia, Nigeria, Guinea, Pakistan, Turkey, Indonesia, Tanzania, Mexico, Iran)	TB +DM	Prevalence*, RR*	Critically low
Lutfiana 2019 [S41]	2012 to Sep 2017; English language	32/41 (South Africa, China, India, Thailand, Bangladesh, Georgia, Brazil, Tanzania, Vietnam, Mongolia)	TB +DM	Prevalence*	Critically low
Noubiap 2019 [S17]	1986 to Jun 2017	138/ 200 (Benin, Ethiopia, Guinea-Bissau, Nigeria, Senegal, South Africa, Tanzania, Uganda, Georgia, Kazakhstan, Romania, Turkey, Egypt, Iran, Libya, Pakistan, Tunisia, Yemen, Guyana, Mexico, Brazil, Peru, Bangladesh, India, Sri Lanka, China, Fiji, Indonesia, Kiribati, Malaysia, Marshall Islands, Thailand)	TB +DM	Prevalence (by income level, regions and countries)	Critically low
Shao-hua 2016 [S18]	up to Nov 2015; in China	13 / 13 (China)	PTB +DM	OR and aOR of adverse outcomes (failure to retreatment, death, and loss)	Critically low
Tegegne 2018 [S42]	up to Jul 2018; English language	17 / 25 (Iran, Georgia, Mexico, Egypt, Thailand, Peru, China, Indonesia, Bangladesh, Turkey)	TB +DM	Prevalence*	High
Workneh 2017 [S43]	up to Mar 2016; English language	80/94 (India, China, Iran, Indonesia, Pakistan, Sri Lanka, Nepal, Thailand, Georgia, Philippines, Malaysia, Benin, Ethiopia, Tanzania, Guinea, Kenya, Ethiopia, Uganda, Nigeria, South Africa, Madagascar, Mexico, Peru, Brazil, Kiribati, Marshall Islands, Guyana, Fiji)	TB +DM	Prevalence*	Low
**TB + Mental disorders**					
Alene 2018 [S19]	up to September 2017	37/40 (1 studies on Canada and 2 on South Korea) (China, Peru, Russia, Argentina, Turkey, Iran, Lesotho, Latvia, South Africa, Tanzania, Haiti, Pakistan, Ethiopia, Vietnam, Indonesia, Nigeria, Namibia)	MDR-TB +Depression; MDR-TB +Anxiety; MDR-TB +Psychosis	Prevalence (also by regions)	Critically low
Duko 2020 [S20]	up to Dec 2019; English language	25/25 (Pakistan, Turkey, India, Brazil, China, Nigeria, Cameroon, Ethiopia)	TB +Depression; MDR-TB +Depression	Prevalence (also by sex)	High
Lee 2020 [S21]	1990 to Oct 2018; English, French, Spanish, Portuguese, and Korean languages	10/10 (South Africa, Ethiopia, Zimbabwe, Zambia, Tanzania, Peru, China)	TB +Mental Disorders	OR of poor TB treatment outcomes, loss to follow-up and non-adherence to treatment. Prevalence*, Mortality*	Critically low
Rensburg 2020 [S44]	2000 to 2019; English language	100/100 (Pakistan, South Africa, Peru, Pakistan, China, Ethiopia, Thailand, India, Sudan, Cameroon, Kazakhstan, Sri Lanka, Nigeria, Zambia, Russia, Brazil, Poland, Burkina Faso, Estonia, Angola, Romania, Ukraine)	TB +Mental illness (Depression, Anxiety, Alcohol use, and General Mental health)	Prevalence*	Critically low

For peer review only - http://bmjopen.bmj.com/site/about/guidelines.xhtml

Ruiz-Grosso 2020 [S3]	up to Aug 2019; English language	8/8 (South Africa, Peru, Ethiopia, China, Zimbabwe, Zambia, Tanzania)	TB +Depression	OR of negative outcomes (death and loss to follow-up), death, loss to follow-up and non-adherence	Critically low
**TB + HCV**					
Behzadifar 2019 [S22]	2000 to Mar 2018; English language	13/21 (Georgia, Argentina, Iran, Brazil, Egypt, Pakistan, China, Sudan, Iraq)	TB +HCV	Prevalence	Moderate
**TB + other**					
Basham 2020 [S45]	up to Jan 2020 (databases), 2013 to Dec 2019 (The International Journal of Tuberculosis and Lung Disease); English language	5/16 (Tanzania, Egypt, Peru, Russia, Estonia)	TB + cardiovascular disease	OR*, Mortality*	Critically low
Leung 2020 [S23]	up to Jun 2019; any language	14/47 (Lithuania, China, Czech Republic)	TB +Lung cancer; TB +non-Hodgkin's lymphoma; TB +Leukaemia	RR of lung cancer, non-Hodgkin's lymphoma, and leukaemia	Low
Rehm 2009 [S46]	up to Sep 2008; English language	14/53 (Russia, India , Brazil, Belarus, Kazakhstan, Romania, Slovenia)	TB+AUD (Alcohol Use Disorder)	Prevalence*	Critically low

Notes: aOR: adjsuted OR; DM: Diabetes Mellitus; DR-TB: Drug resistant TB; HCV: Hepatitis C Virus; HIV: Human immunodeficiency virus; LMIC: Low- and middle-income countries; MDR-TB: Multidrug resistant TB; OR: Odds Ratio; PTB: Pulmonary TB; RR: Relative Risk; SSA: Sub-Saharan Africa; Tuberculosis; XDR-TB: Extensively drug-resistant TB.

* Outcomes available for individual studies, but not pooled

For peer review only - http://bmjopen.bmj.com/site/about/guidelines.xhtml

Supplementary Table 4: Outcomes reported by each systematic review

Lead author and year	Clusters covered	Outcomes	Quality rating
**TB+HIV**			
Alemu 2020	TB +HIV	Outcomes available for individual studies, but not pooled: Prevalence*: range 7.1% - 30.4%	Low
Arega 2020	TB +HIV	Outcomes available for individual studies, but not pooled: Prevalence*: range 1.70%-45.80%	Critically low
Bastos 2019	TB +HIV	Outcomes available for individual studies, but not pooled: Mortality*: range 3.6% - 30.9%, Treatment outcomes*: cure (33% - 62%), abandonment of treatment (4.2% - 13.6%)	Critically low
Bisson 2020	MDR-TB +HIV	Low and lower-middle income countries: 5.1% (130 of (2421+130) pooled individuals from two studies) - no MA<br>Upper-middle: 3585/(3585+3244)= 52.5% (pooled individuals from two studies) - no MA	Low
Chem 2019	MDR-TB +HIV	Successful treatment: OR 0.87 (0.79 - 0.96), 6 studies, number of participants NR, I^2 NR, range 0.75 - 1.26)<br>Mortality: 18% (14%-23%, 9 studies, number of participants NR, I^2=91.1%, range=9%-31%)<br>Cured: 34% (22%-45%, 9 studies, number of participants NR, I^2=98.9%, range= 3%-60%)<br>Outcomes available for individual studies, but not pooled: Prevalence*: 21.73% - 100%	Critically low
Edessa 2020	DR-TB +HIV	Unsuccessful treatment: † RR 1.18 (1.07-1.30, 19 studies, 8301 participants, I^2=48%, range=0.71-2.37)<br>Unsuccessful treatment (western SSA region) : RR 1.42 (0.95-2.13, 2 studies, 790 participants, I^2=12%, range=1.31-2.37)<br>Unsuccessful treatment (eastern SSA region): RR 1.47 (95% CI: 1.23–1.75, 6 studies, 1970 participants, I^2=0%, range=1.14-1.77)<br>Unsuccessful treatment (southern SSA region): † RR 1.09 (0.98-1.20, 11 studies, 5541 participants, I^2=43%, range=0.71-1.41)<br><br>Mortality: † RR 1.50 (1.30-1.74, 16 studies, 7365 participants, I^2=39%, range=0.73-2.18)<br>Mortality (western SSA region) : RR 1.42 (0.96-2.09, 1 study, 588 participants)<br>Mortality (eastern SSA region): RR 1.52 (95% CI: 1.19–1.93, 5 studies, 1442 participants, ,I^2=0%, range=1.20-2.18)<br>Mortality (southern SSA region): † RR 1.49 (1.21-1.83, 10 studies, 5335 participants, I^2=60%, range=0.73-1.47)<br><br>Treatment failure: † RR 0.66 (0.38-1.13, 10 studies, 5474 participants, I^2=73%, range=0.15-2.40)<br>Loss to follow up: † RR 0.82 (0.74-0.92, 14 studies, 7051 participants, I^2=0%, range=0.49-2.61)	High
Endalamaw 2019	TB +HIV; PTB +HIV	Prevalence: 23.40% (95% CI 19.56%-27.24%, 13 studies, 19212 participants, I^2=97.6%, range=9.50%-52.10%)<br>PTB. 22.08% (95% CI 14.36%-29.81%, 3 studies, 1079 participants, I^2=89.9%, range 14.97%-28.60%)	Moderate
Eshetie 2018	TB +HIV	Successful treatment: prev 67% (56%-79%, number of studies NR, number of participants NR, I^2 NR, range NR)<br>Unsuccessful treatment: prev 33% (21%-44%, number of studies NR, number of participants NR, I^2 NR, range NR)<br>Unsuccessful treatment: OR (TB+HIV vs TB) 1.98 (1.56-2.52, 20 studies, number of participants NR, I^2 =81.0%, range 0.82-14.31)	Critically low
Gao 2010	TB +HIV	Prevalence: 0.9% (95% CI 0.6%–1.4%, 18 studies, number of participants NR, I^2= 92.21, range 0.1%–4.5%)<br>Men: 1.1% (95% CI 0.6% - 2.0%, 9 studies, number of participants NR, I^2=94.7%)<br>Women: 0.6% (95% CI 0.3% - 1.1%, 9 studies, number of participants NR, I^2=71.8%)	Critically low
Gao 2013	TB +HIV	Africa: 31.2% (95% CI 19.3% - 43.2%), 17 studies, number of participants NR, I^2=99.6%, range NR)<br>Latin America: 25% (95% CI 19.3% - 30.8%), 7 studies, number of participants NR, I^2=95.2%, range NR)	Critically low
Gelaw 2019	TB +HIV	SSA: prevalence 31.81% (95% CI 27.83%-36.07%; 68 studies, 62696 participants, I^2=98%, range=6.03%-72.25%)<br>Eastern region (SSA): prev. 31.14% (95% CI 25.39%-37.54%, 32 studies, 33637 participants, I^2=98%, range=6.03%-60.51%)<br>Western region (SSA): prev. 25.48% (95% CI 19.70%-32.27%), 21 studies, 16145 participants, I^2=98%, range=10.26%-72.13%)<br>Southern region (SSA): prev. 43.67% (95% CI 35.05%-52.69%, 12 studies, 11148 subject, I^2=99%, range=23.84%-72.25%)<br>Central region (SSA): prev. 41.33% (95% CI 30.39%-53.19%, 3 studies, 2039 participants, I^2=96%, range=31.29%-51.56%)	High
Huddart 2020	TB +HIV	Case-fatality rate (during treatment): 10.91% (7.68%-15.50%), 35 studies, number of participants NR, Tau^2=0.90 (considered low heterogeneity if <4, according to the authors)<br>Case-fatality rate (after treatment): 4.15% (1.06% to 16.24%), 5 studies, number of participants NR, Tau2=1.902 (considered low heterogeneity if <4, according to the authors)	Critically low

For peer review only - http://bmjopen.bmj.com/site/about/guidelines.xhtml

Lukoye 2015	TB +HIV	Outcomes available for individual studies, but not pooled: Prevalence*: range 25.4% - 79.9%	Critically low
McMurry 2018	TB +HIV; TB +DM	Outcomes available for individual studies, but not pooled: Prevalence*: range 1.9% - 45%	Low
Mekonnen 2019	TB lymphadenitis +HIV	Africa: 52% (95% CI 33%-71%, 14 studies, number of participants NR, I^2=99.2%, range 6%-91%)<br>Ethiopia: 21% (95% CI 12%-30%, 6 studies, number of participants NR, I^2=92.9%, range 6%-67%)	High
Mesfin 2014	MDR-TB +HIV	Outcomes available for individual studies, but not pooled: Prevalence*: range 3.4% - 31.6%, OR*	Critically low
Pormohammad 2018	TB Meningitis +HIV	Outcomes available for individual studies, but not pooled: Prevalence*: range 0% - 100%	Critically low
Pourakbari 2019	TB +HIV	Outcomes available for individual studies, but not pooled: Prevalence*: range 0.4% - 38%	Critically low
Purmohamad 2020	TB meningitis +HIV	Outcomes available for individual studies, but not pooled: Prevalence*: range 0% - 91%	Critically low
Rajendran 2020	TB +HIV; TB +DM	Outcomes available for individual studies, but not pooled: Prevalence*: MDR-TB+DM: 26.7% (1 study); MDR-TB+HIV: 17.6% (1 study)	Critically low
Reddy 2010	TB +HIV	161 / 166 (97%) – no meta-analysis	Critically low
Samuels 2018	MDR/XDR-TB +HIV ; MDR/XDR-TB +DM	Unsuccessful treatment:<br>MDR/XDR-TB + HIV (low gross domestic product countries): RR 2.23 (1.60-3.11, 7 studies, 2662 participants, I^2=41%, range=0.67-3.33)<br>MDR/XDR-TB + HIV (LMIC): RR 1.34 (1.04-1.72, 13 studies, 5816 participants, I^2=88%, range=0.55-3.33)<br>MDR/XDR-TB + DM (vs MDR/XDR-TB only): RR 0.90 (0.65-1.23, 3 studies, 687 participants, I^2=19%, range=0.23-0.98)<br><br>Treatment Failure (defined as 5 cultures positive within the last 12 months of therapy or any culture positivity within the last 3 cultures; alternatively, failure was defined as treatment discontinuation due to lack of appropriate response or significant adverse events):<br>MDR/XDR-TB + HIV (vs MDR/XDR-TB only): RR 0.75 (0.44-129, 7 studies, 5930 participants, I^2=55%, range=0.32-2.40)<br>Outcomes available for individual studies, but not pooled: Prevalence*:	Low
Seid 2018	TB +HIV	Outcomes available for individual studies, but not pooled: Prevalence*: range 2.0% - 100%	Critically low
Sotgiu 2009	MDR-TB +HIV; XDR-TB +HIV	Outcomes available for individual studies, but not pooled: Mortality*: , Treatment outcomes*:	Critically low
Straetemans 2011	TB +HIV	Outcomes available for individual studies, but not pooled: Mortality*: range 2.2% - 34.4%	Critically low
Tesfaye 2018	TB +HIV	Outcomes available for individual studies, but not pooled: Prevalence*: range 11.4% - 36.2%	Critically low
Teweldemedhin 2018	TB +HIV	Outcomes available for individual studies, but not pooled: Prevalence*: range 6%-52.1%	Critically low
Uchida 2019	TB +HIV	Outcomes available for individual studies, but not pooled: Treatment outcomes*: Unsuccessful outcomes (death, failure, loss to follow-up and transferred-out HIV positive): aOR 3.6 (95%CI 1.1-11.7) (1 study)	Critically low
Waitt 2011	TB +HIV; TB + Non-infective comorbidities	Outcomes available for individual studies, but not pooled: Mortality*:	Critically low
Wang 2019	TB meningitis +HIV	Prevalence: 10.6% (95% CI: 4.2%–24.6%, number of studies NR, number of participants NR, I^2 NR, range NR)<br>Mortality: 53.4% (42.4%–64.1%, 7 studies, 547 participants, I^2= 2.1%, range NR)	Critically low
Wu 2016	MDR-TB +HIV	Outcomes available for individual studies, but not pooled: Prevalence*:	Critically low
**TB+DM**			

For peer review only

Alebel 2019	TB +DM	Prevalence TB+DM: 9% (95% CI 6%-12%, 16 studies, 13286 participants, I^2= 97.48%, range: 2%-38%)<br>Nigeria: 15% (95% CI 7%-23%, 4 studies, 4998 participants, I^2 NR, range NR)<br>Ethiopia: 10% (95% CI 6%-13%, 3 studies, 1633 participants, I^2 NR, range NR)<br>Tanzania: 11% (95% CI 9%-12%, 2 studies, 1309 participants, I^2 NR, range NR)	Low
Almeida 2018	PTB +DM	Outcomes available for individual studies, but not pooled: Mortality*: OR 9.70 (95% CI 2.92-32.22)	Critically low
Baker 2011	TB +DM	Outcomes available for individual studies, but not pooled: Prevalence*: , Mortality*: range of RR 1.26 - 28.47, Treatment outcomes*: relapse: range of RR 1.88 - 5.96; remaining sputum culture positive: range of RR 0.79 - 2.17, failure/death: range of RR 1.44 - 3.13	Critically low
Chen 2013	PTB +DM	Prev: 7.20% (95% CI 6.01%-8.39%, 22 studies, 56805 participants, I^2 NR, range 2.08%-16.16%)	Critically low
Gautam 2021	TB +DM	Pooled prevalence 21% (95% CI 18%-23%, 65 studies, 49,792 patients, I^2=98.28%, range NR)<br>MDR-TB OR 1.05 (95% CI 0.63-1.74, 4 studies, number of participants NR, I^2=40,71%, range NR)<br>Among adults only: 21.0% (95% CI 18.0–23.0%, 55 studies, number of participants NR, I^2= 97.99%, range NR)<br><br>Bangladesh prevalence 11.0% (95% CI 10.0%–12.0%, 2 studies, number of participants NR, I^2 NR, range NR)<br>India prevalence 22.0% (95% CI 19.0%–25.0%, 47 studies, number of participants NR, I^2=97.92%, range NR)<br>Nepal prevalence 12.0% (95% CI 4.0%–20.0%, 4 studies, number of participants NR, I^2=96.70%, range NR)<br>Pakistan prevalence 19.0% (95% CI 11.0%–27.0%, 10 studies, number of participants NR, I^2=99.18%, range NR)<br>Sri Lanka prevalence 24.0% (95% CI 21.0%–27.0%, 2 studies, number of participants NR, I^2 NR, range NR)<br>Mortality in TB+DM vs TB: OR 1.74 (1.21-2.51, 5 studies, number of participants NR, I^2=19.43%, range 0.14-1.95)<br>Treatment failure: OR 1.65 (1.12-2.44, 5 studies, number of participants NR, I^2=49.63%, range 1.34-21.91)<br>Cured: OR 0.32, 95% CI 0.10 - 1.05, 1 study)<br>Recurrence: OR 0.53 (95% CI 0.32, 0.87, 1 study)<br>MDR-TB: OR 1.05 (0.63-1.74, 4 studies, number of participants NR, I^2=40.71%, range=0.45-4.70)	Low
Han 2016	TB +DM	Outcomes available for individual studies, but not pooled: Mortality*: range of OR 0.41 - 29.22, Treatment outcomes*: Sputum culture conversion at 2 to 3 months: range of OR 0.57 - 5.27; Failure/death: range of OR 0.86 - 18.91; Relapse: range of OR 0.97 - 6.35	Critically low
Huang 2020	TB +DM	Outcomes available for individual studies, but not pooled: Prevalence*: range 9% - 49%	Critically low
Huangfu 2019	TB +DM	Mortality: OR 1.80 (95%CI 1.35–2.40; 32 studies, number of participants NR, I^2=91%, range NR)<br>Treatment failure or death: OR 1.90 (95%CI 1.43–2.53; 22 studies, number of participants NR, I^2=87.3%, range NR)	Low
Jeon 2010	TB +DM	Outcomes available for individual studies, but not pooled: Prevalence*: , RR*	Critically low
Lutfiana 2019	TB +DM	Outcomes available for individual studies, but not pooled: Prevalence*: range 3.3%-100%	Critically low
Noubiap 2019	TB +DM	Prevalence:<br>Low income countries: 7.9% (95% CI 4.9%-11.5%, 15 studies, 9434 participants, I^2=96.8%, range NR)<br>Lower-middle income: 17.7% (95% CI 15.1%-20.5%, 48 studies, 48036 participants, I^2=98.3%, range NR)<br>Upper-middle income: 14.4% (95% CI 12.8%-16.0%, 75 studies, 1,994,027 participants, I^2=99.9%, range NR)<br><br>African region: 8.0% (95% CI 5.9%-10.4%, 119 studies, 474,944 participants, I^2=99.8%, range 1.9%-32.4%)<br>Southeast Asia: 19.0% (95% CI 16.2%-21.9%, 30 studies, 30382 participants, I^2=97.0%, range 5.1%-54.1%)<br><br>Benin: 1.9% (95% CI 0.2%-4.7%, 1 study, 159 participants)<br>Ethiopia: 18.8% (95% CI 1.9%-47.1%, 2 studies, 1749 participants, I^2=99.2%, range: 8.3%-32.4%)<br>Guinea-Bissau: 2.7% (95% CI 0.3%-6.8%, 1 study, 110 participants)<br>Nigeria: 7.8% (95% CI 4.4%-12.0%, 4 studies, 9821 participants, I^2=97.8%, range=4.8%-12.0%)<br>Senegal: 4.9% (95% CI 2.2%-8.5%, 2 studies, 2848 participants, I^2=75.1%, range=3.8%-7.0%)<br>South Africa: 9.4% (95% CI 7.6%-11.3%, 1 study, 947 participants)<br>Tanzania: 8.5% (95% CI 4.8%-13.0%, 7 studies, 4178 participants, I^2=95.1%, range=2.6%-16.7%)	Critically low

For peer review only - http://bmjopen.bmj.com/site/about/guidelines.xhtml

		Uganda: 7.3% (95% CI 4.7%-10.3%, 2 studies, 390 participants, I^2=9.9%, range=5.4%-8.5%)<br><br>Kazakhstan: 7.1% (95% CI 5.1%-9.4%, 1 study, 562 participants)<br>Romania: 18.4% (95% CI 13.6%-23.7%, 1 study, 228 participants)<br>Turkey: 7.8% (95% CI 6.8%-8.8%, 3 studies, 2773 participants, I2=0%, range=7.9%-8.6%)<br>Georgia: 12.4% (95% CI 7.4%-18.5%, 1 study, 137 participants)<br><br>Egypt: 22.8% (95% CI 15.2%-31.4%, 3 studies, 578 participants, I2=81.4%, range=15.8%-27.7%)<br>Iran: 17.8% (95% CI 12.5%-23.8%, 11 studies, 3134 participants, I2=93.3%, range=5.5%-40.0%)<br>Libya: 6.1% (95% CI 3.5%-9.4%, 1 study, 262 participants)<br>Pakistan: 22.0% (95% CI 12.8%-32.8%, 6 studies, 5201 participants, I2=98.8%, range=11.4%-39.6%)<br>Tunisia: 7.6% (95% CI 5.9%-9.6%, 1 study, 788 subject)<br>Yemen: 9.5% (95% CI 6.0%-13.8%, 1 study, 220 participants)<br><br>Guyana: 14.0% (95% CI 7.8%-21.6%, 1 study, 100 participants)<br>Mexico: 30.8% (95% CI 26.4%-35.3%, 10 studies, 192420 participants, I2=97.9%, range=19.3%-54.4%)<br><br>Brazil: 7.2% (95% CI 6.3%-8.1%, 12 study, 1726436 participants, I2=99.7%, range=3.3%-33.1%)<br>Peru: 4.8% (95% CI 1.7%-9.5%, 4 studies, 3983 participants, I2=96.8%, range=2.5%-11.1%)<br><br>Bangladesh: 10.6% (95% CI 7.2%-14.5%, 3 studies, 3010 participants, I2=85.9%, range=8.3%-12.8%)<br>India: 19.9% (95% CI 16.8%-23.2%, 26 studies, 27260 participants, I2=97.2%, range=5.1%-54.1%)<br>Sri Lanka: 24.1% (95% CI 16.6%-32.5%, 1 study, 112 participants)<br><br>China: 14.5% (95% CI 10.5%-19.0%, 14 studies, 19529 participants, I2=98.4%, range=2.7%-30.1%)<br>Fiji: 10.1% (95% CI 4.4%-17.7%, 3 studies, 1139 participants, I2=91.8%, range=5.2%-13.7%)<br>Indonesia: 14.8% (95% CI 12.2%-17.7%, 1 study, 634 participants)<br>Kiribati: 36.7% (95% CI 31.1%-42.5%, 1 study, 275 participants)<br>Malaysia: 26.9% (95% CI 17.8%-37.0%, 5 studies, 23438 participants)<br>Marshall Island: 45.2% (95% CI 32.9%-57.7%, 1 study, 62 participants)<br>Thailand: 7.5% (95% CI 6.2%-8.8%, 5 studies, 17862 participants, I2=81.6%, range=6.0%-16.3%)	
Shao-hua 2016	PTB +DM	Retreatment:<br>OR 2.05 (1.30-3.22, 3 studies, 499 participants, I^2=0%, range NR)<br>aOR 3.38 (1.56-7.29, 2 studies, n participants NR, I^2=75%, range NR)	Critically low
Tegegne 2018	TB +DM	Outcomes available for individual studies, but not pooled: Prevalence*: range 5% - 36%	High
Workneh 2017	TB +DM	Outcomes available for individual studies, but not pooled: Prevalence*: range 1.9% - 45%	Low
**TB+Mental disorders**			
Alene 2018	MDR-TB + Depression; MDR-TB +Anxiety; MDR-TB +Psychosis	Depression:<br>Overall: Prev. 25% (95% CI 14%-39%, 15 studies, n participants NR, I^2=98%, range= 3%-79%)<br>African region: 16% (95% CI 9%-24%, 3 studies, n participants NR, I^2 NR, range NR)<br>The Americas Region: 36% (95% CI 23%-50%, 3 studies, n participants NR, I^2 NR, range NR)<br>South-East Asia Region: 22% (95% CI 0%-60%, 3 studies, n participants NR, I^2 NR, range NR)<br>European region: 11% (95% CI 4%-21%, 3 studies, n participants NR, I^2 NR, range NR)<br>Eastern Mediterranean Region: 73% (95% CI 64%-81%, 2 studies, n participants NR, I^2 NR, range NR)<br>Western Pacific Region: 5% (95% CI 1%-12%, 1 study, , n participants NR, I^2 NR, range NR)	Critically low

For peer review only - http://bmjopen.bmj.com/site/about/guidelines.xhtml

		Anxiety:<br>Overall: Prev: 24% (95% CI 2%-57%, 3 studies, n participants NR, I^2=95%, range=12%-56%)<br>The Americas Region: 14% (95% CI 9%-21%, 2 studies, n participants NR, I^2 NR, range NR)<br>South-East Asia Region: 56% (95% CI 45%-66%, 1 studies, n participants NR, I^2 NR, range NR)<br><br>Psychosis:<br>(Overall includes a study from S.Korea)<br>African region: 12% (95% CI 8%-17%, 5 studies, n participants NR, I^2 NR, range NR)<br>The Americas Region: 11% (95% CI 7%-17%, 2 studies, n participants NR, I^2 NR, range NR)<br>South-East Asia Region: 10% (95% CI 5%-17%, 2 studies, n participants NR, I^2 NR, range NR)<br>European region: 6% (95% CI 0%-17%, 2 studies, n participants NR, I^2 NR, range NR)<br>Eastern Mediterranean Region: 7% (95% CI 1%-17%,1 studies, n participants NR, I^2 NR, range NR)	
Duko 2020	TB + Depression; MDR-TB +Depression	Prevalence: 45.19% (95% CI 38.04%-52.55%, 25 studies, 4903 participants, I^2=96.28%, range=15.56%-80.00%)<br>Women: 51.54% (95% CI 40.34%–62.60%, 17 studies, number of participants NR, I^2 = 92. 55%, range NR)<br>Men: 45.25% (95% CI 35.19%–55.71%, 17 studies, number of participants NR, I^2 = 95.09%, range NR)<br>MDR-TB: 52.34% (95% CI 38.09%-66.22%, 5 studies, number of participants NR, I^2=92.55%, range=NR)	High
Lee 2020	TB +Mental Disorders	Unsuccessful treatment: OR 2.13 (95%CI 0.85-5.37, 4 studies, 1196 participants, I^2=82%, range NR)<br>Loss to follow up: OR 1.90 (95%CI 0.33-10.91, 2 studies, 1139 participants, I^2=78%, range NR)<br>Non-adherence to treatment (measured by self-report, missed visits, pill count, or physiological tests): OR 1.60 (95% CI 0.84-3.02, 4 studies, 10851 participants, I^2=86%, range=0.94-3.67)<br>Outcomes available for individual studies, but not pooled: Prevalence*: Depression: range 37.5% - 53.9%; Mental disorder: range 18.9%-22.4%; Psychological distress: range 22% - 67.6%; PTSD: 29.6% (1 study), Mortality*:	Critically low
Rensburg 2020	TB +Mental illness (Depression, Anxiety, Alcohol use, and General Mental health)	Outcomes available for individual studies, but not pooled: Prevalence*: Depression: range 9.3% - 84%; Anxiety: range 2%-47.2%; Alcohol use: range 5% - 63%; Psychiatric comorbidity: range 3%; Psychological distress: range 22% - 83.6%; Poor mental quality: range 13.1% (1 study); Common mental disorder/Mental disorder: range 22.4%-38.3%	Critically low
Ruiz-Grosso 2020	TB +Depression	Mortality or loss to follow-up: OR = 4.26 (95% CI 2.33–7.79, 2 studies, 973 participants, I^2=0%, range=3.65-4.88)<br>Mortality: OR 2.85 (1.52-5.36, 2 studies, 973 participants, I^2=0%, range=1.76-2.99)<br>Loss to follow up: OR 8.70 (4.95-9.09, 2 studies, 973 participants, I^2=0%, range=4.95-9.09)<br>Non-adherence to TB treatment: OR 1.38 (0.70-2.72, 3 studies, 9349 participants, I^2=94.36%, range=0.92-3.67)	Critically low
**TB+HCV**			
Behzadifar 2019	TB +HCV	Africa: 11% (95% CI 1%-23%, 3 studies, 327 participants, I^2=93.9%, range=NR)	Moderate
**TB+other**			
Basham 2020	TB + cardiovascular disease	Outcomes available for individual studies, but not pooled: Mortality*: range of ORs 2.50 - 3.01	Critically low
Leung 2020	TB +Lung cancer; TB +non-Hodgkin's lymphoma; TB +Leukaemia	Upper middle-income countries<br>Lung cancer: RR 1.53 (95% CI 1.25-1.87, 9 studies, number of participants NR, I^2=94.6%, range NR)<br>non-Hodgkin's lymphoma: RR 1.70 (95% CI 1.13-2.56, 1 study, number of participants NR, I^2=NA)<br>leukaemia: RR 1.61 (95% CI 1.13-2.29, 1 study, number of participants NR, I^2= NA)	Low
Rehm 2009	TB+ Alcohol Use Disorder	Outcomes available for individual studies, but not pooled: Prevalence*:	Critically low

For peer review only - http://bmjopen.bmj.com/site/about/guidelines.xhtml

Note: Quality was assessed using the AMSTAR2 tool. aOR: adjusted OR; DM: Diabetes Mellitus; DR-TB: Drug resistant TB; HCV: Hepatitis C Virus; HIV: Human immunodeficiency virus; LMIC: Low- and middle-income countries; MDR-TB: Multidrug resistant TB; NR: Not reported; OR: Odds Ratio; prev.: prevalence; PTB: Pulmonary TB; RR: Relative Risk; SSA: Sub-Saharan Africa; Tuberculosis; XDR-TB: Extensively drug-resistant TB.

* Outcomes available for individual studies, but not pooled. Range of effect estimates reported.

† Includes one study focused on children

For peer review only

For peer review only - http://bmjopen.bmj.com/site/about/guidelines.xhtml

Supplementary Table 5: Conditions that were considered chronic (and therefore included) or not (and therefore excluded) for this review

Condition/risk factor	Should be considered as a comorbidity in this review?	Description or details
**Included**		
Acquired immunodeficiency syndrome (AIDS)	Yes	
Anxiety	Yes	Clinical diagnostic of an anxiety disorder or assessed with a validated scale
Arthritis	Yes	
Asthma	Yes	
Autoimmune diseases	Yes	
Cancer	Yes	Any type of cancer
Cardiovascular disease	Yes	
cerebrovascular accident	Yes	
cerebrovascular accident	Yes	Cerebrovascular disease is a form of cardiovascular disease
chronic airflow obstruction	Yes	As a proxy for COPD
Chronic kidney disease / Chronic renal failure	Yes	No if acute renal failure- Yes if chronic renal failure
Chronic liver disease / Cirrhosis / Chronic hepatic dysfunction	Yes	
Chronic lung disease	Yes	
Chronic obstructive pulmonary disease (COPD)	Yes	COPD is form of chronic lung disease
Cor pulmonale	Yes	
Depression	Yes	Clinical diagnostic or assessed with a validated scale (e.g. PHQ-9)
Diabetes mellitus (DM)	Yes	
hearing defect	Yes	
Heart disease / Cardiopathies	Yes	
Heart failure	Yes	
Hepatitis B virus (HBV)	Yes	
Hepatitis C virus (HCV)	Yes	
HIV	Yes	
hyperthyroidism	Yes	
hypothyroidism	Yes	
Mental disorder	Yes	Umbrella term for conditions such as PTSD, OCD, depression, anxiety disorders, etc.
Obsessive compulsive disorder (OCD)	Yes	
Panic disorder	Yes	

For peer review only

For peer review only - http://bmjopen.bmj.com/site/about/guidelines.xhtml

pneumoconiosis	Yes	
Post-traumatic stress disorder (PTSD)	Yes	
seizures (cause not determined)	Yes if called epilepsy	Yes only if called epilepsy
Substance use / drug abuse	Yes	usually reserved for illicit substances
T. pallidum	Yes	
Unstable angina	Yes	
**Excluded**		
Acute Respiratory Distress Syndrome	No	Acute
Anaemia	No	
Aspergilius coinfection	No	complications of HIV
BMI	No	
Candida coinfection	No	complications of HIV
cardiomyopathy	No	Can be acute and/or reversible
Cavitary disease	No	
Chronic corticosteroid therapy	No	Treatment
Chronic diarrhoea	no	Unless it's inflammatory bowel disorders, no.
cryptoccocus	No	complications of HIV
cryptococcal IRIS (immune reconstitution inflammatory syndrome) adenitis	No	
deep venous thrombosis	NO	
dermatitis	No	
Drinking alcohol	No	No, unless it is something like 'harmful use of alcohol' or 'alcohol dependence'
drug-induced hepatotoxicity/ liver injury	No	
Dyslipidemia	No	
haemorrhoids/fistula-in-ano	No	
HTLV (Human T-Cell LymphotropicVirus)	No	complications of HIV
Hypertension	No	
hypokalaemia	No	
hyponatremia	No	complications of HIV
Intestinal parasites	No	
Leukopenia	no	
Malnourishment	No	
Obesity	No	

For peer review only - http://bmjopen.bmj.com/site/about/guidelines.xhtml

pancreatitis	No	Unless Long term pancreatitis and it is clearly specified
Pneumonia	No	Acute condition
pneumothorax	No	
Pulmonary edema	No	Symptom
pulmonary fungal infection	No	complications of HIV
sacroiliitis	NO	
scabies	No	
severe epistaxis	No	
Skin conditions	no	
smoking	No	Would not fall under ‘substance abuse’, even if it is reported as ‘high dependence smoking’
visual impairment (reported as a symptom of DM)	No (reported as a complication)	

For peer review only

For peer review only - http://bmjopen.bmj.com/site/about/guidelines.xhtml

Supplementary Table 6: AMSTAR2 assessment details for each study

**Study Id**	**1**	**2**	**3**	**4**	**5**	**6**	**7**	**8**	**9**	**10**	**11**	**12**	**13**	**14**	**15**	**16**	**confidence rating**
Duko 2020	Yes	**Partial Yes**	Yes	**Partial Yes**	Yes	Yes	**Partial Yes**	Yes	**Yes**	No	**Yes**	Yes	**Yes**	Yes	**Yes**	Yes	High
Alemu 2020	Yes	**Partial Yes**	Yes	**Partial Yes**	Yes	Yes	**No**	Yes	**Yes**	No	**Yes**	Yes	**Yes**	Yes	**Yes**	Yes	Low
Arega 2020	Yes	**No**		**Partial Yes**	Yes	Yes	**No**	Yes	**Yes**	No	**Yes**	Yes	**Yes**	Yes	**Yes**	Yes	Critically low
Huddart 2020	Yes	**No**	Yes	**Partial Yes**	Yes	Yes	**Partial Yes**	Partial Yes	**No**	No	**No**	Yes	**Yes**	Yes	**No**	Yes	Critically low
Huang 2020	Yes	**Partial Yes**	Yes	**No**	Yes	Yes	**No**	Yes	**Yes**	No	**Yes**	Yes	**Yes**	Yes	**Yes**	Yes	Critically low
Edessa 2020	Yes	**Partial Yes**	Yes	**Partial Yes**	Yes	Yes	**Partial Yes**	Yes	**Partial Yes**	No		Yes	**Yes**	Yes	**Yes**	Yes	High
Basham 2020	Yes	**No**	Yes	**Partial Yes**	Yes	Yes	**No**	Yes	**Partial Yes**	No	**No**	Yes	**Yes**	Yes			Critically low
Bisson 2020	Yes	**Yes**	Yes	**Partial Yes**	No	No	**Partial Yes**	No	**No**	No	**NA**	NA	**NA**	No	**NA**	Yes	Low
Huangfu 2019	Yes	**Partial Yes**	Yes	**Partial Yes**	Yes	Yes	**Partial Yes**	Partial Yes	**Partial Yes**	No	**Yes**	Yes	**Yes**	Yes	**No**	Yes	Low
Bastos 2019	Yes	**Yes**	Yes	**Partial Yes**	Yes	Yes	**No**	Partial Yes	**No**	No	**NA**	NA	**NA**	No	**NA**	Yes	Critically low
Endalamaw 2019		**Partial Yes**	Yes	**Partial Yes**	No	Yes	**Partial Yes**	Partial Yes	**Partial Yes**	No	**Yes**	Yes	**Yes**	No	**Yes**	Yes	Moderate
Chem 2019	Yes	**Partial Yes**	Yes	**Partial Yes**	Yes	No	**Partial Yes**	Yes	**Partial Yes**	Yes	**Yes**	Yes	**No**	Yes	**No**	Yes	Critically low
Alebel 2019	Yes	**Yes**	Yes	**Partial Yes**	No	Yes	**No**	Partial Yes	**Yes**	No	**Yes**	Yes	**Yes**	Yes	**Yes**	Yes	Low
Behzadifar 2019	Yes	**Partial Yes**	Yes	**Partial Yes**	Yes	No	**Partial Yes**	Partial Yes	**Yes**	No	**Yes**	Yes	**Yes**	Yes	**Yes**	Yes	Moderate
Gelaw 2019	Yes	**Partial Yes**	Yes	**Partial Yes**	Yes	Yes	**Partial Yes**	Yes	**Yes**	No	**Yes**	Yes	**Yes**	Yes	**Yes**	Yes	High
Alene 2018		**Partial Yes**	Yes	**Partial Yes**	Yes	Yes	**Partial Yes**	Yes	**No**	No	**No**	No	**No**	No	**No**	Yes	Critically low
Almeida 2018	Yes	**Partial Yes**	Yes	**Partial Yes**	Yes	Yes	**Partial Yes**	Partial Yes	**No**	No	**No**	No	**No**	No	**No**	Yes	Critically low
Eshetie 2018		**No**	Yes	**Partial Yes**	Yes	Yes	**No**	Yes	**Yes**	No	**Yes**	Yes	**Yes**	Yes	**No**	Yes	Critically low
Gao 2013	Yes	**No**	Yes	**Partial Yes**	Yes	Yes	**Partial Yes**	Partial Yes	**No**	No	**Yes**	No	**No**	Yes	**No**	Yes	Critically low
	Yes	**No**		**Partial Yes**	No	No	**Partial Yes**	Partial Yes	**No**	No	**NA**	NA	**NA**	No	**NA**	No	Critically low
Baker 2011	Yes	**No**	Yes	**Partial Yes**	No	Yes	**Partial Yes**	Partial Yes	**No**	No	**Yes**	Yes	**No**	Yes	**Yes**	Yes	Critically low
Jeon 2010	Yes	**No**	Yes	**Partial Yes**	No	Yes	**No**	Yes	**No**	No	**NA**	NA	**NA**	No	**NA**	Yes	Critically low
Gao 2010	Yes	**No**	Yes	**Partial Yes**	No	No	**Yes**	Yes	**No**	No	**No**	No	**No**	Yes	**No**	Yes	Critically low

For peer review only

	Yes	**Partial Yes**	Yes	**Partial Yes**	Yes	Yes	**Partial Yes**	Yes	**No**	No	**No**	No	**No**	Yes	**Yes**	Yes	Critically low
Leung 2020	Yes	**Partial Yes**	Yes	**Partial Yes**	Yes	Yes	**Yes**	Yes	**Partial Yes**	No	**No**	Yes	**Yes**	Yes	**Yes**	Yes	Low
Rensburg 2020	Yes	**No**	Yes	**Partial Yes**	Yes	No	**No**	Partial Yes	**No**	No	**NA**	NA	**NA**	No	**NA**	Yes	Critically low
Han 2016	Yes	**No**	Yes	**Partial Yes**	Yes	Yes	**Partial Yes**	No	**No**	No	**No**	No	**No**	Yes	**Yes**	No	Critically low
Lee 2020	Yes	**Yes**	Yes	**Partial Yes**	Yes	Yes	**Partial Yes**	Yes	**Yes**	No	**Yes**	No	**No**	No	**No**	Yes	Critically low
Purmohamad 2020	Yes	**No**	Yes	**Partial Yes**	Yes	Yes	**Partial Yes**	Partial Yes	**No**	No	**No**	No	**No**	Yes	**Yes**	Yes	Critically low
Rajendran 2020	Yes	**No**	Yes	**Partial Yes**	No	No	**Yes**	No	**No**	No	**NA**	NA	**NA**	No	**NA**	Yes	Critically low
Ruiz-Grosso 2020	Yes	**Partial Yes**	Yes	**No**	Yes	Yes	**No**	No	**No**	No	**Yes**	Yes	**No**	No	**No**	Yes	Critically low
Pourakbari 2019	Yes	**No**	Yes	**Partial Yes**	Yes	Yes	**No**	Yes	**No**	No	**Yes**	No	**Yes**	Yes	**Yes**	Yes	Critically low
Lutfiana 2019	Yes	**No**	Yes	**Partial Yes**	No	No	**Partial Yes**	Partial Yes	**No**	No	**NA**	NA	**NA**	No	**NA**	Yes	Critically low
Wang 2019		**No**	Yes	**Partial Yes**	Yes	Yes	**Partial Yes**	Yes	**No**	No	**Yes**	No	**No**	Yes	**Yes**	Yes	Critically low
Uchida 2019	Yes	**No**	Yes	**No**	Yes	No	**No**	Partial Yes	**No**	No	**NA**	NA	**NA**	No	**NA**	Yes	Critically low
Mekonnen 2019	Yes	**Partial Yes**	Yes	**Partial Yes**	Yes	Yes	**Partial Yes**	Yes	**Partial Yes**	No	**Yes**	Yes	**Yes**	Yes	**Yes**	Yes	High
Teweldemedhin 2018	Yes	**No**	Yes	**No**	Yes	No	**Yes**	Partial Yes	**No**	No	**Yes**	No	**No**	Yes	**Yes**	Yes	Critically low
Tegegne 2018	Yes	**Partial Yes**	Yes	**Partial Yes**	Yes	Yes	**Yes**	Partial Yes	**Yes**	No	**Yes**	Yes	**Yes**	Yes	**Yes**	Yes	High
Tesfaye 2018	Yes	**Partial Yes**	Yes	**Partial Yes**	Yes	Yes	**No**	Partial Yes	**No**	No	**Yes**	Yes	**No**	Yes	**Yes**	Yes	Critically low
Seid 2018	Yes	**No**	Yes	**Partial Yes**	Yes	Yes	**Partial Yes**	Partial Yes	**No**	No	**No**	No	**No**	No	**Yes**	Yes	Critically low
Pormohammad 2018	Yes	**No**	Yes	**Partial Yes**	Yes	Yes	**Partial Yes**	Partial Yes	**No**	No	**No**	Yes	**Yes**	No	**Yes**	Yes	Critically low
Samuels 2018	Yes	**Partial Yes**	Yes	**Partial Yes**	Yes	Yes	**Partial Yes**	Yes	**No**	No	**Yes**	Yes	**Yes**	Yes	**Yes**	Yes	Low
Lukoye 2015	Yes	**No**	Yes	**Partial Yes**	Yes	Yes	**Partial Yes**	Yes	**No**	No	**No**	No	**No**	No	**Yes**	Yes	Critically low
Mesfin 2014	Yes	**No**	Yes	**Partial Yes**	Yes	Yes	**Partial Yes**	Yes	**Partial Yes**	No	**No**	No	**No**	Yes	**Yes**	Yes	Critically low
Straetemans 2011	Yes	**Partial Yes**	Yes	**Partial Yes**	No	No	**Yes**	Partial Yes	**No**	No	**Yes**	No	**No**	Yes	**Yes**	Yes	Critically low
Waitt 2011	Yes	**No**	Yes	**No**	No	No	**Partial Yes**	Partial Yes	**No**	No	**No**	NA	**No**	No	**No**	No	Critically low
Reddy 2010	Yes	**No**	No	**Partial Yes**	Yes	Yes	**Yes**	No	**No**	No	**NA**	NA	**NA**	No	**NA**	Yes	Critically low
Rehm 2009	Yes	**No**	Yes	**Partial Yes**	Yes	No	**No**	Partial Yes	**No**	No	**NA**	NA	**NA**	No	**NA**	Yes	Critically low

Sotgiu 2009	Yes	**No**	Yes	**Partial Yes**	Yes	No	**No**	Partial Yes	**No**	No	**NA**	NA	**NA**	No	**NA**	Yes	Critically low
Noubiap 2019		**Partial Yes**	Yes	**Partial Yes**	Yes	Yes	**Partial Yes**	Partial Yes	**No**	No	**No**	Yes	**Yes**	Yes	**Yes**	Yes	Critically low
Wu 2016	Yes	**No**	Yes	**Partial Yes**	No	No	**Partial Yes**	Yes	**No**	No	**Yes**	Yes	**No**	Yes	**No**	Yes	Critically low
Gautam 2021	Yes	**Partial Yes**	Yes	**Partial Yes**	Yes	Yes	**Yes**	Partial Yes	**Partial Yes**	No	**No**	No	**Yes**	Yes	**Yes**	Yes	Low
Chen 2013	Yes	**No**	Yes	**Partial Yes**	Yes	Yes	**Partial Yes**	Partial Yes	**No**	No	**No**	Yes	**Yes**	Yes	**Yes**	No	Critically low
McMurry 2018	Yes	**Partial Yes**	Yes	**Partial Yes**	**Yes**	No	**Partial Yes**	Partial Yes	**No**	No	**NA**	NA	**NA**	No	**NA**	yes	Low
Workneh 2017	Yes	**Partial Yes**	**Yes**	**Partial Yes**	No	No	**Partial Yes**	**Partial Yes**	**No**	No	**NA**	NA	**NA**	no	**NA**	Yes	Low
Shao-hua 2016	Yes	**No**	Yes	**Partial Yes**	Yes	Yes	**Partial Yes**	Partial Yes	**No**	No	**No**	No	**No**	Yes	**No**	No	Critically low

Note: Items assessing critical domains are bolded.
1. Did the research questions and inclusion criteria for the review include the components of PICO?
2. Did the report of the review contain an explicit statement that the review methods were established prior to conduct of the review and did the report justify any significant deviations from the protocol
3. Did the review authors explain their selection of the study designs for inclusion in the review?
4. Did the review authors use a comprehensive literature search strategy?
5. Did the review authors perform study selection in duplicate?
6. Did the review authors perform data extraction in duplicate?
7. Did the review authors provide a list of excluded studies and justify the exclusions?
8. Did the review authors describe the included studies in adequate detail?
9. Did the review authors use a satisfactory technique for assessing the risk of bias (RoB) in individual studies that were included in the review?
10. Did the review authors report on the sources of funding for the studies included in the review?
11. If meta-analysis was performed did the review authors use appropriate methods for statistical combination of results?
12. If meta-analysis was performed, did the review authors assess the potential impact of RoB in individual studies on the results of the meta-analysis or other evidence synthesis?
13. Did the review authors account for RoB in individual studies when interpreting/discussing the results of the review?
14. Did the review authors provide a satisfactory explanation for, and discussion of, any heterogeneity observed in the results of the review?
15. If they performed quantitative synthesis did the review authors carry out an adequate investigation of publication bias (small study bias) and discuss its likely impact on the results of the review?
16. Did the review authors report any potential sources of conflict of interest, including any funding they received for conducting the review?

For peer review only

Medline (October 23rd 2020)

# ▲	Searches	Results
1	exp Noncommunicable Diseases/ or ((Non-communicable or Noncommunicable or Non-infectious) adj (disease* or condition* or illness*)).mp.	11685
2	exp Chronic Disease/ or ((chronic or long-term) adj (disease* or condition* or illness*)).mp.	343000
3	exp Heart Diseases/ or (heart adj (disease* or disorder* or failure)).mp. or (cardiac adj (disease* or disorder* or failure)).mp.	1252363
4	exp Cardiovascular Diseases/ or (cardiovascular adj (disease* or disorder* or failure)).mp.	2482639
5	exp Coronary Disease/ or (coronary adj (disease* or disorder* or failure)).mp.	224638
6	exp Cerebrovascular Disorders/ or (cerebrovascular adj (disease* or disorder* or insufficienc* or occlusion*)).mp. or (vascular adj (disease* or disorder*)).mp. or (carotid* adj (disease* or disorder*)).mp.	460609
7	exp Peripheral Arterial Disease/ or (arter* adj (disease* or disorder*)).mp.	172699
8	exp Rheumatic Heart Disease/ or exp Heart Defects, Congenital/ or (heart adj3 (malform* or defect* or congeni*)).mp.	182266
9	exp Venous Thrombosis/ or ((deep vein or deep venous) adj thrombos*).mp. or phlebothrombos*.mp.	68358
10	exp Pulmonary Embolism/ or (pulmonar* adj (thromboembolism* or embolism* or disease* or disorder*)).mp.	138141
11	exp Stroke/ or stroke.mp.	320701
12	exp Neoplasms/ or Cancer*.mp. or neoplas*.mp. or tumor*.mp.	4416189
13	exp Lung Diseases/ or exp Respiratory Tract Diseases/ or exp Lung Diseases, Obstructive/ or ((lung* or respiratory or pulmonar* or airflow or airway) adj2 (disease* or obstruct* or hypersensitiv*)).mp. or exp Asthma/ or asthma*.mp. or exp Pulmonary Disease, Chronic Obstructive/ or exp Respiratory Hypersensitivity/	1474479
14	exp Diabetes Mellitus/ or diabet*.mp.	710026
15	exp Autoimmune Diseases/ or ((autoimmun* or auto immun* or autoaggress* or auto aggress*) adj (disorder* or disease*)).mp.	516672
16	exp Metabolic Syndrome/ or exp Metabolic Diseases/ or ((metabolic or insulin resistance) adj (disorder* or disease* or syndrome*)).mp.	1067250
17	exp Obesity/ or obes*.mp.	365459
18	exp Osteoporosis/ or osteoporo*.mp. or bone loss.mp. or exp osteolysis/ or osteolysis.mp. or bone resorption.mp.	153480

19	exp Parkinson disease/ or parkinson*.mp. or paralysis agitans.mp.	131499
20	exp Arthritis/ or arthriti*.mp. or polyarthriti*.mp. or rheumarthriti*.mp.	321229
21	exp Kidney Diseases/ or (kidney adj (disease* or disorder*)).mp.	544657
22	exp Liver Diseases/ or (liver adj (disease* or disorder* or dysfunction*)).mp.	591670
23	exp Hypertension/ or high blood pressure*.mp. or hypertens*.mp.	522886
24	exp Hyperlipidemias/ or hyperlipem*.mp. or hyperlipidem*.mp. or lipem*.mp. or lipidem*.mp.	84750
25	exp Hypercholesterolemia/ or ((high* or elevat*) adj cholesterol*).mp. or hypercholesterem*.mp. or hypercholesterolem*.mp.	50873
26	exp Hypertriglyceridemia/ or hypertriglyceridem*.mp.	15254
27	exp Thyroid Diseases/ or (thyroid adj (disease* or disorder*)).mp. or exp Hyperthyroidism/ or hyperthyroid*.mp. or exp Hypothyroidism/ or hypothyroid*.mp. or ((thyroid-stimulating hormone* or tsh) adj deficien*).mp.	166497
28	exp Motor Neuron Disease/ or motor neuron* disease*.mp. or lateral scleros*.mp. or motor system disease*.mp.	38409
29	exp Multiple Sclerosis/ or multiple sclerosis.mp. or disseminated sclerosis.mp.	84720
30	exp Emphysema/ or emphysema*.mp.	38522
31	exp Bronchitis/ or bronchit*.mp.	41201
32	1 or 2 or 3 or 4 or 5 or 6 or 7 or 8 or 9 or 10 or 11 or 12 or 13 or 14 or 15 or 16 or 17 or 18 or 19 or 20 or 21 or 22 or 23 or 24 or 25 or 26 or 27 or 28 or 29 or 30 or 31	10292435
33	exp Mental Disorders/ or exp Psychotic Disorders/ or ((mental* or psychiatr* or psycho*) adj (disorder* or disease* or illness*)).mp.	1313460
34	exp Depressive Disorder, Major/ or exp Depression/ or Depress*.mp. or MDD.mp.	552268
35	exp Anxiety Disorders/ or exp Anxiety/ or anxi*.mp.	289411
36	exp Phobic Disorders/ or phobi*.mp.	17668
37	exp Schizophrenia/ or schizophreni*.mp. or hebephreni*.mp.	150158
38	exp Somatoform Disorders/ or ((somatoform* or somati* or medically unexplained or briquet or pain) adj (disorder* or syndrome* or symptom*)).mp. or exp Medically Unexplained Symptoms/	49713
39	exp Dissociative Disorders/ or (dissociative adj (disorder* or hysteri* or reaction*)).mp. or dissociation*.mp.	115459
40	exp Hysteria/ or hysteri*.mp.	6074
41	exp Mood Disorders/ or ((affective* or mood*) adj (disorder* or disease* or illness* or symptom*)).mp.	158894

42	exp Stress Disorders, Post-Traumatic/ or PTSD.mp. or ((post trauma* or posttrauma*) adj (stress* or neurose*)).mp. or combat disorder*.mp. or war disorder*.mp.	48359
43	exp Cognition Disorders/ or ((cognitive or cognition or mental or neurocognitive) adj (dysfunction* or decline* or impairment* or deterioration* or disorder* or illness* or disease*)).mp.	377493
44	exp Personality Disorders/ or personality disorder*.mp.	48830
45	exp "Disruptive, Impulse Control, and Conduct Disorders"/ or impulse control disorder*.mp. or intermittent explosive disorder*.mp.	9781
46	exp "Feeding and Eating Disorders"/ or ((eating or appetite or feeding) adj disorder*).mp.	38361
47	exp Bipolar Disorder/ or ((bipolar or mani*) adj (disorder* or illness* or disease*)).mp.	51927
48	exp Obsessive-Compulsive Disorder/ or OCD*.mp. or ((obsess*-compulsi* or obsess* or compulsi*) adj (disorder* or illness* or disease* or neuros*)).mp.	22060
49	exp Panic Disorder/ or (panic adj (attack* or disorder*)).mp.	12638
50	exp Agoraphobia/ or agoraphobi*.mp.	4210
51	exp Neurotic Disorders/ or neuros*.mp. or neurotic disorder*.mp. or psychoneuros*.mp.	190989
52	33 or 34 or 35 or 36 or 37 or 38 or 39 or 40 or 41 or 42 or 43 or 44 or 45 or 46 or 47 or 48 or 49 or 50 or 51	2107749
53	exp Communicable Diseases/ or ((communic* or contag* or transmi* or infect*) adj (disease* or infection* or illness*)).mp.	212955
54	exp Bacterial Infections/ or bacteri* infection*.mp.	916393
55	exp Conjunctivitis/ or conjunctivitis.mp.	24690
56	exp HIV/ or hiv.mp. or Human immuno deficiency virus.mp.	362332
57	exp Acquired Immunodeficiency Syndrome/ or AIDS.mp. or immunodeficiency associated virus.mp. or immun* deficiency associated virus.mp. or acquired immunodeficiency syndrome*.mp. or acquired immun* deficiency syndrome*.mp.	226679
58	exp Buruli Ulcer/ or Bairnsdale.mp. or Buruli.mp.	1104
59	exp Onchocerciasis/ or onchocer*.mp.	5978
60	hepatitis.mp. or exp Hepatitis B/ or exp Hepatitis C/	256450
61	exp Leishmaniasis/ or leishmania*.mp.	37940
62	exp Leprosy/ or lepros*.mp. or hansen*.mp.	31193
63	exp Elephantiasis, Filarial/ or elephantias*.mp. or filaria*.mp.	14354
64	exp Trachoma/ or egyptian ophthalmia*.mp. or trachoma*.mp.	20432
65	exp Chikungunya Fever/ or chickungunya.mp. or chikungunya.mp.	5766

66	exp Taeniasis/ or taenia*.mp.	12366
67	exp Cysticercosis/ or cysticercos*.mp.	7146
68	exp Echinococcosis/ or hydatid*.mp. or echinococc*.mp.	29913
69	exp Chagas Disease/ or trypanosom*.mp. or chagas.mp.	44250
70	exp Trypanosomiasis/ or sleeping sickness.mp.	23416
71	exp Encephalitis, Japanese/ or (japanese adj3 encephalitis).mp.	5847
72	exp Syphilis/ or syphilis.mp.	37477
73	53 or 54 or 55 or 56 or 57 or 58 or 59 or 60 or 61 or 62 or 63 or 64 or 65 or 66 or 67 or 68 or 69 or 70 or 71 or 72	1872806
74	exp Tuberculosis/	192915
75	Tuberculos*.mp.	256334
76	TB.mp.	56951
77	koch*.mp.	9469
78	exp Tuberculosis/ or Tuberculos*.mp. or TB.mp. or koch*.mp.	283447
79	(multiple adj (ill* or disease* or condition* or syndrom* or disorder*)).mp.	5522
80	((Cooccur* or co-occur* or coexist* or co-exist* or multipl* or concord* or discord* or long-term or physical*) adj3 (disease* or ill* or care or condition* or disorder* or health* or medication* or symptom* or syndrom*)).mp.	269814
81	(comorbid* or multimorbid* or co-occurren* or co-morbid* or Multidisease* or multi-disease*).mp.	268450
82	(comorbid* or multimorbid* or co-occurren* or co-morbid* or multi-morbid* or Multidisease* or multi-disease*).mp.	268954
83	exp Comorbidity/ or exp Multimorbidity/ or exp Multiple Chronic Conditions/	111238
84	79 or 80 or 81 or 82 or 83	519584
85	exp "Systematic Review"/	137409
86	"systematic review*".m_titl.	134940
87	exp Meta-Analysis/	121268
88	"meta-analys*".m_titl.	115466
89	exp "Systematic Review"/ or "systematic review*".m_titl. or exp Meta-Analysis/ or "meta-analys*".m_titl.	260449
90	32 or 52 or 73	13060984
91	(32 or 52 or 73) and 78	228989
92	(32 or 52 or 73) and 78 and 84	4072

93	(32 or 52 or 73) and 78 and 84 and 89	89
94	exp Animals/ not exp Humans/	4747614
95	((32 or 52 or 73) and 78 and 84) not 94	4004
96	((32 or 52 or 73) and 78 and 84 and 89) not 94	89
97	((32 or 52 or 73) and 78 and 89) not 94	1701

For peer review only

Embase (October 23rd 2020)

# ▲	Searches	Results
1	exp non communicable disease/ or ((Non-communicable or Noncommunicable or Non-infectious) adj (disease* or condition* or illness*)).mp.	16547
2	exp chronic disease/ or ((chronic or long-term) adj (disease* or condition* or illness*)).mp.	268915
3	exp heart disease/ or (heart adj (disease* or disorder* or failure)).mp. or (cardiac adj (disease* or disorder* or failure)).mp.	1935434
4	exp cardiovascular disease/ or (cardiovascular adj (disease* or disorder* or failure)).mp.	4171750
5	exp coronary artery disease/ or (coronary adj (disease* or disorder* or failure)).mp.	338836
6	exp cerebrovascular disease/ or (cerebrovascular adj (disease* or disorder* or insufficienc* or occlusion*)).mp. or (vascular adj (disease* or disorder*)).mp. or (carotid* adj (disease* or disorder*)).mp.	760319
7	exp peripheral occlusive artery disease/ or (arter* adj (disease* or disorder*)).mp.	427607
8	exp rheumatic heart disease/ or exp congenital heart malformation/ or (heart adj3 (malform* or defect* or congeni*)).mp.	187115
9	exp vein thrombosis/ or ((deep vein or deep venous) adj thrombos*).mp. or phlebothrombos*.mp.	137063
10	exp lung embolism/ or (pulmonar* adj (thromboembolism* or embolism* or disease* or disorder*)).mp.	205531
11	exp cerebrovascular accident/ or stroke.mp.	513128
12	exp neoplasm/ or Cancer*.mp. or neoplas*.mp. or tumor*.mp.	5810559
13	exp lung disease/ or exp respiratory tract disease/ or exp chronic obstructive lung disease/ or ((lung* or respiratory or pulmonar* or airflow or airway) adj2 (disease* or obstruct* or hypersensitiv*)).mp. or exp asthma/ or asthma*.mp. or exp chronic obstructive lung disease/ or exp respiratory tract allergy/	2529309
14	exp diabetes mellitus/ or diabet*.mp.	1165861
15	exp autoimmune disease/ or ((autoimmun* or auto immun* or autoaggress* or auto aggress*) adj (disorder* or disease*)).mp.	628015
16	exp metabolic syndrome X/ or exp metabolic disorder/ or ((metabolic or insulin resistance) adj (disorder* or disease* or syndrome*)).mp.	2728282
17	exp obesity/ or obes*.mp.	632387
18	exp osteoporosis/ or osteoporo*.mp. or bone loss.mp. or exp osteolysis/ or osteolysis.mp. or bone resorption.mp.	237604
19	exp Parkinson disease/ or parkinson*.mp. or paralysis agitans.mp.	212471

20	exp arthritis/ or arthriti*.mp. or polyarthriti*.mp. or rheumarthriti*.mp.	510423
21	exp kidney disease/ or (kidney adj (disease* or disorder*)).mp.	973568
22	exp liver disease/ or (liver adj (disease* or disorder* or dysfunction*)).mp.	1021173
23	exp hypertension/ or high blood pressure*.mp. or hypertens*.mp.	1010094
24	exp hyperlipidemia/ or hyperlipem*.mp. or hyperlipidem*.mp. or lipem*.mp. or lipidem*.mp.	170635
25	exp hypercholesterolemia/ or ((high* or elevat*) adj cholesterol*).mp. or hypercholesterem*.mp. or hypercholesterolem*.mp.	90843
26	exp hypertriglyceridemia/ or hypertriglyceridem*.mp.	31673
27	exp thyroid disease/ or (thyroid adj (disease* or disorder*)).mp. or exp hyperthyroidism/ or hyperthyroid*.mp. or exp hypothyroidism/ or hypothyroid*.mp. or ((thyroid-stimulating hormone* or tsh) adj deficien*).mp.	238358
28	exp motor neuron disease/ or motor neuron* disease*.mp. or lateral scleros*.mp. or motor system disease*.mp.	52581
29	exp multiple sclerosis/ or multiple sclerosis.mp. or disseminated sclerosis.mp.	143653
30	exp emphysema/ or emphysema*.mp.	52663
31	exp bronchitis/ or bronchit*.mp.	70185
32	1 or 2 or 3 or 4 or 5 or 6 or 7 or 8 or 9 or 10 or 11 or 12 or 13 or 14 or 15 or 16 or 17 or 18 or 19 or 20 or 21 or 22 or 23 or 24 or 25 or 26 or 27 or 28 or 29 or 30 or 31	13930977
33	exp mental disease/ or exp psychosis/ or ((mental* or psychiatr* or psycho*) adj (disorder* or disease* or illness*)).mp.	2252075
34	exp major depression/ or exp depression/ or Depress*.mp. or MDD.mp.	837980
35	exp anxiety disorder/ or exp anxiety/ or anxi*.mp.	518902
36	exp phobia/ or phobi*.mp.	35665
37	exp schizophrenia/ or schizophreni*.mp. or hebephreni*.mp.	209868
38	exp somatoform disorder/ or ((somatoform* or somati* or medically unexplained or briquet or pain) adj (disorder* or syndrome* or symptom*)).mp. or exp medically unexplained symptom/	82640
39	exp dissociative disorder/ or (dissociative adj (disorder* or hysteri* or reaction*)).mp. or dissociation*.mp.	142193
40	exp hysteria/ or hysteri*.mp.	7294
41	exp mood disorder/ or ((affective* or mood*) adj (disorder* or disease* or illness* or symptom*)).mp.	539796
42	exp posttraumatic stress disorder/ or PTSD.mp. or ((post trauma* or posttrauma*) adj (stress* or neurose*)).mp. or combat disorder*.mp. or war disorder*.mp.	68828

43	exp cognitive defect/ or ((cognitive or cognition or mental or neurocognitive) adj (dysfunction* or decline* or impairment* or deterioration* or disorder* or illness* or disease*)).mp.	802224
44	exp personality disorder/ or personality disorder*.mp.	64830
45	exp impulse control disorder/ or impulse control disorder*.mp. or intermittent explosive disorder*.mp.	12514
46	exp eating disorder/ or ((eating or appetite or feeding) adj disorder*).mp.	64625
47	exp bipolar disorder/ or ((bipolar or mani*) adj (disorder* or illness* or disease*)).mp.	73910
48	exp obsessive compulsive disorder/ or OCD*.mp. or ((obsess*-compulsi* or obsess* or compulsi*) adj (disorder* or illness* or disease* or neuros*)).mp.	46156
49	exp panic/ or (panic adj (attack* or disorder*)).mp.	25651
50	exp agoraphobia/ or agoraphobi*.mp.	7128
51	exp neurosis/ or neuros*.mp. or neurotic disorder*.mp. or psychoneuros*.mp.	284790
52	33 or 34 or 35 or 36 or 37 or 38 or 39 or 40 or 41 or 42 or 43 or 44 or 45 or 46 or 47 or 48 or 49 or 50 or 51	3032495
53	exp communicable disease/ or ((communic* or contag* or transmi* or infect*) adj (disease* or infection* or illness*)).mp.	227165
54	exp bacterial infection/ or bacteri* infection*.mp.	875706
55	exp conjunctivitis/ or conjunctivitis.mp.	42120
56	exp Human immunodeficiency virus/ or hiv.mp. or Human immuno deficiency virus.mp.	440016
57	exp acquired immune deficiency syndrome/ or AIDS.mp. or immunodeficiency associated virus.mp. or immun* deficiency associated virus.mp. or acquired immunodeficiency syndrome*.mp. or acquired immun* deficiency syndrome*.mp.	245085
58	exp Buruli ulcer/ or Bairnsdale.mp. or Buruli.mp.	1384
59	exp onchocerciasis/ or onchocer*.mp.	7089
60	hepatitis.mp. or exp hepatitis B/ or exp hepatitis C/	401827
61	exp leishmaniasis/ or leishmania*.mp.	45114
62	exp leprosy/ or lepros*.mp. or hansen*.mp.	32759
63	exp elephantiasis/ or elephantias*.mp. or filaria*.mp.	17315
64	exp trachoma/ or egyptian ophthalmia*.mp. or trachoma*.mp.	25318
65	exp chikungunya/ or chickungunya.mp. or chikungunya.mp.	8172
66	exp taeniasis/ or taenia*.mp.	13801
67	exp Cysticercosis/ or cysticercos*.mp.	5977

68	exp echinococcosis/ or hydatid*.mp. or echinococc*.mp.	30362
69	exp Chagas disease/ or trypanosom*.mp. or chagas.mp.	47764
70	exp trypanosomiasis/ or sleeping sickness.mp.	25238
71	exp Japanese encephalitis/ or (japanese adj3 encephalitis).mp.	7060
72	exp syphilis/ or syphilis.mp.	32633
73	53 or 54 or 55 or 56 or 57 or 58 or 59 or 60 or 61 or 62 or 63 or 64 or 65 or 66 or 67 or 68 or 69 or 70 or 71 or 72	2039904
74	exp tuberculosis/	193084
75	Tuberculos*.mp.	245295
76	TB.mp.	72736
77	koch*.mp.	12432
78	exp tuberculosis/ or Tuberculos*.mp. or TB.mp. or koch*.mp.	284028
79	(multiple adj (ill* or disease* or condition* or syndrom* or disorder*)).mp.	7515
80	((Cooccur* or co-occur* or coexist* or co-exist* or multipl* or concord* or discord* or long-term or physical*) adj3 (disease* or ill* or care or condition* or disorder* or health* or medication* or symptom* or syndrom*)).mp.	468523
81	(comorbid* or multimorbid* or co-occurren* or co-morbid* or Multidisease* or multi-disease*).mp.	461825
82	(comorbid* or multimorbid* or co-occurren* or co-morbid* or multi-morbid* or Multidisease* or multi-disease*).mp.	462635
83	exp Comorbidity/ or exp Multimorbidity/ or exp Multiple Chronic Conditions/	281021
84	79 or 80 or 81 or 82 or 83	895311
85	exp "systematic review"/	268161
86	"systematic review* ".m_titl.	163162
87	exp meta analysis/	200254
88	"meta-analys* ".m_titl.	144571
89	exp "systematic review"/ or "systematic review* ".m_titl. or exp meta analysis/ or "meta-analys* ".m_titl.	411674
90	32 or 52 or 73	16955591
91	(32 or 52 or 73) and 78	237676
92	(32 or 52 or 73) and 78 and 84	9231
93	(32 or 52 or 73) and 78 and 84 and 89	271
94	exp animal/ not exp human/	4710933

95	((32 or 52 or 73) and 78 and 84) not 94	9130
96	((32 or 52 or 73) and 78 and 84 and 89) not 94	271
97	((32 or 52 or 73) and 78 and 89) not 94	3315

For peer review only

PsycINFO (October 23rd 2020)

# ▲	Searches	Results
1	((Non-communicable or Noncommunicable or Non-infectious) adj (disease* or condition* or illness*)).mp.	1054
2	exp Chronic Illness/ or ((chronic or long-term) adj (disease* or condition* or illness*)).mp.	48942
3	exp Heart Disorders/ or (heart adj (disease* or disorder* or failure)).mp. or (cardiac adj (disease* or disorder* or failure)).mp.	22461
4	exp Cardiovascular Disorders/ or (cardiovascular adj (disease* or disorder* or failure)).mp.	68973
5	(coronary adj (disease* or disorder* or failure)).mp.	450
6	exp Cerebrovascular Disorders/ or (cerebrovascular adj (disease* or disorder* or insufficienc* or occlusion*)).mp. or (vascular adj (disease* or disorder*)).mp. or (carotid* adj (disease* or disorder*)).mp.	31536
7	(arter* adj (disease* or disorder*)).mp.	2605
8	(heart adj3 (malform* or defect* or congeni*)).mp.	1007
9	exp Thromboses/ or ((deep vein or deep venous) adj thrombos*).mp. or phlebothrombos*.mp.	1070
10	exp Embolisms/ or (pulmonar* adj (thromboembolism* or embolism* or disease* or disorder*)).mp.	3511
11	exp Cerebrovascular Accidents/ or stroke.mp.	36761
12	exp Neoplasms/ or Cancer*.mp. or neoplas*.mp. or tumor*.mp.	83903
13	exp Lung Disorders/ or exp Respiratory Tract Disorders/ or ((lung* or respiratory or pulmonar* or airflow or airway) adj2 (disease* or obstruct* or hypersensitiv*)).mp. or exp Asthma/ or asthma*.mp. or exp Chronic Obstructive Pulmonary Disease/	20498
14	exp Diabetes Mellitus/ or diabet*.mp.	33566
15	((autoimmun* or auto immun* or autoaggress* or auto aggress*) adj (disorder* or disease*)).mp.	2453
16	exp Metabolic Syndrome/ or ((metabolic or insulin resistance) adj (disorder* or disease* or syndrome*)).mp.	5856

17	exp Obesity/ or obes*.mp.	45201
18	exp Osteoporosis/ or osteoporo*.mp. or bone loss.mp. or osteolysis.mp. or bone resorption.mp.	2582
19	exp Parkinson's Disease/ or parkinson*.mp. or paralysis agitans.mp.	36257
20	exp Arthritis/ or arthriti*.mp. or polyarthriti*.mp. or rheumarthriti*.mp.	7176
21	exp Kidney Diseases/ or (kidney adj (disease* or disorder*)).mp.	2912
22	exp Liver Disorders/ or (liver adj (disease* or disorder* or dysfunction*)).mp.	5371
23	exp Hypertension/ or high blood pressure*.mp. or hypertens*.mp.	20357
24	(hyperlipem* or hyperlipidem* or lipem* or lipidem*).mp.	1314
25	(((high* or elevat*) adj cholesterol*) or hypercholesterem* or hypercholesterolem*).mp.	1637
26	hypertriglyceridem*.mp.	293
27	exp Thyroid Disorders/ or (thyroid adj (disease* or disorder*)).mp. or exp Hyperthyroidism/ or hyperthyroid*.mp. or exp Hypothyroidism/ or hypothyroid*.mp. or ((thyroid-stimulating hormone* or tsh) adj deficien*).mp.	2924
28	exp Nervous System Disorders/ or motor neuron* disease*.mp. or lateral scleros*.mp. or motor system disease*.mp.	319159
29	exp Multiple Sclerosis/ or multiple sclerosis.mp. or disseminated sclerosis.mp.	16491
30	exp Pulmonary Emphysema/ or emphysema*.mp.	285
31	exp Bronchial Disorders/ or bronchit*.mp.	476
32	1 or 2 or 3 or 4 or 5 or 6 or 7 or 8 or 9 or 10 or 11 or 12 or 13 or 14 or 15 or 16 or 17 or 18 or 19 or 20 or 21 or 22 or 23 or 24 or 25 or 26 or 27 or 28 or 29 or 30 or 31	567022
33	exp Mental Disorders/ or exp Psychosis/ or ((mental* or psychiatr* or psycho*) adj (disorder* or disease* or illness*)).mp.	921435
34	exp Major Depression/ or exp "Depression (Emotion)"/ or Depress*.mp. or MDD.mp.	374034
35	exp Anxiety Disorders/ or exp Anxiety/ or anxi*.mp.	273241

36	exp Phobias/ or phobi*.mp.	24242
37	exp Schizophrenia/ or schizophreni*.mp. or hebephreni*.mp.	140371
38	exp Somatoform Disorders/ or ((somatoform* or somati* or medically unexplained or briquet or pain) adj (disorder* or syndrome* or symptom*)).mp.	27313
39	exp Dissociative Disorders/ or (dissociative adj (disorder* or hysteri* or reaction*)).mp. or dissociation*.mp.	24759
40	exp Hysteria/ or hysteri*.mp.	8253
41	exp Affective Disorders/ or ((affective* or mood*) adj (disorder* or disease* or illness* or symptom*)).mp.	170875
42	exp Posttraumatic Stress Disorder/ or PTSD.mp. or ((post trauma* or posttrauma*) adj (stress* or neurose*)).mp. or combat disorder*.mp. or war disorder*.mp.	51508
43	exp Cognitive Impairment/ or ((cognitive or cognition or mental or neurocognitive) adj (dysfunction* or decline* or impairment* or deterioration* or disorder* or illness* or disease*)).mp.	221483
44	exp Personality Disorders/ or personality disorder*.mp.	49598
45	exp Impulse Control Disorders/ or impulse control disorder*.mp. or intermittent explosive disorder*.mp.	2232
46	exp Eating Disorders/ or ((eating or appetite or feeding) adj disorder*).mp.	39055
47	exp Bipolar Disorder/ or ((bipolar or mani*) adj (disorder* or illness* or disease*)).mp.	38332
48	exp Obsessive Compulsive Disorder/ or OCD*.mp. or ((obsess*-compulsi* or obsess* or compulsi*) adj (disorder* or illness* or disease* or neuros*)).mp.	21451
49	exp Panic Disorder/ or (panic adj (attack* or disorder*)).mp.	13643
50	exp Agoraphobia/ or agoraphobi*.mp.	6095
51	exp Neurosis/ or neuros*.mp. or neurotic disorder*.mp. or psychoneuros*.mp.	81072
52	33 or 34 or 35 or 36 or 37 or 38 or 39 or 40 or 41 or 42 or 43 or 44 or 45 or 46 or 47 or 48 or 49 or 50 or 51	1284709

53	exp Infectious Disorders/ or ((communic* or contag* or transmi* or infect*) adj (disease* or infection* or illness*)).mp.	70725
54	exp Bacterial Disorders/ or bacteri* infection*.mp.	2731
55	exp Eye Disorders/ or conjunctivitis.mp.	4950
56	exp HIV/ or hiv.mp. or Human immuno deficiency virus.mp.	57416
57	exp AIDS/ or AIDS.mp. or immunodeficiency associated virus.mp. or immun* deficiency associated virus.mp. or acquired immunodeficiency syndrome*.mp. or acquired immun* deficiency syndrome*.mp.	48739
58	(Bairnsdale or Buruli).mp.	7
59	onchocer*.mp.	47
60	hepatitis.mp. or exp Hepatitis/	4736
61	leishmania*.mp.	61
62	(lepros* or hansen*).mp.	1155
63	(elephantias* or filaria*).mp.	75
64	(egyptian ophthalmia* or trachoma*).mp.	309
65	(chickungunya or chikungunya).mp.	39
66	taenia*.mp.	160
67	cysticercos*.mp.	102
68	(hydatid* or echinococc*).mp.	39
69	(trypanosom* or chagas).mp.	217
70	sleeping sickness.mp.	50
71	(japanese adj3 encephalitis).mp.	73
72	exp Syphilis/ or syphilis.mp.	1870

73	53 or 54 or 55 or 56 or 57 or 58 or 59 or 60 or 61 or 62 or 63 or 64 or 65 or 66 or 67 or 68 or 69 or 70 or 71 or 72	108493
74	exp Tuberculosis/	1223
75	Tuberculos*.mp.	2795
76	TB.mp.	1354
77	koch*.mp.	1045
78	exp Tuberculosis/ or Tuberculos*.mp. or TB.mp. or koch*.mp.	4450
79	(multiple adj (ill* or disease* or condition* or syndrom* or disorder*)).mp.	913
80	((Cooccur* or co-occur* or coexist* or co-exist* or multipl* or concord* or discord* or long-term or physical*) adj3 (disease* or ill* or care or condition* or disorder* or health* or medication* or symptom* or syndrom*)).mp.	116177
81	(comorbid* or multimorbid* or co-occurren* or co-morbid* or multi-morbid* or Multidisease* or multi-disease*).mp.	88147
82	exp Comorbidity/	33088
83	79 or 80 or 81 or 82	192590
84	exp "Systematic Review"/	460
85	"systematic review*".m_titl.	21652
86	exp Meta Analysis/	4825
87	"meta-analys*".m_titl.	18645
88	exp "Systematic Review"/ or "systematic review*".m_titl. or exp Meta Analysis/ or "meta-analys*".m_titl.	37279
89	32 or 52 or 73	1665397
90	(32 or 52 or 73) and 78	3046
91	(32 or 52 or 73) and 78 and 83	362

92	(32 or 52 or 73) and 78 and 83 and 88	6
93	(32 or 52 or 73) and 78 and 88	27

For peer review only

Web of Science (October 23rd 2020)

#90	1,039	(#74 OR #52 OR #32) AND #82 AND #85<br>*Indexes=SCI-EXPANDED, SSCI, A&HCI, CPCI-S, CPCI-SSH, ESCI Timespan=All years*
#89	62	(#74 OR #52 OR #32) AND #82 AND #78 AND #85<br>*Indexes=SCI-EXPANDED, SSCI, A&HCI, CPCI-S, CPCI-SSH, ESCI Timespan=All years*
#88	3,336	(#74 OR #52 OR #32) AND #82 AND #78<br>*Indexes=SCI-EXPANDED, SSCI, A&HCI, CPCI-S, CPCI-SSH, ESCI Timespan=All years*
#87	87,846	(#74 OR #52 OR #32) AND #82<br>*Indexes=SCI-EXPANDED, SSCI, A&HCI, CPCI-S, CPCI-SSH, ESCI Timespan=All years*
#86	11,086,931	#74 OR #52 OR #32<br>*Indexes=SCI-EXPANDED, SSCI, A&HCI, CPCI-S, CPCI-SSH, ESCI Timespan=All years*
#85	226,528	#84 OR #83<br>*Indexes=SCI-EXPANDED, SSCI, A&HCI, CPCI-S, CPCI-SSH, ESCI Timespan=All years*
#84	134,872	TI="Meta-analysis"<br>*Indexes=SCI-EXPANDED, SSCI, A&HCI, CPCI-S, CPCI-SSH, ESCI Timespan=All years*
#83	147,319	TI = "Systematic Review"<br>*Indexes=SCI-EXPANDED, SSCI, A&HCI, CPCI-S, CPCI-SSH, ESCI Timespan=All years*
#82	234,479	#81 OR #80 OR #79<br>*Indexes=SCI-EXPANDED, SSCI, A&HCI, CPCI-S, CPCI-SSH, ESCI Timespan=All years*
#81	19,329	TS=koch*<br>*Indexes=SCI-EXPANDED, SSCI, A&HCI, CPCI-S, CPCI-SSH, ESCI Timespan=All years*
#80	80,779	TS=TB<br>*Indexes=SCI-EXPANDED, SSCI, A&HCI, CPCI-S, CPCI-SSH, ESCI Timespan=All years*

# 79	170,591	TS=Tuberculos*<br>*Indexes=SCI-EXPANDED, SSCI, A&HCI, CPCI-S, CPCI-SSH, ESCI Timespan=All years*
# 78	659,174	#77 OR #76 OR #75<br>*Indexes=SCI-EXPANDED, SSCI, A&HCI, CPCI-S, CPCI-SSH, ESCI Timespan=All years*
# 77	238,429	TS=(comorbid* or multimorbid* or co-occurren* or co-morbid* or multi-morbid* or Multidisease* or multi-disease*)<br>*Indexes=SCI-EXPANDED, SSCI, A&HCI, CPCI-S, CPCI-SSH, ESCI Timespan=All years*
# 76	325,588	TS=((Cooccur* or co-occur* or coexist* or co-exist* or multipl* or concord* or discord* or long-term or physical*) NEAR/3 (disease* or ill* or care or condition* or disorder* or health* or medication* or symptom* or syndrom*))<br>*Indexes=SCI-EXPANDED, SSCI, A&HCI, CPCI-S, CPCI-SSH, ESCI Timespan=All years*
# 75	191,473	TS=(multiple NEAR (ill* or disease* or condition* or syndrom* or disorder*))<br>*Indexes=SCI-EXPANDED, SSCI, A&HCI, CPCI-S, CPCI-SSH, ESCI Timespan=All years*
# 74	2,677,956	#73 OR #72 OR #71 OR #70 OR #69 OR #68 OR #67 OR #66 OR #65 OR #64 OR #63 OR #62 OR #61 OR #60 OR #59 OR #58 OR #57 OR #56 OR #55 OR #54 OR #53<br>*Indexes=SCI-EXPANDED, SSCI, A&HCI, CPCI-S, CPCI-SSH, ESCI Timespan=All years*
# 73	23,389	TS=syphilis<br>*Indexes=SCI-EXPANDED, SSCI, A&HCI, CPCI-S, CPCI-SSH, ESCI Timespan=All years*
# 72	5,981	TS=(japanese NEAR/3 encephalitis)<br>*Indexes=SCI-EXPANDED, SSCI, A&HCI, CPCI-S, CPCI-SSH, ESCI Timespan=All years*
# 71	4,257	TS=sleeping sickness<br>*Indexes=SCI-EXPANDED, SSCI, A&HCI, CPCI-S, CPCI-SSH, ESCI Timespan=All years*
# 70	50,013	TS=trypanosom* or TS=chagas<br>*Indexes=SCI-EXPANDED, SSCI, A&HCI, CPCI-S, CPCI-SSH, ESCI Timespan=All years*
# 69	19,785	TS=hydatid* OR TS=echinococc*<br>*Indexes=SCI-EXPANDED, SSCI, A&HCI, CPCI-S, CPCI-SSH, ESCI Timespan=All years*

# 68	4,657	TS=cysticercos*<br>*Indexes=SCI-EXPANDED, SSCI, A&HCI, CPCI-S, CPCI-SSH, ESCI Timespan=All years*
# 67	8,257	TS=taenia*<br>*Indexes=SCI-EXPANDED, SSCI, A&HCI, CPCI-S, CPCI-SSH, ESCI Timespan=All years*
# 66	6,629	TS=chickungunya Or TS=chikungunya<br>*Indexes=SCI-EXPANDED, SSCI, A&HCI, CPCI-S, CPCI-SSH, ESCI Timespan=All years*
# 65	19,987	TS=trachoma*<br>*Indexes=SCI-EXPANDED, SSCI, A&HCI, CPCI-S, CPCI-SSH, ESCI Timespan=All years*
# 64	5	TS=egyptian ophthalmia*<br>*Indexes=SCI-EXPANDED, SSCI, A&HCI, CPCI-S, CPCI-SSH, ESCI Timespan=All years*
# 63	11,257	TS=elephantias* OR TS=filaria*<br>*Indexes=SCI-EXPANDED, SSCI, A&HCI, CPCI-S, CPCI-SSH, ESCI Timespan=All years*
# 62	30,101	TS=lepros* OR TS=hansen*<br>*Indexes=SCI-EXPANDED, SSCI, A&HCI, CPCI-S, CPCI-SSH, ESCI Timespan=All years*
# 61	41,800	TS=leishmania*<br>*Indexes=SCI-EXPANDED, SSCI, A&HCI, CPCI-S, CPCI-SSH, ESCI Timespan=All years*
# 60	267,930	TS=hepatitis<br>*Indexes=SCI-EXPANDED, SSCI, A&HCI, CPCI-S, CPCI-SSH, ESCI Timespan=All years*
# 59	6,453	TS=onchocer*<br>*Indexes=SCI-EXPANDED, SSCI, A&HCI, CPCI-S, CPCI-SSH, ESCI Timespan=All years*
# 58	1,120	TS=Bairnsdale OR TS=Buruli<br>*Indexes=SCI-EXPANDED, SSCI, A&HCI, CPCI-S, CPCI-SSH, ESCI Timespan=All years*
# 57	640,388	TS=AIDS OR TS=immunodeficiency associated virus OR TS=immun* deficiency associated virus OR TS=acquired immunodeficiency syndrome* OR TS=acquired immun* deficiency syndrome*

		Indexes=SCI-EXPANDED, SSCI, A&HCI, CPCI-S, CPCI-SSH, ESCI Timespan=All years
# 56	374,418	TS=hiv OR TS=Human immuno deficiency virus<br>*Indexes=SCI-EXPANDED, SSCI, A&HCI, CPCI-S, CPCI-SSH, ESCI Timespan=All years*
# 55	10,545	TS=conjunctivitis<br>*Indexes=SCI-EXPANDED, SSCI, A&HCI, CPCI-S, CPCI-SSH, ESCI Timespan=All years*
# 54	240,625	TS=bacteri* infection*<br>*Indexes=SCI-EXPANDED, SSCI, A&HCI, CPCI-S, CPCI-SSH, ESCI Timespan=All years*
# 53	1,710,573	TS=((communic* or contag* or transmi* or infect*) NEAR (disease* or infection* or illness*))<br>*Indexes=SCI-EXPANDED, SSCI, A&HCI, CPCI-S, CPCI-SSH, ESCI Timespan=All years*
# 52	1,891,336	#51 OR #50 OR #49 OR #48 OR #47 OR #46 OR #45 OR #44 OR #43 OR #42 OR #41 OR #40 OR #39 OR #38 OR #37 OR #36 OR #35 OR #34 OR #33<br>*Indexes=SCI-EXPANDED, SSCI, A&HCI, CPCI-S, CPCI-SSH, ESCI Timespan=All years*
# 51	155,801	TS=neuros* OR TS=neurotic disorder* OR TS=psychoneuros*<br>*Indexes=SCI-EXPANDED, SSCI, A&HCI, CPCI-S, CPCI-SSH, ESCI Timespan=All years*
# 50	5,165	TS=agoraphobi*<br>*Indexes=SCI-EXPANDED, SSCI, A&HCI, CPCI-S, CPCI-SSH, ESCI Timespan=All years*
# 49	19,459	TS=(panic NEAR (attack* or disorder*))<br>*Indexes=SCI-EXPANDED, SSCI, A&HCI, CPCI-S, CPCI-SSH, ESCI Timespan=All years*
# 48	32,551	TS=OCD* OR TS=((obsess*-compulsi* or obsess* or compulsi*) NEAR (disorder* or illness* or disease* or neuros*))<br>*Indexes=SCI-EXPANDED, SSCI, A&HCI, CPCI-S, CPCI-SSH, ESCI Timespan=All years*
# 47	137,039	TS=((bipolar or mani*) NEAR (disorder* or illness* or disease*))<br>*Indexes=SCI-EXPANDED, SSCI, A&HCI, CPCI-S, CPCI-SSH, ESCI Timespan=All years*
# 46	35,573	TS=((eating or appetite or feeding) NEAR disorder*)<br>*Indexes=SCI-EXPANDED, SSCI, A&HCI, CPCI-S, CPCI-SSH, ESCI Timespan=All years*

#45	4,535	TS=impulse control disorder* OR TS=intermittent explosive disorder*<br>*Indexes=SCI-EXPANDED, SSCI, A&HCI, CPCI-S, CPCI-SSH, ESCI Timespan=All years*
#44	52,384	TS=personality disorder*<br>*Indexes=SCI-EXPANDED, SSCI, A&HCI, CPCI-S, CPCI-SSH, ESCI Timespan=All years*
#43	325,105	TS=((cognitive or cognition or mental or neurocognitive) NEAR (dysfunction* or decline* or impairment* or deterioration* or disorder* or illness* or disease*))<br>*Indexes=SCI-EXPANDED, SSCI, A&HCI, CPCI-S, CPCI-SSH, ESCI Timespan=All years*
#42	69,002	TS=PTSD OR TS=(((post NEAR trauma*) or posttrauma*) NEAR (stress* or neurose*)) OR TS=combat disorder* OR TS=war disorder*<br>*Indexes=SCI-EXPANDED, SSCI, A&HCI, CPCI-S, CPCI-SSH, ESCI Timespan=All years*
#41	73,280	TS=((affective* or mood*) NEAR (disorder* or disease* or illness* or symptom*))<br>*Indexes=SCI-EXPANDED, SSCI, A&HCI, CPCI-S, CPCI-SSH, ESCI Timespan=All years*
#40	6,468	TS=hysteri*<br>*Indexes=SCI-EXPANDED, SSCI, A&HCI, CPCI-S, CPCI-SSH, ESCI Timespan=All years*
#39	215,870	TS=(dissociative NEAR (disorder* or hysteri* or reaction*)) OR TS=dissociation*<br>*Indexes=SCI-EXPANDED, SSCI, A&HCI, CPCI-S, CPCI-SSH, ESCI Timespan=All years*
#38	122,031	TS=((somatoform* or somati* or (medically NEAR unexplained) or briquet or pain) NEAR (disorder* or syndrome* or symptom*))<br>*Indexes=SCI-EXPANDED, SSCI, A&HCI, CPCI-S, CPCI-SSH, ESCI Timespan=All years*
#37	200,099	TS=schizophreni* OR TS=hebephreni*<br>*Indexes=SCI-EXPANDED, SSCI, A&HCI, CPCI-S, CPCI-SSH, ESCI Timespan=All years*
#36	19,359	TS=phobi*<br>*Indexes=SCI-EXPANDED, SSCI, A&HCI, CPCI-S, CPCI-SSH, ESCI Timespan=All years*
#35	300,816	TS=anxi*<br>*Indexes=SCI-EXPANDED, SSCI, A&HCI, CPCI-S, CPCI-SSH, ESCI Timespan=All years*
#34	645,434	TS=Depress* OR TS=MDD<br>*Indexes=SCI-EXPANDED, SSCI, A&HCI, CPCI-S, CPCI-SSH, ESCI Timespan=All years*

# 33	283,212	TS=((mental* or psychiatr* or psycho*) NEAR (disorder* or disease* or illness*))<br>*Indexes=SCI-EXPANDED, SSCI, A&HCI, CPCI-S, CPCI-SSH, ESCI Timespan=All years*
# 32	7,479,780	#31 OR #30 OR #29 OR #28 OR #27 OR #26 OR #25 OR #24 OR #23 OR #22 OR #21 OR #20 OR #19 OR #18 OR #17 OR #16 OR #15 OR #14 OR #13 OR #12 OR #11 OR #10 OR #9 OR #8 OR #7 OR #6 OR #5 OR #4 OR #3 OR #2 OR #1<br>*Indexes=SCI-EXPANDED, SSCI, A&HCI, CPCI-S, CPCI-SSH, ESCI Timespan=All years*
# 31	20,591	TS=bronchit*<br>*Indexes=SCI-EXPANDED, SSCI, A&HCI, CPCI-S, CPCI-SSH, ESCI Timespan=All years*
# 30	26,727	TS=emphysema*<br>*Indexes=SCI-EXPANDED, SSCI, A&HCI, CPCI-S, CPCI-SSH, ESCI Timespan=All years*
# 29	129,081	TS=multiple sclerosis OR TS=disseminated sclerosis<br>*Indexes=SCI-EXPANDED, SSCI, A&HCI, CPCI-S, CPCI-SSH, ESCI Timespan=All years*
# 28	67,922	TS=motor neuron* disease* OR TS=lateral scleros* OR TS=motor system disease*<br>*Indexes=SCI-EXPANDED, SSCI, A&HCI, CPCI-S, CPCI-SSH, ESCI Timespan=All years*
# 27	71,105	TS=(thyroid NEAR (disease* or disorder*)) OR TS=hyperthyroid* OR TS=hypothyroid* OR TS=(((thyroid-stimulating NEAR hormone*) or tsh) NEAR deficien*)<br>*Indexes=SCI-EXPANDED, SSCI, A&HCI, CPCI-S, CPCI-SSH, ESCI Timespan=All years*
# 26	14,111	TS=hypertriglyceridem*<br>*Indexes=SCI-EXPANDED, SSCI, A&HCI, CPCI-S, CPCI-SSH, ESCI Timespan=All years*
# 25	113,697	TS=((high* or elevat*) NEAR cholesterol*) Or TS=hypercholesterem* OR TS=hypercholesterolem*<br>*Indexes=SCI-EXPANDED, SSCI, A&HCI, CPCI-S, CPCI-SSH, ESCI Timespan=All years*
# 24	36,981	TS=hyperlipem* OR TS=hyperlipidem* or TS=lipem* OR TS=lipidem*<br>*Indexes=SCI-EXPANDED, SSCI, A&HCI, CPCI-S, CPCI-SSH, ESCI Timespan=All years*
# 23	613,541	TS=high blood pressure* OR TS=hypertens*<br>*Indexes=SCI-EXPANDED, SSCI, A&HCI, CPCI-S, CPCI-SSH, ESCI Timespan=All years*

# 22	161,103	TS=(liver NEAR (disease* or disorder* or dysfunction*))<br>*Indexes=SCI-EXPANDED, SSCI, A&HCI, CPCI-S, CPCI-SSH, ESCI Timespan=All years*
# 21	125,492	TS=(kidney NEAR (disease* or disorder*))<br>*Indexes=SCI-EXPANDED, SSCI, A&HCI, CPCI-S, CPCI-SSH, ESCI Timespan=All years*
# 20	278,897	TS=arthriti* OR TS=polyarthriti* OR TS=rheumarthriti*<br>*Indexes=SCI-EXPANDED, SSCI, A&HCI, CPCI-S, CPCI-SSH, ESCI Timespan=All years*
# 19	181,735	TS=parkinson* OR TS=paralysis agitans<br>*Indexes=SCI-EXPANDED, SSCI, A&HCI, CPCI-S, CPCI-SSH, ESCI Timespan=All years*
# 18	195,021	TS=osteoporo* OR TS=bone loss OR TS=osteolysis or TS=bone resorption<br>*Indexes=SCI-EXPANDED, SSCI, A&HCI, CPCI-S, CPCI-SSH, ESCI Timespan=All years*
# 17	426,882	TS=obes*<br>*Indexes=SCI-EXPANDED, SSCI, A&HCI, CPCI-S, CPCI-SSH, ESCI Timespan=All years*
# 16	174,058	TS=((metabolic or (insulin near resistance)) NEAR (disorder* or disease* or syndrome*))<br>*Indexes=SCI-EXPANDED, SSCI, A&HCI, CPCI-S, CPCI-SSH, ESCI Timespan=All years*
# 15	109,133	TS=((autoimmun* or (auto NEAR immun*) or autoaggress* or (auto NEAR aggress*)) NEAR (disorder* or disease*))<br>*Indexes=SCI-EXPANDED, SSCI, A&HCI, CPCI-S, CPCI-SSH, ESCI Timespan=All years*
# 14	771,913	TS=diabet*<br>*Indexes=SCI-EXPANDED, SSCI, A&HCI, CPCI-S, CPCI-SSH, ESCI Timespan=All years*
# 13	385,258	TS=((lung* or respiratory or pulmonar* or airflow or airway) NEAR/2 (disease* or obstruct* or hypersensitiv*)) OR TS=asthma*<br>*Indexes=SCI-EXPANDED, SSCI, A&HCI, CPCI-S, CPCI-SSH, ESCI Timespan=All years*
# 12	3,520,631	TS=Cancer* or TS=neoplas* OR TS=tumor*<br>*Indexes=SCI-EXPANDED, SSCI, A&HCI, CPCI-S, CPCI-SSH, ESCI Timespan=All years*
# 11	356,775	TS=stroke<br>*Indexes=SCI-EXPANDED, SSCI, A&HCI, CPCI-S, CPCI-SSH, ESCI Timespan=All years*

# 10	164,438	TS=(pulmonar* NEAR (thromboembolism* or embolism* or disease* or disorder*))<br>*Indexes=SCI-EXPANDED, SSCI, A&HCI, CPCI-S, CPCI-SSH, ESCI Timespan=All years*
# 9	34,725	TS=(("deep vein" or "deep venous") NEAR thrombos*) OR TS=phlebothrombos*<br>*Indexes=SCI-EXPANDED, SSCI, A&HCI, CPCI-S, CPCI-SSH, ESCI Timespan=All years*
# 8	52,910	TS=(heart NEAR/3 (malform* or defect* or congeni*))<br>*Indexes=SCI-EXPANDED, SSCI, A&HCI, CPCI-S, CPCI-SSH, ESCI Timespan=All years*
# 7	223,717	TS=(arter* NEAR (disease* or disorder*))<br>*Indexes=SCI-EXPANDED, SSCI, A&HCI, CPCI-S, CPCI-SSH, ESCI Timespan=All years*
# 6	131,935	TS=(cerebrovascular NEAR (disease* or disorder* or insufficienc* or occlusion*)) OR TS=(vascular NEAR (disease* or disorder*)) OR TS=(carotid* NEAR (disease* or disorder*))<br>*Indexes=SCI-EXPANDED, SSCI, A&HCI, CPCI-S, CPCI-SSH, ESCI Timespan=All years*
# 5	260,403	TS=(coronary NEAR (disease* or disorder* or failure))<br>*Indexes=SCI-EXPANDED, SSCI, A&HCI, CPCI-S, CPCI-SSH, ESCI Timespan=All years*
# 4	286,220	TS=(cardiovascular NEAR (disease* or disorder* or failure))<br>*Indexes=SCI-EXPANDED, SSCI, A&HCI, CPCI-S, CPCI-SSH, ESCI Timespan=All years*
# 3	561,756	TS=(heart NEAR (disease* or disorder* or failure)) OR TS=(cardiac NEAR (disease* or disorder* or failure))<br>*Indexes=SCI-EXPANDED, SSCI, A&HCI, CPCI-S, CPCI-SSH, ESCI Timespan=All years*
# 2	500,606	TS=((chronic or long-term) NEAR (disease* or condition* or illness*))<br>*Indexes=SCI-EXPANDED, SSCI, A&HCI, CPCI-S, CPCI-SSH, ESCI Timespan=All years*
# 1	12,403	TS=((Non-communicable or Noncommunicable or Non-infectious) NEAR/1 (disease* or condition* or illness*))<br>*Indexes=SCI-EXPANDED, SSCI, A&HCI, CPCI-S, CPCI-SSH, ESCI Timespan=All years*

Global Index Medicus (October 23rd 2020)

(((("Noncommunicable Diseases" OR ((("Non-communicable" or Noncommunicable or "Non-infectious") AND (disease* or condition* or illness*)))) OR ("Chronic Disease" OR (((chronic or "long-term") AND (disease* or condition* or illness*)))) OR ("Heart Diseases" OR ((heart AND (disease* or disorder* or failure)) or (cardiac AND (disease* or disorder* or failure)))) OR ("Cardiovascular Diseases" OR ((cardiovascular AND (disease* or disorder* or failure)))) OR ("Coronary Disease" OR (coronary AND (disease* or disorder* or failure))) OR ("Cerebrovascular Disorders" OR ((cerebrovascular AND (disease* or disorder* or insufficienc* or occlusion*)) or (vascular AND (disease* or disorder*)) or (carotid* AND (disease* or disorder*)))) OR ("Peripheral Arterial Disease" OR ((arter* AND (disease* or disorder*)))) OR ("Rheumatic Heart Disease" OR "Heart Defects, Congenital" OR ((heart AND (malform* or defect* or congeni*)))) OR ("Venous Thrombosis" OR ((("deep vein" or "deep venous") AND thrombos*) or phlebothrombos*)) OR ("Pulmonary Embolism" OR ((pulmonar* AND (thromboembolism* or embolism* or disease* or disorder*)))) OR ("Stroke" OR (stroke)) OR ("Neoplasms" OR (Cancer* or neoplas* or tumor*)) OR ("Lung Diseases" OR "Respiratory Tract Diseases" OR "Lung Diseases, Obstructive" OR (((lung* or respiratory or pulmonar* or airflow or airway) AND (disease* or obstruct* or hypersensitiv*))) OR "Asthma" OR asthma* OR "Pulmonary Disease, Chronic Obstructive" OR "Respiratory Hypersensitivity") OR ("Diabetes Mellitus" or diabet*) OR ("Autoimmune Diseases" OR ((autoimmun* or (auto AND immun*) or autoaggress* or (auto AND aggress*)) AND (disorder* or disease*))) OR ("Metabolic Syndrome" OR "Metabolic Diseases" OR (((metabolic or "insulin resistance") AND (disorder* or disease* or syndrome*)))) OR ("Obesity" OR obes*) OR ("Osteoporosis" OR (osteoporo* or "bone loss" or "Osteolysis" or osteolysis or "bone resorption")) OR ("Parkinson Disease" OR (parkinson* or "paralysis agitans")) OR ("Arthritis" OR (arthriti* or polyarthriti* or rheumarthriti*)) OR ("Kidney Diseases" OR ((kidney AND (disease* or disorder*)))) OR ("Liver Diseases" OR (liver AND (disease* or disorder* or dysfunction*))) OR ("Hypertension" OR ("high blood pressure" or hypertens*)) OR ("Hyperlipidemias" OR (hyperlipem* or hyperlipidem* or lipem* or lipidem*)) OR ("Hypercholesterolemia" OR (((high* or elevat*) AND cholesterol*) or hypercholesterem* or hypercholesterolem*)) OR ("Hypertriglyceridemia " OR (hypertriglyceridem*)) OR ("Thyroid Diseases" OR (thyroid AND (disease* or disorder*)) OR "Hyperthyroidism " OR (hyperthyroid*) OR "Hypothyroidism" OR (hypothyroid*) OR ((("thyroid-stimulating hormone" or tsh) AND deficien*))) OR ("Motor Neuron Disease" OR ("motor neuron disease") OR (lateral AND scleros*) OR ("motor system disease")) OR ("Multiple Sclerosis" OR ("multiple sclerosis" or "disseminated sclerosis")) OR ("Emphysema" OR emphysema*) OR ("Bronchitis" OR bronchit*)) OR (("Mental Disorders" OR "Psychotic Disorders" OR (((mental* or psychiatr* or psycho*) AND (disorder* or disease* or illness*)))) OR ("Depressive Disorder, Major" OR "Depression" OR (Depress* or MDD)) OR ("Anxiety Disorders" OR "Anxiety" OR anxi*) OR ("Phobic Disorders" OR phobi*) OR ("Schizophrenia" Or (schizophreni* or hebephreni*)) OR ("Somatoform Disorders" OR "Medically Unexplained Symptoms" OR (((somatoform* or somati* or (medically AND unexplained) or briquet or pain) AND (disorder* or syndrome* or symptom*)))) OR ("Dissociative Disorders" OR (dissociative AND (disorder* or hysteri* or reaction*)) or dissociation*) OR ("Hysteria" or hysteri*) OR ("Mood Disorders" OR ((affective* or mood*) AND (disorder* or disease* or illness* or symptom*))) OR ("Stress Disorders, Post-Traumatic" OR (PTSD or ((posttrauma* or trauma*) AND (stress* or neurose*)) or "combat disorder" or "war disorder")) OR ("Cognition Disorders" OR (((cognitive or cognition or mental or neurocognitive) AND (dysfunction* or decline* or impairment* or deterioration* or disorder* or illness* or disease*)))) OR ("Personality Disorders" OR "personality disorder") OR ("Disruptive, Impulse Control, and Conduct Disorders" OR ("impulse control disorder" or "intermittent explosive disorder")) OR ("Feeding and Eating Disorders" OR ((eating or appetite or feeding) AND disorder*)) OR ("Bipolar Disorder" OR ((bipolar or mani*) AND (disorder* or illness* or disease*))) OR ("Obsessive-Compulsive Disorder" OR OCD* or ((obsess* or compulsi*) AND (disorder* or illness* or disease*

or neuros*))) OR ("Panic Disorder" OR ((panic AND (attack* or disorder*)))) OR ("Agoraphobia" OR agoraphobi*) OR ("Neurotic Disorders" OR (neuros* or "neurotic disorder" or psychoneuros*))) OR (("Communicable Diseases" OR (((communic* or contag* or transmi* or infect*) AND (disease* or infection* or illness*)))) OR ("Bacterial Infections" OR (bacteri* infection*)) OR ("Conjunctivitis") OR ("HIV" OR "Human immuno deficiency virus") OR ("Acquired Immunodeficiency Syndrome" OR (AIDS or "immunodeficiency associated virus" or (immun* deficiency associated virus) or (acquired immunodeficiency syndrome*) or (acquired immun* deficiency syndrome*))) OR ("Buruli Ulcer" OR (Bairnsdale or Buruli)) OR ("Onchocerciasis" OR (onchocer*)) OR ("Hepatitis B" OR "Hepatitis C" OR (hepatitis)) OR ("Leishmaniasis" OR (leishmania*)) OR ("Leprosy" OR (lepros* or hansen*)) OR ("Elephantiasis, Filarial" OR (elephantias* or filaria*)) OR ("Trachoma" OR ((egyptian ophthalmia*) or trachoma*)) OR ("Chikungunya Fever" OR (chickungunya or chikungunya)) OR ("Taeniasis" OR taenia*) OR ("Cysticercosis" OR cysticercos*) OR ("Echinococcosis" OR (hydatid or echinococc*)) OR ("Chagas Disease" OR (trypanosom* or chagas)) OR ("Trypanosomiasis" OR ("sleeping sickness")) OR ("Encephalitis, Japanese" OR (japanese encephalitis)) OR ("Syphilis"))) AND ("Tuberculosis" OR Tuberculos* OR TB OR koch*)) AND ("Systematic Review" OR (systematic AND review*) OR "Meta-Analysis" OR meta-analys*)
131

OpenGrey (23/10/2020)

(((("Noncommunicable Diseases" OR ((("Non-communicable" or Noncommunicable or "Non-infectious") AND (disease* or condition* or illness*)))) OR ("Chronic Disease" OR (((chronic or "long-term") AND (disease* or condition* or illness*)))) OR ("Heart Diseases" OR ((heart AND (disease* or disorder* or failure)) or (cardiac AND (disease* or disorder* or failure)))) OR ("Cardiovascular Diseases" OR ((cardiovascular AND (disease* or disorder* or failure)))) OR ("Coronary Disease" OR (coronary AND (disease* or disorder* or failure))) OR ("Cerebrovascular Disorders" OR ((cerebrovascular AND (disease* or disorder* or insufficienc* or occlusion*)) or (vascular AND (disease* or disorder*)) or (carotid* AND (disease* or disorder*)))) OR ("Peripheral Arterial Disease" OR ((arter* AND (disease* or disorder*)))) OR ("Rheumatic Heart Disease" OR "Heart Defects, Congenital" OR ((heart AND (malform* or defect* or congeni*)))) OR ("Venous Thrombosis" OR ((("deep vein" or "deep venous") AND thrombos*) or phlebothrombos*)) OR ("Pulmonary Embolism" OR ((pulmonar* AND (thromboembolism* or embolism* or disease* or disorder*)))) OR ("Stroke" OR (stroke)) OR ("Neoplasms" OR (Cancer* or neoplas* or tumor*)) OR ("Lung Diseases" OR "Respiratory Tract Diseases" OR "Lung Diseases, Obstructive" OR (((lung* or respiratory or pulmonar* or airflow or airway) AND (disease* or obstruct* or hypersensitiv*))) OR "Asthma" OR asthma* OR "Pulmonary Disease, Chronic Obstructive" OR "Respiratory Hypersensitivity") OR ("Diabetes Mellitus" or diabet*) OR ("Autoimmune Diseases" OR ((autoimmun* or (auto AND immun*) or autoaggress* or (auto AND aggress*)) AND (disorder* or disease*))) OR ("Metabolic Syndrome" OR "Metabolic Diseases" OR (((metabolic or "insulin resistance") AND (disorder* or disease* or syndrome*)))) OR ("Obesity" OR obes*) OR ("Osteoporosis" OR (osteoporo* or "bone loss" or "Osteolysis" or osteolysis or "bone resorption")) OR ("Parkinson Disease" OR (parkinson* or "paralysis agitans")) OR ("Arthritis" OR (arthriti* or polyarthriti* or rheumarthriti*)) OR ("Kidney Diseases" OR ((kidney AND (disease* or disorder*)))) OR ("Liver Diseases" OR (liver AND (disease* or disorder* or dysfunction*))) OR ("Hypertension" OR ("high blood pressure" or hypertens*)) OR ("Hyperlipidemias" OR (hyperlipem* or hyperlipidem* or lipem* or lipidem*)) OR ("Hypercholesterolemia" OR (((high* or elevat*) AND cholesterol*) or hypercholesterem* or hypercholesterolem*)) OR ("Hypertriglyceridemia " OR (hypertriglyceridem*)) OR ("Thyroid Diseases" OR (thyroid AND (disease* or disorder*)) OR

For peer review only

"Hyperthyroidism " OR (hyperthyroid*) OR "Hypothyroidism" OR (hypothyroid*) OR ((("thyroid-stimulating hormone" or tsh) AND deficien*))) OR ("Motor Neuron Disease" OR ("motor neuron disease") OR (lateral AND scleros*) OR ("motor system disease")) OR ("Multiple Sclerosis" OR ("multiple sclerosis" or "disseminated sclerosis")) OR ("Emphysema" OR emphysema*) OR ("Bronchitis" OR bronchit*)) OR (("Mental Disorders" OR "Psychotic Disorders" OR (((mental* or psychiatr* or psycho*) AND (disorder* or disease* or illness*)))) OR ("Depressive Disorder, Major" OR "Depression" OR (Depress* or MDD)) OR ("Anxiety Disorders" OR "Anxiety" OR anxi*) OR ("Phobic Disorders" OR phobi*) OR ("Schizophrenia" Or (schizophreni* or hebephreni*)) OR ("Somatoform Disorders" OR "Medically Unexplained Symptoms" OR (((somatoform* or somati* or (medically AND unexplained) or briquet or pain) AND (disorder* or syndrome* or symptom*)))) OR ("Dissociative Disorders" OR (dissociative AND (disorder* or hysteri* or reaction*)) or dissociation*) OR ("Hysteria" or hysteri*) OR ("Mood Disorders" OR ((affective* or mood*) AND (disorder* or disease* or illness* or symptom*))) OR ("Stress Disorders, Post-Traumatic" OR (PTSD or ((posttrauma* or trauma*) AND (stress* or neurose*)) or "combat disorder" or "war disorder")) OR ("Cognition Disorders" OR (((cognitive or cognition or mental or neurocognitive) AND (dysfunction* or decline* or impairment* or deterioration* or disorder* or illness* or disease*)))) OR ("Personality Disorders" OR "personality disorder") OR ("Disruptive, Impulse Control, and Conduct Disorders" OR ("impulse control disorder" or "intermittent explosive disorder")) OR ("Feeding and Eating Disorders" OR ((eating or appetite or feeding) AND disorder*)) OR ("Bipolar Disorder" OR ((bipolar or mani*) AND (disorder* or illness* or disease*))) OR ("Obsessive-Compulsive Disorder" OR OCD* or ((obsess* or compulsi*) AND (disorder* or illness* or disease* or neuros*))) OR ("Panic Disorder" OR ((panic AND (attack* or disorder*)))) OR ("Agoraphobia" OR agoraphobi*) OR ("Neurotic Disorders" OR (neuros* or "neurotic disorder" or psychoneuros*))) OR (("Communicable Diseases" OR (((communic* or contag* or transmi* or infect*) AND (disease* or infection* or illness*)))) OR ("Bacterial Infections" OR (bacteri* infection*)) OR ("Conjunctivitis") OR ("HIV" OR "Human immuno deficiency virus") OR ("Acquired Immunodeficiency Syndrome" OR (AIDS or "immunodeficiency associated virus" or (immun* deficiency associated virus) or (acquired immunodeficiency syndrome*) or (acquired immun* deficiency syndrome*))) OR ("Buruli Ulcer" OR (Bairnsdale or Buruli)) OR ("Onchocerciasis" OR (onchocer*)) OR ("Hepatitis B" OR "Hepatitis C" OR (hepatitis)) OR ("Leishmaniasis" OR (leishmania*)) OR ("Leprosy" OR (lepros* or hansen*)) OR ("Elephantiasis, Filarial" OR (elephantias* or filaria*)) OR ("Trachoma" OR ((egyptian ophthalmia*) or trachoma*)) OR ("Chikungunya Fever" OR (chickungunya or chikungunya)) OR ("Taeniasis" OR taenia*) OR ("Cysticercosis" OR cysticercos*) OR ("Echinococcosis" OR (hydatid or echinococc*)) OR ("Chagas Disease" OR (trypanosom* or chagas)) OR ("Trypanosomiasis" OR ("sleeping sickness")) OR ("Encephalitis, Japanese" OR (japanese encephalitis)) OR ("Syphilis"))) AND ("Tuberculosis" OR Tuberculos* OR TB OR koch*)) AND ("Systematic Review" OR (systematic AND review*) OR "Meta-Analysis" OR meta-analys*)
3

For peer review only

PROSPERO (07/10/2020)

(((((Non-communicable or Noncommunicable or Non-infectious) AnD (disease* or condition* or illness*))) oR ((MeSH DESCRIPTOR Chronic Disease EXPLODE ALL TREES) or ((chronic or long-term) AnD (disease* or condition* or illness*))) oR ((MeSH DESCRIPTOR Heart Diseases EXPLODE ALL TREES) or ((heart AnD (disease* or disorder* or failure)) or (cardiac AnD (disease* or disorder* or failure)))) oR ((MeSH DESCRIPTOR Cardiovascular Diseases EXPLODE ALL TREES) OR ((cardiovascular AnD (disease* or disorder* or failure)))) oR ((MeSH DESCRIPTOR Coronary Disease EXPLODE ALL TREES) OR ((coronary AnD (disease* or disorder* or failure)))) oR ((MeSH DESCRIPTOR Cerebrovascular Disorders EXPLODE ALL TREES) OR ((cerebrovascular AnD (disease* or disorder* or insufficienc* or occlusion*))) OR ((vascular AnD (disease* or disorder*))) OR ((carotid* AnD (disease* or disorder*)))) oR ((MeSH DESCRIPTOR Peripheral Arterial Disease EXPLODE ALL TREES) OR ((arter* AnD (disease* or disorder*)))) oR ((MeSH DESCRIPTOR Rheumatic Heart Disease EXPLODE ALL TREES) OR (MeSH DESCRIPTOR Heart Defects, Congenital EXPLODE ALL TREES) or ((heart AnD (malform* or defect* or congeni*)))) oR ((MeSH DESCRIPTOR Venous Thrombosis EXPLODE ALL TREES) OR (((deep vein or deep venous) AnD thrombos*)) OR (phlebothrombos*)) oR ((MeSH DESCRIPTOR Pulmonary Embolism EXPLODE ALL TREES) OR ((pulmonar* AnD (thromboembolism* or embolism* or disease* or disorder*)))) oR ((MeSH DESCRIPTOR Stroke EXPLODE ALL TREES) OR (stroke)) oR ((MeSH DESCRIPTOR Neoplasms EXPLODE ALL TREES) OR (Cancer*) OR (neoplas*) OR (tumor*)) oR ((MeSH DESCRIPTOR Lung Diseases EXPLODE ALL TREES) OR (MeSH DESCRIPTOR Respiratory Tract Diseases EXPLODE ALL TREES) OR (MeSH DESCRIPTOR Lung Diseases, Obstructive EXPLODE ALL TREES) OR (((lung* or respiratory or pulmonar* or airflow or airway) AnD (disease* or obstruct* or hypersensitiv*))) OR (MeSH DESCRIPTOR Asthma EXPLODE ALL TREES) Or (asthma*) OR (MeSH DESCRIPTOR Pulmonary Disease, Chronic Obstructive EXPLODE ALL TREES) OR (MeSH DESCRIPTOR Respiratory Hypersensitivity EXPLODE ALL TREES)) oR ((MeSH DESCRIPTOR Diabetes Mellitus EXPLODE ALL TREES) OR (diabet*)) oR ((MeSH DESCRIPTOR Autoimmune Diseases EXPLODE ALL TREES) OR (((autoimmun* or auto immun* or autoaggress* or auto aggress*) AnD (disorder* or disease*)))) oR ((MeSH DESCRIPTOR Metabolic Syndrome X EXPLODE ALL TREES) OR (MeSH DESCRIPTOR Metabolic Diseases EXPLODE ALL TREES) OR (((metabolic or insulin resistance) AnD (disorder* or disease* or syndrome*)))) oR ((MeSH DESCRIPTOR Obesity EXPLODE ALL TREES) OR (obes*)) oR ((MeSH DESCRIPTOR Osteoporosis EXPLODE ALL TREES) OR (osteoporo* or bone loss) OR (MeSH DESCRIPTOR Osteolysis EXPLODE ALL TREES) OR (osteolysis or "bone resorption")) oR ((MeSH DESCRIPTOR Parkinson Disease EXPLODE ALL TREES) OR (parkinson* or "paralysis agitans")) oR ((MeSH DESCRIPTOR Arthritis EXPLODE ALL TREES) OR (arthriti* or polyarthriti* or rheumarthriti*)) oR ((MeSH DESCRIPTOR Kidney Diseases EXPLODE ALL TREES) OR ((kidney AnD (disease* or disorder*)))) oR ((MeSH DESCRIPTOR Liver Diseases EXPLODE ALL TREES) OR ((liver AnD (disease* or disorder* or dysfunction*)))) oR ((MeSH DESCRIPTOR Hypertension EXPLODE ALL TREES) OR (high blood pressure* or hypertens*)) oR ((MeSH DESCRIPTOR Hyperlipidemias EXPLODE ALL TREES) OR (hyperlipem* or hyperlipidem* or lipem* or lipidem*)) oR ((MeSH DESCRIPTOR Hypercholesterolemia EXPLODE ALL TREES) OR (((high* or elevat*) AnD cholesterol*)) OR (hypercholesterem* or hypercholesterolem*)) oR ((MeSH DESCRIPTOR Hypertriglyceridemia EXPLODE ALL TREES) OR (hypertriglyceridem*)) oR ((MeSH DESCRIPTOR Thyroid Diseases EXPLODE ALL TREES) OR ((thyroid AnD (disease* or disorder*))) OR (MeSH DESCRIPTOR Hyperthyroidism EXPLODE ALL TREES) OR (hypothyroid*) OR (((thyroid-stimulating hormone* or tsh) AnD deficien*))) oR ((MeSH DESCRIPTOR Motor Neuron Disease EXPLODE ALL TREES) OR (motor neuron* disease*) Or (lateral scleros*) OR (motor system disease*)) oR ((MeSH DESCRIPTOR Multiple Sclerosis EXPLODE ALL TREES) OR ("multiple sclerosis" or

For peer review only

"disseminated sclerosis")) oR ((MeSH DESCRIPTOR Emphysema EXPLODE ALL TREES) OR (emphysema*)) oR ((MeSH DESCRIPTOR Bronchitis EXPLODE ALL TREES) OR (bronchit*))) OR (((MeSH DESCRIPTOR Mental Disorders EXPLODE ALL TREES) OR (MeSH DESCRIPTOR Psychotic Disorders EXPLODE ALL TREES) OR (((mental* or psychiatr* or psycho*) AnD (disorder* or disease* or illness*)))) oR ((MeSH DESCRIPTOR Depressive Disorder, Major EXPLODE ALL TREES) OR (MeSH DESCRIPTOR Depression EXPLODE ALL TREES) OR (Depress* or MDD)) oR ((MeSH DESCRIPTOR Anxiety Disorders EXPLODE ALL TREES) oR (MeSH DESCRIPTOR Anxiety EXPLODE ALL TREES) OR (anxi*)) oR ((MeSH DESCRIPTOR Phobic Disorders EXPLODE ALL TREES) OR (phobi*)) oR ((MeSH DESCRIPTOR Schizophrenia EXPLODE ALL TREES) OR (schizophreni* or hebephreni*)) oR ((MeSH DESCRIPTOR Somatoform Disorders EXPLODE ALL TREES) OR (((somatoform* or somati* or "medically unexplained" or briquet or pain) AnD (disorder* or syndrome* or symptom*))) or (MeSH DESCRIPTOR Medically Unexplained Symptoms EXPLODE ALL TREES)) oR ((MeSH DESCRIPTOR Dissociative Disorders EXPLODE ALL TREES) OR ((dissociative AnD (disorder* or hysteri* or reaction*))) OR (dissociation*)) oR ((MeSH DESCRIPTOR Hysteria EXPLODE ALL TREES) OR (hysteri*)) oR ((MeSH DESCRIPTOR Mood Disorders EXPLODE ALL TREES) OR (((affective* or mood*) AnD (disorder* or disease* or illness* or symptom*)))) oR ((MeSH DESCRIPTOR Stress Disorders, Post-Traumatic EXPLODE ALL TREES) or (PTSD) OR (((post trauma* or posttrauma*) AnD (stress* or neurose*))) or ("combat disorder") OR ("war disorder")) oR ((MeSH DESCRIPTOR Cognition Disorders EXPLODE ALL TREES) OR (((cognitive or cognition or mental or neurocognitive) AnD (dysfunction* or decline* or impairment* or deterioration* or disorder* or illness* or disease*)))) oR ((MeSH DESCRIPTOR Personality Disorders EXPLODE ALL TREES) OR ("personality disorder") OR ("personality disorders")) oR ((impulse control disorder*) OR (intermittent explosive disorder*)) oR ((MeSH DESCRIPTOR Feeding and Eating Disorders EXPLODE ALL TREES) OR (((eating or appetite or feeding) AnD disorder*))) oR ((MeSH DESCRIPTOR Bipolar Disorder EXPLODE ALL TREES) OR (((bipolar or mani*) AnD (disorder* or illness* or disease*)))) oR ((MeSH DESCRIPTOR Obsessive-Compulsive Disorder EXPLODE ALL TREES) OR (OCD*) OR (((obsess*-compulsi* or obsess* or compulsi*) AnD (disorder* or illness* or disease* or neuros*)))) oR ((MeSH DESCRIPTOR Panic Disorder EXPLODE ALL TREES) OR ((panic AnD (attack* or disorder*)))) oR ((MeSH DESCRIPTOR Agoraphobia EXPLODE ALL TREES) OR (agoraphobi*)) oR ((MeSH DESCRIPTOR Neurotic Disorders EXPLODE ALL TREES) OR (neuros* or "neurotic disorder" or psychoneuros*))) OR (((MeSH DESCRIPTOR Communicable Diseases EXPLODE ALL TREES) OR (((communic* or contag* or transmi* or infect*) AnD (disease* or infection* or illness*)))) oR ((MeSH DESCRIPTOR Bacterial Infections EXPLODE ALL TREES) OR (bacteri* infection*)) oR ((MeSH DESCRIPTOR Conjunctivitis EXPLODE ALL TREES) OR (conjunctivitis)) oR ((MeSH DESCRIPTOR HIV EXPLODE ALL TREES) OR (hiv or "Human immuno deficiency virus")) oR ((MeSH DESCRIPTOR Acquired Immunodeficiency Syndrome EXPLODE ALL TREES) OR (AIDS) OR ("immunodeficiency associated virus") OR (immun* AND "deficiency associated virus") OR ("acquired immunodeficiency syndrome")) oR ((MeSH DESCRIPTOR Buruli Ulcer EXPLODE ALL TREES) OR (Bairnsdale or Buruli)) oR ((MeSH DESCRIPTOR Onchocerciasis EXPLODE ALL TREES) OR (onchocer*)) oR ((MeSH DESCRIPTOR Hepatitis B EXPLODE ALL TREES) OR (MeSH DESCRIPTOR Hepatitis C EXPLODE ALL TREES) OR (hepatitis)) oR ((MeSH DESCRIPTOR Leishmaniasis EXPLODE ALL TREES) OR (leishmania*)) oR ((MeSH DESCRIPTOR Leprosy EXPLODE ALL TREES) OR (lepros* or hansen*)) oR ((MeSH DESCRIPTOR Elephantiasis, Filarial EXPLODE ALL TREES) OR (elephantias* or filaria*)) oR ((MeSH DESCRIPTOR Trachoma EXPLODE ALL TREES) OR ("egyptian ophthalmia" or trachoma*)) oR ((MeSH DESCRIPTOR Chikungunya Fever EXPLODE ALL TREES) OR (chickungunya or chikungunya)) oR ((MeSH DESCRIPTOR Taeniasis EXPLODE ALL TREES) OR taenia*) oR ((MeSH DESCRIPTOR Cysticercosis EXPLODE ALL TREES) OR (cysticercos*)) oR ((MeSH DESCRIPTOR Echinococcosis EXPLODE ALL TREES)

For peer review only

OR (hydatid* or echinococc*)) oR ((MeSH DESCRIPTOR Chagas Disease EXPLODE ALL TREES) OR (trypanosom* or chagas)) oR ((MeSH DESCRIPTOR Trypanosomiasis EXPLODE ALL TREES) OR ("sleeping sickness")) oR ((MeSH DESCRIPTOR Encephalitis, Japanese EXPLODE ALL TREES) OR (japanese AnD encephalitis)) oR ((MeSH DESCRIPTOR Syphilis EXPLODE ALL TREES) OR (syphilis)))) AND ((MeSH DESCRIPTOR Tuberculosis EXPLODE ALL TREES) OR (Tuberculos*) OR (TB) OR (koch*))
1341

For peer review only

APPENDIX 1: References of included studies

S1 Huddart S, Svadzian A, Nafade V, *et al.* Tuberculosis case fatality in India: a systematic review and meta-analysis. *BMJ Glob Health* 2020;**5**:e002080. doi:10.1136/bmjgh-2019-002080

S2 Eshetie S, Gizachew M, Alebel A, *et al.* Tuberculosis treatment outcomes in Ethiopia from 2003 to 2016, and impact of HIV co-infection and prior drug exposure: A systematic review and meta-analysis. *PloS One* 2018;**13**:e0194675. doi:10.1371/journal.pone.0194675

S3 Ruiz-Grosso P, Cachay R, de la Flor A, *et al.* Association between tuberculosis and depression on negative outcomes of tuberculosis treatment: A systematic review and meta-analysis. *PloS One* 2020;**15**:e0227472. doi:10.1371/journal.pone.0227472

S4 Gautam S, Shrestha N, Mahato S, *et al.* Diabetes among tuberculosis patients and its impact on tuberculosis treatment in South Asia: a systematic review and meta-analysis. *Sci Rep* 2021;**11**:2113. doi:10.1038/s41598-021-81057-2

S5 Chem ED, Van Hout MC, Hope V. Treatment outcomes and antiretroviral uptake in multidrug-resistant tuberculosis and HIV co-infected patients in Sub Saharan Africa: a systematic review and meta-analysis. *BMC Infect Dis* 2019;**19**:723. doi:10.1186/s12879-019-4317-4

S6 Edessa D, Sisay M, Dessie Y. Unfavorable outcomes to second-line tuberculosis therapy among HIV-infected versus HIV-uninfected patients in sub-Saharan Africa: A systematic review and meta-analysis. *PloS One* 2020;**15**:e0237534. doi:10.1371/journal.pone.0237534

S7 Endalamaw A, Ambachew S, Geremew D, *et al.* HIV infection and unknown HIV status among tuberculosis patients in Ethiopia: a systematic review and meta-analysis. *Int J Tuberc Lung Dis Off J Int Union Tuberc Lung Dis* 2019;**23**:187–94. doi:10.5588/ijtld.18.0363

S8 Gao L, Zhou F, Li X, *et al.* HIV/TB co-infection in mainland China: a meta-analysis. *PloS One* 2010;**5**:e10736. doi:10.1371/journal.pone.0010736

S9 Gao J, Zheng P, Fu H. Prevalence of TB/HIV co-infection in countries except China: a systematic review and meta-analysis. *PloS One* 2013;**8**:e64915. doi:10.1371/journal.pone.0064915

S10 Gelaw YA, Williams G, Soares Magalhaes RJ, *et al.* HIV Prevalence Among Tuberculosis Patients in Sub-Saharan Africa: A Systematic Review and Meta-analysis. *AIDS Behav* 2019;**23**:1561–75. doi:10.1007/s10461-018-02386-4

S11 Mekonnen D, Derbie A, Abeje A, *et al.* Epidemiology of tuberculous lymphadenitis in Africa: A systematic review and meta-analysis. *PloS One* 2019;**14**:e0215647. doi:10.1371/journal.pone.0215647

S12 Samuels JP, Sood A, Campbell JR, *et al.* Comorbidities and treatment outcomes in multidrug resistant tuberculosis: a systematic review and meta-analysis. *Sci Rep* 2018;**8**:4980. doi:10.1038/s41598-018-23344-z

S13 Wang M-G, Luo L, Zhang Y, *et al.* Treatment outcomes of tuberculous meningitis in adults: a systematic review and meta-analysis. *BMC Pulm Med* 2019;**19**:200. doi:10.1186/s12890-019-0966-8

S14 Alebel A, Wondemagegn AT, Tesema C, *et al.* Prevalence of diabetes mellitus among tuberculosis patients in Sub-Saharan Africa: a systematic review and meta-analysis of observational studies. *BMC Infect Dis* 2019;**19**:254. doi:10.1186/s12879-019-3892-8

S15 Chen H, Liu M, Gu F. [Meta-analysis on the co-morbidity rate between tuberculosis and diabetes mellitus in China]. *Zhonghua Liu Xing Bing Xue Za Zhi Zhonghua Liuxingbingxue Zazhi* 2013;**34**:1128–33.

For peer review only - http://bmjopen.bmj.com/site/about/guidelines.xhtml

S16 Huangfu P, Ugarte-Gil C, Golub J, *et al.* The effects of diabetes on tuberculosis treatment outcomes: an updated systematic review and meta-analysis. *Int J Tuberc Lung Dis Off J Int Union Tuberc Lung Dis* 2019;**23**:783–96. doi:10.5588/ijtld.18.0433

S17 Noubiap JJ, Nansseu JR, Nyaga UF, *et al.* Global prevalence of diabetes in active tuberculosis: a systematic review and meta-analysis of data from 2.3 million patients with tuberculosis. *Lancet Glob Health* 2019;**7**:e448–60. doi:10.1016/S2214-109X(18)30487-X

S18 S.H Y, M.-L D, D J, *et al.* Risk factors of retreatment pulmonary tuberculosis patients with unfavorable treatment outcome in China: A meta-analysis. *Chin J Evid-Based Med* 2016;**16**:795–801.

S19 Alene KA, Clements ACA, McBryde ES, *et al.* Mental health disorders, social stressors, and health-related quality of life in patients with multidrug-resistant tuberculosis: A systematic review and meta-analysis. *J Infect* 2018;**77**:357–67. doi:10.1016/j.jinf.2018.07.007

S20 Duko B, Bedaso A, Ayano G. The prevalence of depression among patients with tuberculosis: a systematic review and meta-analysis. *Ann Gen Psychiatry* 2020;**19**:30. doi:10.1186/s12991-020-00281-8

S21 Ga Eun Lee JS Jerome Galea, Sanghyuk Shin, Annika Sweetland, Elizabeth Magill. Impact of mental illness on active tuberculosis (TB) treatment outcome: a systematic review and meta-analysis.

S22 Behzadifar M, Heydarvand S, Behzadifar M, *et al.* Prevalence of Hepatitis C Virus in Tuberculosis Patients: A Systematic Review and Meta-Analysis. *Ethiop J Health Sci* 2019;**29**:945–56. doi:10.4314/ejhs.v29i1.17

S23 C.Y L, H.-L H, M.M R, *et al.* Cancer incidence attributable to tuberculosis in 2015: Global, regional, and national estimates. *BMC Cancer* 2020;**20**:412.

S24 Arega B, Minda A, Mengistu G, *et al.* Unknown HIV status and the TB/HIV collaborative control program in Ethiopia: systematic review and meta-analysis. *BMC Public Health* 2020;**20**:1021. doi:10.1186/s12889-020-09117-2

S25 Bastos SH, Taminato M, Fernandes H, *et al.* Sociodemographic and health profile of TB/HIV co-infection in Brazil: a systematic review. *Rev Bras Enferm* 2019;**72**:1389–96. doi:10.1590/0034-7167-2018-0285

S26 Lukoye D, Ssengooba W, Musisi K, *et al.* Variation and risk factors of drug resistant tuberculosis in sub-Saharan Africa: a systematic review and meta-analysis. *BMC Public Health* 2015;**15**:291. doi:10.1186/s12889-015-1614-8

S27 McMurry HS, Mendenhall E, Rajendrakumar A, *et al.* Coprevalence of type 2 diabetes mellitus and tuberculosis in low-income and middle-income countries: A systematic review. *Diabetes Metab Res Rev* 2019;**35**:e3066. doi:10.1002/dmrr.3066

S28 Pourakbari B, Mamishi S, Banar M, *et al.* Prevalence of TB/ HIV co-infection in Iran: a systematic review and meta-analysis. *Ann Ig Med Prev E Comunita* 2019;**31**:333–48. doi:10.7416/ai.2019.2295

S29 Rajendran M, Zaki RA, Aghamohammadi N. Contributing risk factors towards the prevalence of multidrug-resistant tuberculosis in Malaysia: A systematic review. *Tuberc Edinb Scotl* 2020;**122**:101925. doi:10.1016/j.tube.2020.101925

S30 Reddy EA, Shaw AV, Crump JA. Community-acquired bloodstream infections in Africa: a systematic review and meta-analysis. *Lancet Infect Dis* 2010;**10**:417–32. doi:10.1016/S1473-3099(10)70072-4

S31 Seid MA, Ayalew MB, Muche EA, *et al.* Drug-susceptible tuberculosis treatment success and associated factors in Ethiopia from 2005 to 2017: a systematic review and meta-analysis. *BMJ Open* 2018;**8**:e022111. doi:10.1136/bmjopen-2018-022111

For peer review only - http://bmjopen.bmj.com/site/about/guidelines.xhtml

S32 Straetemans M, Glaziou P, Bierrenbach AL, *et al.* Assessing tuberculosis case fatality ratio: a meta-analysis. *PloS One* 2011;**6**:e20755. doi:10.1371/journal.pone.0020755

S33 Tesfaye B, Alebel A, Gebrie A, *et al.* The twin epidemics: Prevalence of TB/HIV co-infection and its associated factors in Ethiopia; A systematic review and meta-analysis. *PloS One* 2018;**13**:e0203986. doi:10.1371/journal.pone.0203986

S34 Teweldemedhin M, Asres N, Gebreyesus H, *et al.* Tuberculosis-Human Immunodeficiency Virus (HIV) co-infection in Ethiopia: a systematic review and meta-analysis. *BMC Infect Dis* 2018;**18**:676. doi:10.1186/s12879-018-3604-9

S35 Uchida S, Komiya K, Honjo K, *et al.* A mini systematic review of prognostic factors in elderly patients with tuberculosis. *Respir Investig* 2019;**57**:207–12. doi:10.1016/j.resinv.2018.12.004

S36 Waitt CJ, Squire SB. A systematic review of risk factors for death in adults during and after tuberculosis treatment. *Int J Tuberc Lung Dis Off J Int Union Tuberc Lung Dis* 2011;**15**:871–85. doi:10.5588/ijtld.10.0352

S37 Baker MA, Harries AD, Jeon CY, *et al.* The impact of diabetes on tuberculosis treatment outcomes: a systematic review. *BMC Med* 2011;**9**:81. doi:10.1186/1741-7015-9-81

S38 Han X, Wang Q, Wang Y, *et al.* The impact of diabetes on tuberculosis treatment outcomes: evidence based on a cumulative meta-analysis. *Int J DIABETES Dev Ctries* 2016;**36**:490–507. doi:10.1007/s13410-016-0514-5

S39 Huang D, Wang Y, Wang Y, *et al.* The impact of diabetes mellitus on drug resistance in patients with newly diagnosed tuberculosis: a systematic review and meta-analysis. *Ann Palliat Med* 2020;**9**:152–62. doi:10.21037/apm.2020.02.16

S40 Jeon CY, Harries AD, Baker MA, *et al.* Bi-directional screening for tuberculosis and diabetes: a systematic review. *Trop Med Int Health TM IH* 2010;**15**:1300–14. doi:10.1111/j.1365-3156.2010.02632.x

S41 Lutfiana NC, van Boven JFM, Masoom Zubair MA, *et al.* Diabetes mellitus comorbidity in patients enrolled in tuberculosis drug efficacy trials around the world: A systematic review. *Br J Clin Pharmacol* 2019;**85**:1407–17. doi:10.1111/bcp.13935

S42 Tegegne BS, Mengesha MM, Teferra AA, *et al.* Association between diabetes mellitus and multi-drug-resistant tuberculosis: evidence from a systematic review and meta-analysis. *Syst Rev* 2018;**7**:161. doi:10.1186/s13643-018-0828-0

S43 Workneh MH, Bjune GA, Yimer SA. Prevalence and associated factors of tuberculosis and diabetes mellitus comorbidity: A systematic review. *PloS One* 2017;**12**:e0175925. doi:10.1371/journal.pone.0175925

S44 A JVR, A D, R C, *et al.* Comorbidities between tuberculosis and common mental disorders: A scoping review of epidemiological patterns and person-centred care interventions from low-to-middle income and BRICS countries. *Infect Dis Poverty* 2020;**9**:4.

S45 Basham CA, Smith SJ, Romanowski K, *et al.* Cardiovascular morbidity and mortality among persons diagnosed with tuberculosis: A systematic review and meta-analysis. *PloS One* 2020;**15**:e0235821. doi:10.1371/journal.pone.0235821

S46 Rehm J, Samokhvalov AV, Neuman MG, *et al.* The association between alcohol use, alcohol use disorders and tuberculosis (TB). A systematic review. *BMC Public Health* 2009;**9**:450. doi:10.1186/1471-2458-9-450

S47 Sotgiu G, Ferrara G, Matteelli A, *et al.* Epidemiology and clinical management of XDR-TB: a systematic review by TBNET. *Eur Respir J* 2009;**33**:871–81. doi:10.1183/09031936.00168008

For peer review only - http://bmjopen.bmj.com/site/about/guidelines.xhtml

S48 Bisson GP, Bastos M, Campbell JR, *et al.* Mortality in adults with multidrug-resistant tuberculosis and HIV by antiretroviral therapy and tuberculosis drug use: an individual patient data meta-analysis. *Lancet Lond Engl* 2020;**396**:402–11. doi:10.1016/S0140-6736(20)31316-7

S49 Mesfin YM, Hailemariam D, Biadgilign S, *et al.* Association between HIV/AIDS and multi-drug resistance tuberculosis: a systematic review and meta-analysis. *PloS One* 2014;**9**:e82235. doi:10.1371/journal.pone.0082235

S50 Wu S, Zhang Y, Sun F, *et al.* Adverse Events Associated With the Treatment of Multidrug-Resistant Tuberculosis: A Systematic Review and Meta-analysis. *Am J Ther* 2016;**23**:e521-30. doi:10.1097/01.mjt.0000433951.09030.5a

S51 de Almeida CPB, Ziegelmann PK, Couban R, *et al.* Predictors of In-Hospital Mortality among Patients with Pulmonary Tuberculosis: A Systematic Review and Meta-analysis. *Sci Rep* 2018;**8**:7230. doi:10.1038/s41598-018-25409-5

S52 Pormohammad A, Nasiri MJ, Riahi SM, *et al.* Human immunodeficiency virus in patients with tuberculous meningitis: systematic review and meta-analysis. *Trop Med Int Health TM IH* 2018;**23**:589–95. doi:10.1111/tmi.13059

S53 Purmohamad A, Azimi T, Nasiri MJ, *et al.* HIV-Tuberculous Meningitis Co-infection: A Systematic Review and Meta-analysis. *Curr Pharm Biotechnol* Published Online First: 2020. doi:10.2174/1389201021666200730143906

S54 Alemu A, Bitew ZW, Worku T. Poor treatment outcome and its predictors among drug-resistant tuberculosis patients in Ethiopia: A systematic review and meta-analysis. *Int J Infect Dis IJID Off Publ Int Soc Infect Dis* 2020;**98**:420–39. doi:10.1016/j.ijid.2020.05.087

S55 Wongtrakul W, Charoenngam N, Ungprasert P. Tuberculosis and risk of coronary heart disease: A systematic review and meta-analysis. *Indian J Tuberc* 2020;**67**:182–8. doi:10.1016/j.ijtb.2020.01.008

For peer review only

For peer review only - http://bmjopen.bmj.com/site/about/guidelines.xhtml

PRISMA 2020 Checklist

Section and Topic	Item #	Checklist item	Location where item is reported
**TITLE**			
Title	1	Identify the report as a systematic review.	
**ABSTRACT**			
Abstract	2	See the PRISMA 2020 for Abstracts checklist.	
**INTRODUCTION**			
Rationale	3	Describe the rationale for the review in the context of existing knowledge.	
Objectives	4	Provide an explicit statement of the objective(s) or question(s) the review addresses.	
**METHODS**			
Eligibility criteria	5	Specify the inclusion and exclusion criteria for the review and how studies were grouped for the syntheses.	
Information sources	6	Specify all databases, registers, websites, organisations, reference lists and other sources searched or consulted to identify studies. Specify the date when each source was last searched or consulted.	
Search strategy	7	Present the full search strategies for all databases, registers and websites, including any filters and limits used.	
Selection process	8	Specify the methods used to decide whether a study met the inclusion criteria of the review, including how many reviewers screened each record and each report retrieved, whether they worked independently, and if applicable, details of automation tools used in the process.	
Data collection process	9	Specify the methods used to collect data from reports, including how many reviewers collected data from each report, whether they worked independently, any processes for obtaining or confirming data from study investigators, and if applicable, details of automation tools used in the process.	
Data items	10a	List and define all outcomes for which data were sought. Specify whether all results that were compatible with each outcome domain in each study were sought (e.g. for all measures, time points, analyses), and if not, the methods used to decide which results to collect.	
	10b	List and define all other variables for which data were sought (e.g. participant and intervention characteristics, funding sources). Describe any assumptions made about any missing or unclear information.	
Study risk of bias assessment	11	Specify the methods used to assess risk of bias in the included studies, including details of the tool(s) used, how many reviewers assessed each study and whether they worked independently, and if applicable, details of automation tools used in the process.	
Effect measures	12	Specify for each outcome the effect measure(s) (e.g. risk ratio, mean difference) used in the synthesis or presentation of results.	
Synthesis methods	13a	Describe the processes used to decide which studies were eligible for each synthesis (e.g. tabulating the study intervention characteristics and comparing against the planned groups for each synthesis (item #5)).	
	13b	Describe any methods required to prepare the data for presentation or synthesis, such as handling of missing summary statistics, or data conversions.	
	13c	Describe any methods used to tabulate or visually display results of individual studies and syntheses.	
	13d	Describe any methods used to synthesize results and provide a rationale for the choice(s). If meta-analysis was performed, describe the model(s), method(s) to identify the presence and extent of statistical heterogeneity, and software package(s) used.	
	13e	Describe any methods used to explore possible causes of heterogeneity among study results (e.g. subgroup analysis, meta-regression).	
	13f	Describe any sensitivity analyses conducted to assess robustness of the synthesized results.	
Reporting bias assessment	14	Describe any methods used to assess risk of bias due to missing results in a synthesis (arising from reporting biases).	
Certainty assessment	15	Describe any methods used to assess certainty (or confidence) in the body of evidence for an outcome.	

PRISMA 2020 Checklist

Section and Topic	Item #	Checklist item	Location where item is reported
**RESULTS**			
Study selection	16a	Describe the results of the search and selection process, from the number of records identified in the search to the number of studies included in the review, ideally using a flow diagram.	
	16b	Cite studies that might appear to meet the inclusion criteria, but which were excluded, and explain why they were excluded.	
Study characteristics	17	Cite each included study and present its characteristics.	
Risk of bias in studies	18	Present assessments of risk of bias for each included study.	
Results of individual studies	19	For all outcomes, present, for each study: (a) summary statistics for each group (where appropriate) and (b) an effect estimate and its precision (e.g. confidence/credible interval), ideally using structured tables or plots.	
Results of syntheses	20a	For each synthesis, briefly summarise the characteristics and risk of bias among contributing studies.	
	20b	Present results of all statistical syntheses conducted. If meta-analysis was done, present for each the summary estimate and its precision (e.g. confidence/credible interval) and measures of statistical heterogeneity. If comparing groups, describe the direction of the effect.	
	20c	Present results of all investigations of possible causes of heterogeneity among study results.	
	20d	Present results of all sensitivity analyses conducted to assess the robustness of the synthesized results.	
Reporting biases	21	Present assessments of risk of bias due to missing results (arising from reporting biases) for each synthesis assessed.	
Certainty of evidence	22	Present assessments of certainty (or confidence) in the body of evidence for each outcome assessed.	
**DISCUSSION**			
Discussion	23a	Provide a general interpretation of the results in the context of other evidence.	
	23b	Discuss any limitations of the evidence included in the review.	
	23c	Discuss any limitations of the review processes used.	
	23d	Discuss implications of the results for practice, policy, and future research.	
**OTHER INFORMATION**			
Registration and protocol	24a	Provide registration information for the review, including register name and registration number, or state that the review was not registered.	
	24b	Indicate where the review protocol can be accessed, or state that a protocol was not prepared.	
	24c	Describe and explain any amendments to information provided at registration or in the protocol.	
Support	25	Describe sources of financial or non-financial support for the review, and the role of the funders or sponsors in the review.	
Competing interests	26	Declare any competing interests of review authors.	
Availability of data, code and other materials	27	Report which of the following are publicly available and where they can be found: template data collection forms; data extracted from included studies; data used for all analyses; analytic code; any other materials used in the review.	

From: Page MJ, McKenzie JE, Bossuyt PM, Boutron I, Hoffmann TC, Mulrow CD, et al. The PRISMA 2020 statement: an updated guideline for reporting systematic reviews. BMJ 2021;372:n71. doi: 10.1136/bmj.n71

For peer review only - http://bmjopen.bmj.com/site/about/guidelines.xhtml

For more information, visit: http://www.prisma-statement.org/

For peer review only
